# The Ratiometric Transcript Signature MX2/GPR183 Is Consistently Associated With RTS,S-Mediated Protection Against Controlled Human Malaria Infection

**DOI:** 10.3389/fimmu.2020.00669

**Published:** 2020-04-28

**Authors:** Ying Du, Ethan G. Thompson, Julius Muller, Joseph Valvo, Jackie Braun, Smitha Shankar, Robert A. van den Berg, Erik Jongert, Drew Dover, Jerald Sadoff, Jenny Hendriks, Malcolm J. Gardner, W. Ripley Ballou, Jason A. Regules, Robbert van der Most, Alan Aderem, Christian F. Ockenhouse, Adrian V. Hill, Ulrike Wille-Reece, Daniel E. Zak

**Affiliations:** ^1^Center for Global Infectious Disease Research, Seattle Children's Research Institute, Seattle, WA, United States; ^2^Center for Infectious Disease Research, Seattle, WA, United States; ^3^The Jenner Institute, Oxford, United Kingdom; ^4^GSK Vaccines, Rockville, MD, United States; ^5^GSK Vaccines, Rixensart, Belgium; ^6^Janssen Vaccines and Prevention BV, Leiden, Netherlands; ^7^Infectious Diseases, J. Craig Venter Institute, La Jolla, CA, United States; ^8^Walter Reed Army Institute of Research, Silver Spring, MD, United States; ^9^PATH's Malaria Vaccine Initiative, Washington, DC, United States

**Keywords:** malaria vaccines, clinical immunology, vaccine correlates, human challenge, systems vaccinology, interferon response

## Abstract

The RTS,S/AS01 vaccine provides partial protection against *Plasmodium falciparum* infection but determinants of protection and/or disease are unclear. Previously, anti-circumsporozoite protein (CSP) antibody titers and blood RNA signatures were associated with RTS,S/AS01 efficacy against controlled human malaria infection (CHMI). By analyzing host blood transcriptomes from five RTS,S vaccination CHMI studies, we demonstrate that the transcript ratio MX2/GPR183, measured 1 day after third immunization, discriminates protected from non-protected individuals. This ratiometric signature provides information that is complementary to anti-CSP titer levels for identifying RTS,S/AS01 immunized people who developed protective immunity and suggests a role for interferon and oxysterol signaling in the RTS,S mode of action.

## Background

Considerable progress has been made in the development of malaria vaccines (1). The most clinically advanced of these is subunit-based RTS,S/AS01 (“RTS,S”), which protects against infection in controlled human malaria infection (CHMI) studies (2–6) and against malaria disease in clinical trials in Africa (7). While anti-circumsporozoite protein (CSP) antibodies correlate with early protection (8), robust correlates of RTS,S efficacy have not been defined. Deep immunological analysis of controlled human infection models provides an ideal setting for the delineation of correlates of vaccine efficacy (9). In the specific case of malaria, CHMI studies capture a subset of mechanisms that impact malaria vaccine efficacy in practice and therefore constitute a crucial translational bridge for rapidly transitioning novel malaria vaccine concepts from controlled animal models to humans (10).

For a wide variety of vaccines and vaccine candidates, analysis of blood transcriptional profiles after vaccination has revealed adjuvant-specific patterns of innate immune activation and inflammation that, in some instances, correlate with vaccine immunogenicity and/or efficacy (11–16). Previous analyses of transcriptional responses after RTS,S vaccination revealed strong AS01-driven peripheral innate immune activation and candidate protection-associated signatures for individual studies (17–21). While biomarker development and understanding of pathogenesis for other infectious diseases have been accelerated by multi-cohort transcriptional analyses (22–25), a multi-study transcriptional analysis of RTS,S-mediated protection in CHMI has not been undertaken. Identification of simple, yet robustly predictive, signatures of RTS.S clinical activity would facilitate evaluation of additional alternative malaria vaccine regimens, providing preclinically testable hypotheses about the RTS,S mode of action and refining candidate correlative variables for testing in field studies.

To identify robust correlates of RTS,S-mediated protection, we compiled blood transcriptome data from all RTS,S CHMI trials that, to our knowledge, had this data available or had blood samples available that could be used to generate transcriptomic data. In total, we compiled microarray data from four RTS,S CHMI studies, generated new RNA-sequencing data for one of these, and generated RNA-Sequencing data for another study. Each of these five independent studies evaluated the most commonly employed RTS,S regimen of 3 monthly 50 μg doses of RTS,S/AS01 (termed “RRR” herein) as well as alternative regimens involving RTS,S. As each study involved relatively small numbers of volunteers, it was not possible to identify robust signatures by conventional training/test splits for signature discovery and validation. Instead, we employed multivariate modeling of pre-challenge data from RRR recipients to first identify a set of candidate signatures that were then evaluated using data from alternative regimen recipients. This analysis identified a two-transcript ratiometric signature that consistently discriminates RTS,S vaccine recipients that will be protected from *P. falciparum* malaria challenge.

## Methods

Written informed consent was obtained from each subject before study procedures were initiated. All laboratories received deidentified samples and performed tests according to protocol, and therefore their work was IRB-exempt.

### Generation and Compilation of Host Blood Gene Expression Profiles From Five Independent RTS,S CHMI Studies

**Figure 1:**
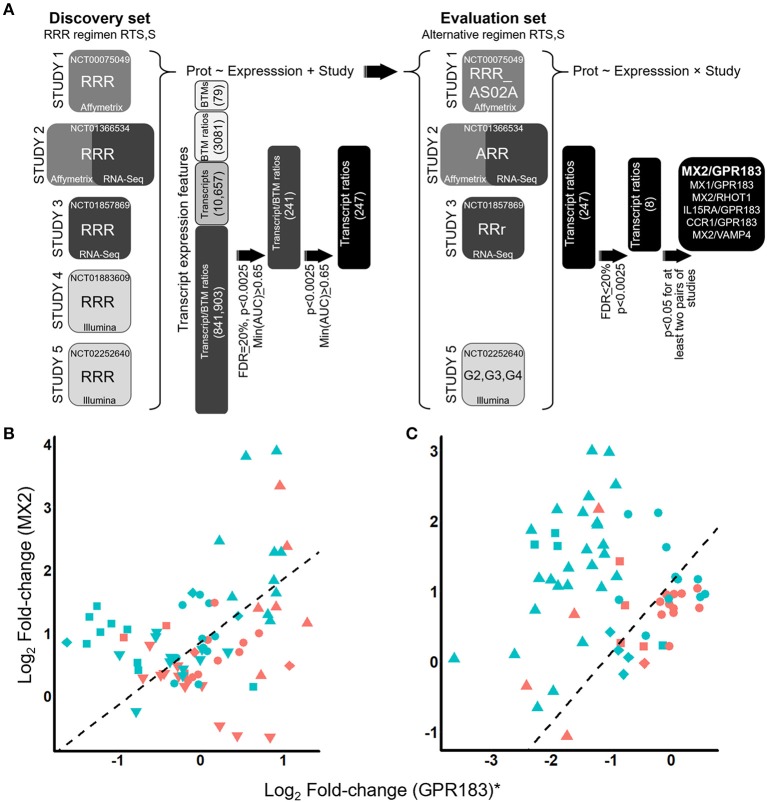
Identification of the ratiometric transcript signature MX2/GPR183 as a consistent discriminator for RTS,S vaccine recipients that will be protected from CHMI. **(A)** RTS,S CHMI studies evaluated and analysis schematic. Pre- and post-challenge blood transcriptomes from five independent RTS,S vaccination CHMI studies were integrated and interrogated for transcript signatures that consistently discriminate vaccine recipients that would be protected from those who would not be protected. The first stage of the analysis (left) involved discovery of signatures through analysis of RRR regimen RTS,S recipients. These signatures were then evaluated using data from recipients of alternative vaccine regimens involving RTS,S (right). Prot, binary protection variable (protected or not). BTM, blood transcriptional module. Definition of vaccine regimens: RRR, 3 monthly 50 μg doses of RTS,S/AS01; RRR_AS02A, 3 monthly 50 μg doses of RTS,S/AS02A; ARR, One dose with Ad35.CS.01 followed 1 month later by 2 monthly doses of 50 μg RTS,S/AS01; RRr, 2 monthly doses of 50 μg RTS,S/AS01 followed 5 months later by a third dose of 10 μg RTS,S/AS01; G2, 2 monthly 50 μg doses of RTS,S/AS01 followed 1 month later by a 10 μg dose of RTS,S/AS01; G3, a 50 μg dose of RTS,S/AS01 co-administered with ChAd63 ME-TRAP followed 1 month later by 2 monthly doses of 50 μg RTS,S/AS01 co-administered with MVA ME-TRAP; G4, a 50 μg dose of RTS,S/AS01 co-administered with ChAd63 ME-TRAP followed 1 month later by a 50 μg dose of RTS,S co-administered with MVA ME-TRAP followed 1 month later by a 10 μg dose of RTS,S co-administered with MVA ME-TRAP. **(B,C)** Scatterplots for log2 fold-changes in MX2 plotted against log2 fold-changes in GPR183 for recipients of RRR regimen RTS,S **(B)** or alternative regimen RTS,S **(C)**. Fold-changes were computed comparing expression levels on Day 1 post-3rd vaccination compared to pre-vaccination values. For visualization purposes, the log2 fold-changes for GPR183 were transformed to study-adjusted values (“GPR183*”) using parameter estimates from the logistic regression models (Supplementary Methods). Colors indicate whether the participants were protected (blue) or not protected (red) after CHMI. Shapes indicate study and vaccine arm. For **(B)** upside-down triangles, Study 1 microarray; circles, Study 2 microarray; triangles, Study 3 RNA-Seq; squares, Study 4 microarray; and diamonds, Study 5 microarray data. For **(C)** circles, Study 2 ARR microarray; triangles, Study 3 RRr RNA-Seq; diamonds, microarray data from Study 5 G2; and squares, Study 5 G3. Dashed line indicates the decision boundary that maximizes the sum of sensitivity and specificity.

The following studies with blood transcriptome data were analyzed (summarized in Figure 1A and Supplementary Tables 1, 2): Study 1 (2), Study 2 (3), Study 3 (4), Study 4 (5), and Study 5 (6). Each study includes an “RRR” RTS,S/AS01 arm (3 monthly 50 μg doses of RTS,S/AS01B), and at least one alternative arm in which vaccine doses, adjuvant, schedules, and/or modes of antigen presentation were modified. PBMC microarray data for Study 1 and Study 2 were obtained from Array Express E-MTAB-4629 (19) and GEO GSE89292 (18), respectively. PBMC RNA-Seq data for Study 2 and 3 were generated and processed using standard methods (26–29) (Supplementary Methods #1) and deposited into GEO (GSE103401 and GSE102288, respectively). Whole blood microarray data for Study 4 and Study 5 were obtained from GSE103862 and GSE103874, respectively. Data were normalized using standard methods (26–29) (Supplementary Methods #1) and integrated in terms of gene symbols after normalization (Supplementary Methods #1). For all studies with pre-existing data, all available data that passed quality control was used in the analyses; for studies with newly-generated RNA-Seq data, all samples with sufficient RNA were submitted for sequencing, and all samples with RNA-Seq data that passed quality control were analyzed. No subselection of samples or participants was performed; the number of data points for each condition are enumerated in Supplementary Table 2. The final 5-study integrated dataset was deposited into GEO (GSE107672). Expression patterns for transcriptional modules were derived using published module definitions (13, 30–32) (Supplementary Methods #2; Supplementary Table 4).

### Quantitative Real-Time PCR

To assess the veracity of the new RNA-Seq data generated for Study 2 (3) and Study 3 (4), we performed multiplex quantitative real-time PCR (qRT-PCR) on a panel of 31 genes with roles in the inflammatory, immune response, and other diverse functions. Reference (“housekeeping”) genes for internal normalization of the PCR data were identified from the RNA-Seq data by minimizing the coefficient of variation. Genes and corresponding commercial TaqMan primer/probe sets (Thermo Fisher Scientific) are listed in Supplementary Table 3. qRT-PCR data was generated using the BioMark HD multiplex microfluidic instrument (Fluidigm, Inc.) in 48 sample X 48 assay multiplex format, as described (33). Data was normalized using the delta-delta cycle threshold (Ct) method (34), using the average Ct of the reference genes for standardization. This analysis was performed using backup or excess RNA samples that remained after completing transcriptomics and comprised 38 samples from Study 2 (21 participants at pre-vaccination and/or the day of the 3rd vaccination) and 110 samples from Study 3 (33 participants at one or more of the following five time points: pre-vaccination, the day of the third vaccination, 3 or 14 days after the third vaccination, and/or the day of challenge). One-sided Spearman rank correlation analysis was used to compare qRT-PCR data to the other platforms for individual genes or z-transformed datasets overall.

### Statistical Tests

The modeling strategy is shown in Figure 1A. Although a variety of time points post-vaccination were profiled in each study, the only time points consistently profiled in all five studies were pre-vaccination and 24 h after the 3rd vaccination (Supplementary Table 2). For this reason, analyses were performed using log2 fold-changes computed between these two time points. Logistic regression (LR) was used to model challenge outcome (protected or not protected) as a function of transcriptional readouts and categorical study terms and significance of discrimination was assessed using Chi-squared tests. Discrimination accuracy was assessed using overall and study-specific ROC AUCs. These analyses were first performed for recipients of the RRR regimen using modules, individual transcripts, ratios between modules, and ratios between transcripts and modules as transcriptional readouts (Supplementary Methods #3). As PBMC transcriptomes for Study 2 participants were analyzed by both microarray and RNA-Seq, parallel analyses were performed in which Study 2 data from either platform were modeled with the other studies and worst-case *p*-values and ROC AUCs for the two analyses were taken as summary statistics. Significant transcript/module ratios were expanded to transcript/transcript ratios by evaluating all members of the modules and retaining those that passed the original filtering criteria. Surviving transcript/transcript ratios were evaluated on data for alternative RTS,S regimens (Supplementary Methods #4). Complementarity between the top transcript ratio (MX2/GPR183) and anti-CSP titers for predicting challenge outcome was assessed by Chi-squared test comparing LR models containing terms for study and anti-CSP titers to models containing the transcript ratio, study, and anti-CSP terms (Supplementary Methods #5). For all analyses, *p*-values were adjusted using the Benjamini-Hochberg false discovery rate (FDR) algorithm.

### Assessment of RNA/Protein Correlations Using Pre-existing Proteogenomic Datasets

We mined three public proteogenomic datasets to determine whether transcript and protein levels of MX2 and GPR183 were generally correlated across diverse human tissue samples. The first was a breast cancer cohort (35) in which transcript and protein (MX2) or phosphopeptide (GPR183:S343) levels were available for 77 patients via a web-based portal (http://prot-shiny-vm.broadinstitute.org:3838/CPTAC-BRCA2016/). The second was a large cohort of hepatitis B virus (HBV)-related hepatocellular carcinoma (HCC) patients (36) for whom transcript and protein (MX2) or phosphopeptide (GPR183: S328, S333, S337, and S343) levels were available. Data for this cohort was obtained from the manuscript online supporting material and associated database and consisted of 298 paired samples after excluding samples with ambiguous matches or mismatches between the metadata gender or inferred gender from the transcriptomics. The third study assessed a panel of 29 healthy tissue samples by paired transcriptomics and proteomics (37). Data for proteomics and transcriptomics for both MX2 and GPR183 was obtained from the manuscript online supporting material. One-sided Spearman rank correlation analysis was performed for all RNA vs. protein or RNA vs. phosphopeptide comparisons.

### Assessment of Gene Expression in Immune Cell Populations Using Pre-existing Single Cell RNA-Seq Datasets

Previously-published single-cell RNA-Seq data from human PBMCs (38) and human blood dendritic cell (DC) and monocyte populations (39) was mined to determine potential cellular origins of the MX2 and GPR183 transcripts in blood. For both studies, processed data and t-distributed stochastic neighbor embedding (t-SNE) (40) visualizations of annotated cell clusters were obtained through the Broad Single Cell Portal (https://singlecell.broadinstitute.org/single_cell).

## Results

We compiled microarray datasets and generated new RNA-Seq datasets for blood samples from all RTS,S-based CHMI studies that, to our knowledge, either had this data available or had materials available to generate this data: Study 1 (2), Study 2 (3), Study 3 (4), Study 4 (5), and Study 5 (6). In total 86 and 124 volunteers were profiled that had received RRR or alternative regimen RTS,S, respectively (Figure 1A; Supplementary Tables 1, 2) and underwent controlled malaria challenge. To confirm the veracity of the new RNA-Seq data that was generated for Study 2 and Study 3, we performed multiplex qRT-PCR analysis of a panel of 31 genes with roles in the inflammatory, immune response, and other diverse functions (Supplementary Table 3) for a subset of time points and participants. Gene-level correlations between qRT-PCR and RNA-Seq were statistically significant and generally high magnitude for both Study 2 (median Spearman Rho = 0.75, IQR = 0.63–0.84) and Study 3 (median Spearman Rho = 0.83, IQR = 0.73–0.91) (Supplementary Table 3). Furthermore, overall correlations between matching z-transformed qRT-PCR and RNA-Seq datasets were significant and high magnitude (Study 2: Spearman Rho = 0.74; Study 3: Spearman Rho = 0.81) (Supplementary Figure 1). To facilitate interpretation of the high dimensional transcriptomics data, we determined which pre-defined blood transcriptional modules (BTMs, Supplementary Methods #2) were coherently expressed across all studies (Supplementary Table 4; Supplementary Figure 2), revealing many modules previously found to be differentially expressed after RTS,S vaccination such as interferon response (17–20).

We next determined whether post-vaccination/pre-challenge expression profiles could discriminate protected from non-protected RRR vaccine recipients. These analyses were performed using fold changes comparing expression levels 1 day after the 3rd immunization (“D1-post-3rd”) to pre-vaccination levels, as these time points were assessed in all five studies (Supplementary Table 2). Analysis of this particular fold-change also has the advantage of potentially capturing both vaccine adjuvant-driven innate immune responses and vaccine antigen-driven adaptive immune recall responses that were primed by the first two doses. While low accuracy discrimination was achieved for transcriptional signatures comprised of individual modules, individual transcripts, or ratios between pairs of modules (Supplementary Table 5), moderate but consistent discrimination accuracy (min(ROC AUC) > 0.65) across all studies and platforms was achieved by considering ratios between modules and transcripts, with 241 transcript/module signatures surviving multiple testing correction at FDR = 20.1 % (Figure 1A, Supplementary Table 6). The most discriminatory signatures were ratios between the microfibrillar transcript MFAP3 and lymphoid lineage (overall ROC AUC = 0.74, FDR = 20.1%), and ratios between the oxysterol receptor GPR183 and innate immunity/interferon- modules (overall ROC AUC = 0.74, FDR = 20%). The top five most frequently selected transcripts (GPR183, AGPAT4, NLRP3, RIPK2, and TNF) were paired with interferon response (Supplementary Figure 3). Given that the transcriptional modules are each comprised of multiple transcripts (Supplementary Methods #2; Supplementary Table 4) we expanded the transcript/module ratios into transcript/transcript ratios (Supplementary Methods #3) to enable future adaptation to alternative platforms (such as transcript-based quantitative PCR). At the original significance thresholds (*p* < 0.0025, FDR = 20.1%) 247 transcript/transcript signatures were obtained (Supplementary Table 7) and are depicted as a Cytoscape interaction network (41) in Supplementary Figure 4.

We tested the 247 transcript ratio signatures for ability to discriminate protected from non-protected recipients of alternative RTS,S regimens (Supplementary Tables 1, 2) constructing the validation testing strategy to the detect transcript ratio signatures that were discriminatory for most (but not necessarily all) of the modified regimens (Supplementary Methods #5). The top signature resulting from the analysis was the ratio between MX2 (an interferon response module transcript) and the oxysterol receptor GPR183. MX2/GPR183 achieved discrimination for alternative RTS,S regimen recipients in Study 2 (“ARR”), Study 3 (“RRr”), and Study 5 (“G2” & “G3”) in a manner consistent with the RRR regimen recipients (Figures 1B,C; Supplementary Table 8; Supplementary Figures 5, 6). For both the RRR regimen and these alternative regimens, patients that exhibited higher D1 post-3rd fold-changes in the MX2/GPR183 ratio were more likely to be protected after challenge (Figures 1B,C; Supplementary Figure 5).

MX2 is a canonical interferon-response gene and belongs to several innate immune response and interferon-associated transcriptional modules [Supplementary Table 4 (13, 31, 32)] that can be induced by a variety of vaccine adjuvants and inflammatory stimuli. Even though RTS,S/AS01 vaccination led to robust induction of these modules after each vaccination (Supplementary Figure 2), the fold changes for these modules at 24 h post-3rd vaccination were not consistently associated with protection against CHMI for RRR recipients, with the two best performing MX2-containing modules achieving minimum ROC AUC across RRR cohorts of 0.59 and 0.52 for “*M165_ENRICHED IN ACTIVATED DENDRITIC CELLS (II)*” (13) and “*HALLMARK_INTERFERON_GAMMA_RESPONSE*” (32), respectively (Supplementary Table 5). Furthermore, comparing the fit of logistic regression models to the entire dataset (RRR + alternative RTS,S regimen recipients) showed that the MX2/GPR183 ratio appreciably outperformed the individual genes MX2 and GPR183 as well as three top-performing interferon-response-associated modules (“*HALLMARK_INTERFERON_GAMMA_RESPONSE*,” “*HALLMARK_INTERFERON_ALPHA_RESPONSE*,” and “*M165_ENRICHED IN ACTIVATED DENDRITIC CELLS (II)*”) (Supplementary Figure 7).

**Figure 2:**
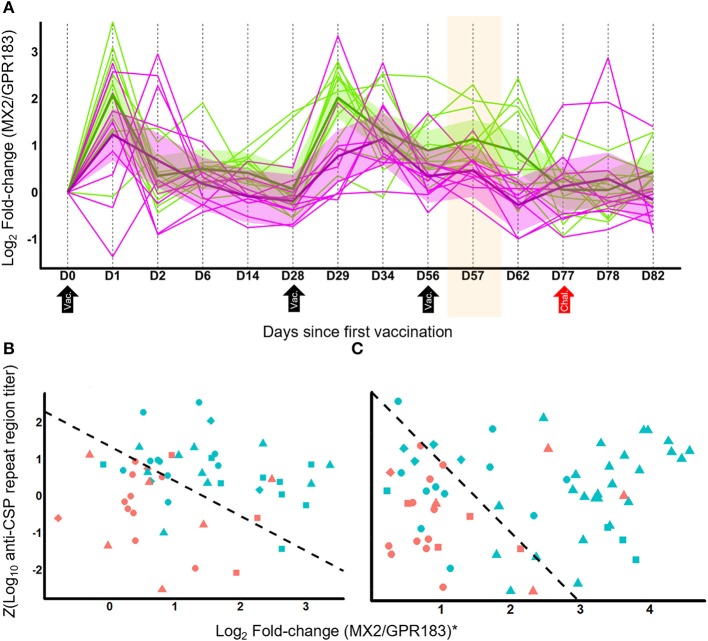
MX2/GPR183 is a transcriptionally dynamic signature that complements anti-CSP titers for identifying which RTS,S vaccine recipients will be protected from CHMI. **(A)** RNA-Seq temporal profile for log2 fold-changes in the MX2/GPR183 expression ratio for RRR regimen RTS,S in Study 2. Magenta lines indicate participants that were not protected, green lines indicate participants that were protected after challenge. Shaded areas indicate 90% confidence intervals for linear mixed models for protected and non-protected vaccine recipients. RTS,S vaccinations were performed on D0, D28, and D56; CHMI was performed on D77. The shaded area highlights D57, which for RRR corresponds to Day 1 after the third vaccination which is the time point used to identify the Log2(MX2/GPR183) as being consistently associated with RTS,S-mediated protection. **(B,C)** Scatterplots of Z-transformed day-of-challenge anti-CSP (repeat region) titers plotted against log2 fold-changes for the MX2/GPR183 ratio for recipients of RRR regimen RTS,S **(B)** or alternative regimen RTS,S **(C)**. Fold-changes for MX2/GPR183 were computed comparing expression levels on Day 1 post-3rd vaccination compared to pre-vaccination values. For visualization purposes, the log2 fold-changes for MX2/GPR183 were transformed to study-adjusted values (“MX2/GPR183*”) using parameter estimates from the logistic regression models (Supplementary Methods). Colors indicate whether the participants were protected (blue) or not protected (red) after CHMI. Shapes indicate study and vaccine arm. For **(B)** circles, Study 2 microarray; triangles, Study 3 RNA-Seq; squares, Study 4 microarray; and diamonds, Study 5 microarray data. For **(C)** circles, Study 2 ARR microarray; triangles, Study 3 RRr RNA-Seq; diamonds, microarray data from Study 5 G2; squares, microarray data from Study 5 G3. Dashed line indicates the decision boundary that maximizes the sum of sensitivity and specificity.

Analysis of the fold-change profile of MX2/GPR183 at additional time points suggested broad discrimination post-vaccination and post-challenge, including D1 post 2nd vaccination, the day of the 3rd vaccination, 3 days after 3rd vaccination, and 5 days after challenge (Supplementary Table 9). Within densely-profiled Study 2, MX2/GPR183 exhibited complex kinetics (Figure 2A) that derive from variable patterns of down-regulation of GPR183 and consistent up-regulation of MX2 (Supplementary Figure 8). Interestingly, analysis of pre-vaccination expression profiles suggested a baseline association between the MX2/GPR183 ratio and protection, but this trend was not observed consistently (e.g., the Study 4 ROC AUC obtained using baseline MX2/GPR183 expression was 0.41, which is markedly reduced compared to the Study 4 ROC AUC of 0.74 obtained using the fold change, Supplementary Table 9).

Given that anti-CSP titers induced after RTS,S vaccination are associated with protective responses (8), we tested whether combining this measurement with the MX2/GPR183 ratio significantly improved discrimination. For both RRR and alternative regimens, including MX2/GPR183 fold changes at Day 1 post-3rd vaccination with day of challenge anti-CSP titers led to statistically significant improvements in discrimination between protected and non-protected vaccine recipients compared to anti-CSP titers alone (*p* = 0.005 and 0.003 for RRR and alternative regimens, respectively; Figure 2; Supplementary Figure 9). Importantly, the MX2/GPR183 expression ratio and anti-CSP titers are not correlated for either RRR or alternative regimens, suggesting that these two readouts capture distinct aspects of the RTS,S-driven immune response.

The practical utility of the MX2/GPR183 signature for assessing immune responses in future malaria vaccine and CHMI studies is reinforced by the ability to measure it using various platforms, given that it was discovered through integrated analysis of cohorts employing three different transcript profiling platforms and two sample types (PBMCs and whole blood), and that the overall veracity of the RNA-Seq measurements used for discovery of the signature was confirmed by qRT-PCR (Supplementary Figure 1). As a further examination of the cross-platform concordance for MX2/GPR183 quantification, we directly compared 613 samples from Study 2 that were assessed by both RNA-Seq and Affymetrix microarrays. Cross-platform correlations for MX2, GPR183, and MX2/GPR183 were high (Spearman rho = 0.96, 0.86, and 0.92, respectively, Supplementary Figure 10), which is remarkable given that the two platforms do not target the same region of either transcript (whole-transcript quantification by RNA-Seq, 3′ transcript targeting by Affymetrix microarray).

**Figure 3:**
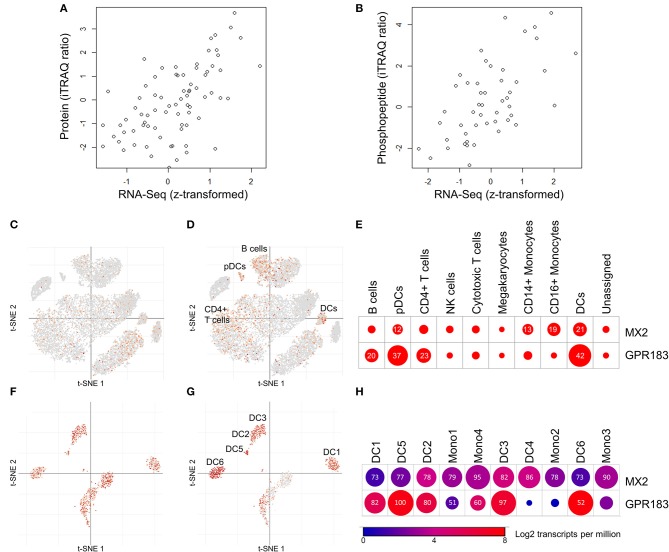
Mining public proteomic and public single-cell RNA-Seq datasets provides support for protein/RNA correlations and cell type-specific expression of MX2 and GPR183. **(A,B)** Correlations between protein or phosphopeptide abundance and transcript abundance across diverse breast cancer tissues (35). **(A)** Correlation between MX2 protein abundance and MX2 transcript (Spearman Rho = 0.58, *p* = 2.4 × 10^−8^, *N* = 77). **(B)** Correlation between GPR183:S343 phosphopeptide abundance and GPR183 transcript (Spearman Rho = 0.60, *p* = 3.8 × 10^−6^, *N* = 49). Data from Mertins et al. (35) was obtained through the data portal provided (http://prot-shiny-vm.broadinstitute.org:3838/CPTAC-BRCA2016/). **(C,E)** Expression of MX2 and GPR183 in individual PBMC cells measured by single-cell RNA-Seq reported in Liu et al. (38). **(C,D)** t-distributed stochastic neighbor embedding (t-SNE) scatterplots demonstrating that MX2 **(C)** is detected sporadically in many lineages, while GPR183 **(D)** is enriched in clusters annotated as DCs, pDCs, B cells, and CD4^+^ T cells. **(E)** Dotplot depicting frequency of MX2 and GPR183 expression specific annotated cell lineages. Numbers and circle sizes depict the percentages of cells from a given linege are positive for a given marker. **(F–H)** Expression of MX2 and GPR183 in individual DCs and monocytes measured by single-cell RNA-Seq reported in Villani et al. (39). **(F–H)** t-SNE scatterplots demonstrating that MX2 **(F)** is frequently detected in all lineages, while GPR183 **(G)** is enriched in all DC clusters (except DC4) but not monocytes. **(H)** Summary dotplot depicting average transcript levels (color) and frequency of detection (numbers) for MX2 and GPR183 in DC and monocyte lineages. For **(C–H)**, data and visualizations were obtained from the Broad Single Cell portal (https://singlecell.broadinstitute.org/single_cell).

Further functional interpretation of the MX2/GPR183 signature for RTS,S induced immune responses requires confirmation that the transcript levels of MX2 and GPR183 are representative of cognate functional protein levels and identification of the specific immune cell populations from which the signature derives. While material constraints prohibit proteomic and single-cell assessments of samples from the five CHMI clinical trials analyzed herein, we mined public proteogenomic and single cell RNA-Seq datasets to inform these considerations for MX2 and GPR183. For protein/RNA correlations, we analyzed data from studies that performed paired transcriptomic and proteomic/phosphoproteomic analysis of human tissues from large scale breast cancer (BrCa) (35), hepatocellular cancer (HCC) (36), or healthy tissue (HT) (37) cohorts (Figures 3A,B). MX2 protein was detected in all three datasets and strong protein/RNA correlations were observed: Spearman Rho for BrCa = 0.58 (*p* = 2.4 × 10^−8^, *N* = 77, Figure 3A); for HCC = 0.62 (*p* = 0, *N* = 298); and for HT = 0.71 (*p* = 7.5 × 10^−6^, *N* = 29). MX2 (S676) phosphopeptide was also detected in the BrCa and HCC cohorts and was correlated with MX2 transcript in both cases: Spearman Rho for BrCa = 0.46 (*p* = 2.9 × 10^−5^, *N* = 73); and for HCC = 0.58 (*p* = 2.3 × 10^−21^, *N* = 222). GPR183 bulk protein was detected only in the HT cohort and was correlated with GPR183 transcript: Spearman Rho = 0.43 (*p* = 0.01, *N* = 29). GPR183 phosphopeptides were detected in both BrCa and HCC cohorts and were correlated with GPR183 transcript in all cases: Spearman Rho for GPR183:S343 in BrCa = 0.60 (*p* = 3.8 × 10^−6^, *N* = 49, Figure 3B); and Spearman Rho in HCC for GPR183:S343 = 0.39 (*p* = 1.6 × 10^−9^, *N* = 212), for GPR183:S328=0.49 (*p* = 1.6 × 10^−15^, *N* = 232), for GPR183:S333 = 0.46 (*p* = 4.8 × 10^−14^, *N* = 232), and for GPR183:S337 = 0.49 (*p* = 8.0 × 10^−20^, *N* = 298).

To gain insight into the immune cell populations expressing MX2 and GPR183, we mined data from single cell RNA-Seq analyses of human PBMCs (38) and human blood dendritic cell (DC) and monocyte sub-populations (39). MX2 exhibited moderately enhanced expression in innate immune cells compared to other PBMCs (Figures 3C,E) but did not exhibit preferential expression between DC or monocyte subpopulations (Figures 3F,H). In contrast, GPR183 displayed preferential expression in DCs, plasmacytoid DCs (pDCs) and, to a lesser extent, B cell and CD4^+^ T cells compared to other PBMCs (Figures 3D,E). Within DC and monocyte populations, GPR183 was abundantly expressed in all DC subpopulations except monocyte-associated DC4 (39) and exhibited limited expression in monocytes (Figures 3G,H).

## Discussion

RTS,S/AS01 is currently the most advanced subunit vaccine to demonstrate protective efficacy against *Plasmodium falciparum* (*Pf* ) infection, but the basis for protection is unclear (7). Identification of predictive correlates for RTS,S efficacy could enable accelerated malaria vaccine development by clarifying additional protective immune responses and thereby facilitating screening and differentiation of novel vaccine candidates. From the integrated transcriptomic analysis of five independent CHMI studies that evaluated the efficacy of RTS,S (2–6) we identified the transcript ratio MX2/GPR183 as a signature that consistently discriminates protected from non-protected recipients of RRR regimen and several alternative RTS,S regimens. Discovery and assessment of the signature was made using fold-changes computed between pre-vaccination and 24 h after the third vaccination, the two time points that were consistently assessed in all five studies (Supplementary Table 2). This time point may nevertheless be advantageous because it has the potential to capture both early inflammatory changes driven by the AS01 adjuvant and recall of adaptive immune responses primed by the first two vaccinations. This conjecture is supported by the observation that enhanced up-regulation of IFN-associated signaling pathways is generally observed in adults after the second dose of AS01-containing vaccines compared to the first dose (42). While the association of MX2/GPR183 with RTS,S-mediated protection against CHMI could not be assessed for other time points for all of the studies, exploratory analysis of the available data suggests that the association is not restricted to 24 h after the third dose (Supplementary Table 9). Notably, the MX2/GPR183 ratio provides information that is complementary to and not redundant with, anti-CSP levels for predicting which vaccine recipients will be protected—with protected volunteers having relatively higher anti-CSP levels and/or higher MX2/GPR183 fold-changes compared to non-protected volunteers (Figures 2B,C). This result suggests that MX2/GPR183 may capture other aspects of RTS,S-driven immunity besides binding antibody titer, such as aspects of RTS,S driven cellular immunity (43), and/or antibody post-translational modifications and Fc effector function (44). Up-regulation of MX2/GPR183 after vaccination with subunit vaccines for other indications also may be associated with protective responses if those same immune mechanisms are protective against the relevant pathogen (for example, *Mycobacterium tuberculosis*).

These data support including quantitative PCR-based assays targeting MX2 and GPR183 alongside assessments of RTS,S-induced humoral and cellular immune responses within biomarkers strategies for CHMI studies of novel vaccines and RTS,S field trials to inform how other variables (such as age and health status of the subject) influence RTS,S -induced immunity. While material limitations precluded qRT-PCR assessment of MX2/GPR183 in the CHMI studies described herein, the discovery of MX2/GPR183 from integrated analysis of three different transcriptomics platforms (Supplementary Table 1), the overall concordance between Study 2 and Study 3 RNA-Seq and qRT-PCR measurements (Supplementary Table 3; Supplementary Figure 1), and the very robust correlation between MX2, GPR183, and MX2/GPR183 levels measured by RNA-Seq and Affymetrix microarray in Study 2 (Supplementary Figure 10) support the robustness of the signature. Furthermore, mining published proteogenomic datasets for healthy tissues (37) and tumor tissues (35, 36) from indications with appreciable immune cell involvement (45, 46) revealed significant and consistent correlations between protein (or phosphopeptide) levels and RNA levels for both MX2 and GPR183 (Figures 3A,B), suggesting that protein-based assessment of the MX2/GPR183 may be feasible.

Notably, signatures that consistently discriminated protected from non-protected RTS,S recipients were only identified when ratios between genes and transcriptional modules were considered, not individual modules or genes (Supplementary Tables 5, 6). This result suggests that a balance between the implicated pathways, rather than the absolute pathway activation levels, may be a determinant and/or readout of RTS,S-induced protective immunity. The utility of signatures based on transcript ratios or ensembles of transcript ratios has been demonstrated for other infectious diseases (22–24, 26), suggesting that assessing balance between biological process captured by transcriptomics may be a broadly practical and informative diagnostics strategy.

Given that MX2 is an exemplar interferon-response gene (13, 31, 32) that is induced by numerous immunogenic stimuli, with consistent induction after each dose of RTS,S (Supplementary Figure 8B), a possible hypothesis is that the MX2/GPR183 ratio simply represents generic adjuvant-driven inflammation. This hypothesis is not supported by our data, however, as the expression of interferon or innate immune response modules by themselves were not consistently associated with RTS,S-mediated protection against CHMI (Supplementary Table 5; Supplementary Figure 7). While activation of interferon response may be a *necessary* component of an RTS,S protection signature, our data indicates that this alone is not *sufficient* for consistent discrimination between protected and non-protected volunteers across multiple studies. This observation is consistent with the results of a detailed comparative analysis of AS01 adjuvants that revealed that the magnitude of the innate immune response did not correlate with the subsequent adaptive immune response magnitude (42). This is in contrast to prior studies of influenza vaccines, where induction of interferon response genes generally correlated with the humoral response (12, 14, 47). Indeed, the relationship between the innate and adaptive immune responses triggered by vaccines is likely to be vaccine-specific, given that these and other varied and apparently contradictory associations have been reported (11, 48, 49), and resolving the underlying circuitry is a critical area of inquiry for systems analysis (50).

GPR183 (also known as EBI2), encodes a pleiotropic GPCR that is both a negative regulator of interferon responses (51, 52) and a chemotactic oxysterol receptor expressed on B cells and T cells (53) and DCs (52, 54). A critical role for GPR183 in B cell activation and germinal center development is suggested by the requirement for GPR183 down-regulation for B cell migration into central follicular areas, and defective antibody responses in GPR183 deficient B cells (53). Furthermore, GPR183 expression on T cells and DCs promotes appropriate localization of these cells in lymphoid organs to promote CD4^+^ T cell responses (55, 56). By mining public data from two proteogenomic tumor tissue analyses of tumor tissues (35, 36) we found that GPR183 phosphopeptides are frequently detected and were significantly correlated with GPR183 RNA in all instances (Figure 3B), adding a potentially novel post-translational regulatory mode for GPR183 function that is reflected in transcript profiles. Unlike MX2, which was induced 24 h after each RTS,S vaccination (Supplementary Figure 8B), GPR183 exhibited a complex expression response pattern that differed for each dose, with GPR183 levels generally being lower in volunteers that would ultimately be protected (Figures 1B,C; Supplementary Figure 8A). To gain insight into the immune cell populations in blood that may predominantly express GPR183, we mined data from two published single cell RNA-Seq datasets (38, 39). Consistent with the reported expression patterns (52–54), GPR183 was preferentially expressed in DCs, B cells and CD4 T cells (Figure 3D), with DCs having the highest positivity (42 and 37% for DCs and pDCs, respectively, Figure 3E). Amongst blood DC and monocyte populations, GPR183 was abundantly expressed in all but one of the six DCs populations and more sporadically expressed in monocytes (Figures 3G,H). Future single cell RNA-Seq or flow cytometry-based analyses of RTS,S trials are needed to resolve whether changes in blood DC populations (which are rare but express high levels of GPR183) and/or changes in blood B cells and CD4^+^ T cells (which are more common but express GPR183 less frequently) underlie the expression changes observed in PBMCs and whole blood. In either case, given the prominent role for GPR183 in immune cell chemotaxis to lymphoid tissues, a plausible interpretation of the reduced blood GPR183 RNA levels in protected individuals would be enhanced migration that can lead to a more robust adaptive immune response.

## Conclusions

Through integrated transcriptomic analysis of five independent CHMI studies we have identified a post-vaccination/pre-challenge transcript ratio signature that consistently discriminates protected from non-protected recipients of RTS,S vaccination. This signature generates hypotheses about the RTS,S clinical mode of action and complements anti-CSP antibody levels for predicting which vaccine recipients will be protected—thereby providing a convenient readout for currently uncharacterized immune mechanisms that, together with binding antibodies, protect against malaria challenge. The relevance of the MX2/GPR183 ratio to RTS,S-mediated protection against *Plasmodium falciparum*. nevertheless needs to be assessed in real world settings in pediatric populations that are subject to complex environmental factors and other variables that influence the host:pathogen interface, including innate immune responses triggered by exposure to the malaria parasite itself (57).

## Data Availability Statement

The datasets generated for this study can be found in the Gene Expression Omnibus with accession numbers GSE103401, GSE102288, GSE107672.

## Ethics Statement

Ethical review and approval was not required for the study on human participants in accordance with the local legislation and institutional requirements. The patients/participants provided their written informed consent to participate in this study.

## Author's Note

Material has been reviewed by the Walter Reed Army Institute of Research. There is no objection to its presentation and/or publication. All reviews complete–scientific, human use, public affairs, and operational security. Approved for release.

## Author Contributions

RB, EJ, DD, JS, JH, MG, WB, JR, RM, AA, CO, AH, UW-R, and DZ: designed the research. YD, ET, JM, JV, JB, SS, and DZ: performed the research. YD and DZ: wrote the manuscript.

## Conflict of Interest

EJ, RB, RM, and WB were and are currently employed by the GSK group of companies. JS and JH were and are currently employed by Janssen Vaccines and prevention (BV, Leiden). The remaining authors declare that the research was conducted in the absence of any commercial or financial relationships that could be construed as a potential conflict of interest.

## Supplementary Material

Supplementary Figure 1 Overall correlation between quantitative real-time PCR (qRT-PCR) and microarray or RNA-Seq data for Study 2 and Study 3. For a panel of 31 genes with roles in the inflammatory, immune response, and other diverse functions (Supplementary Table 3), qRT-PCR data was generated for 21 participants from Study 2 at up to two time points (pre-vaccination and day of 3rd vaccination) and for 33 participants from Study 3 at one or more of 5 time points (pre-vaccination, the day of the third vaccination, 3 or 14 days after the third vaccination, and the day of challenge), giving a total of 1,178 data points for Study 2 and 3,410 data points for Study 3. For overall cross-platform comparisons, normalized data on the log2 scale were z-transformed for each gene and then combined. **(A)** Study 2 microarray vs. qRT-PCR (Spearman Rho = 0.73, *p* = 0, *N* = 1178), **(B)** Study 2 RNA-Seq vs. qRT-PCR (Spearman Rho = 0.74, *p* = 0, *N* = 1178), **(C)** Study 3 RNA-Seq vs. qRT-PCR (Spearman Rho = 0.81, *p* = 0, *N* = 3410). Individual gene-level correlations are provided in Supplementary Table 3.

Supplementary Figure 2 Temporal expression profiles for coherent transcriptional modules in all studies. For each coherent module defined in Supplementary Table 4, Log2 expression fold changes across all genes within the module were computed for each volunteer at each time point. Time course plots depict trajectories of module-average expression for each volunteer (thin lines) and the overall averages across all volunteers. Shown is a representative plot for an individual module (“HALLMARK_INTERFERON_GAMMA_RESPONSE”). For the complete set of module expression profiles, please see: Supplementary Tables 1–9.

Supplementary Figure 3 Frequency of individual modules and transcripts in the transcript/module ratios associated with protection after RTS,S vaccination. **(A)** Barplot depicting the number of significant transcript/module ratios in which specific modules appeared. While a lymphoid lineage module was individually the most frequent module, numerous antiviral/interferon response modules appeared frequently (shown in green). **(B)** Barplot depicting the number of significant transcript/module ratios in which specific transcripts appeared. The oxysterol receptor GPR183 was the most frequently selected gene. **(C)** Heatmap depicting the transcript/module ratios for transcripts and modules that were selected frequently. The top 5 transcripts (GPR183, AGPAT4, NLRP3, RIPK2, and TNF) appeared in significant ratios with interferon and viral response-associated modules.

Supplementary Figure 4 Network representation of 247 transcript/transcript ratios that were selected based on consistent discrimination of protected from non-protected recipients of alternative regimen RTS,S vaccination. Each node (circle) represents an individual gene. The presence of an edge (line) between nodes indicates that transcriptional fold-change ratios (Day 1 after 3rd vaccination compared to pre-vaccination) between those genes consistently discriminate protected from non-protected recipients of RRR regimen RTS,S (Supplementary Table 7). Node color indicates whether the fold-change for the gene is nominally higher in protected vaccine recipients (green) or non-protected vaccine recipients (red). Node size is proportional to the number of ratios that the particular gene appears in. Network visualization was created using Cytoscape (41).

Supplementary Figure 5 Expression profile of Log2(MX2/GPR183) fold-change for RRR and alternative regimen RTS,S vaccine strategies. Shown is the log2 gene expression fold-change for the MX2/GPR183 ratio separated by post-challenge protection status (blue=protected, red=non-protected), Study, and RTS,S vaccination regimen (RRR or alternative). Log2 Fold-changes for MX2/GPR183 were computed comparing expression ratios on Day 1 post-3rd vaccination to pre-vaccination values. Red boxes indicate the two modified RTS,S regimen arms (Study 1 AS02A and Study 5 G4) that did not demonstrate associations between Log2(MX2/GPR183) fold-changes and protection that were observed for the other regimens and studies.

Supplementary Figure 6 Discrimination of protected from non-protected RTS,S recipients based on the Log2(MX2/GPR183) expression fold-change, measured 24 h after the 3rd vaccination. In all plots, the blue line shows the ROC for the logistic regression model fit for the null (STUDY only) model and the green shows the ROC for the logistic regression fit for the full [STUDY+Log2(MX2/GPR183)] model. **(A,B)** ROC for RRR regimen RTS,S for Study 1 (microarray), Study 3 (RNA-Seq), Study 4 (microarray), Study 5 (microarray), and Study 2 RNA-Seq **(A)** or Study 2 microarray **(B)**. **(A)** ROC AUC for null (STUDY only) model (blue) = 0.59, ROC AUC for the STUDY+Log2(MX2/GPR183) model (green) = 0.76, p(ChiSq) = 2 × 10^−5^. **(B)** ROC AUC for null (STUDY only) model (blue) = 0.60, ROC AUC for STUDY+Log2(MX2/GPR183) model (green) = 0.75, p(ChiSq) = 8 × 10^−5^. **(C,D)** ROC for alternative regimen RTS,S for Study 3 RRr (RNA-Seq), Study 5 G2 & G3 (microarray) and Study 2 ARR RNA-Seq **(C)** or Study 2 ARR microarray **(D)**. **(C)** ROC AUC for null (STUDY only) model (blue) = 0.74, ROC AUC for the STUDY+Log2(MX2/GPR183) model (green) = 0.83, p(ChiSq) = 2 × 10^−6^. **(D)** ROC AUC for null (STUDY only) model (blue) = 0.71, ROC AUC for the STUDY+Log2(MX2/GPR183) model (green) = 0.80, p(ChiSq) = 3 × 10^−5^.

Supplementary Figure 7 Overall discrimination of protected from non-protected RTS,S and alternative regimen RTS,S recipients achieved using individual genes, the MX2/GPR183 ratio, or interferon response associated modules. Each line shows the overall ROC for logistic regression model fits obtained for all RRR datasets and the alternative regimen RTS,S groups where MX2/GPR183 behavior was concordant with the RRR group (Study 2 ARR, Study 3 RRr, and Study 5 G2 & G3, as shown in Supplementary Figure 5). For this visualization, both microarray and RNA-Seq data for Study 2 were used. The blue line shows the ROC for the logistic regression model fit for the null (STUDY only) model (ROC AUC = 0.67), the orange line depicts the ROC for the MX2+STUDY model (ROC AUC = 0.78), the purple line depicts the ROC for the GPR183+STUDY model (ROC AUC = 0.76), the red line depicts the ROC for the MX2/GPR183+STUDY model (ROC AUC = 0.82), the bright green line depicts the ROC for a model comprised of the HALLMARK_INTERFERON_GAMMA_RESPONSE module+STUDY (ROC AUC = 0.74), the dark forest green line depicts the ROC for a model comprised of the HALLMARK_INTERFERON_ALPHA_RESPONSE module+STUDY (ROC AUC = 0.74), and the olive green line depicts the ROC for a model comprised of the “M165_enriched in activated dendritic cells (II)” module+STUDY (ROC AUC = 0.72).

Supplementary Figure 8 Temporal Log2 expression fold-change RNA-Seq profile for GPR183 **(A)** and MX2 **(B)** for RRR regimen RTS,S in Study 2. Red lines indicate participants that were not protected, green lines indicate participants that were protected after challenge. Shaded areas indicate 90% confidence intervals for linear mixed models for protected and non-protected vaccine recipients. RTS,S vaccinations were performed on D0, D28, and D56; CHMI was performed on D77. D57 corresponds to Day 1 after the third vaccination, which is the time point used to identify the association between RTS,S-mediated protection and the Log2(MX2/GPR183) score.

Supplementary Figure 9 Discrimination of protected from non-protected RTS,S recipients based on the Log2(MX2/GPR183) expression fold-change (measured 24 h after the 3rd vaccination) in combination with anti-CSP titers (measured on the day of challenge). In all plots, blue line shows the ROC for the logistic regression model fit for a null (STUDY only) model; the purple line shows the ROC for the logistic regression model fit for the model including STUDY and Z-transformed anti-CSP titers (STUDY+ANTI-CSP); the green line shows the ROC for the logistic regression fit of the STUDY+Log2(MX2/GPR183) model; and the red line shows the ROC for the logistic regression fit for the full model [STUDY+ANTI-CSP+Log2(MX2/GPR183)]. **(A,B)** ROC for RRR regimen RTS,S for Study 3 (RNA-Seq), Study 4 (microarray), Study 5 (microarray), and Study 2 RNA-Seq **(A)** or Study 2 microarray **(B)**. **(A)** ROC AUC for null (STUDY only) model (blue) = 0.60, ROC AUC for STUDY+ANTI-CSP model (purple) = 0.80, ROC AUC for STUDY+Log2(MX2/GPR183) model (green) = 0.78, ROC AUC for full model (red) = 0.87. p[ChiSq, red [STUDY+ANTI-CSP+Log2(MX2/GPR183) vs. purple (STUDY+ANTI-CSP)] = 0.001. **(B)** ROC AUC for the null (STUDY only) model (blue) = 0.60, ROC AUC for the STUDY+ANTI-CSP model (purple) = 0.81, ROC AUC for the STUDY+Log2(MX2/GPR183) model (green) = 0.76, ROC AUC for the full model (red) = 0.87. p[ChiSq, red [STUDY+ANTI-CSP+Log2(MX2/GPR183)] vs. purple (STUDY+ANTI-CSP)] = 0.005. **(C,D)** ROC for alternative regimen RTS,S for Study 3 RRr (RNA-Seq), Study 5 G2 & G3 (microarray, treated together) and Study 2 ARR RNA-Seq **(C)** or Study 2 ARR microarray **(D)**. **(C)** ROC AUC for null (STUDY only) model (blue) = 0.74, ROC AUC for the STUDY+ANTI-CSP model (purple) = 0.81, ROC AUC for the STUDY+Log2(MX2/GPR183) model (green) = 0.83, ROC AUC for the full model (red) = 0.86. p(ChiSq, red) [STUDY+ANTI-CSP+Log2(MX2/GPR183)] vs. purple (STUDY+ANTI-CSP)] = 0.002. **(D)** ROC AUC for null (STUDY only) model (blue) = 0.71, ROC AUC for the STUDY+ANTI-CSP model (purple) = 0.78, ROC AUC for the STUDY+Log2(MX2/GPR183) model (green) = 0.80, ROC AUC for the full model (red) = 0.84. p(ChiSq, red) [STUDY+ANTI-CSP+Log2(MX2/GPR183)] vs. purple (STUDY+ANTI-CSP) = 0.003.

Supplementary Figure 10 Correlation between RNA-Seq and microarray-based assessments of MX2, GPR183, and the MX2/GPR183 ratio in Study 2. Within the totality of the Study 2 dataset (all time points, all participants, both RRR and ARR study arms) there were 613 samples with matching RNA-Seq and microarray measurements, allowing for robust cross-platform correlation of normalized data. **(A)** RNA-Seq vs. microarray correlation for MX2 (Spearman Rho = 0.96, *p* = 0, *N* = 613); **(B)** RNA-Seq RNA-Seq vs. microarray correlation for GPR183 (Spearman Rho = 0.86, *p* = 0, *N* = 613), **(C)** RNA-Seq vs. microarray correlation for the MX2/GPR183 ratio (Spearman Rho = 0.92, *p* = 0, *N* = 613).

Supplementary Table 1 Experimental design summaries for the five RTS,S CHMI transcriptional profiling studies evaluated in the meta-analysis.

Supplementary Table 2 Transcriptional analysis time points for the five RTS,S CHMI studies and how the time points relate to each vaccination. Time points that were assessed in more than one study are highlighted in orange; the two time points that were assessed in all studies are highlighted in yellow. Alternative arms lacking samples for protected or non-protected subjects are highlighted in red. The number of samples for non-protected (“NP”) or protected (“P”) subjects are indicated.

Supplementary Table 3 quantitative real-time PCR (qRT-PCR) confirmation of Study 2 and Study 3 RNA-Seq. (Top) Genes and associated Taqman primer/probe sets that were used as housekeeping genes to normalize Ct values. (Bottom) Gene-level spearman rank correlation statistics (Rho, *p*-value, and number of data points) for correlations between normalized qRT-PCR data and (Left) Study 2 microarray data, (Middle) Study 2 RNA-Seq data, and (Right) Study 3 RNA-Seq data. Taqman primer/probe sets used for each individual gene are indicated. For Study 2, data for 21 participants at up to two time points (pre-vaccination and day of 3rd vaccination) were assessed for a total of 38 data points for each gene. For Study 3, data for 33 participants at one or more of 5 time points (pre-vaccination, the day of the third vaccination, 3 or 14 days after the third vaccination, and the day of challenge) were assessed for a total of 110 data points for each gene.

Supplementary Table 4 Transcriptional modules exhibiting coherent patterns of expression within the five RTS,S CHMI studies.

Supplementary Table 5 Logistic regression statistics for discriminating protected from non-protected recipients of RRR regimen RTS,S. Shown are results for all 79 modules and representative data for ratios between modules and individual genes that achieved ROC AUC > 0.5. All analyses used log2 fold-changes comparing the expression values of the transcriptional variable at Day 1 post-3rd vaccination to pre-vaccination values.

Supplementary Table 6 Logistic regression statistics for discriminating protected from non-protected recipients of RRR regimen RTS,S based on transcriptional signatures comprised of ratios between genes and transcriptional modules. Shown are results for 241 selected gene/module ratios that survived multiple testing correction at FDR <20.1% and the minimum ROC AUC across all studies and platforms was >0.65. All analyses used log2 fold-changes comparing the expression values of the transcriptional variable at Day 1 post-3rd vaccination to pre-vaccination values.

Supplementary Table 7 Logistic regression statistics for discriminating protected from non-protected recipients of RRR regimen RTS,S based on transcriptional signatures comprised of ratios between pairs of transcripts. These transcript pairs were generated by expanding the modules in the selected gene/module ratios (Supplementary Table 6) by the cognate genes that comprise each module (Supplementary Table 4). Some transcripts may appear in more than one module. Shown are results for all 2,727 transcript/transcript ratios that are derived in this manner. All analyses used log2 fold-changes comparing the expression values of the transcriptional variable at Day 1 post-3rd vaccination to pre-vaccination values.

Supplementary Table 8 Logistic regression statistics for discriminating protected from non-protected recipients of alternative regimen RTS,S vaccination strategies using the 247 transcript/transcript signatures that were selected from the RRR regimen analysis (Supplementary Table 7). All analyses used Log2 fold-changes comparing the expression values of the transcriptional variable at Day 1 post-3rd vaccination to pre-vaccination values.

Supplementary Table 9 Area under the receiver operating characteristic curves (ROC AUC) for discriminating protected from non-protected recipients of RRR and alternative regimen RTS,S vaccinations using MX2/GPR183 fold-changes measured at alternative time points. Shown are ROC AUCs for time points that were assessed in more than one independent study. ROC AUC values in bold indicate moderate discrimination (ROC AUC > 0.65); ROC AUC values in red indicate anti-prediction (discrimination in the wrong direction; ROC AUC <0.5). Measurements of MX2/GPR183 fold-changes at the time points highlighted in yellow achieve nominally significant discrimination for at least two RRR regimen RTS,S cohorts.
