# Supplementary figures and images for: The Ratiometric Transcript Signature MX2/GPR183 Is Consistently Associated With RTS,S-Mediated Protection Against Controlled Human Malaria Infection
Source: Front Immunol. 2020 Apr 28;11:669. doi: 10.3389/fimmu.2020.00669 (PMC7199517; doi:10.3389/fimmu.2020.00669)

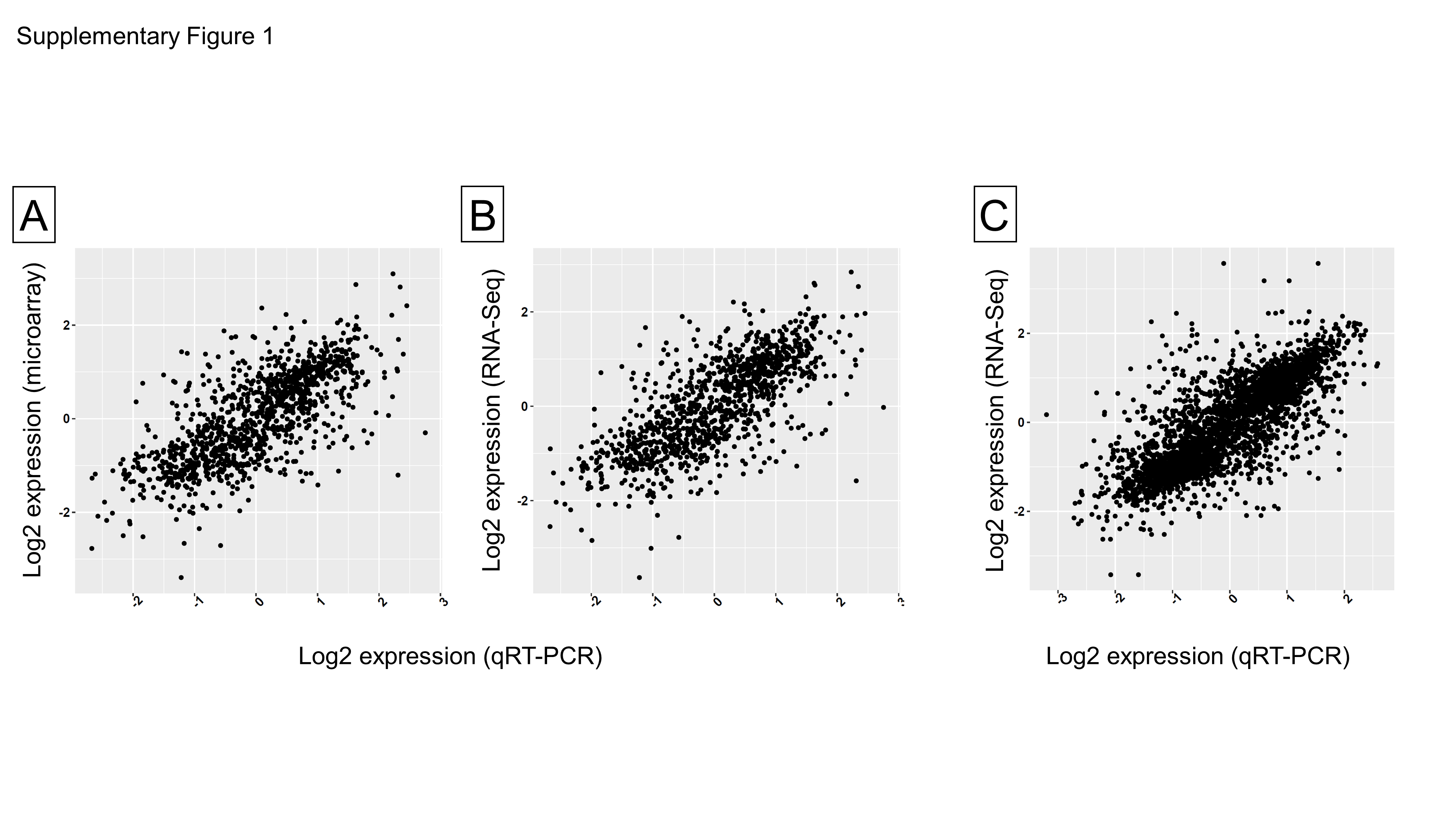

Supplement: Supplementary Figure 1 — Overall correlation between quantitative real-time PCR (qRT-PCR) and microarray or RNA-Seq data for Study 2 and Study 3. For a panel of 31 genes with roles in the inflammatory, immune response, and other diverse functions (Supplementary Table 3), qRT-PCR data was generated for 21 participants from Study 2 at up to two time points (pre-vaccination and day of 3rd vaccination) and for 33 participants from Study 3 at one or more of 5 time points (pre-vaccination, the day of the third vaccination, 3 or 14 days after the third vaccination, and the day of challenge), giving a total of 1,178 data points for Study 2 and 3,410 data points for Study 3. For overall cross-platform comparisons, normalized data on the log2 scale were z-transformed for each gene and then combined. (A) Study 2 microarray vs. qRT-PCR (Spearman Rho = 0.73, p = 0, N = 1178), (B) Study 2 RNA-Seq vs. qRT-PCR (Spearman Rho = 0.74, p = 0, N = 1178), (C) Study 3 RNA-Seq vs. qRT-PCR (Spearman Rho = 0.81, p = 0, N = 3410). Individual gene-level correlations are provided in Supplementary Table 3. [file Image_1.tif]

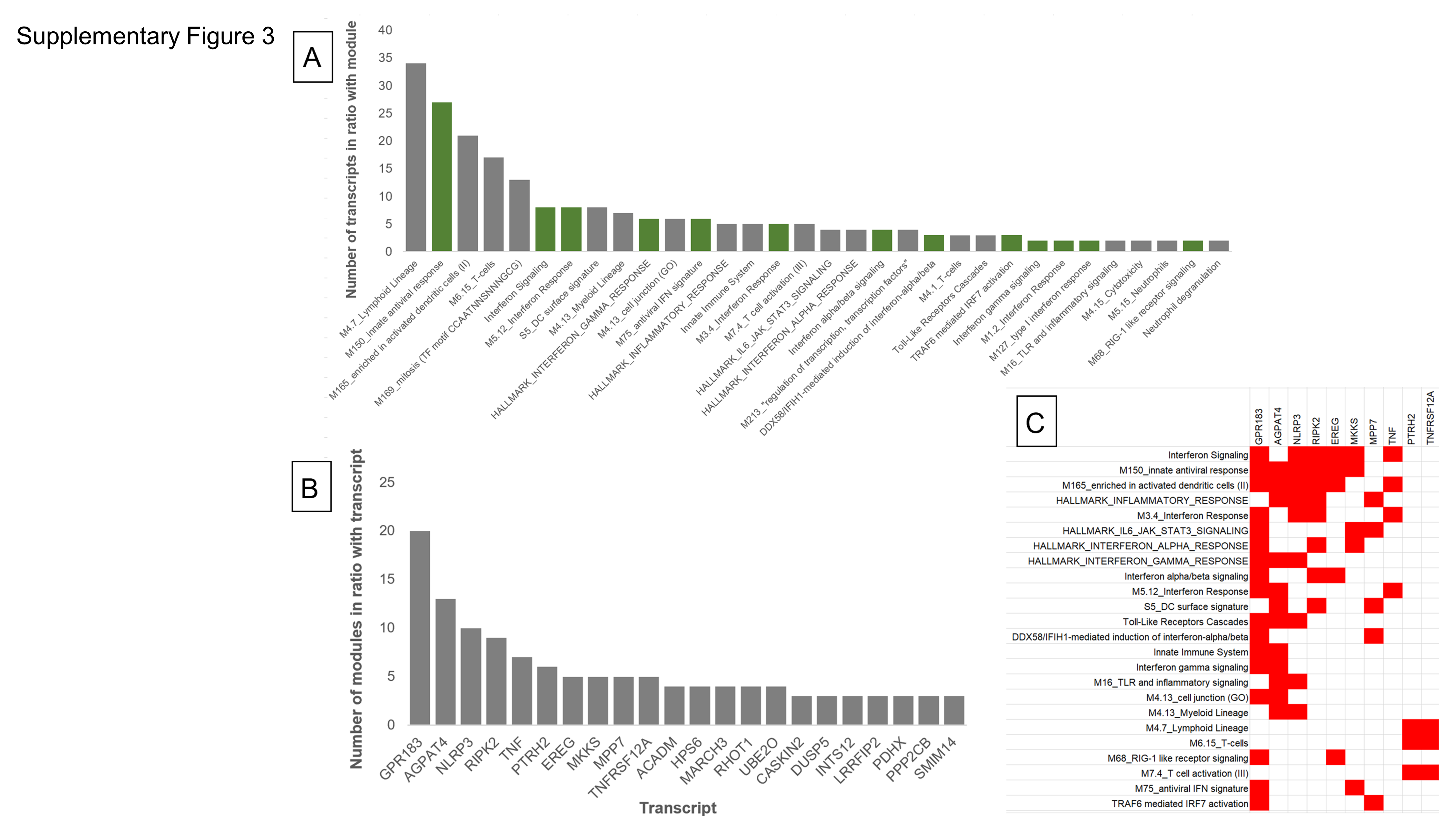

Supplement: Supplementary Figure 3 — Frequency of individual modules and transcripts in the transcript/module ratios associated with protection after RTS,S vaccination. (A) Barplot depicting the number of significant transcript/module ratios in which specific modules appeared. While a lymphoid lineage module was individually the most frequent module, numerous antiviral/interferon response modules appeared frequently (shown in green). (B) Barplot depicting the number of significant transcript/module ratios in which specific transcripts appeared. The oxysterol receptor GPR183 was the most frequently selected gene. (C) Heatmap depicting the transcript/module ratios for transcripts and modules that were selected frequently. The top 5 transcripts (GPR183, AGPAT4, NLRP3, RIPK2, and TNF) appeared in significant ratios with interferon and viral response-associated modules. [file Image_3.tif]

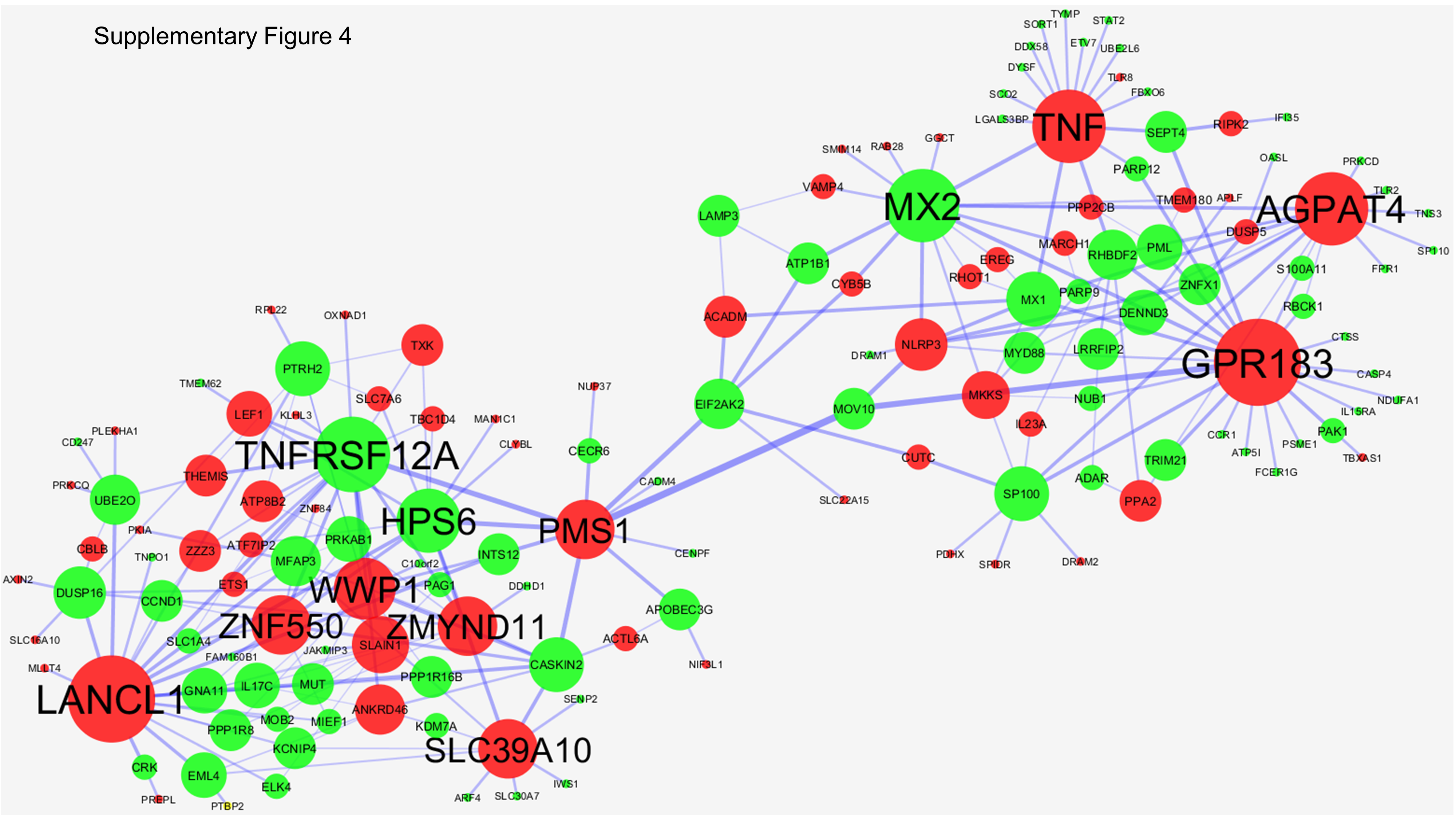

Supplement: Supplementary Figure 4 — Network representation of 247 transcript/transcript ratios that were selected based on consistent discrimination of protected from non-protected recipients of alternative regimen RTS,S vaccination. Each node (circle) represents an individual gene. The presence of an edge (line) between nodes indicates that transcriptional fold-change ratios (Day 1 after 3rd vaccination compared to pre-vaccination) between those genes consistently discriminate protected from non-protected recipients of RRR regimen RTS,S (Supplementary Table 7). Node color indicates whether the fold-change for the gene is nominally higher in protected vaccine recipients (green) or non-protected vaccine recipients (red). Node size is proportional to the number of ratios that the particular gene appears in. Network visualization was created using Cytoscape (41). [file Image_4.tif]

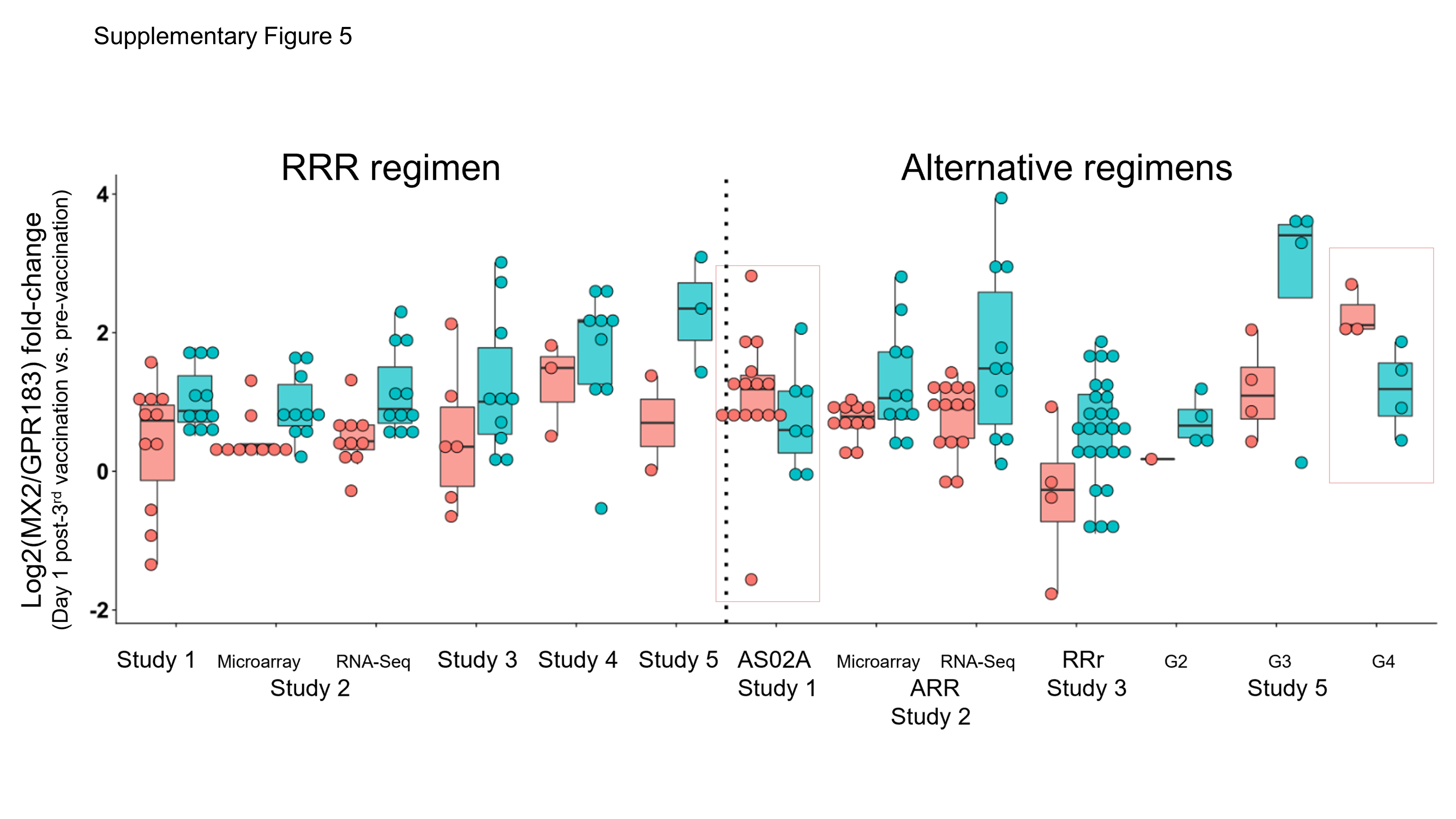

Supplement: Supplementary Figure 5 — Expression profile of Log2(MX2/GPR183) fold-change for RRR and alternative regimen RTS,S vaccine strategies. Shown is the log2 gene expression fold-change for the MX2/GPR183 ratio separated by post-challenge protection status (blue=protected, red=non-protected), Study, and RTS,S vaccination regimen (RRR or alternative). Log2 Fold-changes for MX2/GPR183 were computed comparing expression ratios on Day 1 post-3rd vaccination to pre-vaccination values. Red boxes indicate the two modified RTS,S regimen arms (Study 1 AS02A and Study 5 G4) that did not demonstrate associations between Log2(MX2/GPR183) fold-changes and protection that were observed for the other regimens and studies. [file Image_5.tif]

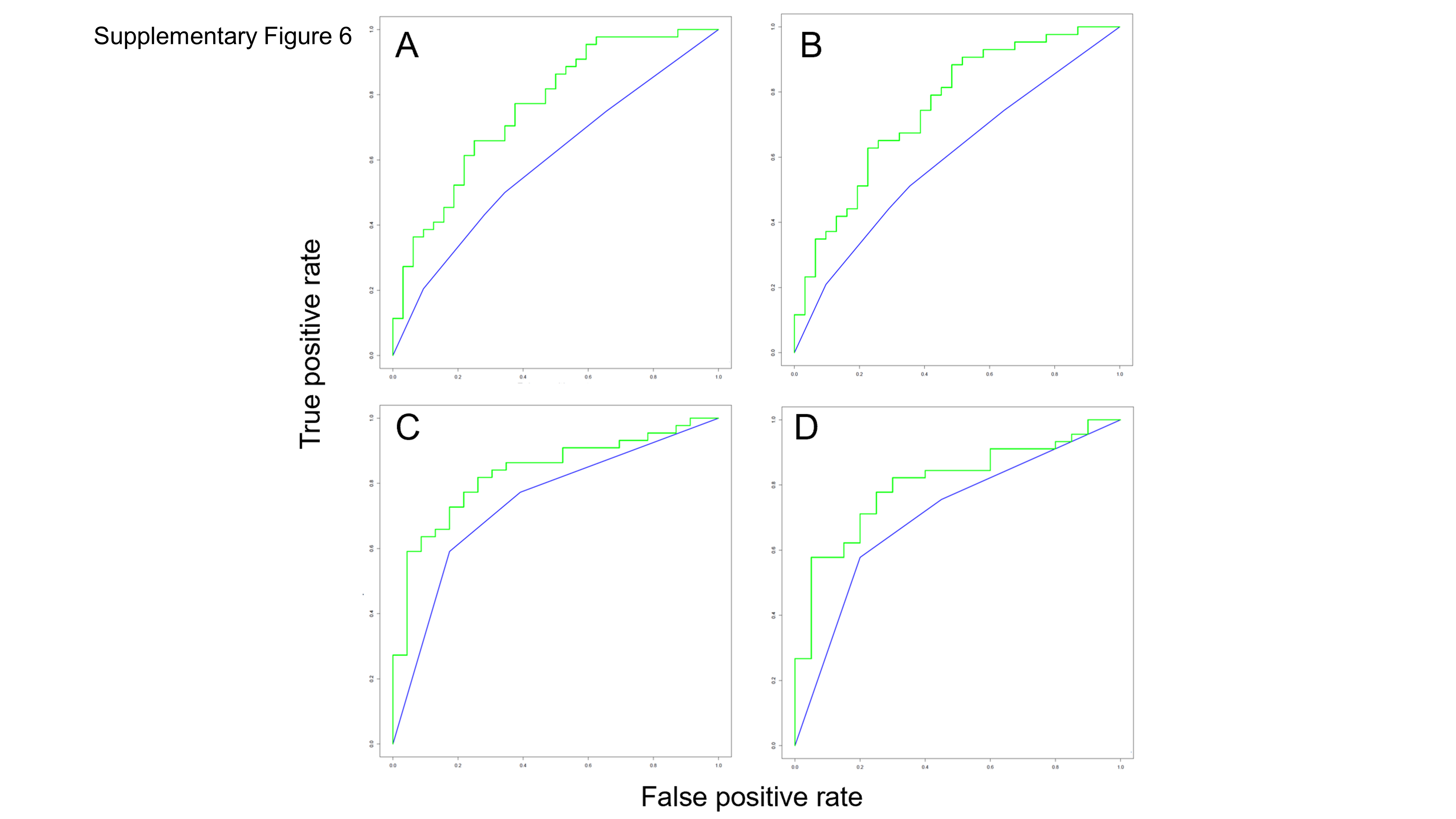

Supplement: Supplementary Figure 6 — Discrimination of protected from non-protected RTS,S recipients based on the Log2(MX2/GPR183) expression fold-change, measured 24 h after the 3rd vaccination. In all plots, the blue line shows the ROC for the logistic regression model fit for the null (STUDY only) model and the green shows the ROC for the logistic regression fit for the full [STUDY+Log2(MX2/GPR183)] model. (A,B) ROC for RRR regimen RTS,S for Study 1 (microarray), Study 3 (RNA-Seq), Study 4 (microarray), Study 5 (microarray), and Study 2 RNA-Seq (A) or Study 2 microarray (B). (A) ROC AUC for null (STUDY only) model (blue) = 0.59, ROC AUC for the STUDY+Log2(MX2/GPR183) model (green) = 0.76, p(ChiSq) = 2 × 10^−5^. (B) ROC AUC for null (STUDY only) model (blue) = 0.60, ROC AUC for STUDY+Log2(MX2/GPR183) model (green) = 0.75, p(ChiSq) = 8 × 10^−5^. (C,D) ROC for alternative regimen RTS,S for Study 3 RRr (RNA-Seq), Study 5 G2 & G3 (microarray) and Study 2 ARR RNA-Seq (C) or Study 2 ARR microarray (D). (C) ROC AUC for null (STUDY only) model (blue) = 0.74, ROC AUC for the STUDY+Log2(MX2/GPR183) model (green) = 0.83, p(ChiSq) = 2 × 10^−6^. (D) ROC AUC for null (STUDY only) model (blue) = 0.71, ROC AUC for the STUDY+Log2(MX2/GPR183) model (green) = 0.80, p(ChiSq) = 3 × 10^−5^. [file Image_6.tif]

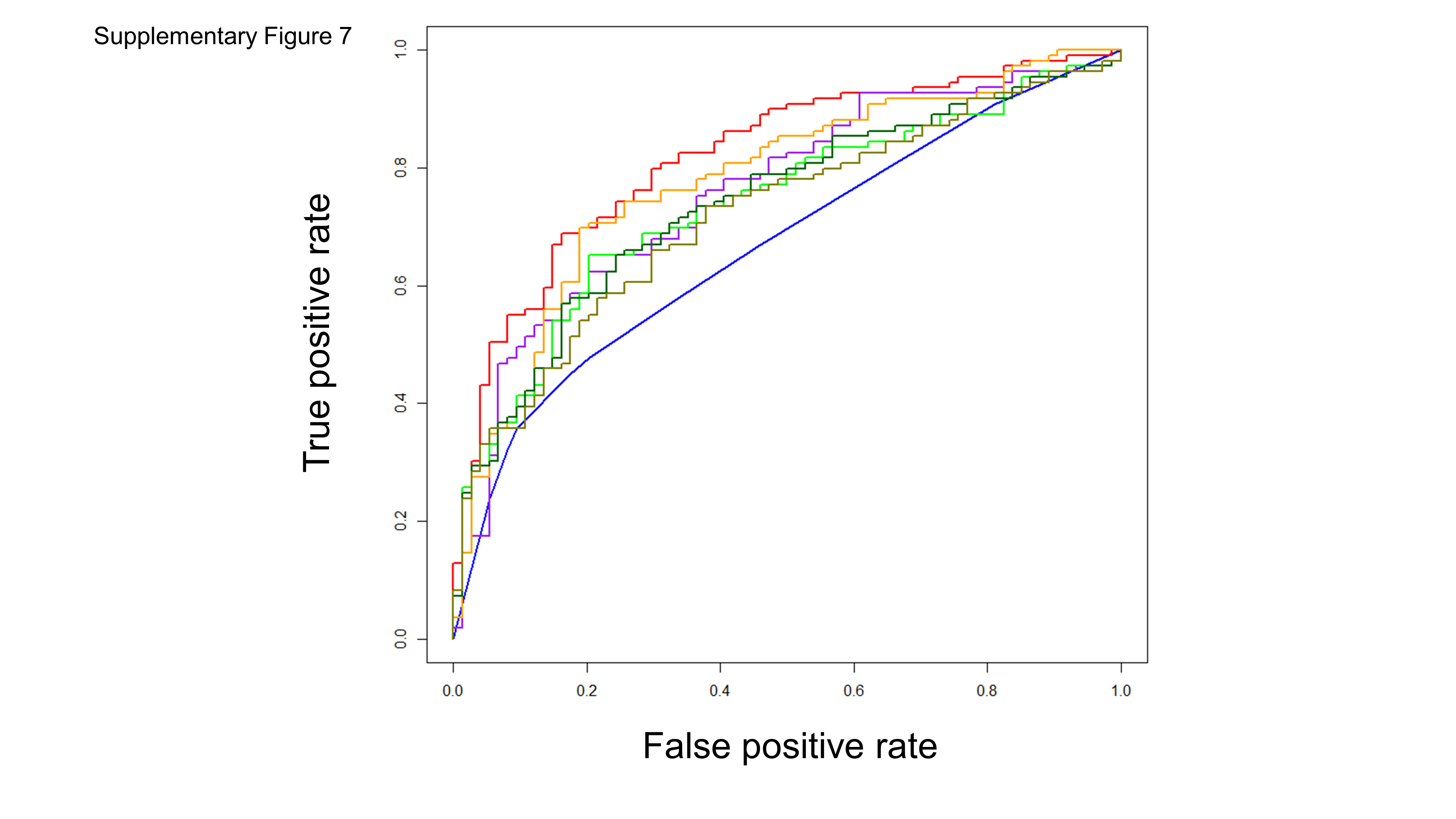

Supplement: Supplementary Figure 7 — Overall discrimination of protected from non-protected RTS,S and alternative regimen RTS,S recipients achieved using individual genes, the MX2/GPR183 ratio, or interferon response associated modules. Each line shows the overall ROC for logistic regression model fits obtained for all RRR datasets and the alternative regimen RTS,S groups where MX2/GPR183 behavior was concordant with the RRR group (Study 2 ARR, Study 3 RRr, and Study 5 G2 & G3, as shown in Supplementary Figure 5). For this visualization, both microarray and RNA-Seq data for Study 2 were used. The blue line shows the ROC for the logistic regression model fit for the null (STUDY only) model (ROC AUC = 0.67), the orange line depicts the ROC for the MX2+STUDY model (ROC AUC = 0.78), the purple line depicts the ROC for the GPR183+STUDY model (ROC AUC = 0.76), the red line depicts the ROC for the MX2/GPR183+STUDY model (ROC AUC = 0.82), the bright green line depicts the ROC for a model comprised of the HALLMARK_INTERFERON_GAMMA_RESPONSE module+STUDY (ROC AUC = 0.74), the dark forest green line depicts the ROC for a model comprised of the HALLMARK_INTERFERON_ALPHA_RESPONSE module+STUDY (ROC AUC = 0.74), and the olive green line depicts the ROC for a model comprised of the “M165_enriched in activated dendritic cells (II)” module+STUDY (ROC AUC = 0.72). [file Image_7.tif]

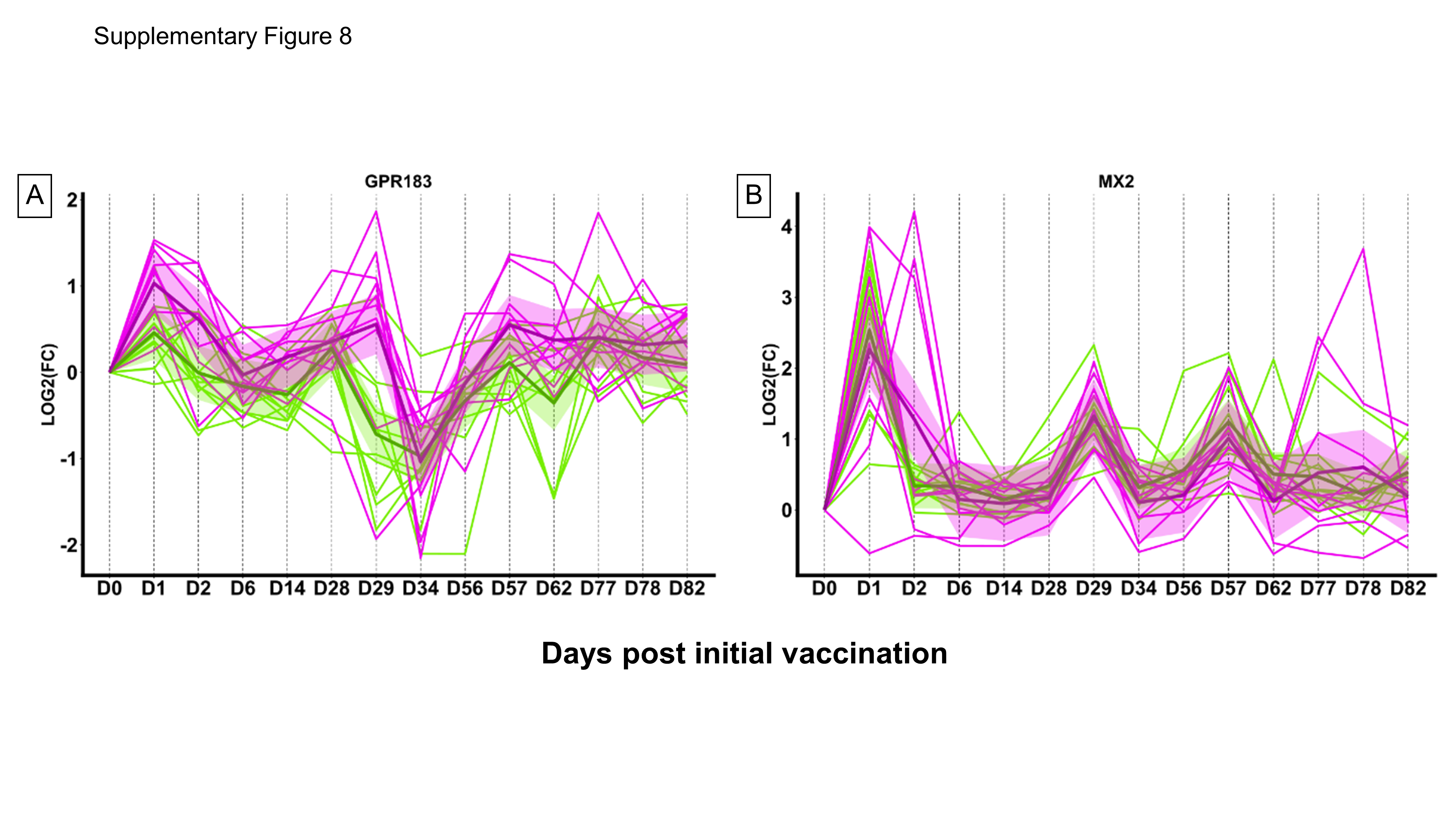

Supplement: Supplementary Figure 8 — Temporal Log2 expression fold-change RNA-Seq profile for GPR183 (A) and MX2 (B) for RRR regimen RTS,S in Study 2. Red lines indicate participants that were not protected, green lines indicate participants that were protected after challenge. Shaded areas indicate 90% confidence intervals for linear mixed models for protected and non-protected vaccine recipients. RTS,S vaccinations were performed on D0, D28, and D56; CHMI was performed on D77. D57 corresponds to Day 1 after the third vaccination, which is the time point used to identify the association between RTS,S-mediated protection and the Log2(MX2/GPR183) score. [file Image_8.tif]

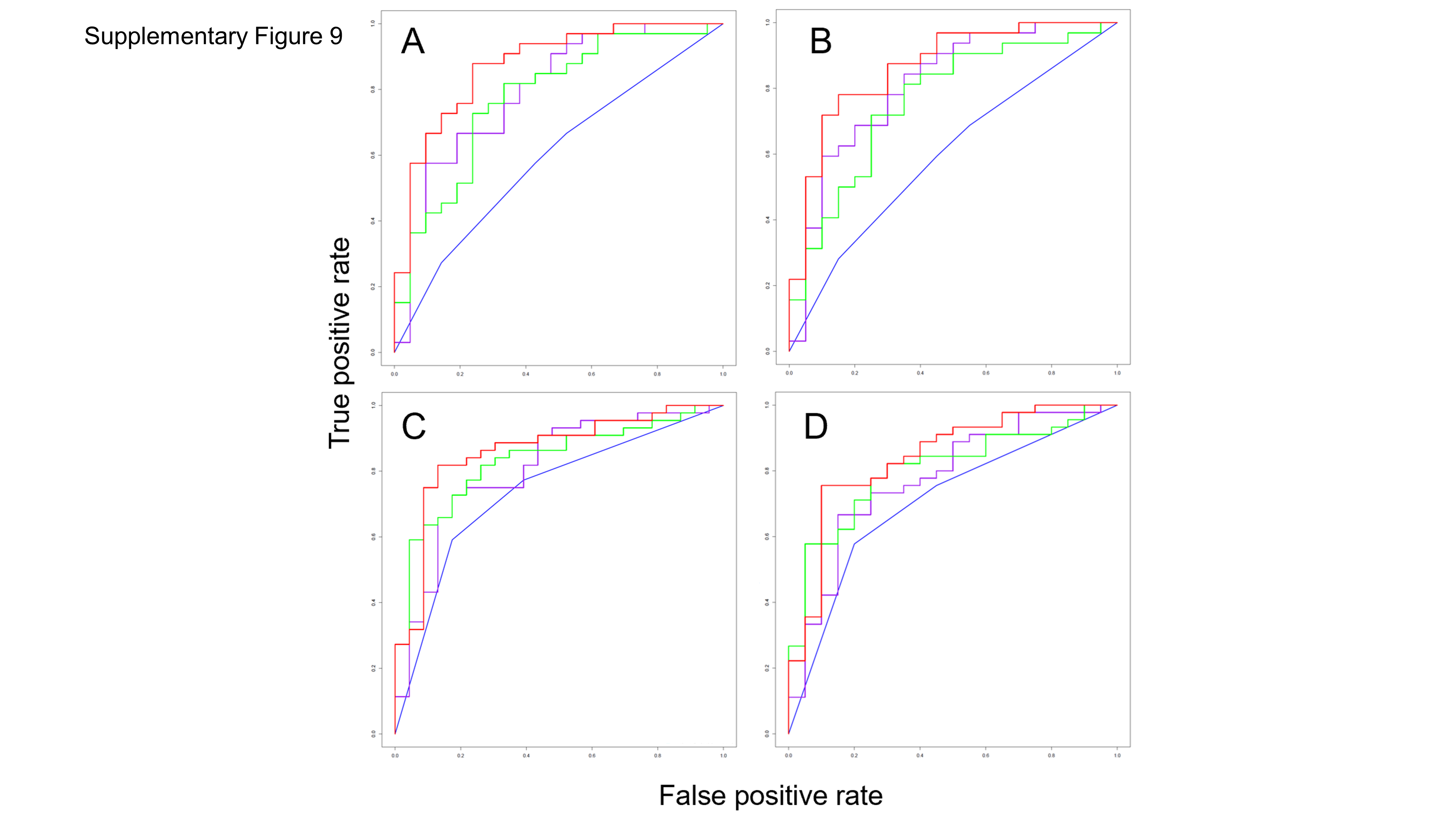

Supplement: Supplementary Figure 9 — Discrimination of protected from non-protected RTS,S recipients based on the Log2(MX2/GPR183) expression fold-change (measured 24 h after the 3rd vaccination) in combination with anti-CSP titers (measured on the day of challenge). In all plots, blue line shows the ROC for the logistic regression model fit for a null (STUDY only) model; the purple line shows the ROC for the logistic regression model fit for the model including STUDY and Z-transformed anti-CSP titers (STUDY+ANTI-CSP); the green line shows the ROC for the logistic regression fit of the STUDY+Log2(MX2/GPR183) model; and the red line shows the ROC for the logistic regression fit for the full model [STUDY+ANTI-CSP+Log2(MX2/GPR183)]. (A,B) ROC for RRR regimen RTS,S for Study 3 (RNA-Seq), Study 4 (microarray), Study 5 (microarray), and Study 2 RNA-Seq (A) or Study 2 microarray (B). (A) ROC AUC for null (STUDY only) model (blue) = 0.60, ROC AUC for STUDY+ANTI-CSP model (purple) = 0.80, ROC AUC for STUDY+Log2(MX2/GPR183) model (green) = 0.78, ROC AUC for full model (red) = 0.87. p[ChiSq, red [STUDY+ANTI-CSP+Log2(MX2/GPR183) vs. purple (STUDY+ANTI-CSP)] = 0.001. (B) ROC AUC for the null (STUDY only) model (blue) = 0.60, ROC AUC for the STUDY+ANTI-CSP model (purple) = 0.81, ROC AUC for the STUDY+Log2(MX2/GPR183) model (green) = 0.76, ROC AUC for the full model (red) = 0.87. p[ChiSq, red [STUDY+ANTI-CSP+Log2(MX2/GPR183)] vs. purple (STUDY+ANTI-CSP)] = 0.005. (C,D) ROC for alternative regimen RTS,S for Study 3 RRr (RNA-Seq), Study 5 G2 & G3 (microarray, treated together) and Study 2 ARR RNA-Seq (C) or Study 2 ARR microarray (D). (C) ROC AUC for null (STUDY only) model (blue) = 0.74, ROC AUC for the STUDY+ANTI-CSP model (purple) = 0.81, ROC AUC for the STUDY+Log2(MX2/GPR183) model (green) = 0.83, ROC AUC for the full model (red) = 0.86. p(ChiSq, red) [STUDY+ANTI-CSP+Log2(MX2/GPR183)] vs. purple (STUDY+ANTI-CSP)] = 0.002. (D) ROC AUC for null (STUDY only) model (blue) = 0.71, ROC AUC for the STUDY+ANTI-CSP model (p [file Image_9.tif]

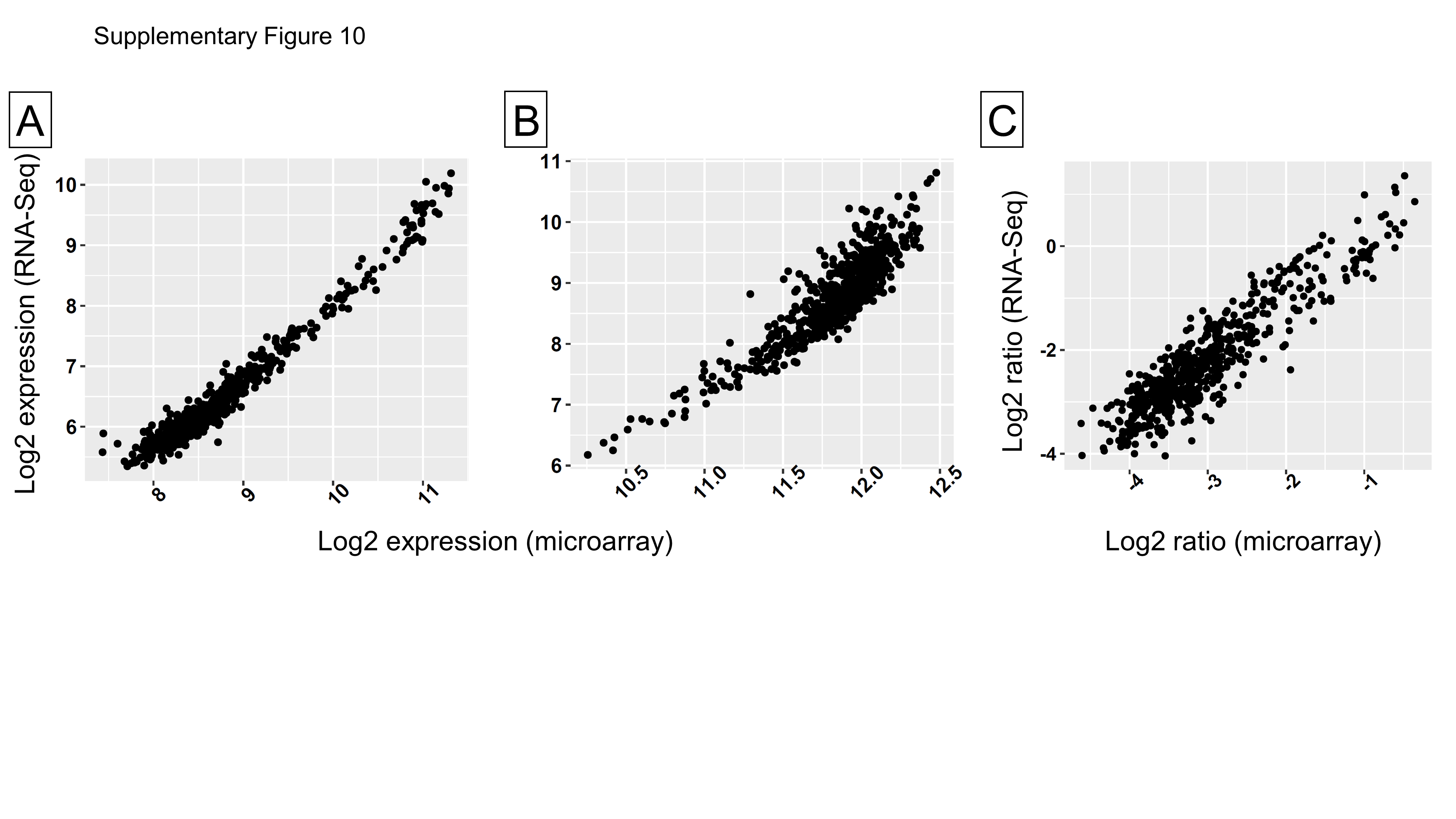

Supplement: Supplementary Figure 10 — Correlation between RNA-Seq and microarray-based assessments of MX2, GPR183, and the MX2/GPR183 ratio in Study 2. Within the totality of the Study 2 dataset (all time points, all participants, both RRR and ARR study arms) there were 613 samples with matching RNA-Seq and microarray measurements, allowing for robust cross-platform correlation of normalized data. (A) RNA-Seq vs. microarray correlation for MX2 (Spearman Rho = 0.96, p = 0, N = 613); (B) RNA-Seq RNA-Seq vs. microarray correlation for GPR183 (Spearman Rho = 0.86, p = 0, N = 613), (C) RNA-Seq vs. microarray correlation for the MX2/GPR183 ratio (Spearman Rho = 0.92, p = 0, N = 613). [file Image_10.tif]
