# Supplementary material for: The Ratiometric Transcript Signature MX2/GPR183 Is Consistently Associated With RTS,S-Mediated Protection Against Controlled Human Malaria Infection
Source: Front Immunol. 2020 Apr 28;11:669. doi: 10.3389/fimmu.2020.00669 (PMC7199517; doi:10.3389/fimmu.2020.00669)
Supplement: Supplementary Figure 2 — Temporal expression profiles for coherent transcriptional modules in all studies. For each coherent module defined in Supplementary Table 4, Log2 expression fold changes across all genes within the module were computed for each volunteer at each time point. Time course plots depict trajectories of module-average expression for each volunteer (thin lines) and the overall averages across all volunteers. Shown is a representative plot for an individual module (“HALLMARK_INTERFERON_GAMMA_RESPONSE”). For the complete set of module expression profiles, please see: Supplementary Tables 1–9. [file Image_2.pdf]

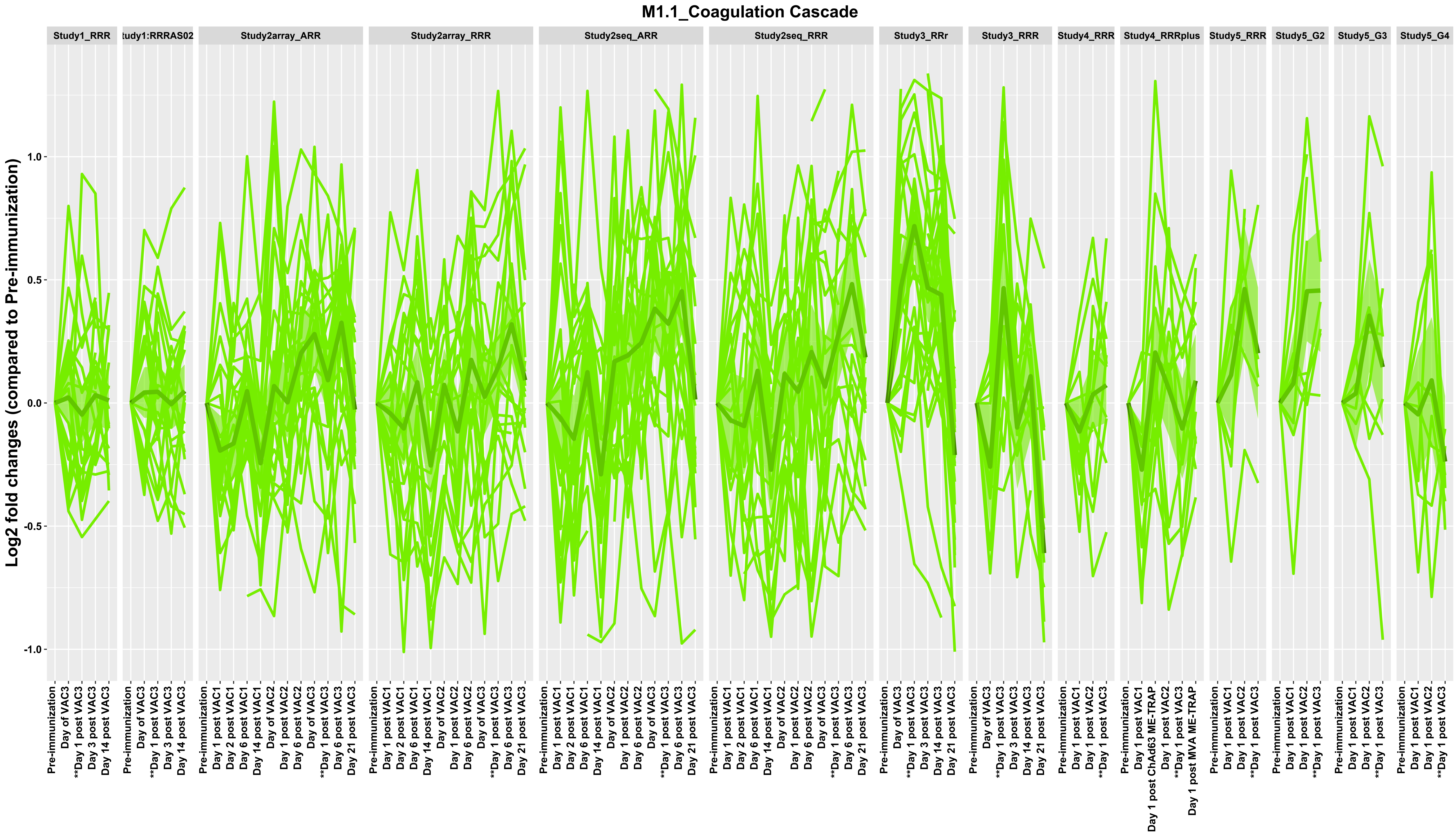

M1.1_Coagulation Cascade
Study1_RRR
tudy1:RRRAS02
Study2array_ARR
Study2array_RRR
Study2seq_ARR
Study2seq_RRR
Study3_RRr
Study3_RRR
Study4_RRR
Study4_RRRplus
Study5_RRR
Study5_G2
Study5_G3
Study5_G4
Log2 fold changes (compared to Pre-immunization)
1.0
0.5
0.0
-0.5
-1.0
Pre-immunization
Day of VAC3
**Day 1 post VAC3
Day 3 post VAC3
Day 14 post VAC3
Day 1 post VAC1
Day 2 post VAC1
Day 6 post VAC1
Day 14 post VAC1
Day of VAC2
Day 1 post VAC2
Day 6 post VAC2
Day 6 post VAC3
Day 21 post VAC3
Day 1 post ChAd63 ME-TRAP
Day 1 post MVA ME-TRAP
**Day 1 post VAC2

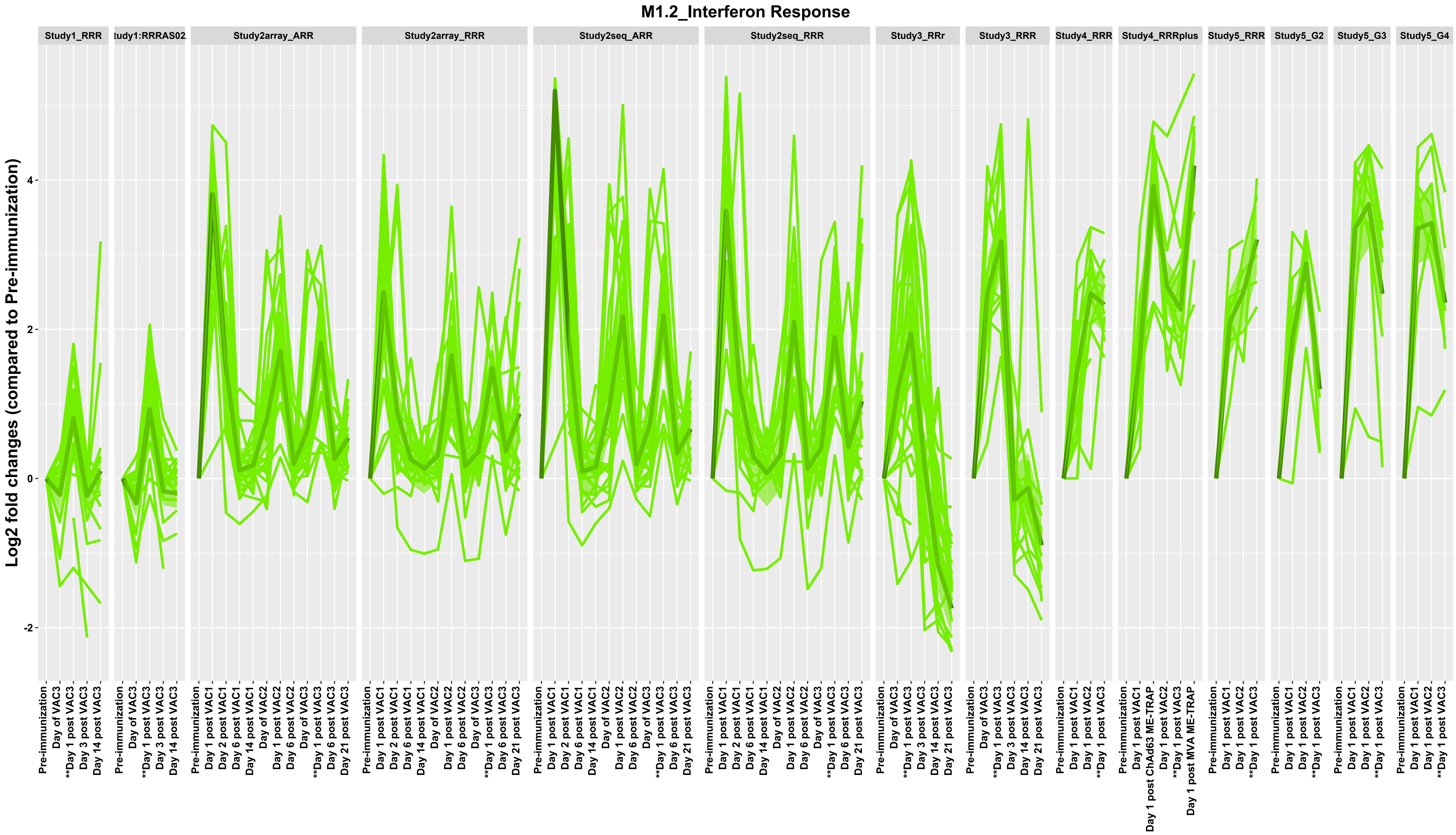
M1.2_Interferon Response
Study1_RRR
tudy1:RRRAS02
Study2array_ARR
Study2array_RRR
Study2seq_ARR
Study2seq_RRR
Study3_RRr
Study3_RRR
Study4_RRR
Study4_RRRplus
Study5_RRR
Study5_G2
Study5_G3
Study5_G4
Log2 fold changes (compared to Pre-immunization)
4
2
0
-2
Pre-immunization
Day of VAC3
**Day 1 post VAC3
Day 3 post VAC3
Day 14 post VAC3
Day 1 post VAC1
Day 2 post VAC1
Day 6 post VAC1
Day 14 post VAC1
Day of VAC2
Day 1 post VAC2
Day 6 post VAC2
Day 6 post VAC3
Day 21 post VAC3
Day 1 post VAC3
Day 1 post ChAd63 ME-TRAP
**Day 1 post VAC2
Day 1 post MVA ME-TRAP

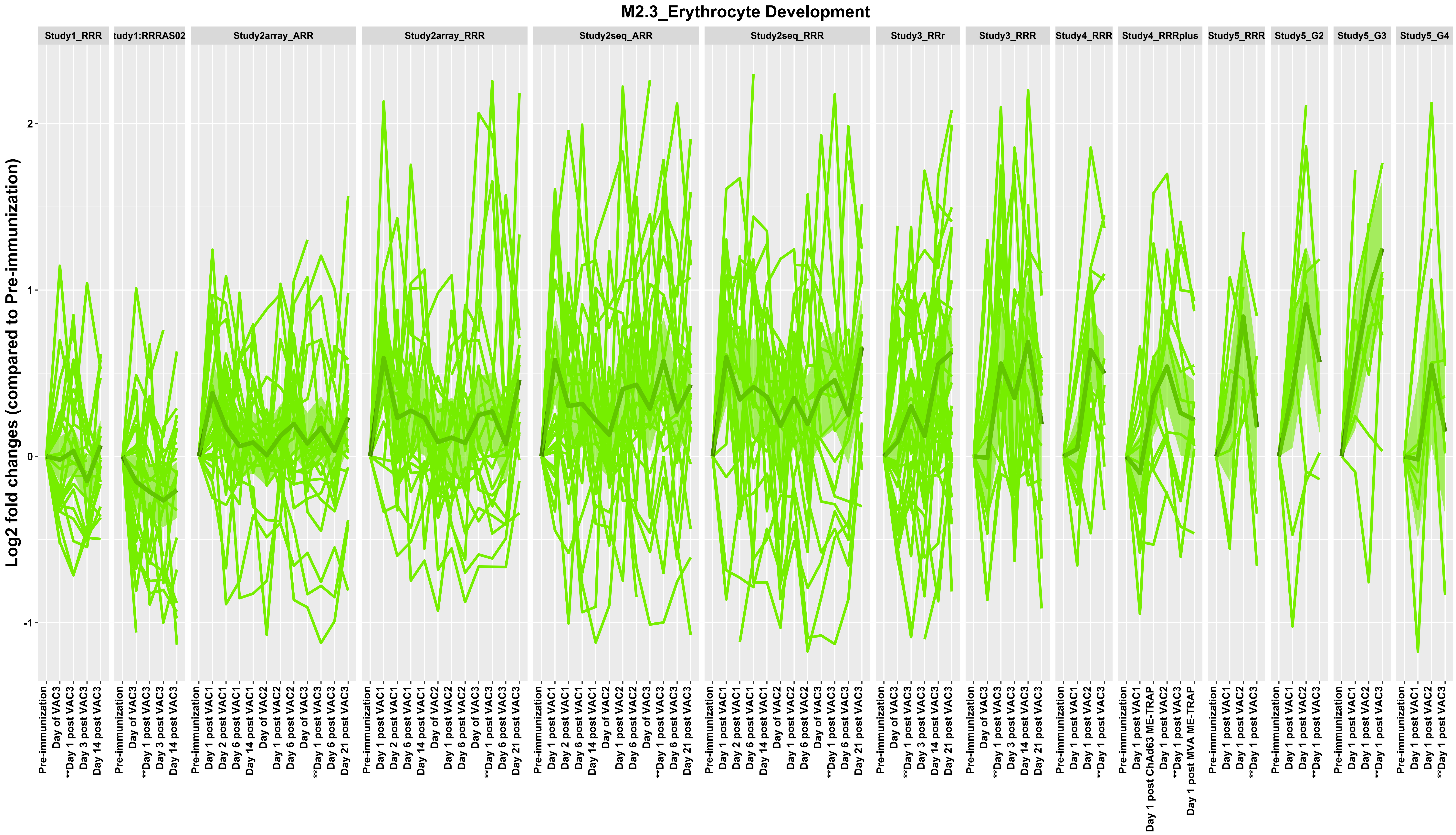

M2.3_Erythrocyte Development
Study1_RRR
tudy1:RRRAS02
Study2array_ARR
Study2array_RRR
Study2seq_ARR
Study2seq_RRR
Study3_RRr
Study3_RRR
Study4_RRR
Study4_RRRplus
Study5_RRR
Study5_G2
Study5_G3
Study5_G4
Log2 fold changes (compared to Pre-immunization)
2
1
0
-1
Pre-immunization
Day of VAC3
**Day 1 post VAC3
Day 3 post VAC3
Day 14 post VAC3
Day 1 post VAC1
Day 2 post VAC1
Day 6 post VAC1
Day 14 post VAC1
Day of VAC2
Day 1 post VAC2
Day 6 post VAC2
Day 6 post VAC3
Day 21 post VAC3
**Day 1 post VAC2
Day 1 post ChAd63 ME-TRAP
Day 1 post MVA ME-TRAP

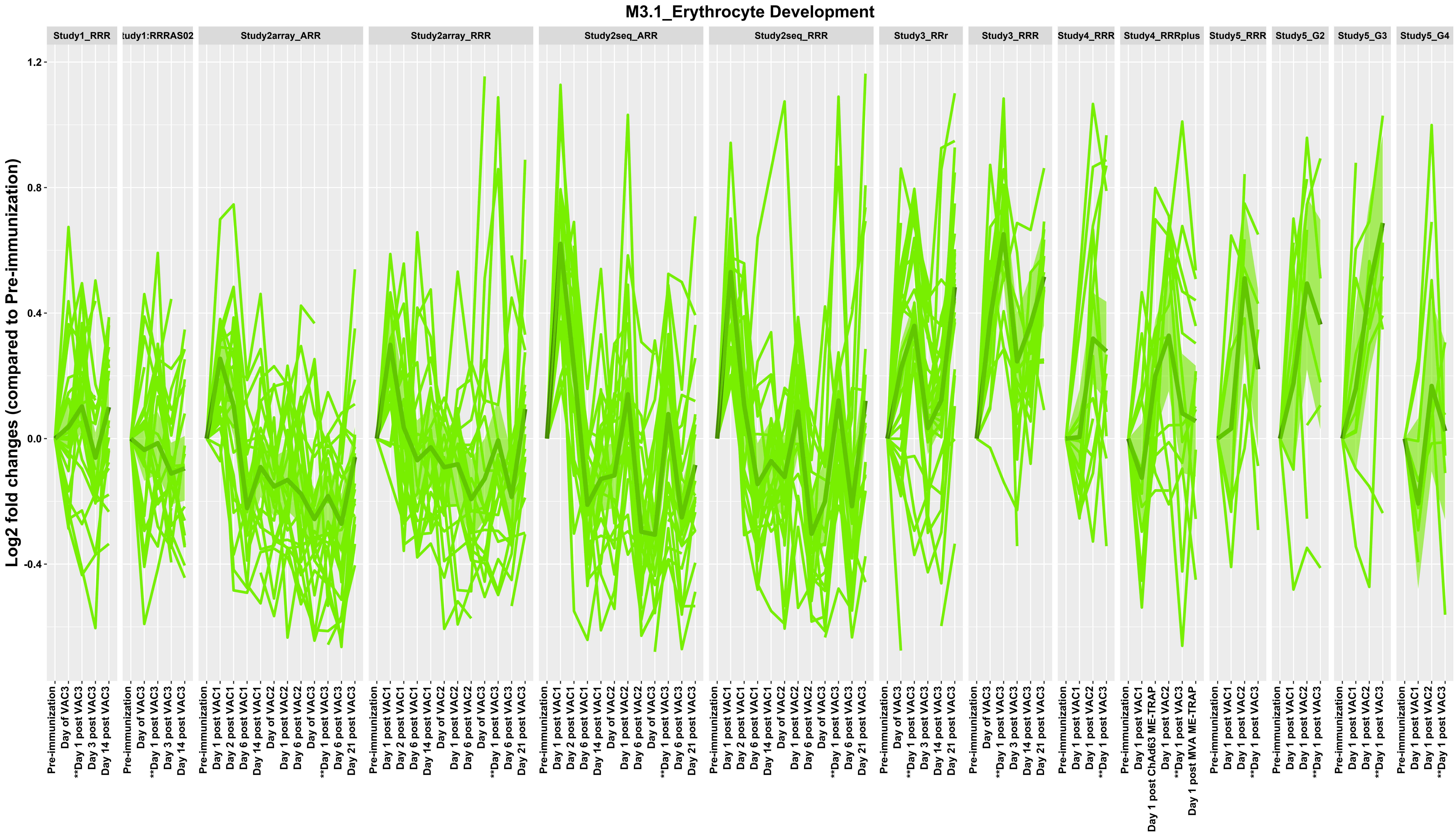

M3.1_Erythrocyte Development
Log2 fold changes (compared to Pre-immunization)
Study1_RRR
tudy1:RRRAS02
Study2array_ARR
Study2array_RRR
Study2seq_ARR
Study2seq_RRR
Study3_RRr
Study3_RRR
Study4_RRR
Study4_RRRplus
Study5_RRR
Study5_G2
Study5_G3
Study5_G4
1.2
0.8
0.4
0.0
-0.4
Pre-immunization
Day of VAC3
**Day 1 post VAC3
Day 3 post VAC3
Day 14 post VAC3
Day 1 post VAC1
Day 2 post VAC1
Day 6 post VAC1
Day 14 post VAC1
Day of VAC2
Day 1 post VAC2
Day 6 post VAC2
Day 6 post VAC3
Day 21 post VAC3
Day 21 post VAC3
Day 1 post ChAd63 ME-TRAP
Day 1 post MVA ME-TRAP

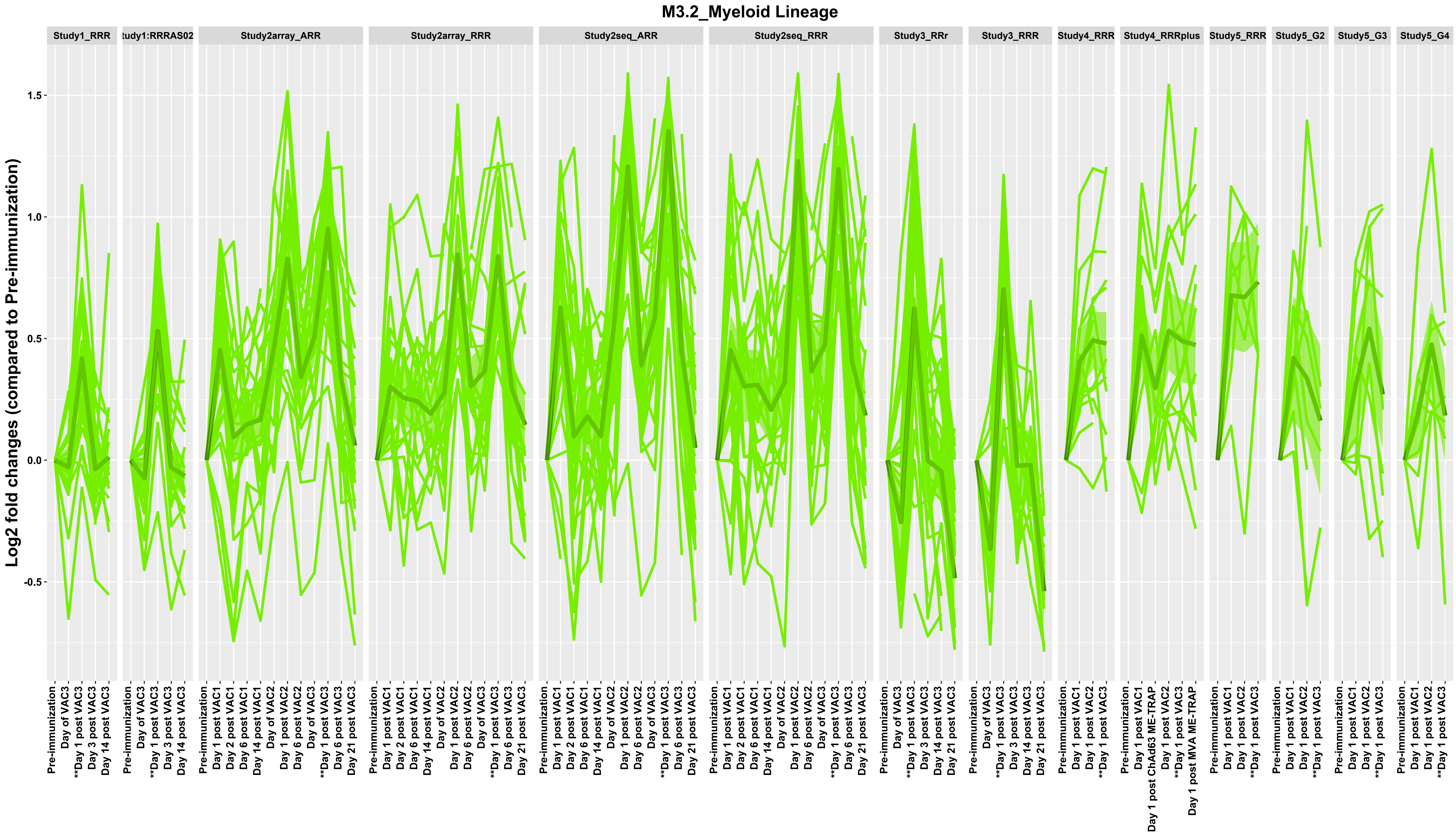
M3.2_Myeloid Lineage
Study1_RRR
tudy1:RRRAS02
Study2array_ARR
Study2array_RRR
Study2seq_ARR
Study2seq_RRR
Study3_RRr
Study3_RRR
Study4_RRR
Study4_RRRplus
Study5_RRR
Study5_G2
Study5_G3
Study5_G4
Log2 fold changes (compared to Pre-immunization)
1.5
1.0
0.5
0.0
-0.5
Pre-immunization
Day of VAC3
**Day 1 post VAC3
Day 3 post VAC3
Day 14 post VAC3
Day 1 post VAC1
Day 2 post VAC1
Day 6 post VAC1
Day 14 post VAC1
Day of VAC2
Day 1 post VAC2
Day 6 post VAC2
Day 6 post VAC3
Day 21 post VAC3
Day 1 post ChAd63 ME-TRAP
Day 1 post MVA ME-TRAP

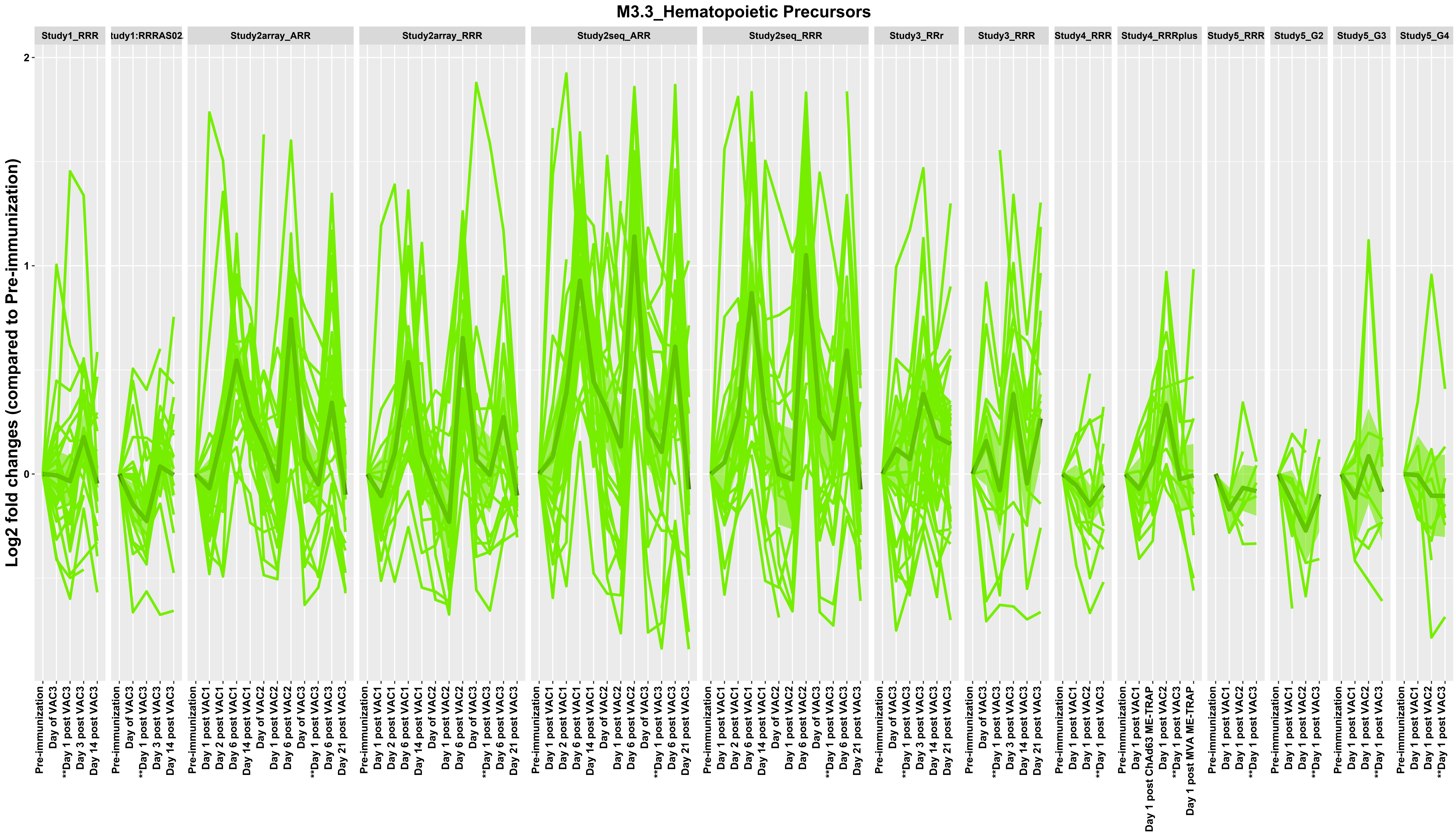
M3.3_Hematopoietic Precursors
Log2 fold changes (compared to Pre-immunization)
Study1_RRR
tudy1:RRRAS02
Study2array_ARR
Study2array_RRR
Study2seq_ARR
Study2seq_RRR
Study3_RRr
Study3_RRR
Study4_RRR
Study4_RRRplus
Study5_RRR
Study5_G2
Study5_G3
Study5_G4
2
1
0
Pre-immunization
Day of VAC3
**Day 1 post VAC3
Day 3 post VAC3
Day 14 post VAC3
Day 1 post VAC1
Day 2 post VAC1
Day 6 post VAC1
Day 14 post VAC1
Day of VAC2
Day 1 post VAC2
Day 6 post VAC2
Day 6 post VAC3
Day 21 post VAC3
Day 21 post VAC1
**Day 1 post VAC2
Day 1 post ChAd63 ME-TRAP
Day 1 post MVA ME-TRAP

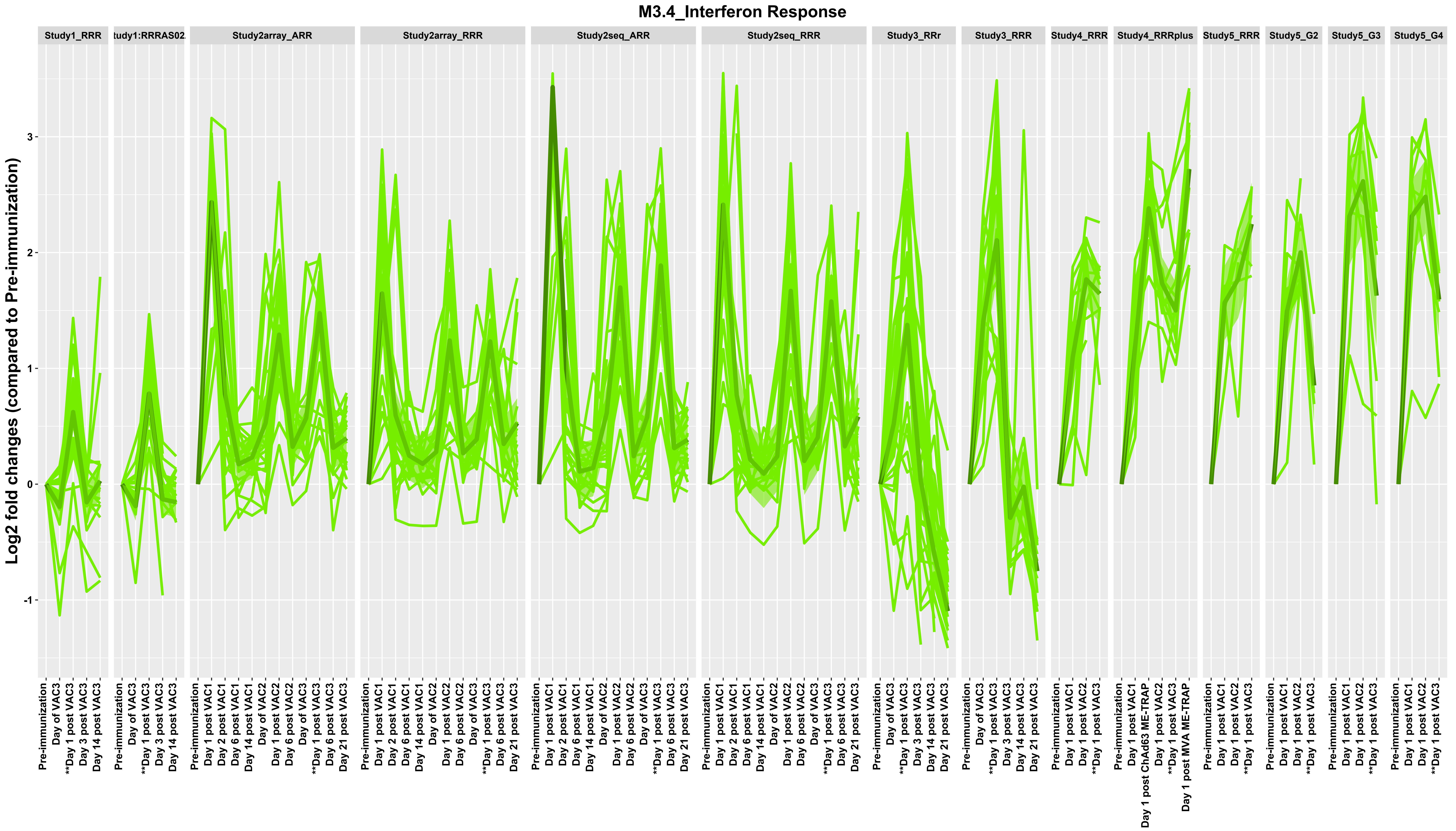
M3.4_Interferon Response
Log2 fold changes (compared to Pre-immunization)
Study1_RRR
tudy1:RRRAS02
Study2array_ARR
Study2array_RRR
Study2seq_ARR
Study2seq_RRR
Study3_RRr
Study3_RRR
Study4_RRR
Study4_RRRplus
Study5_RRR
Study5_G2
Study5_G3
Study5_G4
3
2
1
0
-1
Pre-immunization
Day of VAC3
**Day 1 post VAC3
Day 3 post VAC3
Day 14 post VAC3
Day 1 post VAC1
Day 2 post VAC1
Day 6 post VAC1
Day 14 post VAC1
Day of VAC2
Day 1 post VAC2
Day 6 post VAC2
Day 6 post VAC3
Day 21 post VAC3
Day 1 post ChAd63 ME-TRAP
Day 1 post MVA ME-TRAP
**Day 1 post VAC3

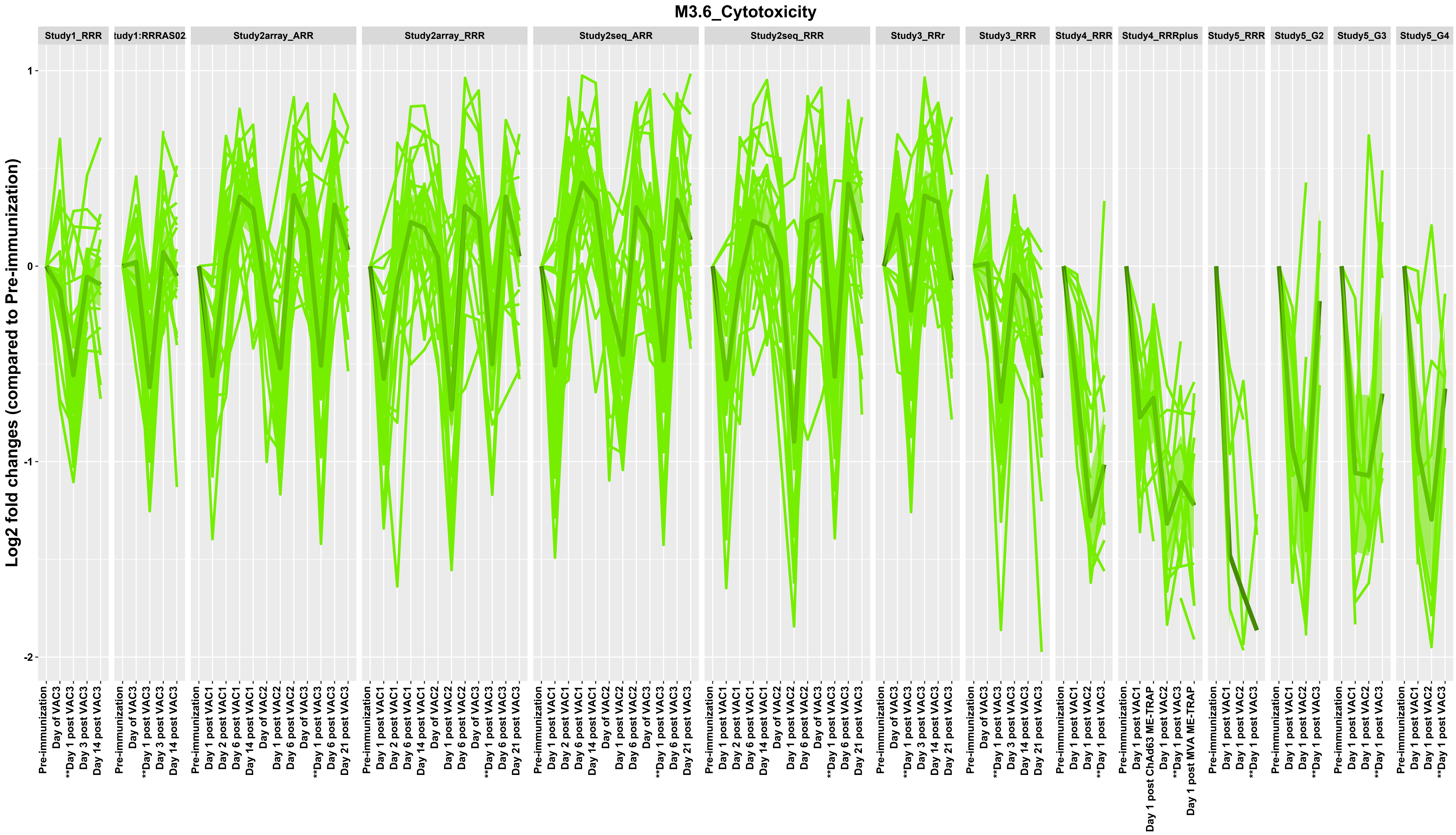

M3.6_Cytotoxicity
Study1_RRR
tudy1:RRRAS02
Study2array_ARR
Study2array_RRR
Study2seq_ARR
Study2seq_RRR
Study3_RRr
Study3_RRR
Study4_RRR
Study4_RRRplus
Study5_RRR
Study5_G2
Study5_G3
Study5_G4
Log2 fold changes (compared to Pre-immunization)
1
0
-1
-2
Pre-immunization
Day of VAC3
**Day 1 post VAC3
Day 3 post VAC3
Day 14 post VAC3
Day 1 post VAC1
Day 2 post VAC1
Day 6 post VAC1
Day 14 post VAC1
Day of VAC2
Day 1 post VAC2
Day 6 post VAC2
Day 6 post VAC3
Day 21 post VAC3
**Day 1 post VAC2
Day 1 post ChAd63 ME-TRAP
Day 1 post MVA ME-TRAP
**Day 1 post VAC1

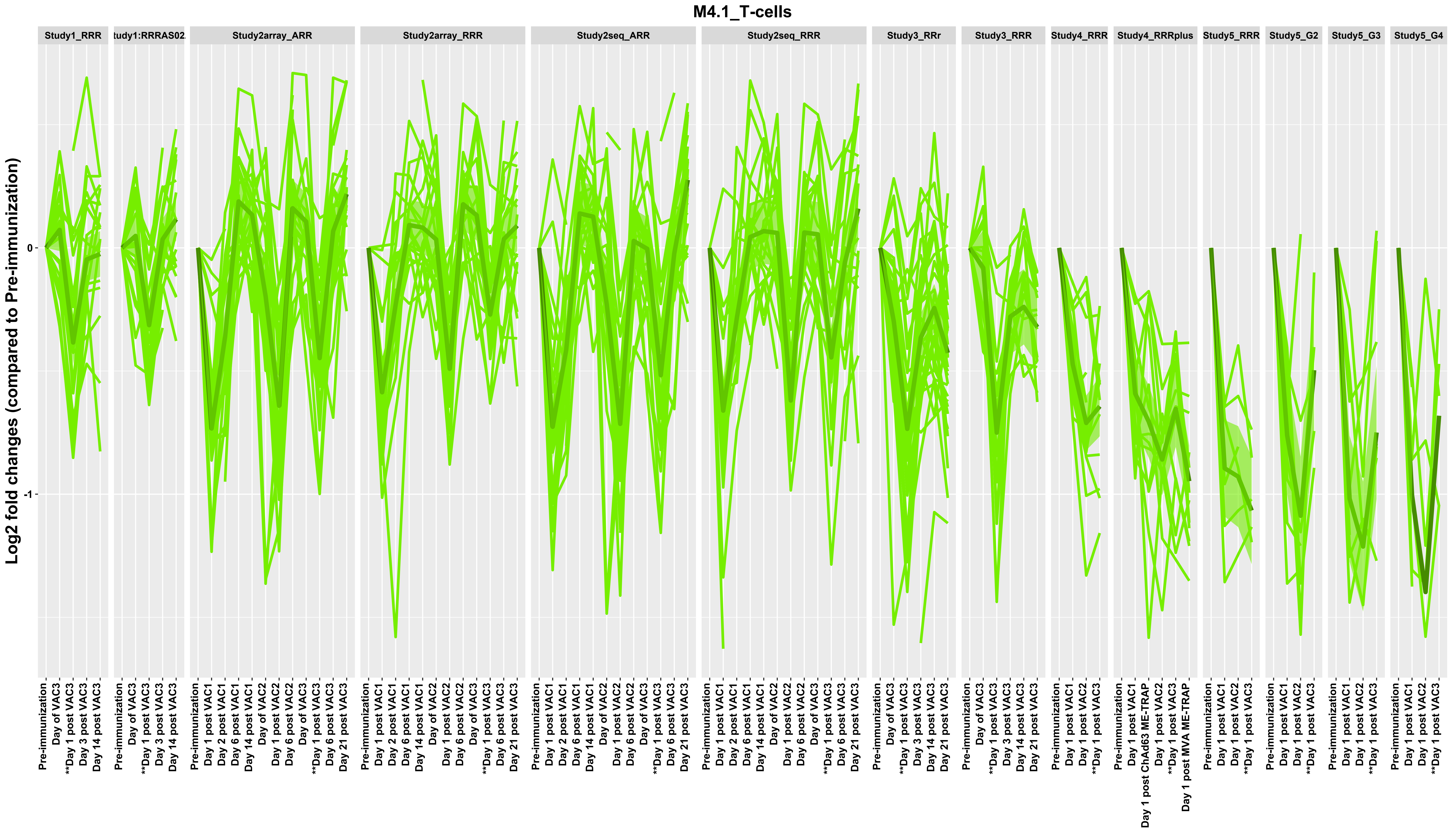
M4.1_T-cells
Log2 fold changes (compared to Pre-immunization)
0
-1
Study1_RRR
tudy1:RRRAS02
Study2array_ARR
Study2array_RRR
Study2seq_ARR
Study2seq_RRR
Study3_RRr
Study3_RRR
Study4_RRR
Study4_RRRplus
Study5_RRR
Study5_G2
Study5_G3
Study5_G4
Pre-immunization
Day of VAC3
**Day 1 post VAC3
Day 3 post VAC3
Day 14 post VAC3
Day 1 post VAC1
Day 2 post VAC1
Day 6 post VAC1
Day 14 post VAC1
Day of VAC2
Day 1 post VAC2
Day 6 post VAC2
Day 6 post VAC3
Day 21 post VAC3
Day 1 post ChAd63 ME-TRAP
Day 1 post MVA ME-TRAP
**Day 1 post VAC3

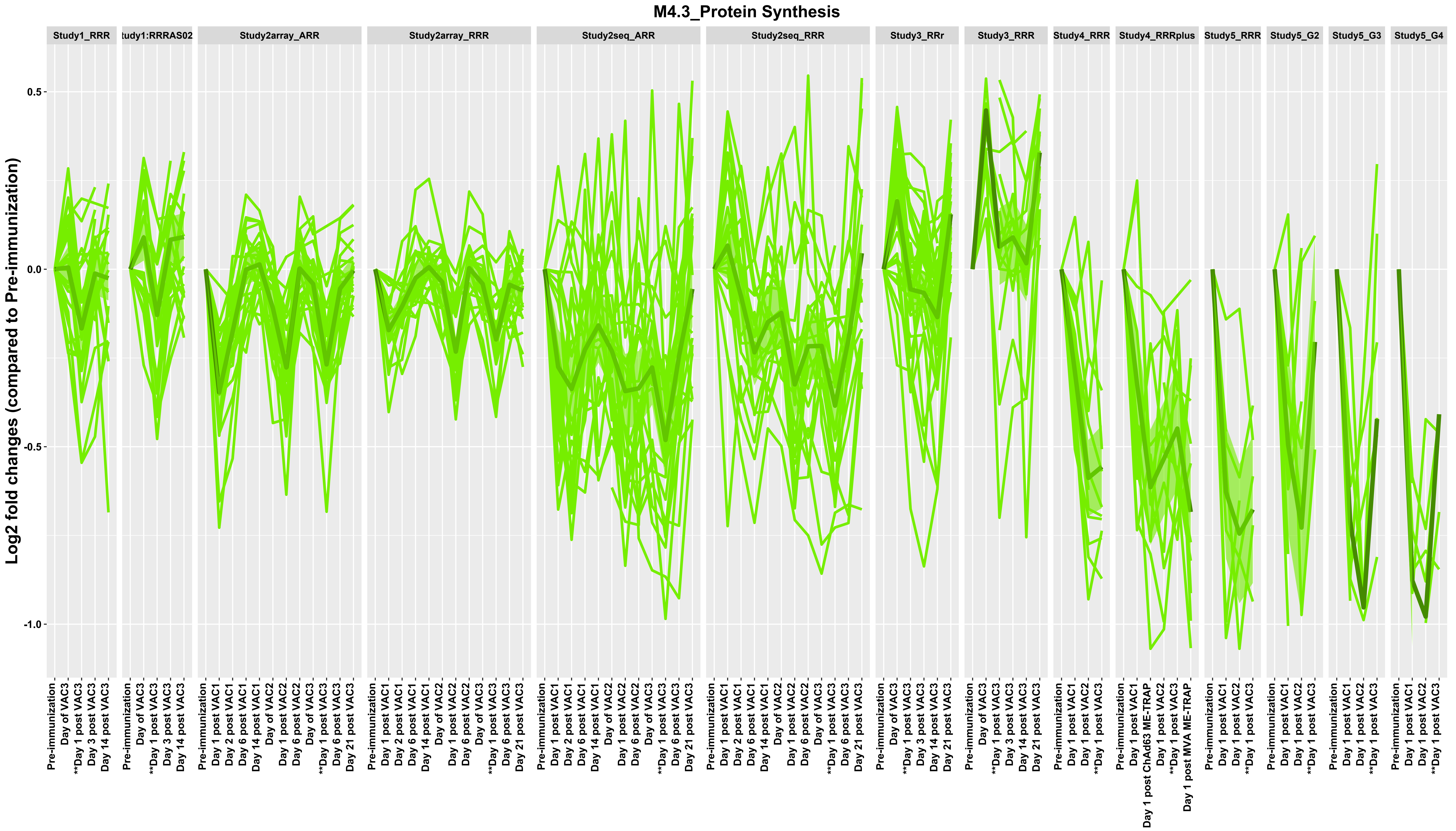

M4.3_Protein Synthesis
Log2 fold changes (compared to Pre-immunization)
0.5
0.0
-0.5
-1.0
Study1_RRR
tudy1:RRRAS02
Study2array_ARR
Study2array_RRR
Study2seq_ARR
Study2seq_RRR
Study3_RRr
Study3_RRR
Study4_RRR
Study4_RRRplus
Study5_RRR
Study5_G2
Study5_G3
Study5_G4
Pre-immunization
Day of VAC3
**Day 1 post VAC3
Day 3 post VAC3
Day 14 post VAC3
Day 1 post VAC1
Day 2 post VAC1
Day 6 post VAC1
Day 14 post VAC1
Day of VAC2
Day 1 post VAC2
Day 6 post VAC2
Day 6 post VAC3
Day 21 post VAC3
Day 1 post ChAd63 ME-TRAP
Day 1 post MVA ME-TRAP
**Day 1 post VAC2

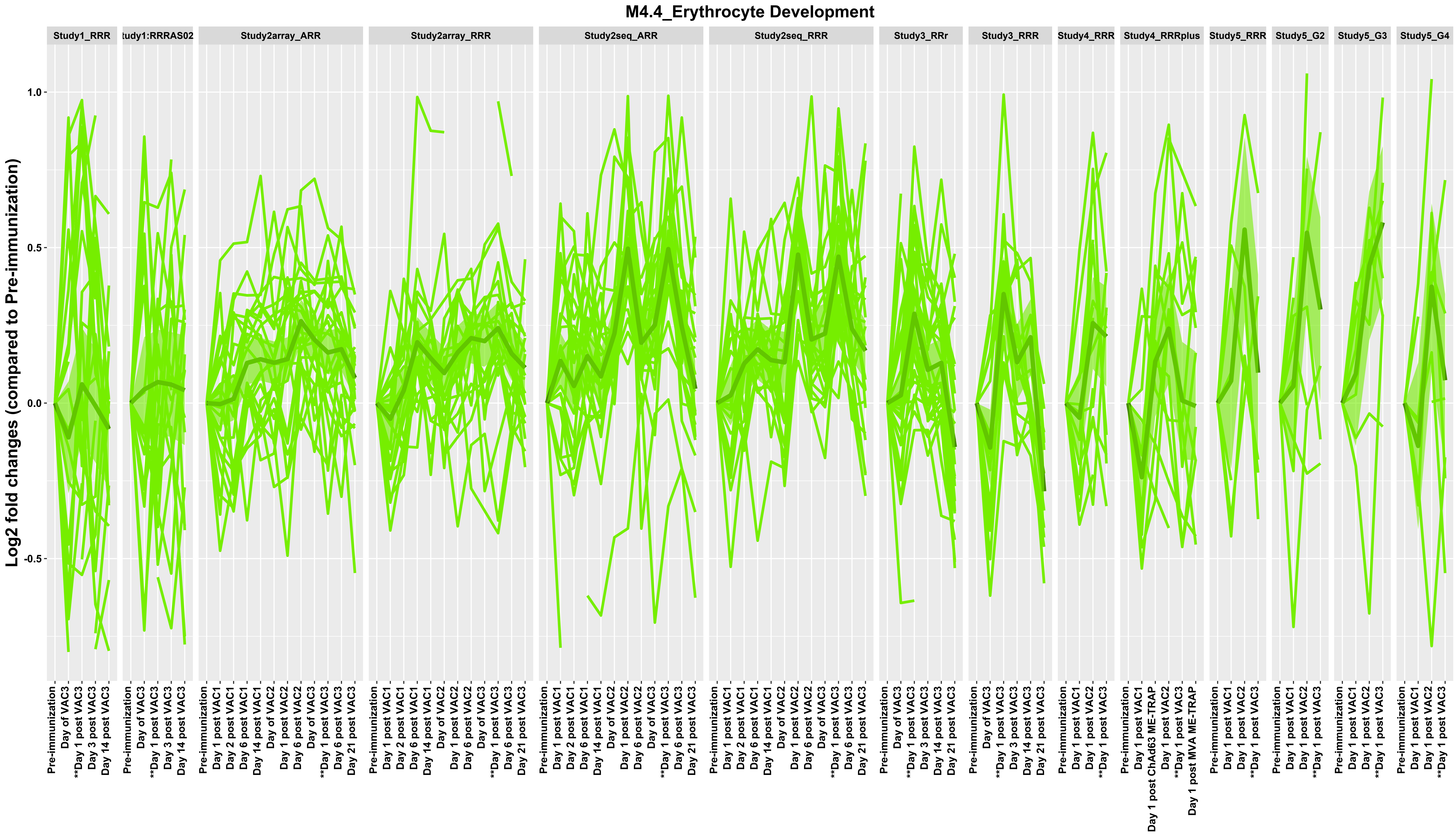
M4.4_Erythrocyte Development
Study1_RRR
tudy1:RRRAS02
Study2array_ARR
Study2array_RRR
Study2seq_ARR
Study2seq_RRR
Study3_RRr
Study3_RRR
Study4_RRR
Study4_RRRplus
Study5_RRR
Study5_G2
Study5_G3
Study5_G4
Log2 fold changes (compared to Pre-immunization)
1.0
0.5
0.0
-0.5
Pre-immunization
Day of VAC3
**Day 1 post VAC3
Day 3 post VAC3
Day 14 post VAC3
Day 1 post VAC1
Day 2 post VAC1
Day 6 post VAC1
Day 14 post VAC1
Day of VAC2
Day 1 post VAC2
Day 6 post VAC2
Day 6 post VAC3
Day 21 post VAC3
Day 1 post ChAd63 ME-TRAP
Day 1 post MVA ME-TRAP

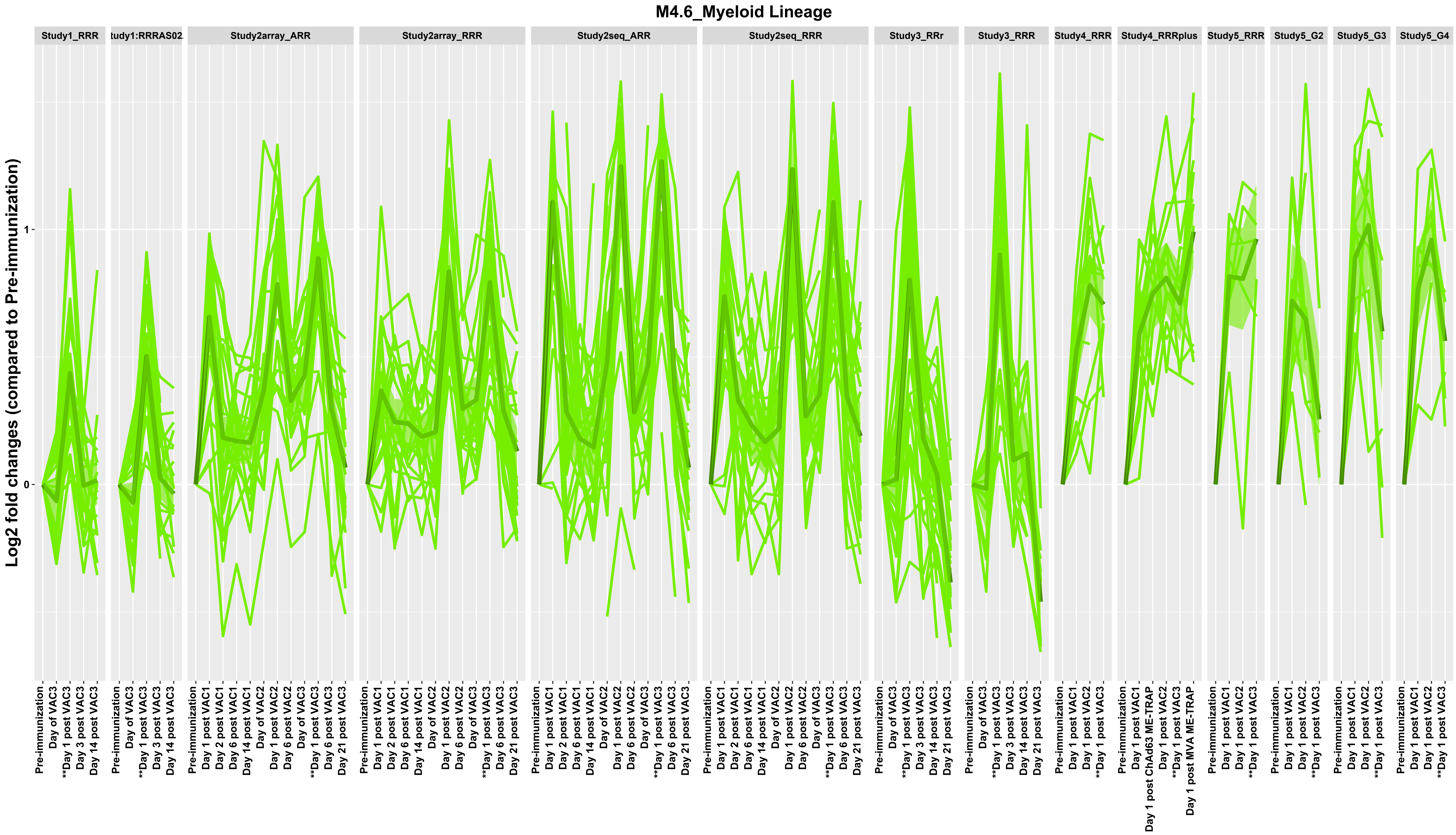

M4.6_Myeloid Lineage
Log2 fold changes (compared to Pre-immunization)
Study1_RRR
tudy1:RRRAS02
Study2array_ARR
Study2array_RRR
Study2seq_ARR
Study2seq_RRR
Study3_RRr
Study3_RRR
Study4_RRR
Study4_RRRplus
Study5_RRR
Study5_G2
Study5_G3
Study5_G4
1
0
Pre-immunization
Day of VAC3
**Day 1 post VAC3
Day 3 post VAC3
Day 14 post VAC3
Day 1 post VAC1
Day 2 post VAC1
Day 6 post VAC1
Day 14 post VAC1
Day of VAC2
Day 1 post VAC2
Day 6 post VAC2
Day 6 post VAC3
Day 21 post VAC3
Day 21 post VAC3
Day 1 post ChAd63 ME-TRAP
Day 1 post MVA ME-TRAP

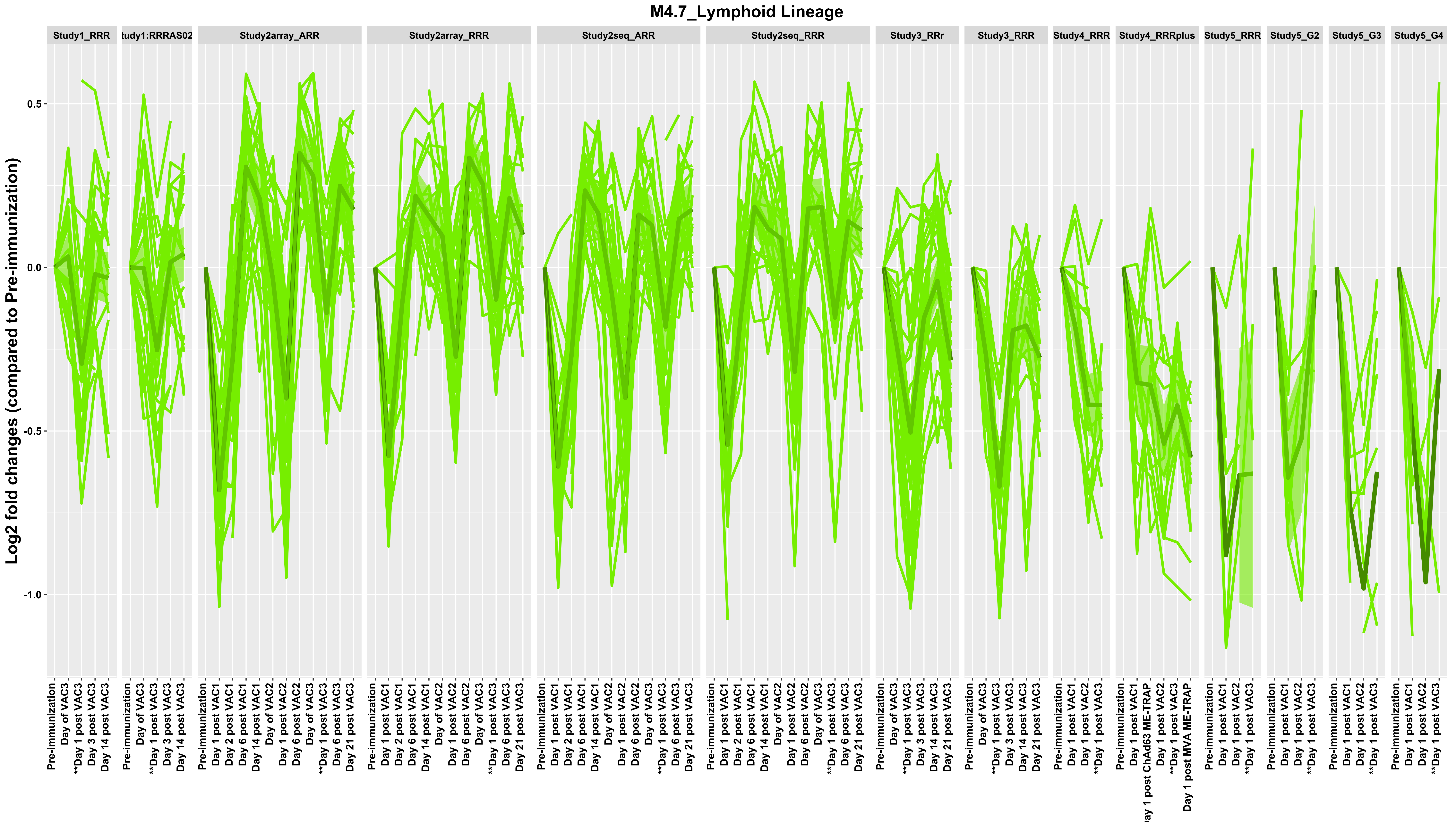
M4.7_Lymphoid Lineage
Log2 fold changes (compared to Pre-immunization)
0.5
0.0
-0.5
-1.0
Study1_RRR
tudy1:RRRAS02
Study2array_ARR
Study2array_RRR
Study2seq_ARR
Study2seq_RRR
Study3_RRr
Study3_RRR
Study4_RRR
Study4_RRRplus
Study5_RRR
Study5_G2
Study5_G3
Study5_G4
Pre-immunization
Day of VAC3
**Day 1 post VAC3
Day 3 post VAC3
Day 14 post VAC3
Day 1 post VAC1
Day 2 post VAC1
Day 6 post VAC1
Day 14 post VAC1
Day of VAC2
Day 1 post VAC2
Day 6 post VAC2
Day 6 post VAC3
Day 21 post VAC3
Day 1 post ChAd63 ME-TRAP
Day 1 post MVA ME-TRAP
**Day 1 post VAC2

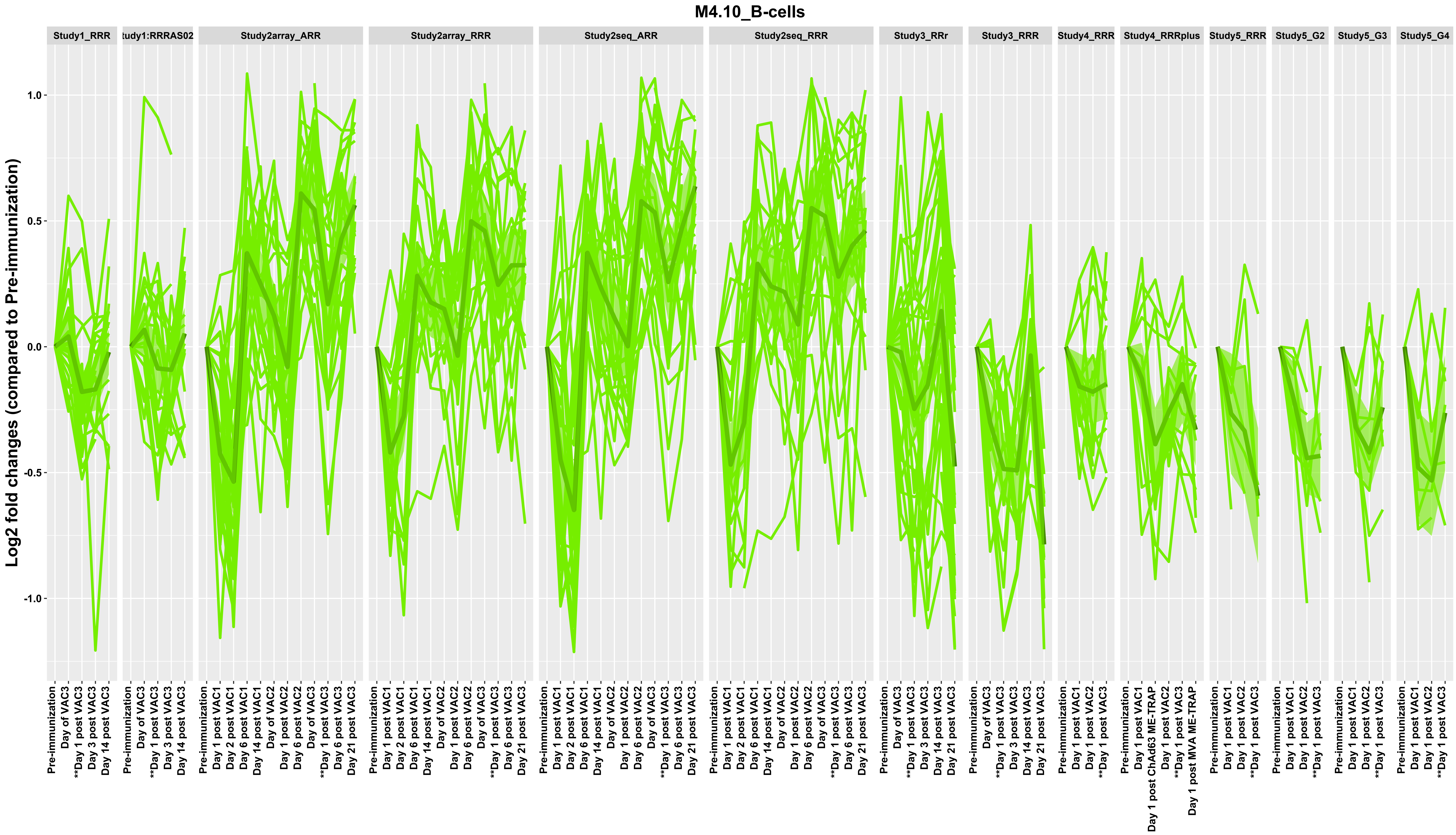

M4.10_B-cells
Study1_RRR
tudy1:RRRAS02
Study2array_ARR
Study2array_RRR
Study2seq_ARR
Study2seq_RRR
Study3_RRr
Study3_RRR
Study4_RRR
Study4_RRRplus
Study5_RRR
Study5_G2
Study5_G3
Study5_G4
Log2 fold changes (compared to Pre-immunization)
1.0
0.5
0.0
-0.5
-1.0
Pre-immunization
Day of VAC3
**Day 1 post VAC3
Day 3 post VAC3
Day 14 post VAC3
Day 1 post VAC1
Day 2 post VAC1
Day 6 post VAC1
Day 14 post VAC1
Day of VAC2
Day 1 post VAC2
Day 6 post VAC2
Day 6 post VAC3
Day 21 post VAC3
Day 1 post ChAd63 ME-TRAP
Day 1 post MVA ME-TRAP

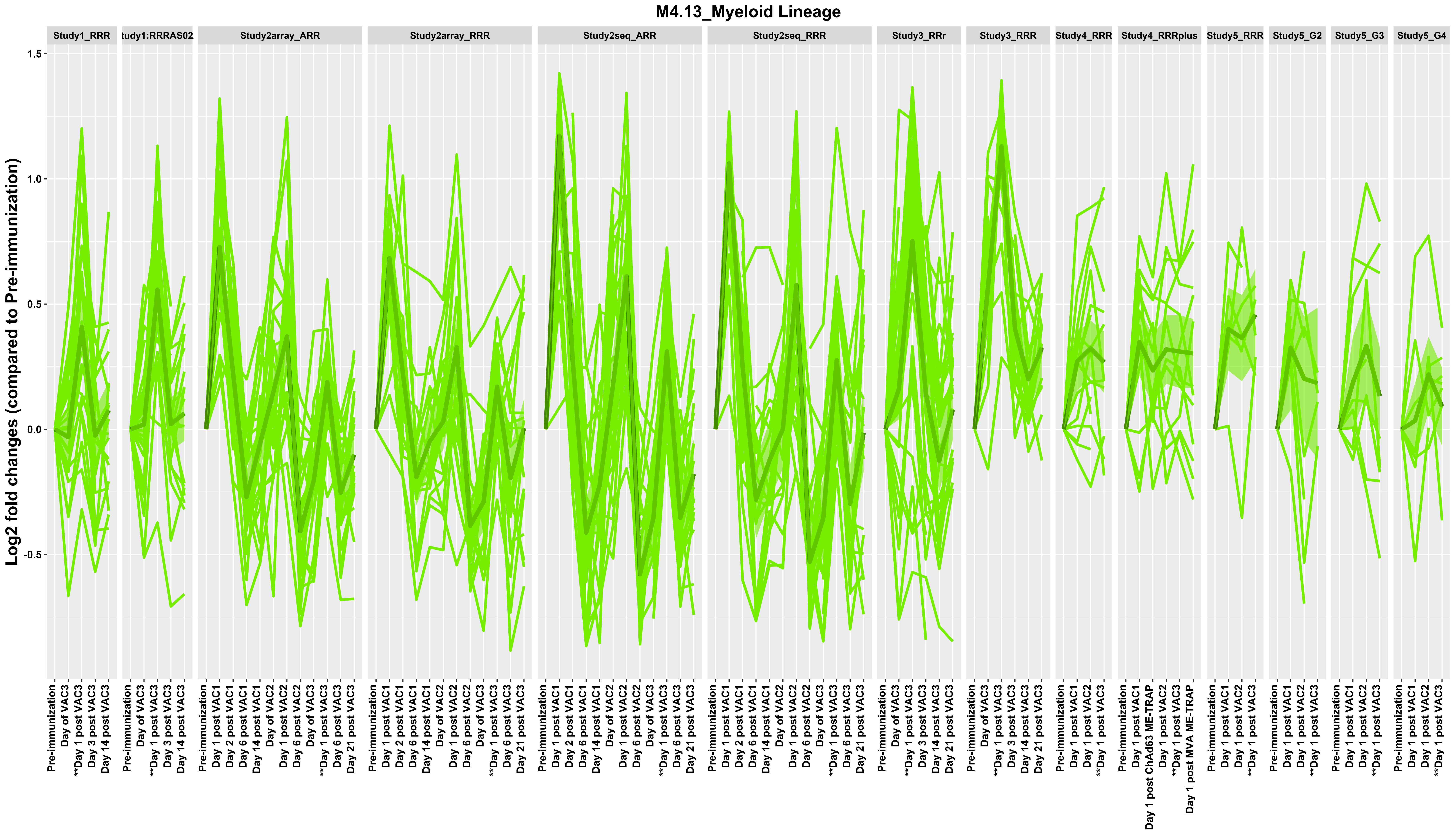
M4.13_Myeloid Lineage
Study1_RRR
tudy1:RRRAS02
Study2array_ARR
Study2array_RRR
Study2seq_ARR
Study2seq_RRR
Study3_RRr
Study3_RRR
Study4_RRR
Study4_RRRplus
Study5_RRR
Study5_G2
Study5_G3
Study5_G4
Log2 fold changes (compared to Pre-immunization)
1.5
1.0
0.5
0.0
-0.5
Pre-immunization
Day of VAC3
**Day 1 post VAC3
Day 3 post VAC3
Day 14 post VAC3
Day 1 post VAC1
Day 2 post VAC1
Day 6 post VAC1
Day 14 post VAC1
Day of VAC2
Day 1 post VAC2
Day 6 post VAC2
Day 6 post VAC3
Day 21 post VAC3
Day 1 post ChAd63 ME-TRAP
Day 1 post MVA ME-TRAP

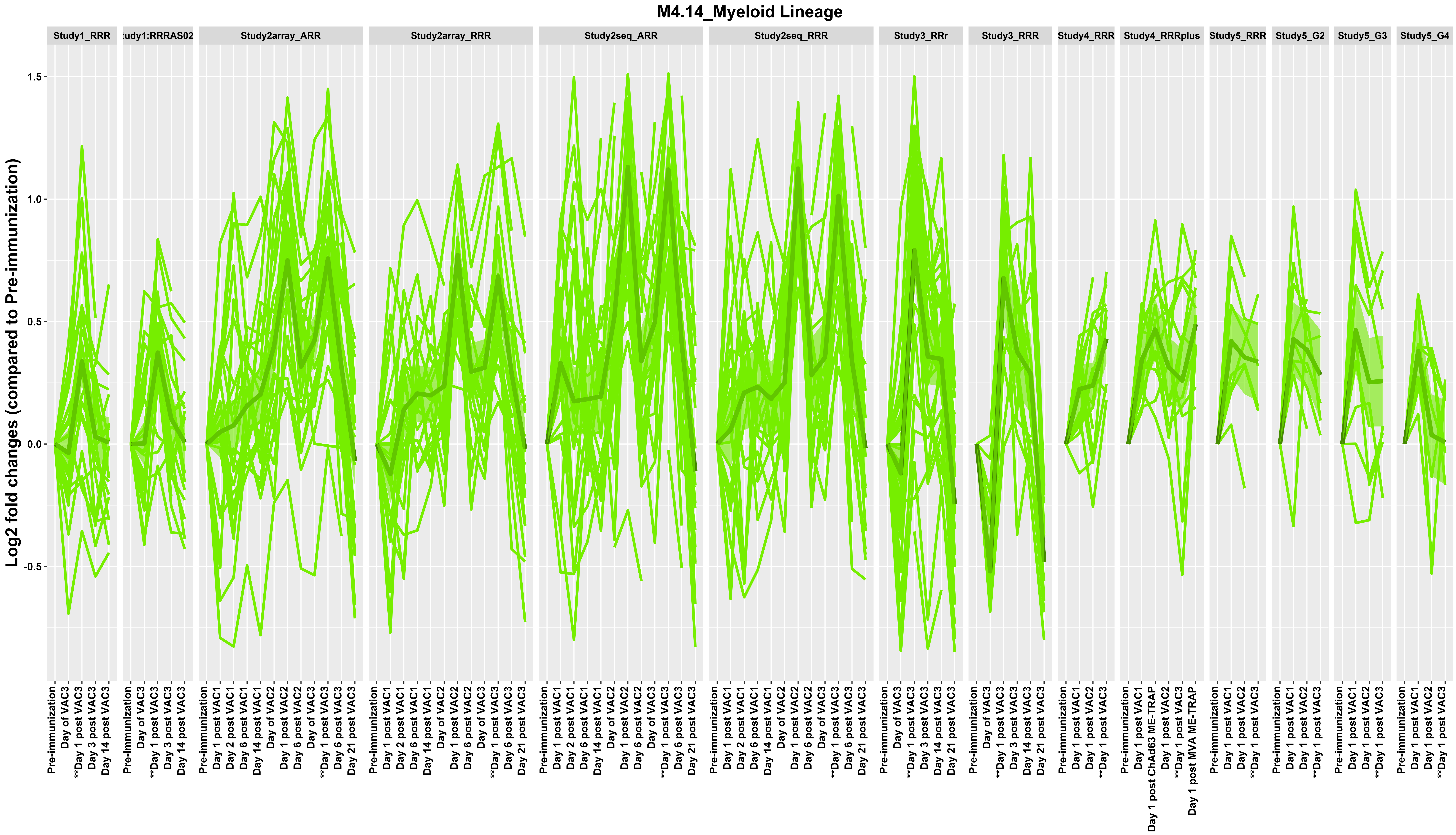

M4.14_Myeloid Lineage
Study1_RRR
tudy1:RRRAS02
Study2array_ARR
Study2array_RRR
Study2seq_ARR
Study2seq_RRR
Study3_RRr
Study3_RRR
Study4_RRR
Study4_RRRplus
Study5_RRR
Study5_G2
Study5_G3
Study5_G4
Log2 fold changes (compared to Pre-immunization)
1.5
1.0
0.5
0.0
-0.5
Pre-immunization
Day of VAC3
**Day 1 post VAC3
Day 3 post VAC3
Day 14 post VAC3
Day 1 post VAC1
Day 2 post VAC1
Day 6 post VAC1
Day 14 post VAC1
Day of VAC2
Day 1 post VAC2
Day 6 post VAC2
Day 6 post VAC3
Day 21 post VAC3
Day 1 post ChAd63 ME-TRAP
Day 1 post MVA ME-TRAP

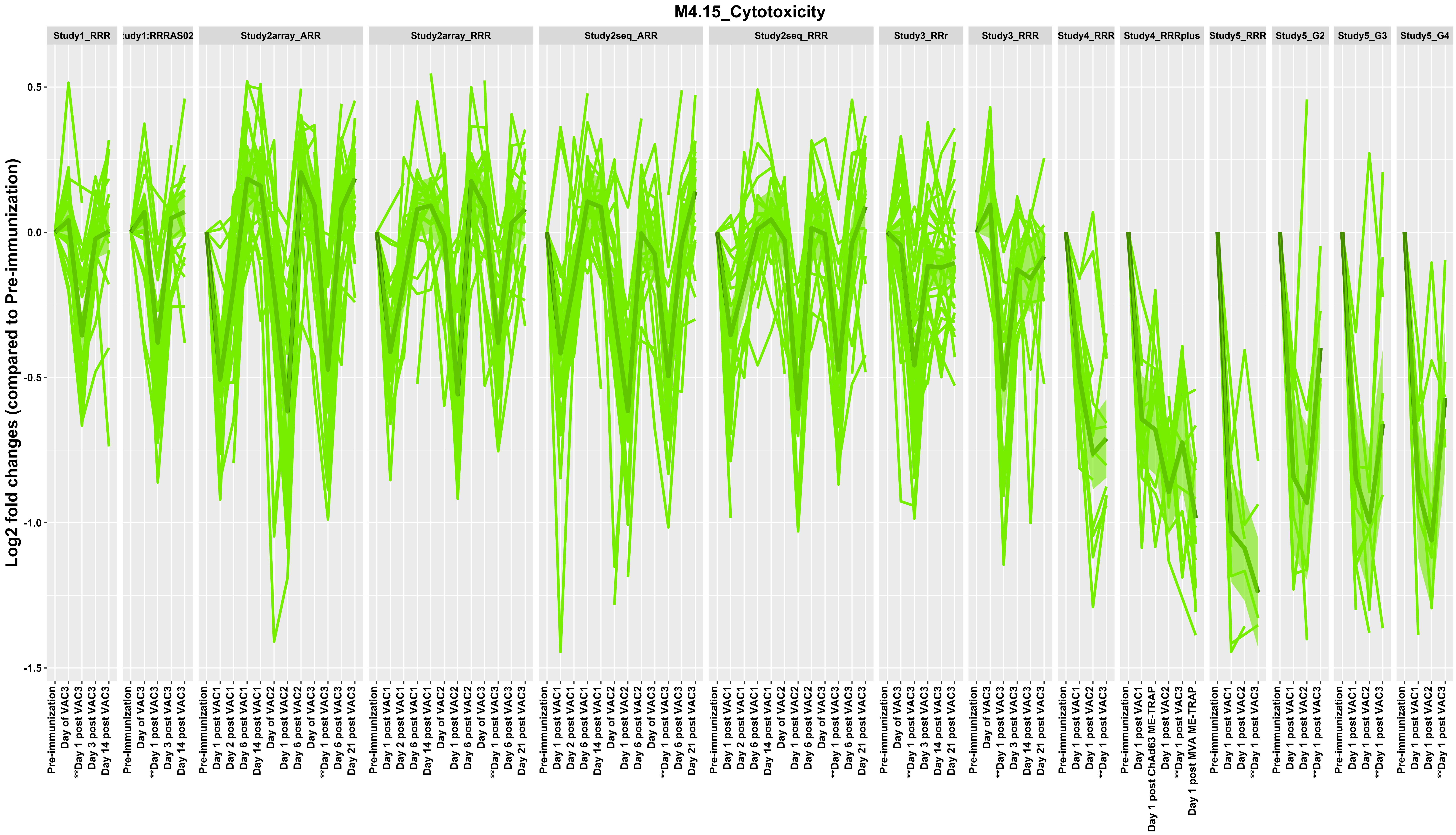
M4.15_Cytotoxicity
Study1_RRR
tudy1:RRRAS02
Study2array_ARR
Study2array_RRR
Study2seq_ARR
Study2seq_RRR
Study3_RRr
Study3_RRR
Study4_RRR
Study4_RRRplus
Study5_RRR
Study5_G2
Study5_G3
Study5_G4
Log2 fold changes (compared to Pre-immunization)
0.5
0.0
-0.5
-1.0
-1.5
Pre-immunization
Day of VAC3
**Day 1 post VAC3
Day 3 post VAC3
Day 14 post VAC3
Day 1 post VAC1
Day 2 post VAC1
Day 6 post VAC1
Day 14 post VAC1
Day of VAC2
Day 1 post VAC2
Day 6 post VAC2
Day 6 post VAC3
Day 21 post VAC3
Day 1 post ChAd63 ME-TRAP
Day 1 post MVA ME-TRAP

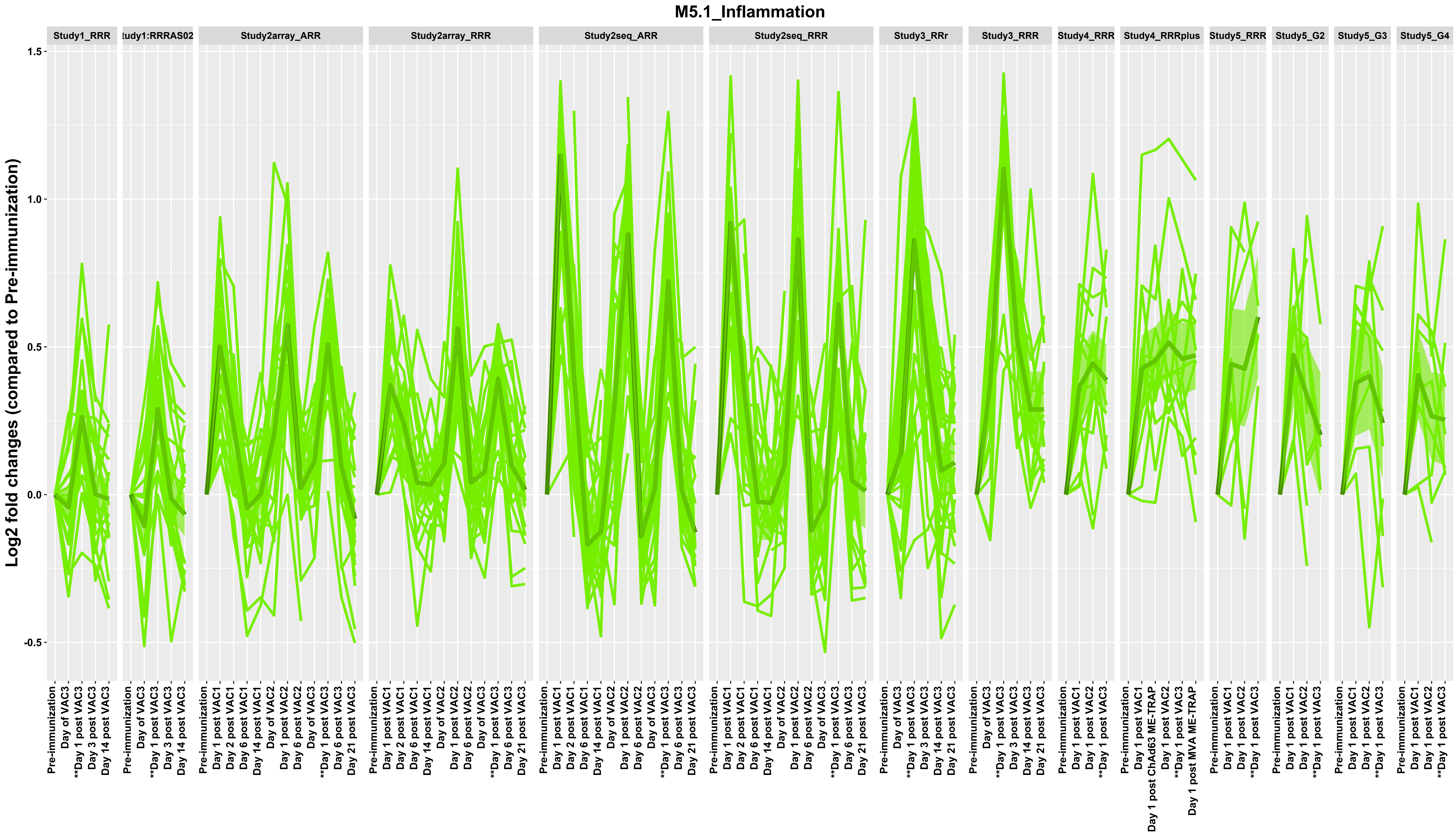
M5.1_Inflammation
Log2 fold changes (compared to Pre-immunization)
1.5
1.0
0.5
0.0
-0.5
Study1_RRR
tudy1:RRRAS02
Study2array_ARR
Study2array_RRR
Study2seq_ARR
Study2seq_RRR
Study3_RRr
Study3_RRR
Study4_RRR
Study4_RRRplus
Study5_RRR
Study5_G2
Study5_G3
Study5_G4
Pre-immunization
Day of VAC3
**Day 1 post VAC3
Day 3 post VAC3
Day 14 post VAC3
Day 1 post VAC1
Day 2 post VAC1
Day 6 post VAC1
Day 14 post VAC1
Day of VAC2
Day 1 post VAC2
Day 6 post VAC2
Day 6 post VAC3
Day 21 post VAC3
Day 21 post VAC3
Day 1 post ChAd63 ME-TRAP
Day 1 post MVA ME-TRAP

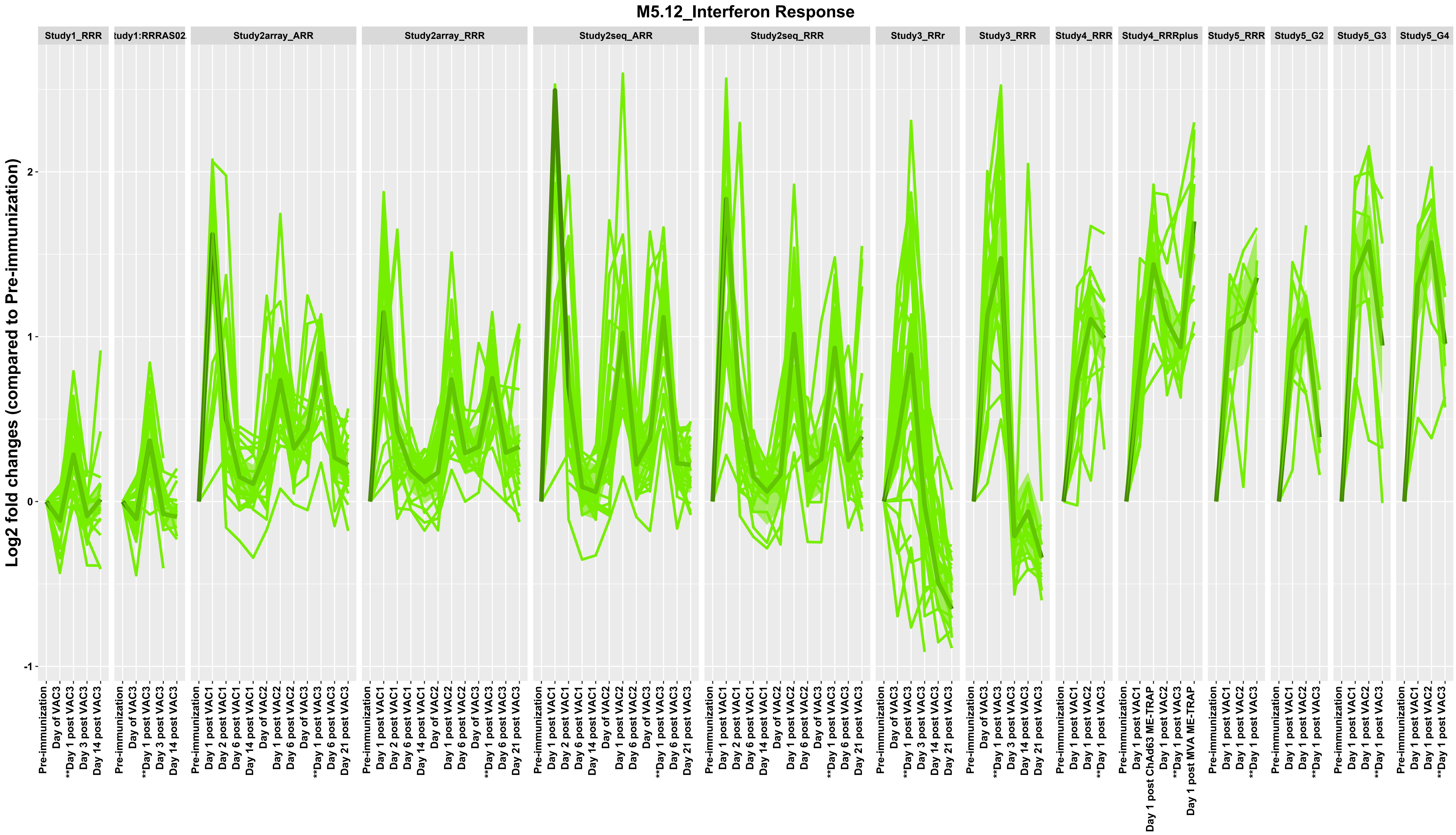
M5.12_Interferon Response
Log2 fold changes (compared to Pre-immunization)
2
1
0
-1
Study1_RRR
tudy1:RRRAS02
Study2array_ARR
Study2array_RRR
Study2seq_ARR
Study2seq_RRR
Study3_RRr
Study3_RRR
Study4_RRR
Study4_RRRplus
Study5_RRR
Study5_G2
Study5_G3
Study5_G4
Pre-immunization
Day of VAC3
**Day 1 post VAC3
Day 3 post VAC3
Day 14 post VAC3
Day 1 post VAC1
Day 2 post VAC1
Day 6 post VAC1
Day 14 post VAC1
Day of VAC2
Day 1 post VAC2
Day 6 post VAC2
Day 6 post VAC3
Day 21 post VAC3
Day 1 post ChAd63 ME-TRAP
Day 1 post MVA ME-TRAP
**Day 1 post VAC3

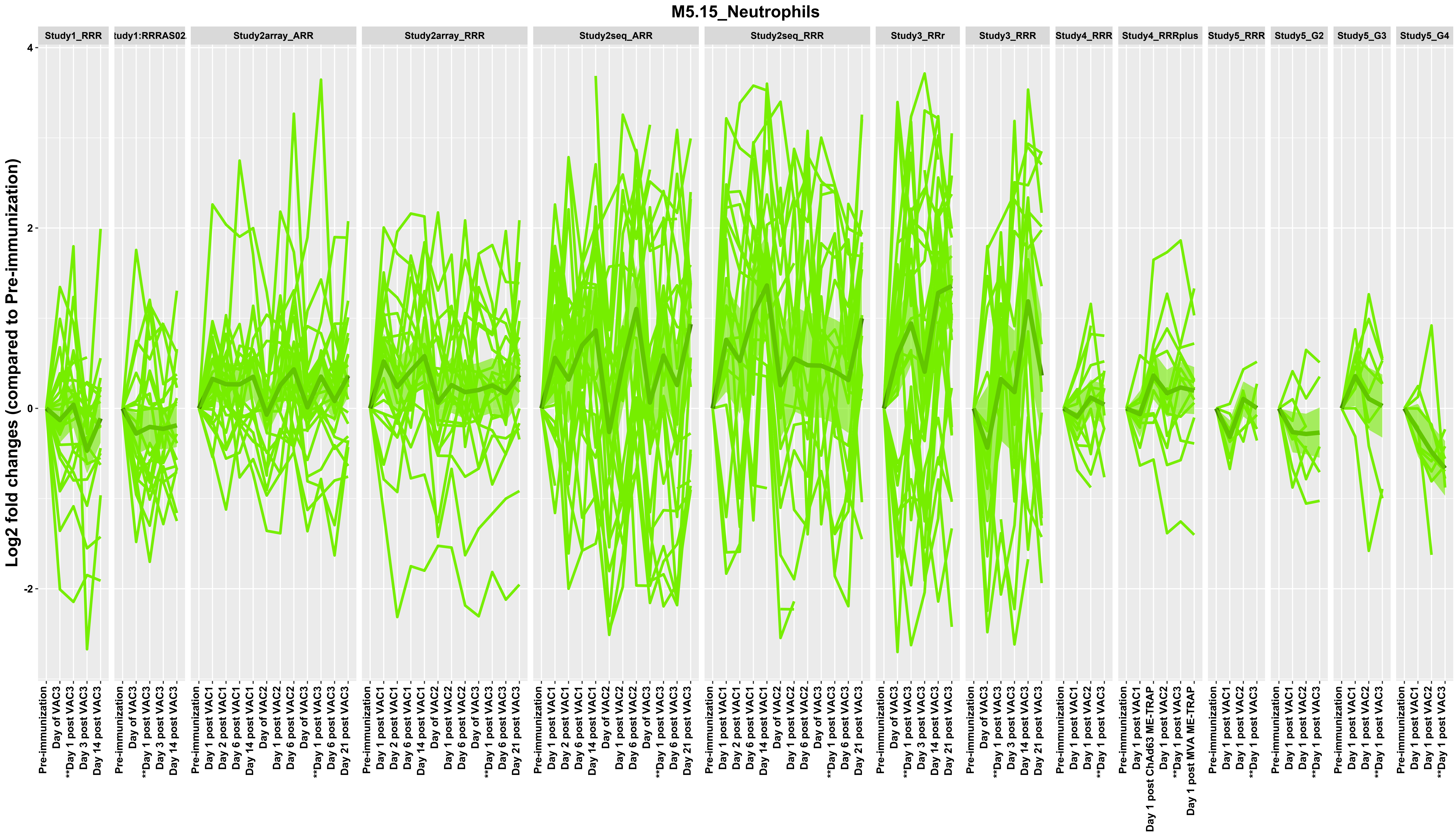

M5.15_Neutrophils
Log2 fold changes (compared to Pre-immunization)
Study1_RRR
tudy1:RRRAS02
Study2array_ARR
Study2array_RRR
Study2seq_ARR
Study2seq_RRR
Study3_RRr
Study3_RRR
Study4_RRR
Study4_RRRplus
Study5_RRR
Study5_G2
Study5_G3
Study5_G4
4
2
0
-2
Pre-immunization
Day of VAC3
**Day 1 post VAC3
Day 3 post VAC3
Day 14 post VAC3
Day 1 post VAC1
Day 2 post VAC1
Day 6 post VAC1
Day 14 post VAC1
Day of VAC2
Day 1 post VAC2
Day 6 post VAC2
Day 6 post VAC3
Day 21 post VAC3
Day 1 post ChAd63 ME-TRAP
Day 1 post MVA ME-TRAP
**Day 1 post VAC3

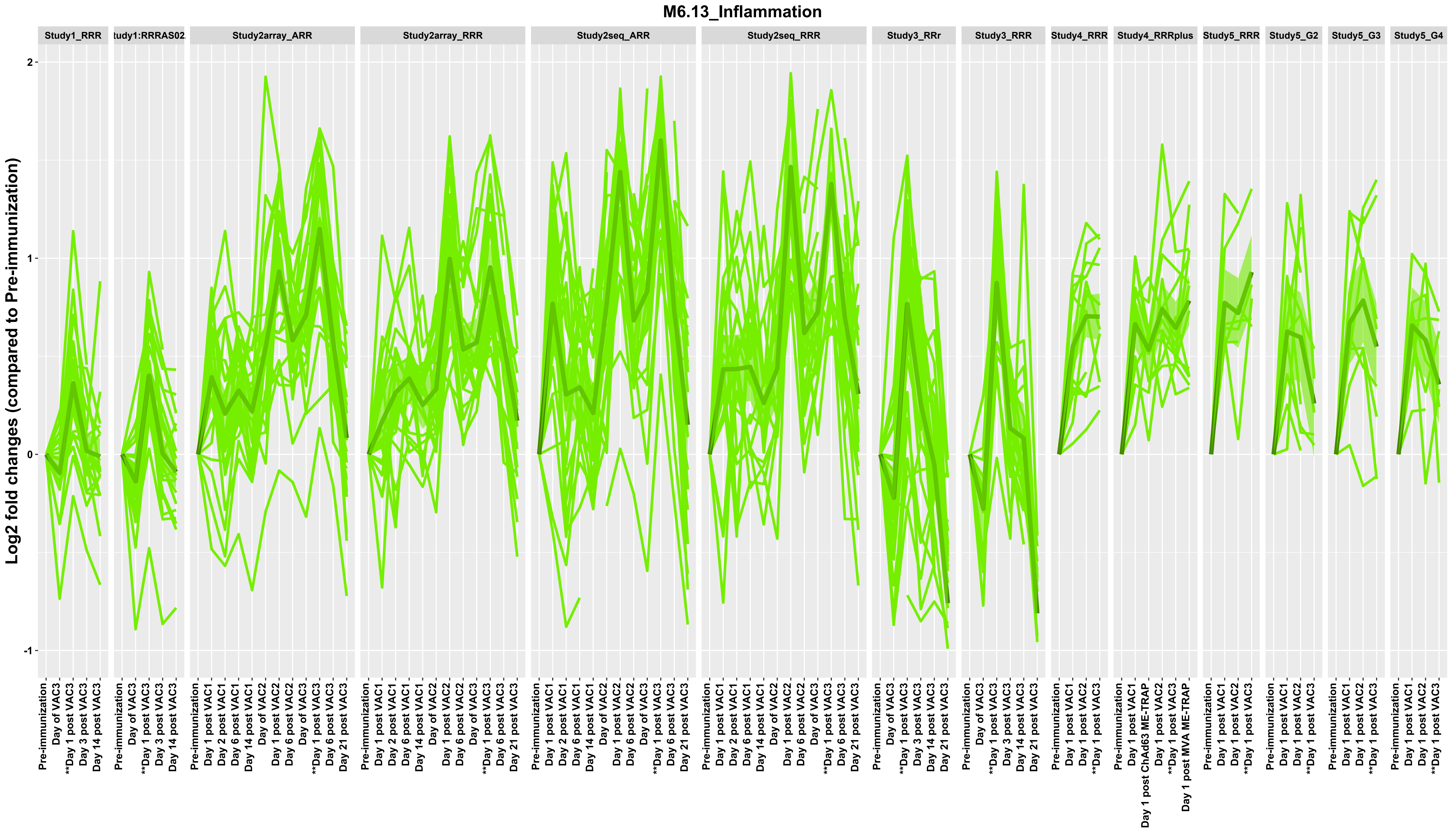
M6.13_Inflammation
Log2 fold changes (compared to Pre-immunization)
Study1_RRR
tudy1:RRRAS02
Study2array_ARR
Study2array_RRR
Study2seq_ARR
Study2seq_RRR
Study3_RRr
Study3_RRR
Study4_RRR
Study4_RRRplus
Study5_RRR
Study5_G2
Study5_G3
Study5_G4
2
1
0
-1
Pre-immunization
Day of VAC3
**Day 1 post VAC3
Day 3 post VAC3
Day 14 post VAC3
Day 1 post VAC1
Day 2 post VAC1
Day 6 post VAC1
Day 14 post VAC1
Day of VAC2
Day 1 post VAC2
Day 6 post VAC2
Day 6 post VAC3
Day 21 post VAC3
Day 21 post VAC3
Day 1 post ChAd63 ME-TRAP
Day 1 post MVA ME-TRAP
**Day 1 post VAC2

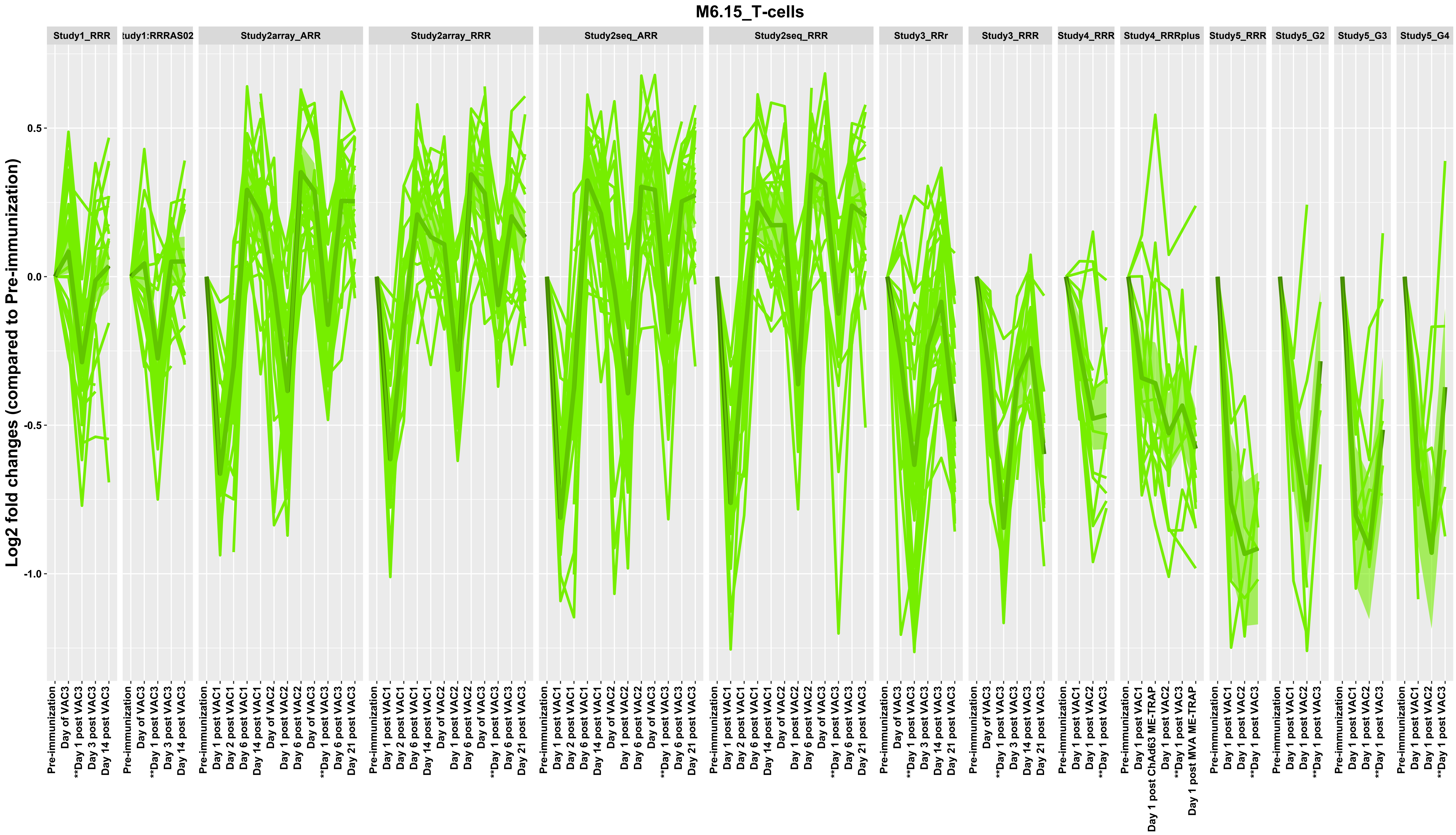
M6.15_T-cells
Study1_RRR
tudy1:RRRAS02
Study2array_ARR
Study2array_RRR
Study2seq_ARR
Study2seq_RRR
Study3_RRr
Study3_RRR
Study4_RRR
Study4_RRRplus
Study5_RRR
Study5_G2
Study5_G3
Study5_G4
Log2 fold changes (compared to Pre-immunization)
0.5
0.0
-0.5
-1.0
Pre-immunization
Day of VAC3
**Day 1 post VAC3
Day 3 post VAC3
Day 14 post VAC3
Day 1 post VAC1
Day 2 post VAC1
Day 6 post VAC1
Day 14 post VAC1
Day of VAC2
Day 1 post VAC2
Day 6 post VAC2
Day 6 post VAC3
Day 21 post VAC3
Day 1 post ChAd63 ME-TRAP
Day 1 post MVA ME-TRAP

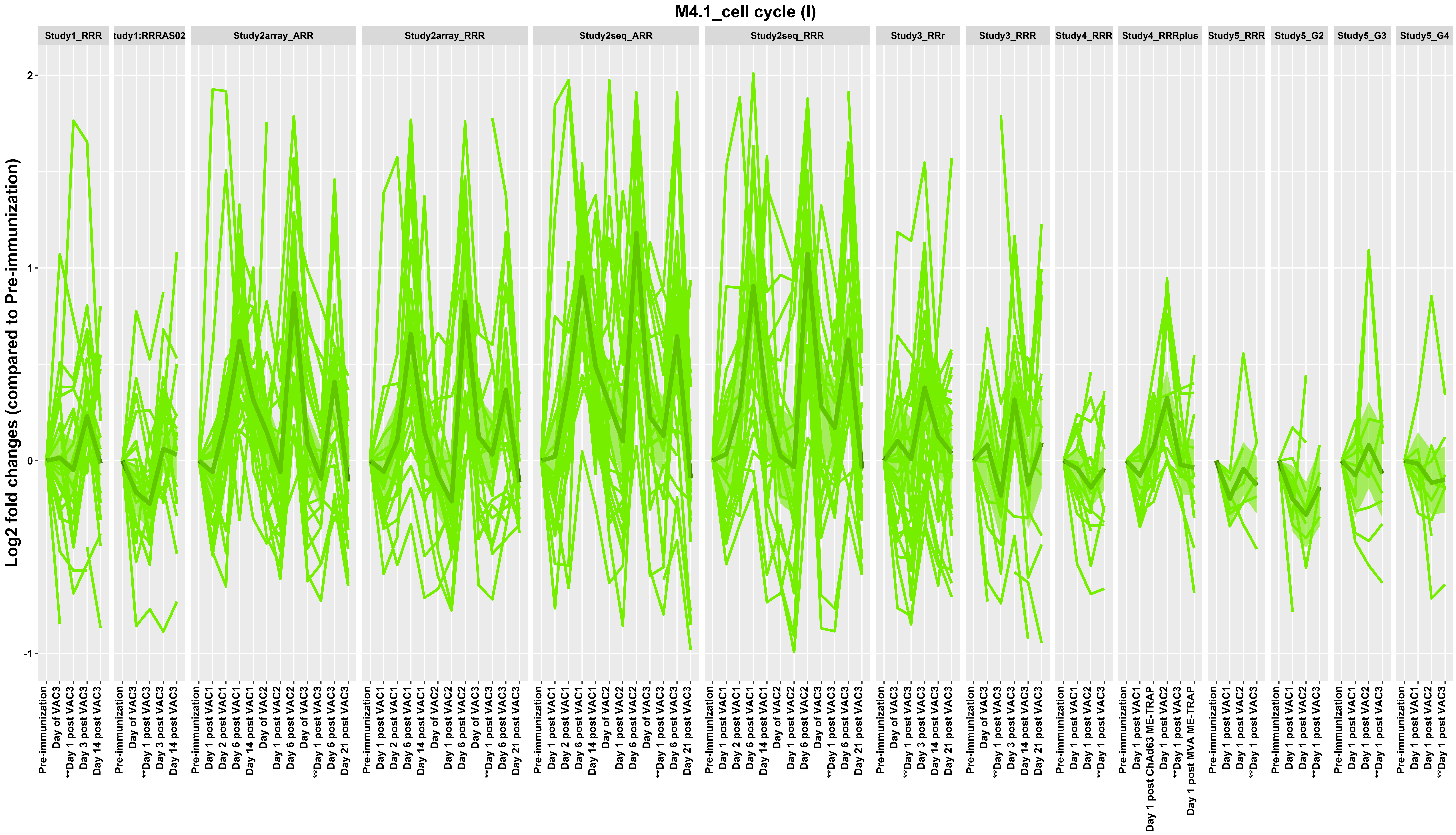
M4.1_cell cycle (I)
Study1_RRR
tudy1:RRRAS02
Study2array_ARR
Study2array_RRR
Study2seq_ARR
Study2seq_RRR
Study3_RRr
Study3_RRR
Study4_RRR
Study4_RRRplus
Study5_RRR
Study5_G2
Study5_G3
Study5_G4
Log2 fold changes (compared to Pre-immunization)
2
1
0
-1
Pre-immunization
Day of VAC3
**Day 1 post VAC3
Day 3 post VAC3
Day 14 post VAC3
Day 1 post VAC1
Day 2 post VAC1
Day 6 post VAC1
Day 14 post VAC1
Day of VAC2
Day 1 post VAC2
Day 6 post VAC2
Day 6 post VAC3
Day 21 post VAC3
Day 2 post VAC3
Day 1 post ChAd63 ME-TRAP
Day 1 post MVA ME-TRAP
**Day 1 post VAC2

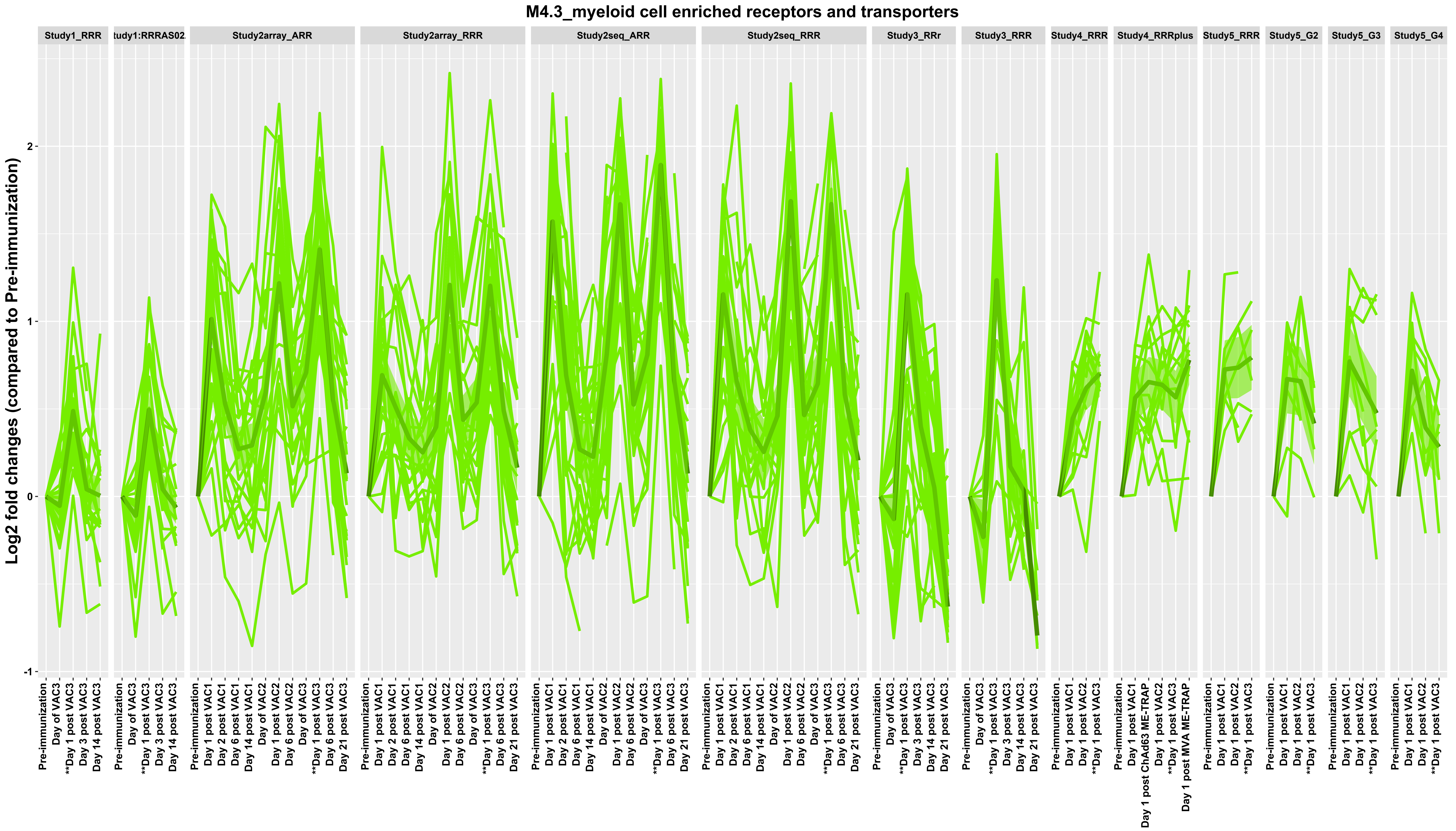
M4.3_myeloid cell enriched receptors and transporters
Log2 fold changes (compared to Pre-immunization)
Study1_RRR
Study2array_ARR
Study2array_RRR
Study2seq_ARR
Study2seq_RRR
Study3_RRr
Study3_RRR
Study4_RRR
Study4_RRRplus
Study5_RRR
Study5_G2
Study5_G3
Study5_G4
2
1
0
-1
Pre-immunization
Day of VAC3
**Day 1 post VAC3
Day 3 post VAC3
Day 14 post VAC3
Day 1 post VAC1
Day 2 post VAC1
Day 6 post VAC1
Day 14 post VAC1
Day of VAC2
Day 1 post VAC2
Day 6 post VAC2
Day 6 post VAC3
Day 21 post VAC3
Day 1 post ChAd63 ME-TRAP
Day 1 post MVA ME-TRAP

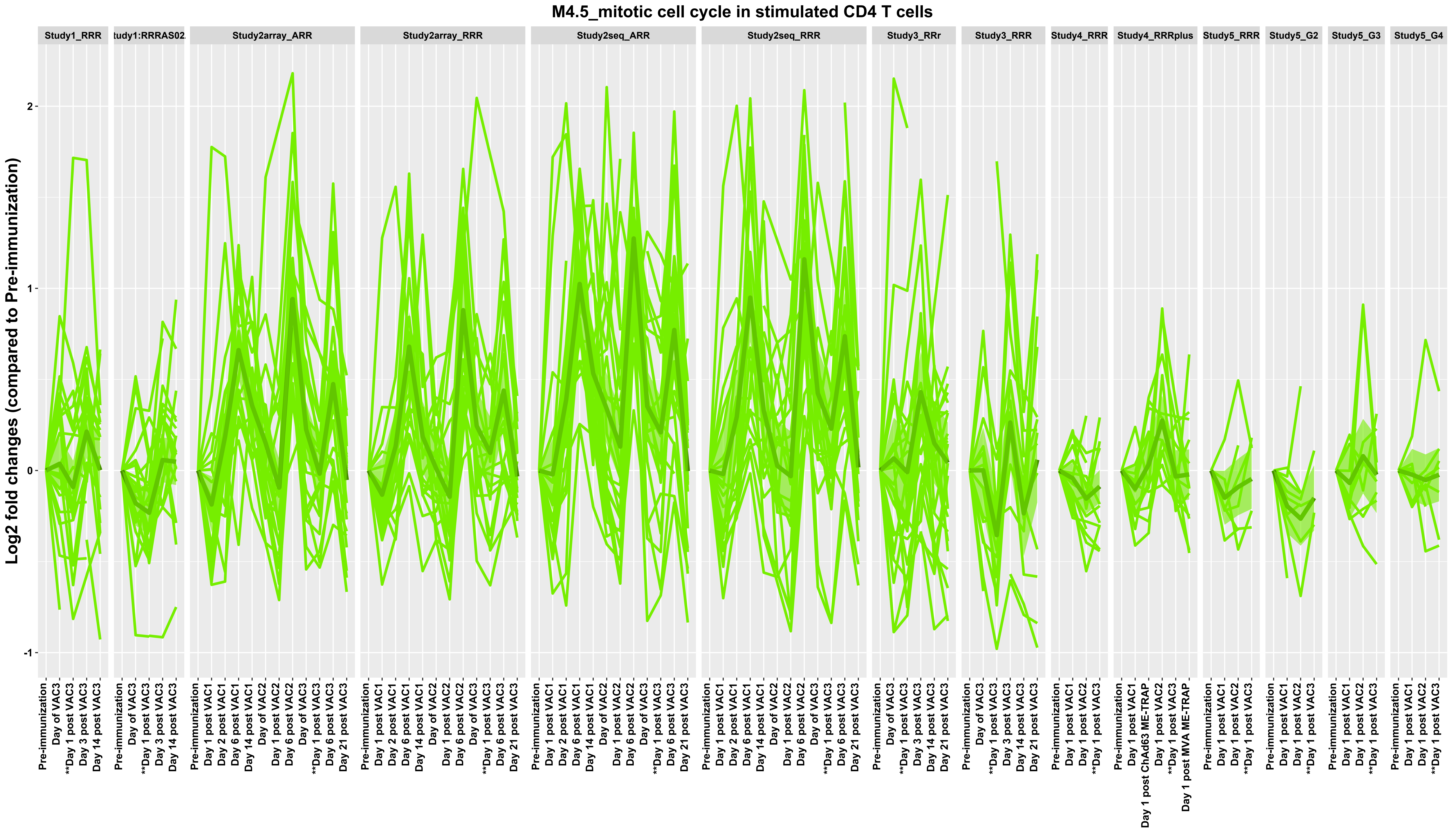
M4.5_mitotic cell cycle in stimulated CD4 T cells
Log2 fold changes (compared to Pre-immunization)
Study1_RRR
tudy1:RRRAS02
Study2array_ARR
Study2array_RRR
Study2seq_ARR
Study2seq_RRR
Study3_RRr
Study3_RRR
Study4_RRR
Study4_RRRplus
Study5_RRR
Study5_G2
Study5_G3
Study5_G4
Pre-immunization
Day of VAC3
**Day 1 post VAC3
Day 3 post VAC3
Day 14 post VAC3
Day 1 post VAC1
Day 2 post VAC1
Day 6 post VAC1
Day 14 post VAC1
Day of VAC2
Day 1 post VAC2
Day 6 post VAC2
Day 6 post VAC3
Day 21 post VAC3
Day 1 post ChAd63 ME-TRAP
**Day 1 post VAC2
Day 1 post MVA ME-TRAP

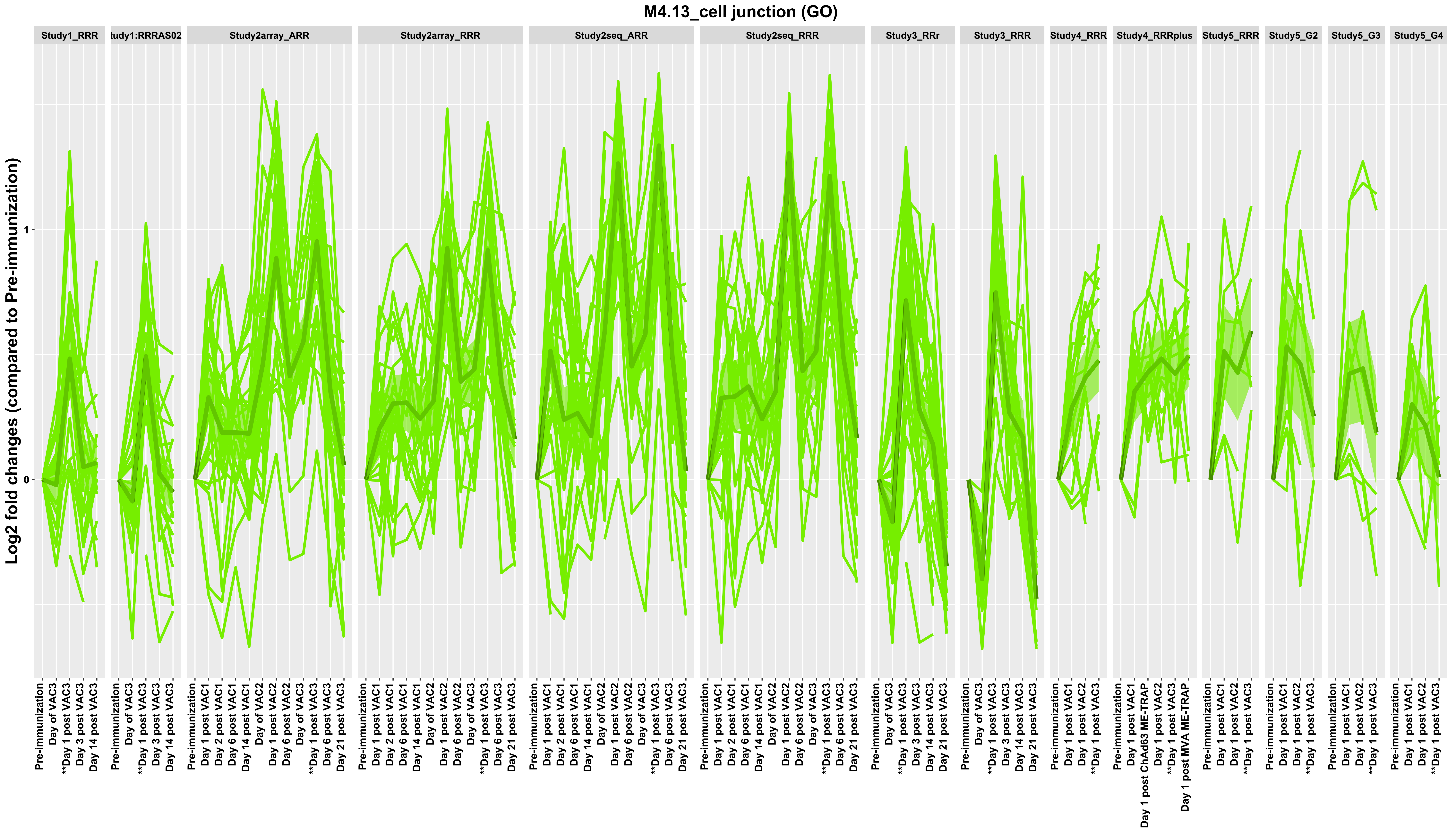

M4.13_cell junction (GO)
Study1_RRR
tudy1:RRRAS02
Study2array_ARR
Study2array_RRR
Study2seq_ARR
Study2seq_RRR
Study3_RRr
Study3_RRR
Study4_RRR
Study4_RRRplus
Study5_RRR
Study5_G2
Study5_G3
Study5_G4
Log2 fold changes (compared to Pre-immunization)
1
0
Pre-immunization
Day of VAC3
**Day 1 post VAC3
Day 3 post VAC3
Day 14 post VAC3
Day 1 post VAC1
Day 2 post VAC1
Day 6 post VAC1
Day 14 post VAC1
Day of VAC2
Day 1 post VAC2
Day 6 post VAC2
Day 6 post VAC3
Day 21 post VAC3
Day 1 post VAC3
Day 21 post VAC3
Day 1 post ChAd63 ME-TRAP
Day 1 post MVA ME-TRAP
**Day 1 post VAC3

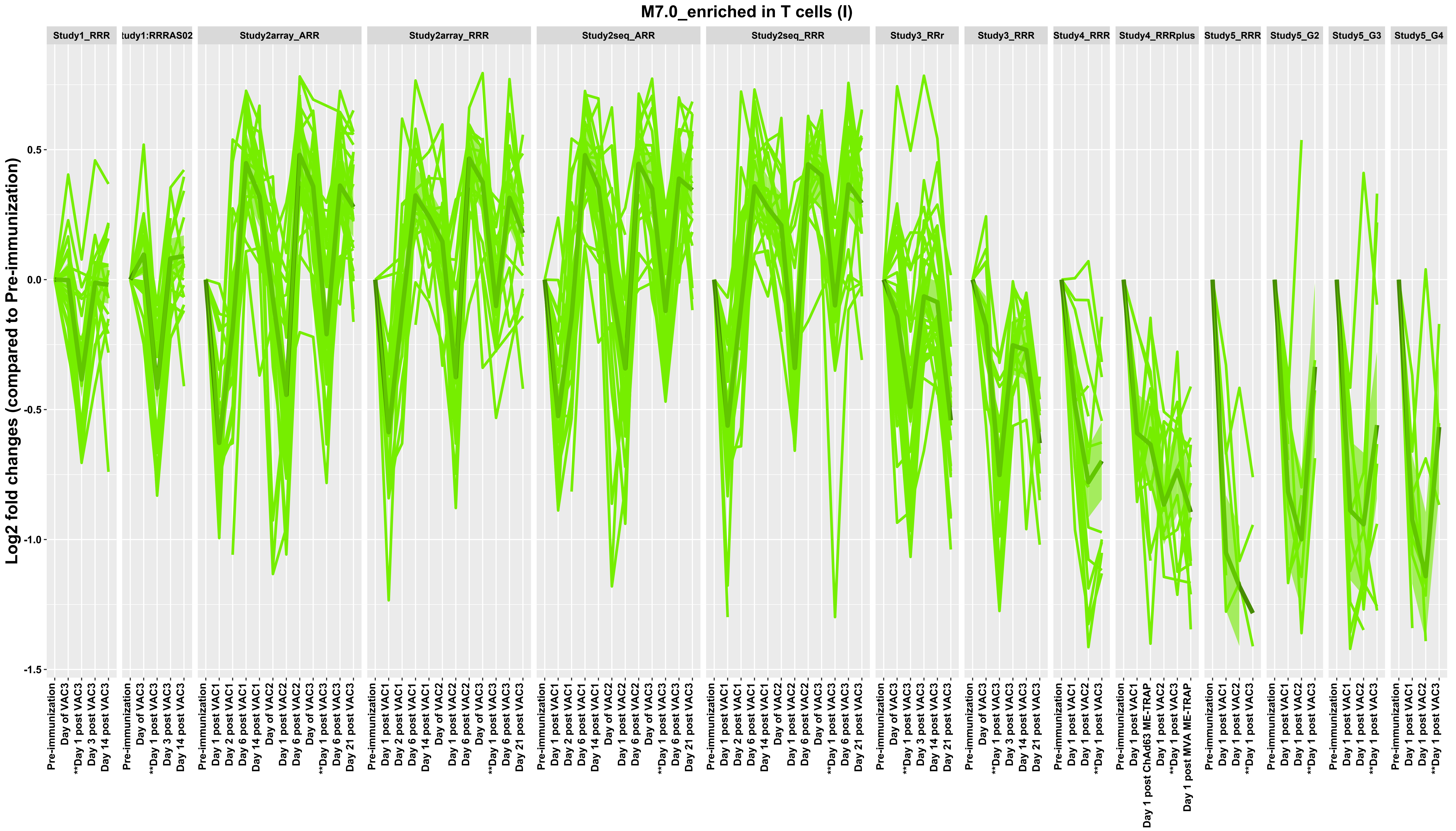
M7.0_enriched in T cells (I)
Study1_RRR
tudy1:RRRAS02
Study2array_ARR
Study2array_RRR
Study2seq_ARR
Study2seq_RRR
Study3_RRr
Study3_RRR
Study4_RRR
Study4_RRRplus
Study5_RRR
Study5_G2
Study5_G3
Study5_G4
Log2 fold changes (compared to Pre-immunization)
0.5
0.0
-0.5
-1.0
-1.5
Pre-immunization
Day of VAC3
**Day 1 post VAC3
Day 3 post VAC3
Day 14 post VAC3
Day 1 post VAC1
Day 2 post VAC1
Day 6 post VAC1
Day 14 post VAC1
Day of VAC2
Day 1 post VAC2
Day 6 post VAC2
Day 6 post VAC3
Day 21 post VAC3
Day 1 post ChAd63 ME-TRAP
Day 1 post MVA ME-TRAP

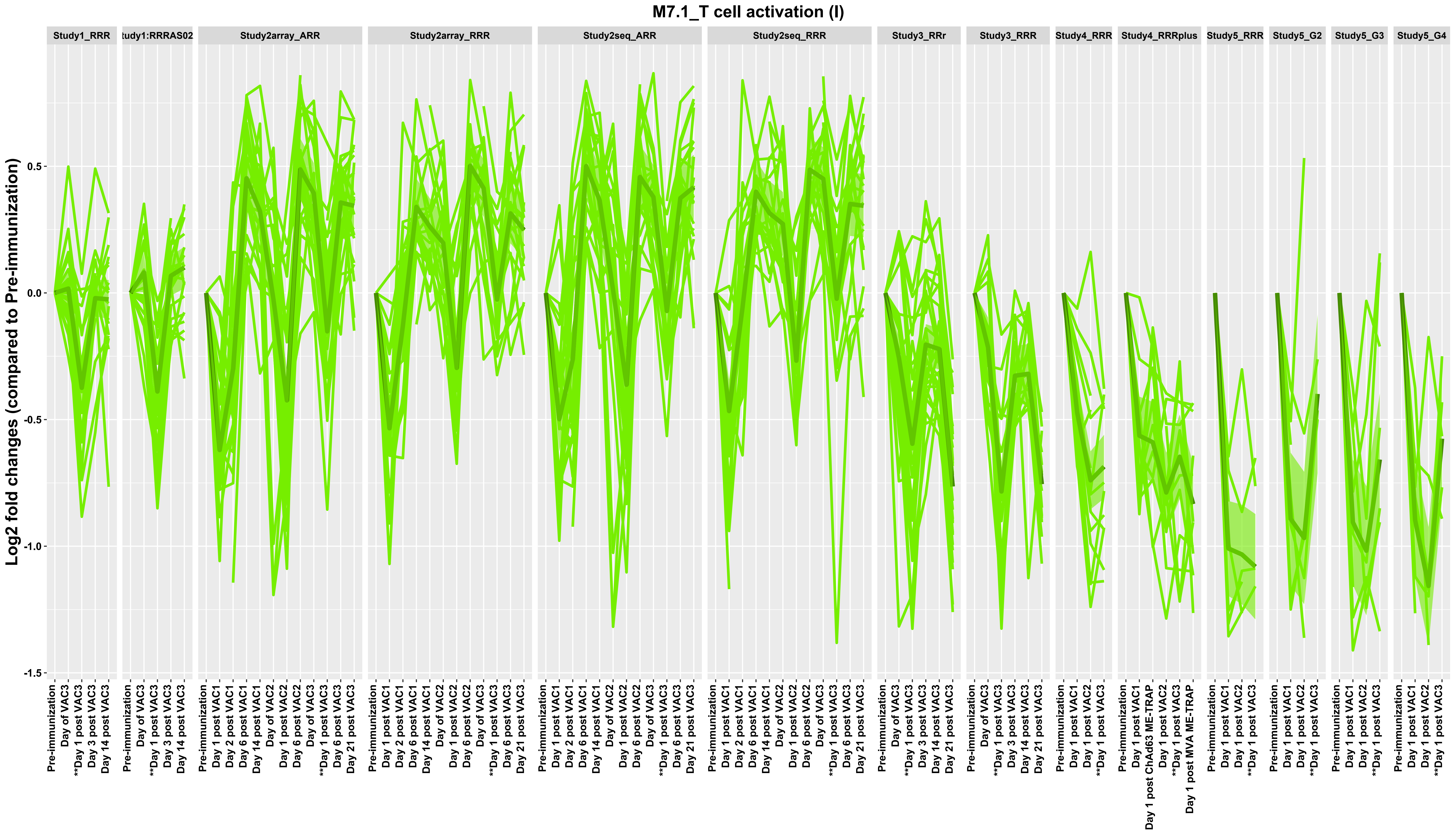
M7.1_T cell activation (I)
Log2 fold changes (compared to Pre-immunization)
Study1_RRR
tudy1:RRRAS02
Study2array_ARR
Study2array_RRR
Study2seq_ARR
Study2seq_RRR
Study3_RRr
Study3_RRR
Study4_RRR
Study4_RRRplus
Study5_RRR
Study5_G2
Study5_G3
Study5_G4
0.5
0.0
-0.5
-1.0
-1.5
Pre-immunization
Day of VAC3
**Day 1 post VAC3
Day 3 post VAC3
Day 14 post VAC3
Day 1 post VAC1
Day 2 post VAC1
Day 6 post VAC1
Day 14 post VAC1
Day of VAC2
Day 1 post VAC2
Day 6 post VAC2
Day 6 post VAC3
Day 21 post VAC3
Day 1 post ChAd63 ME-TRAP
Day 1 post MVA ME-TRAP
**Day 1 post VAC3

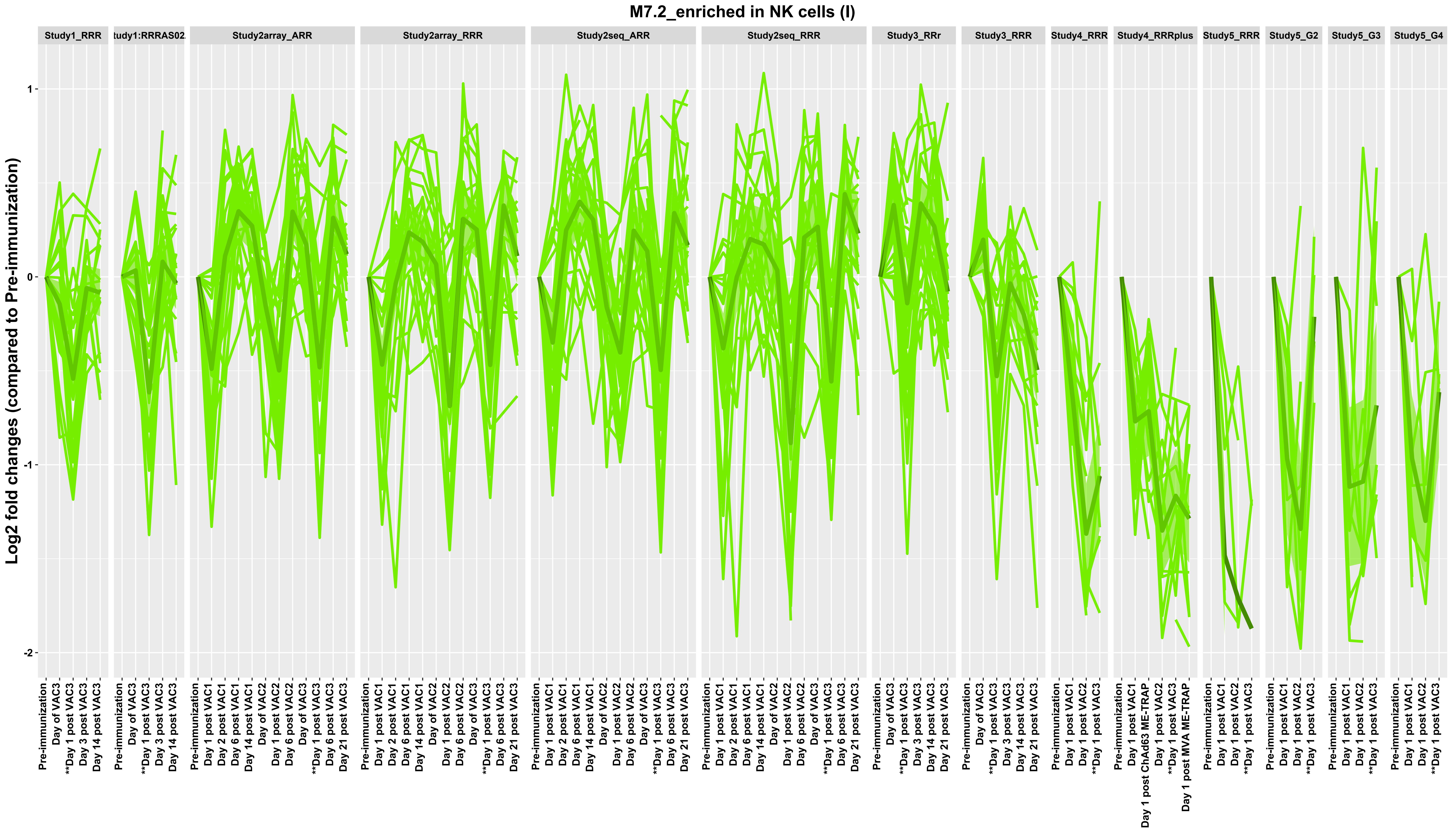
M7.2_enriched in NK cells (I)
Study1_RRR
tudy1:RRRAS02
Study2array_ARR
Study2array_RRR
Study2seq_ARR
Study2seq_RRR
Study3_RRr
Study3_RRR
Study4_RRR
Study4_RRRplus
Study5_RRR
Study5_G2
Study5_G3
Study5_G4
Log2 fold changes (compared to Pre-immunization)
1
0
-1
-2
Pre-immunization
Day of VAC3
**Day 1 post VAC3
Day 3 post VAC3
Day 14 post VAC3
Day 1 post VAC1
Day 2 post VAC1
Day 6 post VAC1
Day 14 post VAC1
Day of VAC2
Day 1 post VAC2
Day 6 post VAC2
Day 6 post VAC3
Day 21 post VAC3
Day 1 post ChAd63 ME-TRAP
Day 1 post MVA ME-TRAP
**Day 1 post VAC3

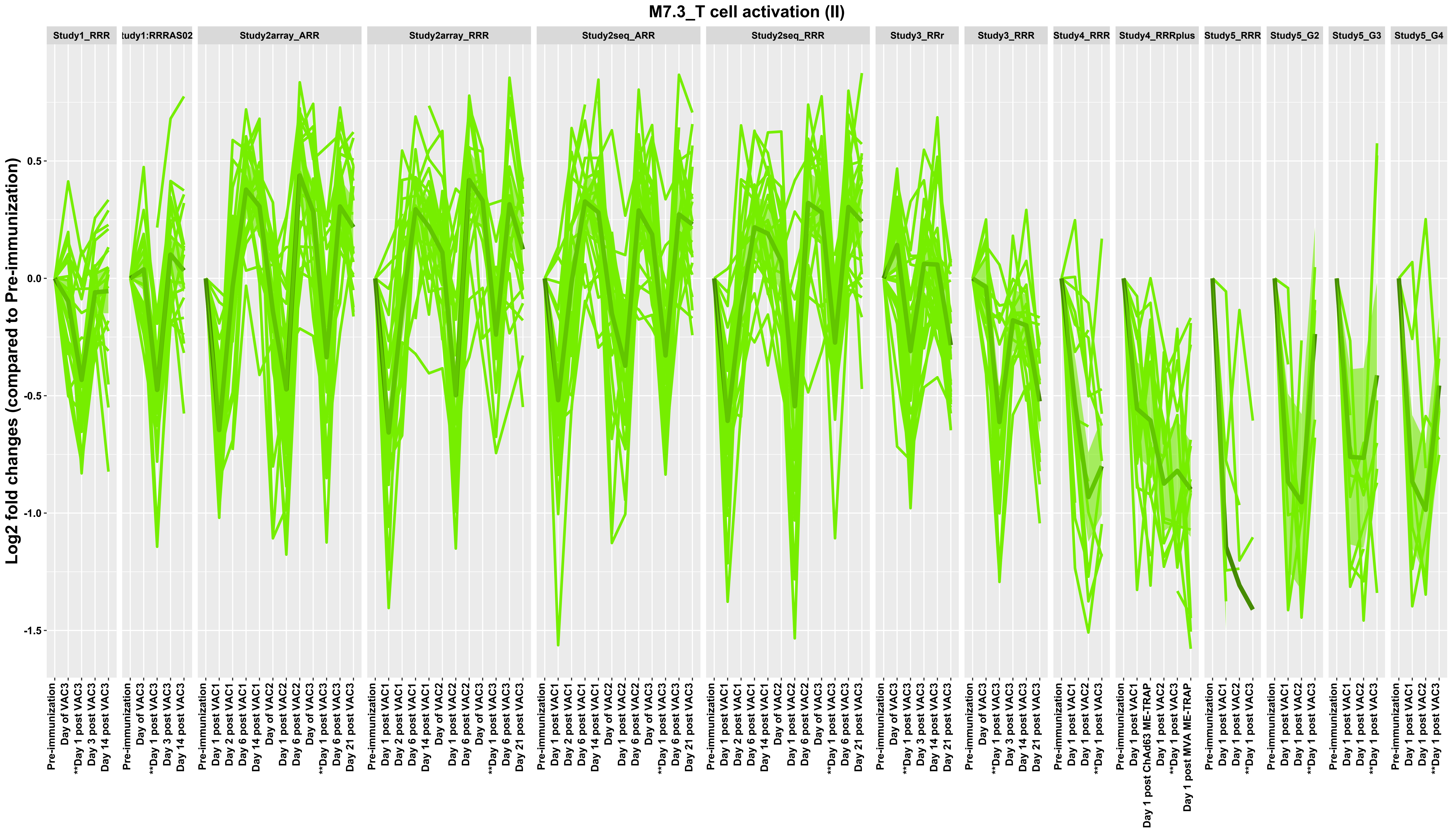
M7.3_T cell activation (II)
Log2 fold changes (compared to Pre-immunization)
0.5
0.0
-0.5
-1.0
-1.5
Study1_RRR
tudy1:RRRAS02
Study2array_ARR
Study2array_RRR
Study2seq_ARR
Study2seq_RRR
Study3_RRr
Study3_RRR
Study4_RRR
Study4_RRRplus
Study5_RRR
Study5_G2
Study5_G3
Study5_G4
Pre-immunization
Day of VAC3
**Day 1 post VAC3
Day 3 post VAC3
Day 14 post VAC3
Day 1 post VAC1
Day 2 post VAC1
Day 6 post VAC1
Day 14 post VAC1
Day of VAC2
Day 1 post VAC2
Day 6 post VAC2
Day 6 post VAC3
Day 21 post VAC3
Day 21 post VAC3
Day 1 post ChAd63 ME-TRAP
Day 1 post MVA ME-TRAP
**Day 1 post VAC2

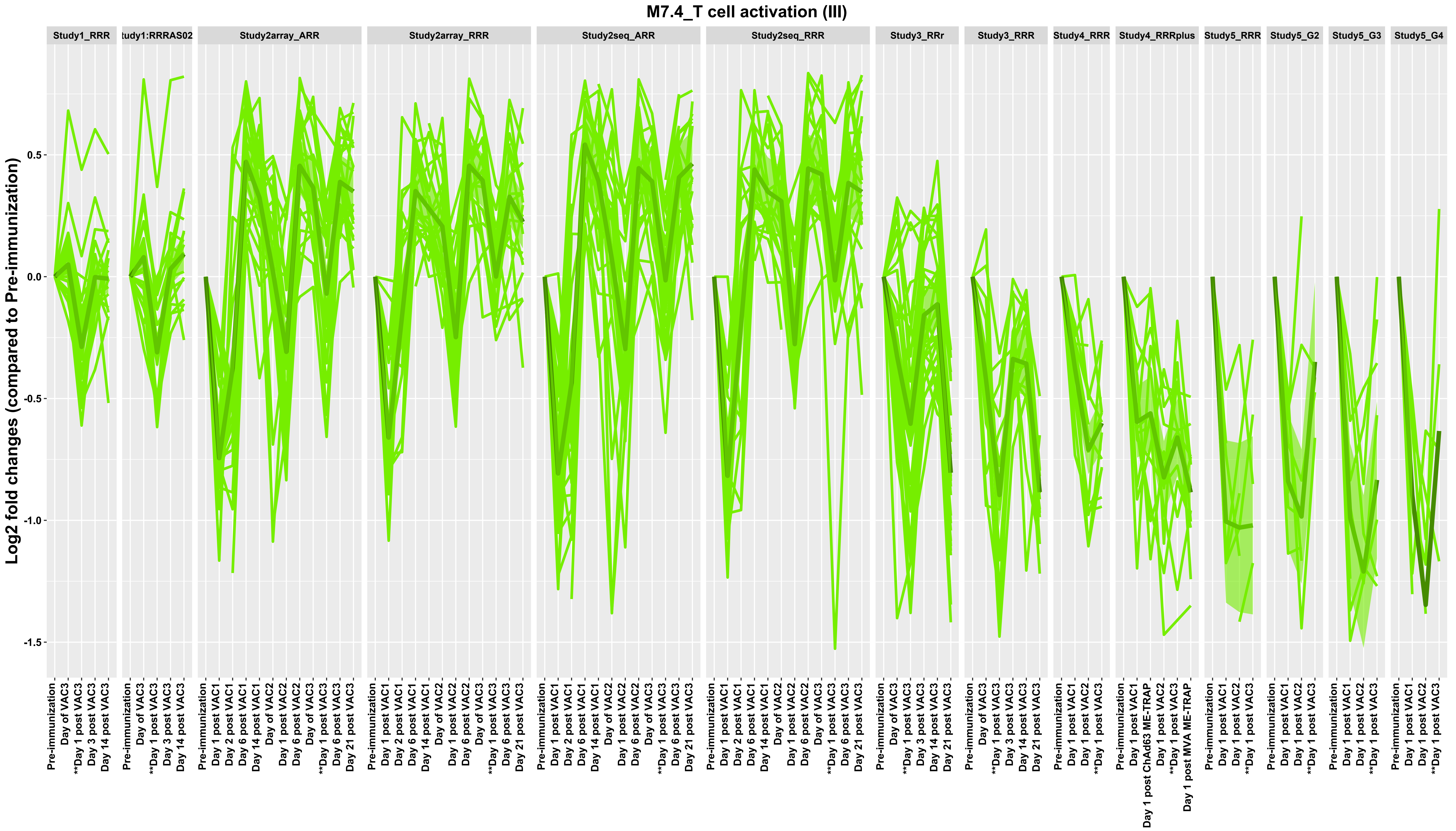

M7.4_T cell activation (III)
Log2 fold changes (compared to Pre-immunization)
0.5
0.0
-0.5
-1.0
-1.5
Study1_RRR
tudy1:RRRAS02
Study2array_ARR
Study2array_RRR
Study2seq_ARR
Study2seq_RRR
Study3_RRr
Study3_RRR
Study4_RRR
Study4_RRRplus
Study5_RRR
Study5_G2
Study5_G3
Study5_G4
Pre-immunization
Day of VAC3
**Day 1 post VAC3
Day 3 post VAC3
Day 14 post VAC3
Day 1 post VAC1
Day 2 post VAC1
Day 6 post VAC1
Day 14 post VAC1
Day of VAC2
Day 1 post VAC2
Day 6 post VAC2
Day 6 post VAC3
Day 21 post VAC3
Day 1 post ChAd63 ME-TRAP
Day 1 post MVA ME-TRAP

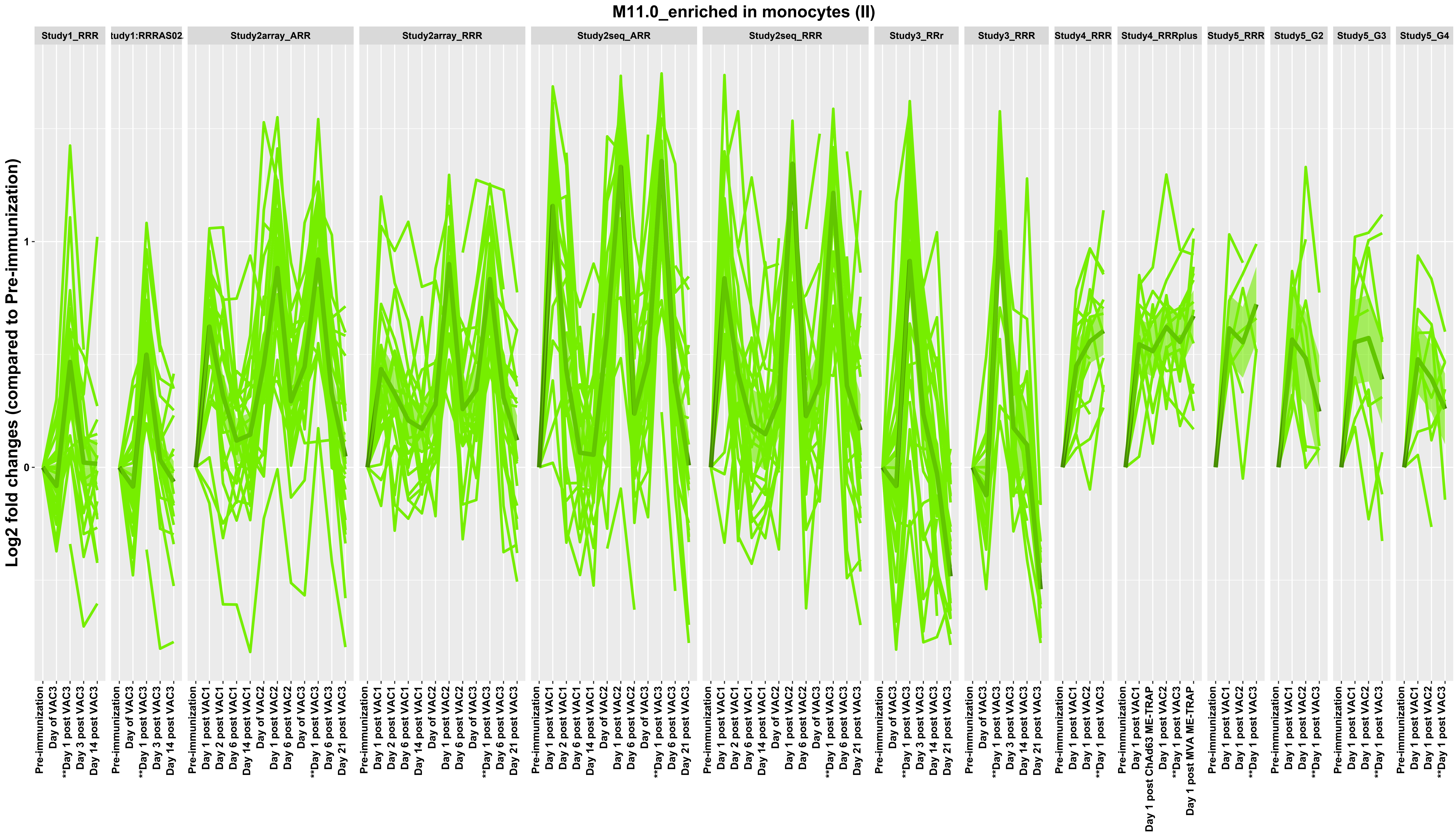
M11.0_enriched in monocytes (II)
Log2 fold changes (compared to Pre-immunization)
Study1_RRR
tudy1:RRRAS02
Study2array_ARR
Study2array_RRR
Study2seq_ARR
Study2seq_RRR
Study3_RRr
Study3_RRR
Study4_RRR
Study4_RRRplus
Study5_RRR
Study5_G2
Study5_G3
Study5_G4
1
0
Pre-immunization
Day of VAC3
**Day 1 post VAC3
Day 3 post VAC3
Day 14 post VAC3
Day 1 post VAC1
Day 2 post VAC1
Day 6 post VAC1
Day 14 post VAC1
Day of VAC2
Day 1 post VAC2
Day 6 post VAC2
Day 6 post VAC3
Day 21 post VAC3
Day 21 post VAC3
Day 1 post ChAd63 ME-TRAP
Day 1 post MVA ME-TRAP

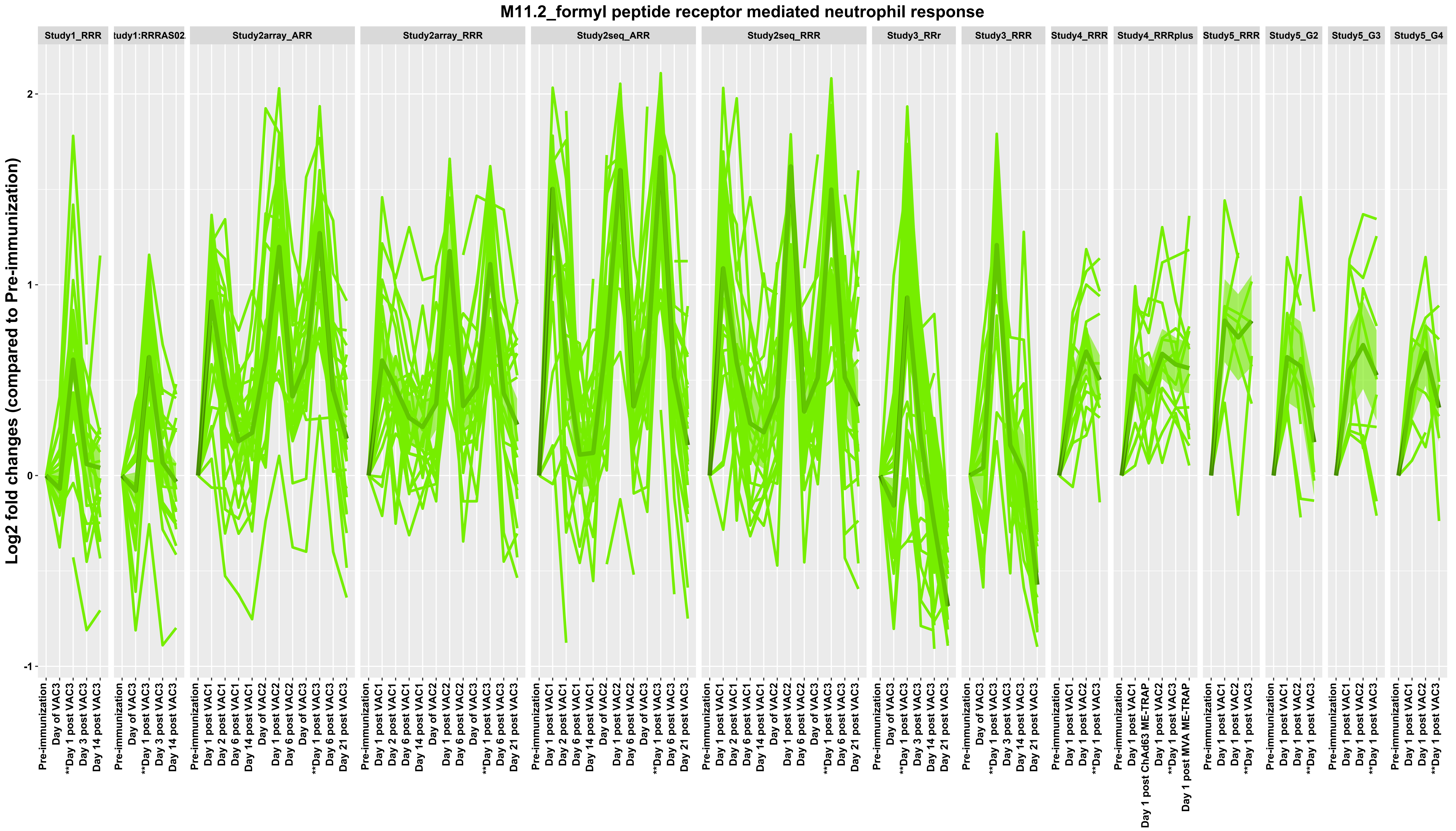
M11.2_formyl peptide receptor mediated neutrophil response
Log2 fold changes (compared to Pre-immunization)
Study1_RRR
tudy1:RRRAS02
Study2array_ARR
Study2array_RRR
Study2seq_ARR
Study2seq_RRR
Study3_RRr
Study3_RRR
Study4_RRR
Study4_RRRplus
Study5_RRR
Study5_G2
Study5_G3
Study5_G4
Pre-immunization
Day of VAC3
**Day 1 post VAC3
Day 3 post VAC3
Day 14 post VAC3
Day 1 post VAC1
Day 2 post VAC1
Day 6 post VAC1
Day 14 post VAC1
Day of VAC2
Day 1 post VAC2
Day 6 post VAC2
Day 6 post VAC3
Day 21 post VAC3
Day 1 post ChAd63 ME-TRAP
Day 1 post MVA ME-TRAP

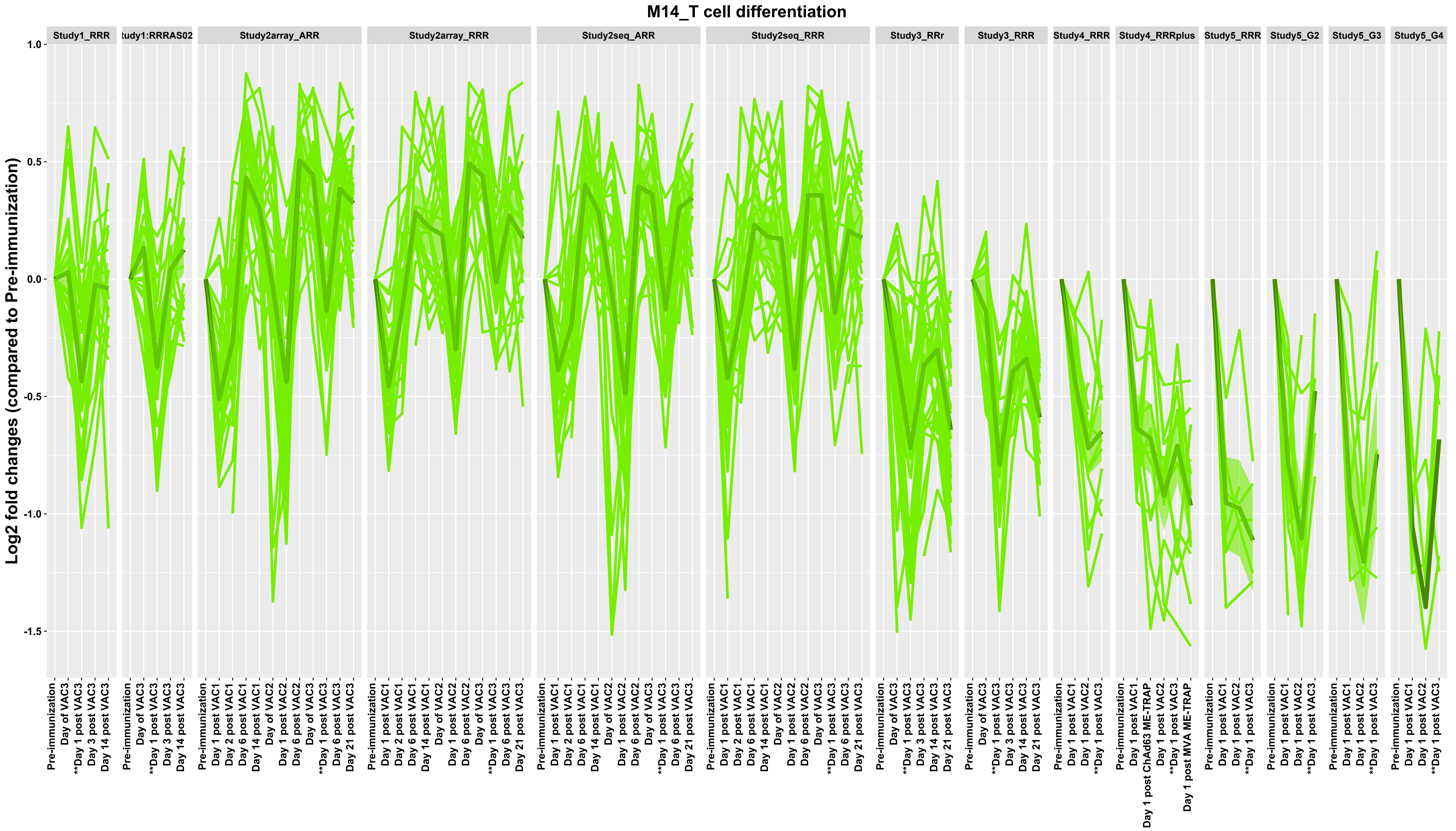

M14_T cell differentiation
Log2 fold changes (compared to Pre-immunization)
1.0
0.5
0.0
-0.5
-1.0
-1.5
Study1_RRR
tudy1:RRRAS02
Study2array_ARR
Study2array_RRR
Study2seq_ARR
Study2seq_RRR
Study3_RRr
Study3_RRR
Study4_RRR
Study4_RRRplus
Study5_RRR
Study5_G2
Study5_G3
Study5_G4
Pre-immunization
Day of VAC3
**Day 1 post VAC3
Day 3 post VAC3
Day 14 post VAC3
Day 1 post VAC1
Day 2 post VAC1
Day 6 post VAC1
Day 14 post VAC1
Day of VAC2
Day 1 post VAC2
Day 6 post VAC2
Day 6 post VAC3
Day 21 post VAC3
Day 21 post VAC3
Day 1 post ChAd63 ME-TRAP
Day 1 post MVA ME-TRAP
**Day 1 post VAC3

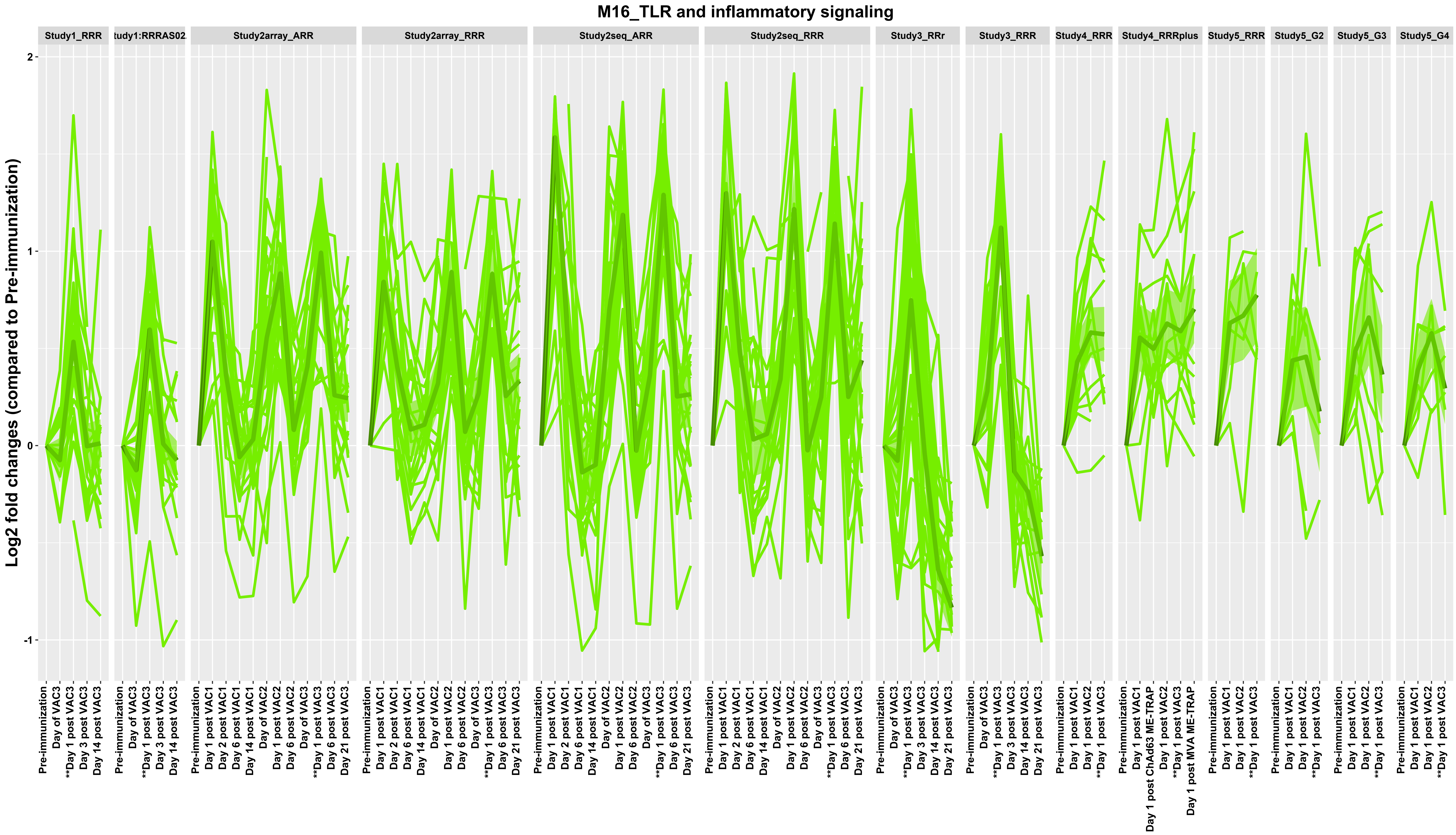

M16_TLR and inflammatory signaling
Study1_RRR
tudy1:RRRAS02
Study2array_ARR
Study2array_RRR
Study2seq_ARR
Study2seq_RRR
Study3_RRr
Study3_RRR
Study4_RRR
Study4_RRRplus
Study5_RRR
Study5_G2
Study5_G3
Study5_G4
Log2 fold changes (compared to Pre-immunization)
2
1
0
-1
Pre-immunization
Day of VAC3
**Day 1 post VAC3
Day 3 post VAC3
Day 14 post VAC3
Day 1 post VAC1
Day 2 post VAC1
Day 6 post VAC1
Day 14 post VAC1
Day of VAC2
Day 1 post VAC2
Day 6 post VAC2
Day 6 post VAC3
Day 21 post VAC3
Day 1 post ChAd63 ME-TRAP
Day 1 post MVA ME-TRAP

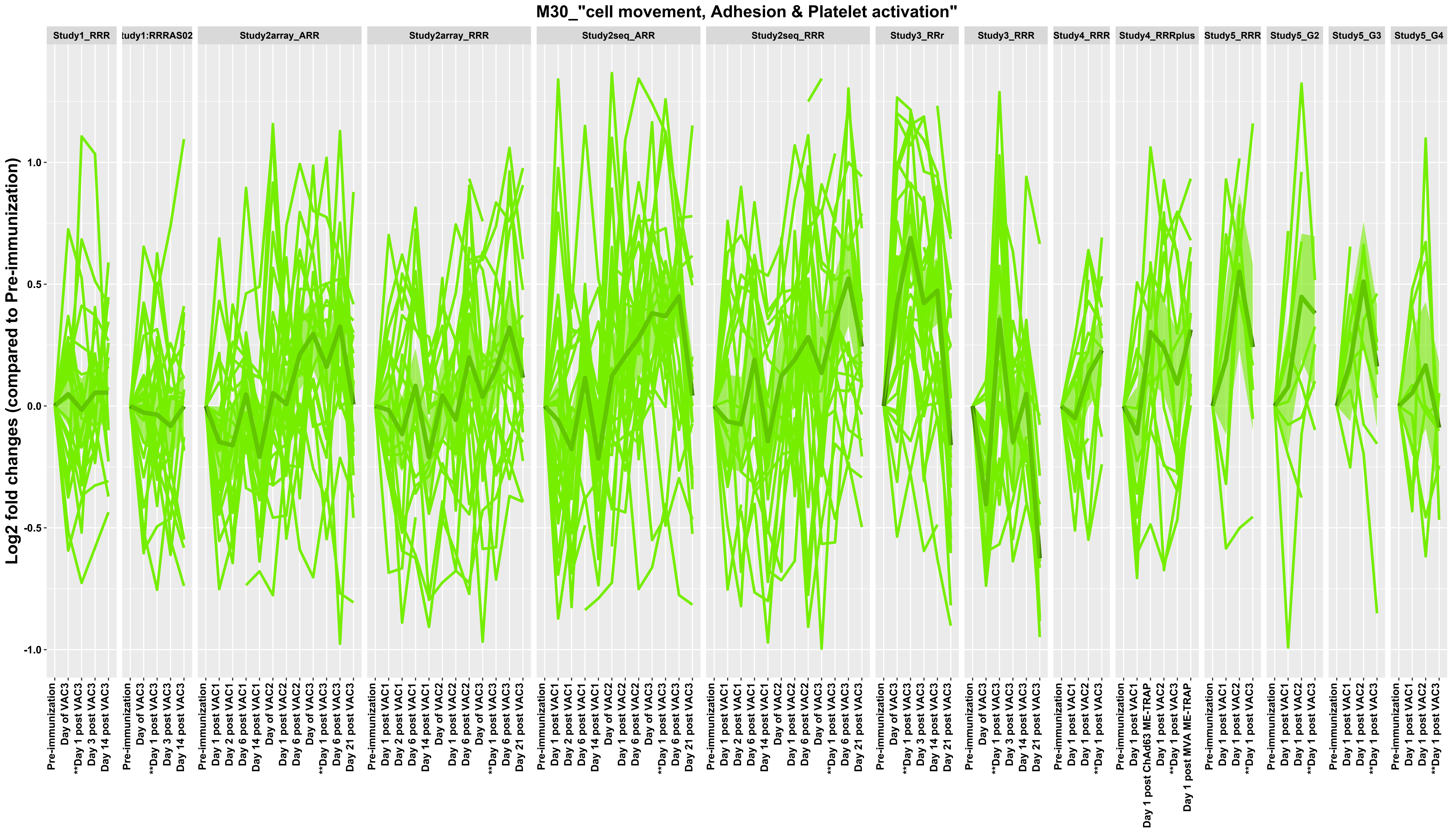
M30_"cell movement, Adhesion & Platelet activation"
Log2 fold changes (compared to Pre-immunization)
1.0
0.5
0.0
-0.5
-1.0
Study1_RRR
tudy1:RRRAS02
Study2array_ARR
Study2array_RRR
Study2seq_ARR
Study2seq_RRR
Study3_RRr
Study3_RRR
Study4_RRR
Study4_RRRplus
Study5_RRR
Study5_G2
Study5_G3
Study5_G4
Pre-immunization
Day of VAC3
**Day 1 post VAC3
Day 3 post VAC3
Day 14 post VAC3
Pre-immunization
Day of VAC3
**Day 1 post VAC3
Day 3 post VAC3
Day 14 post VAC3
Pre-immunization
Day 1 post VAC1
Day 2 post VAC1
Day 6 post VAC1
Day 14 post VAC1
Day of VAC2
Day 1 post VAC2
Day 6 post VAC2
Day of VAC3
**Day 1 post VAC3
Day 6 post VAC3
Day 21 post VAC3
Pre-immunization
Day 1 post VAC1
Day 2 post VAC1
Day 6 post VAC1
Day 14 post VAC1
Day of VAC2
Day 1 post VAC2
Day 6 post VAC2
Day of VAC3
**Day 1 post VAC3
Day 6 post VAC3
Day 21 post VAC3
Pre-immunization
Day 1 post VAC1
Day 2 post VAC1
Day 6 post VAC1
Day 14 post VAC1
Day of VAC2
Day 1 post VAC2
Day 6 post VAC2
Day of VAC3
**Day 1 post VAC3
Day 6 post VAC3
Day 21 post VAC3
Pre-immunization
Day 1 post VAC1
Day 2 post VAC1
Day 6 post VAC1
Day 14 post VAC1
Day of VAC2
Day 1 post VAC2
Day 6 post VAC2
Day of VAC3
**Day 1 post VAC3
Day 6 post VAC3
Day 21 post VAC3
Pre-immunization
Day of VAC3
**Day 1 post VAC3
Day 3 post VAC3
Day 14 post VAC3
Day 21 post VAC3
Pre-immunization
Day of VAC3
**Day 1 post VAC3
Day 3 post VAC3
Day 14 post VAC3
Day 21 post VAC3
Pre-immunization
Day 1 post VAC1
Day 1 post VAC2
**Day 1 post VAC3
Pre-immunization
Day 1 post VAC1
Day 1 post ChAd63 ME-TRAP
Day 1 post VAC2
**Day 1 post VAC3
Day 1 post MVA ME-TRAP
Pre-immunization
Day 1 post VAC1
Day 1 post VAC2
**Day 1 post VAC3
Pre-immunization
Day 1 post VAC1
Day 1 post VAC2
**Day 1 post VAC3
Pre-immunization
Day 1 post VAC1
Day 1 post VAC2
**Day 1 post VAC3
Pre-immunization
Day 1 post VAC1
Day 1 post VAC2
**Day 1 post VAC3

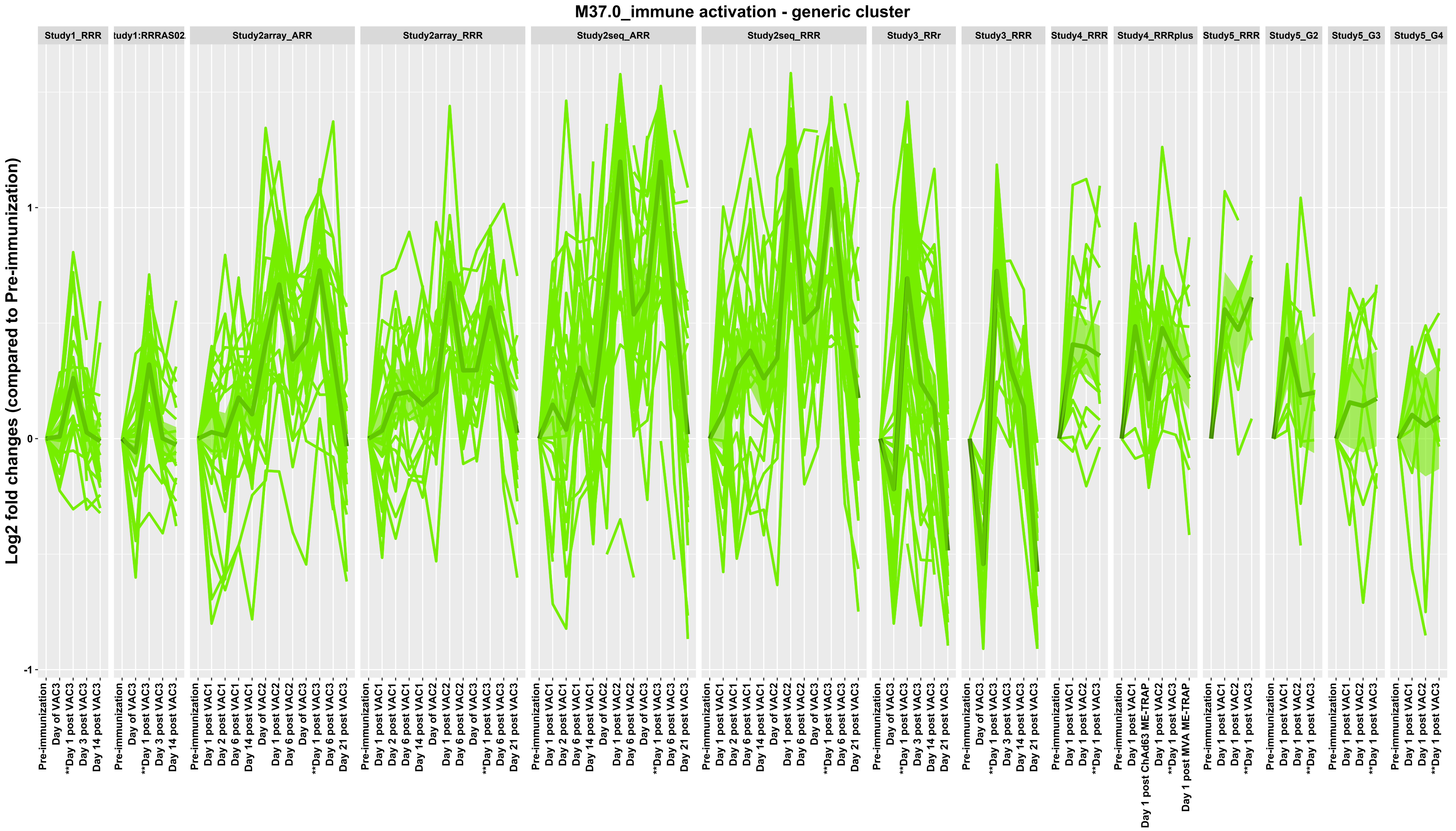
M37.0_immune activation - generic cluster
Study1_RRR
tudy1:RRRAS02
Study2array_ARR
Study2array_RRR
Study2seq_ARR
Study2seq_RRR
Study3_RRr
Study3_RRR
Study4_RRR
Study4_RRRplus
Study5_RRR
Study5_G2
Study5_G3
Study5_G4
Log2 fold changes (compared to Pre-immunization)
1
0
-1
Pre-immunization
Day of VAC3
**Day 1 post VAC3
Day 3 post VAC3
Day 14 post VAC3
Day 1 post VAC1
Day 2 post VAC1
Day 6 post VAC1
Day 14 post VAC1
Day of VAC2
Day 1 post VAC2
Day 6 post VAC2
Day 6 post VAC3
Day 21 post VAC3
Day 1 post ChAd63 ME-TRAP
Day 1 post MVA ME-TRAP
**Day 1 post VAC3

M37.1_enriched in neutrophils (I)

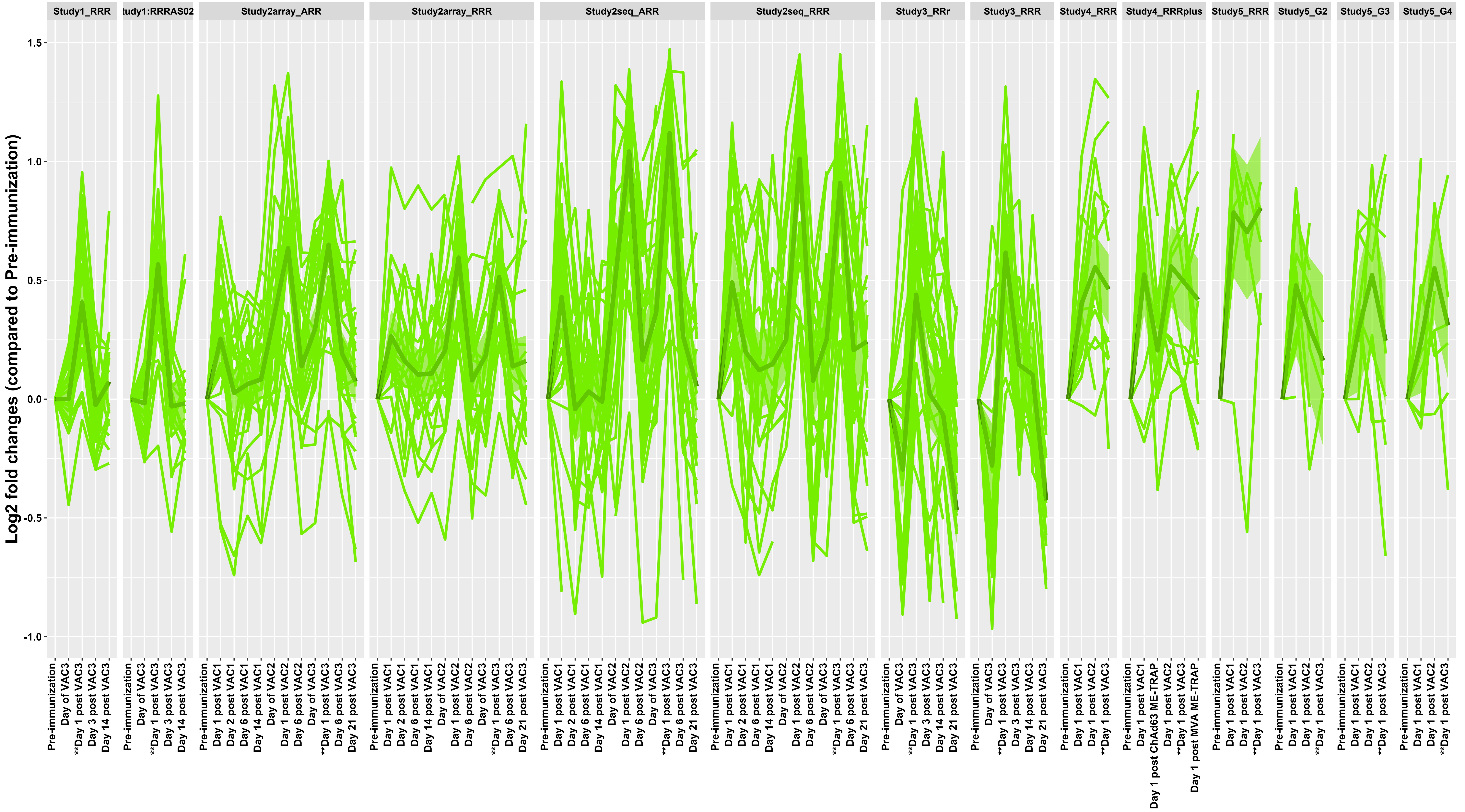

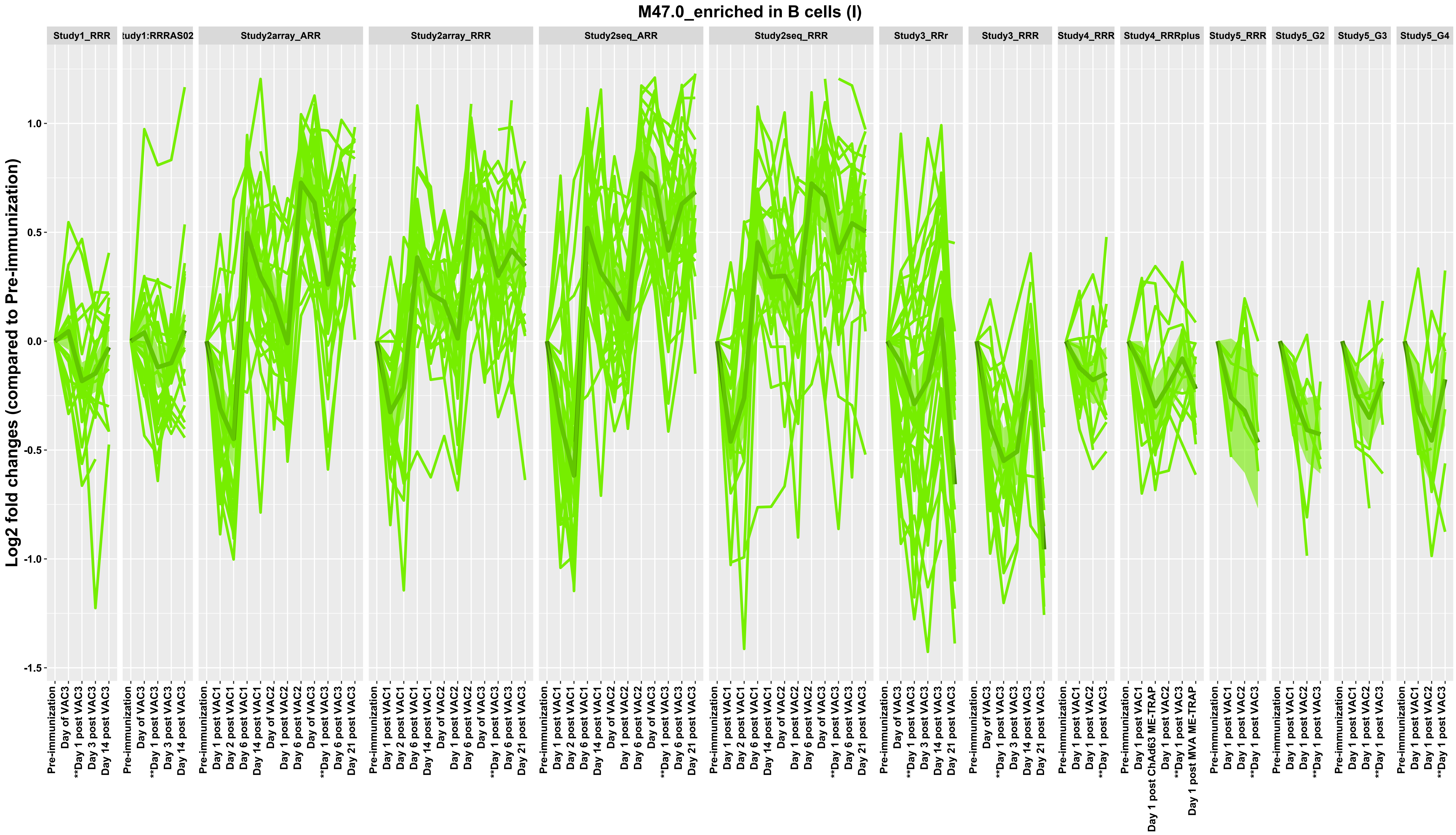
M47.0_enriched in B cells (I)
Study1_RRR
tudy1:RRRAS02
Study2array_ARR
Study2array_RRR
Study2seq_ARR
Study2seq_RRR
Study3_RRr
Study3_RRR
Study4_RRR
Study4_RRRplus
Study5_RRR
Study5_G2
Study5_G3
Study5_G4
Log2 fold changes (compared to Pre-immunization)
1.0
0.5
0.0
-0.5
-1.0
-1.5
Pre-immunization
Day of VAC3
**Day 1 post VAC3
Day 3 post VAC3
Day 14 post VAC3
Day 1 post VAC1
Day 2 post VAC1
Day 6 post VAC1
Day 14 post VAC1
Day of VAC2
Day 1 post VAC2
Day 6 post VAC2
Day 6 post VAC3
Day 21 post VAC3
Day 1 post ChAd63 ME-TRAP
**Day 1 post VAC3
Day 1 post MVA ME-TRAP

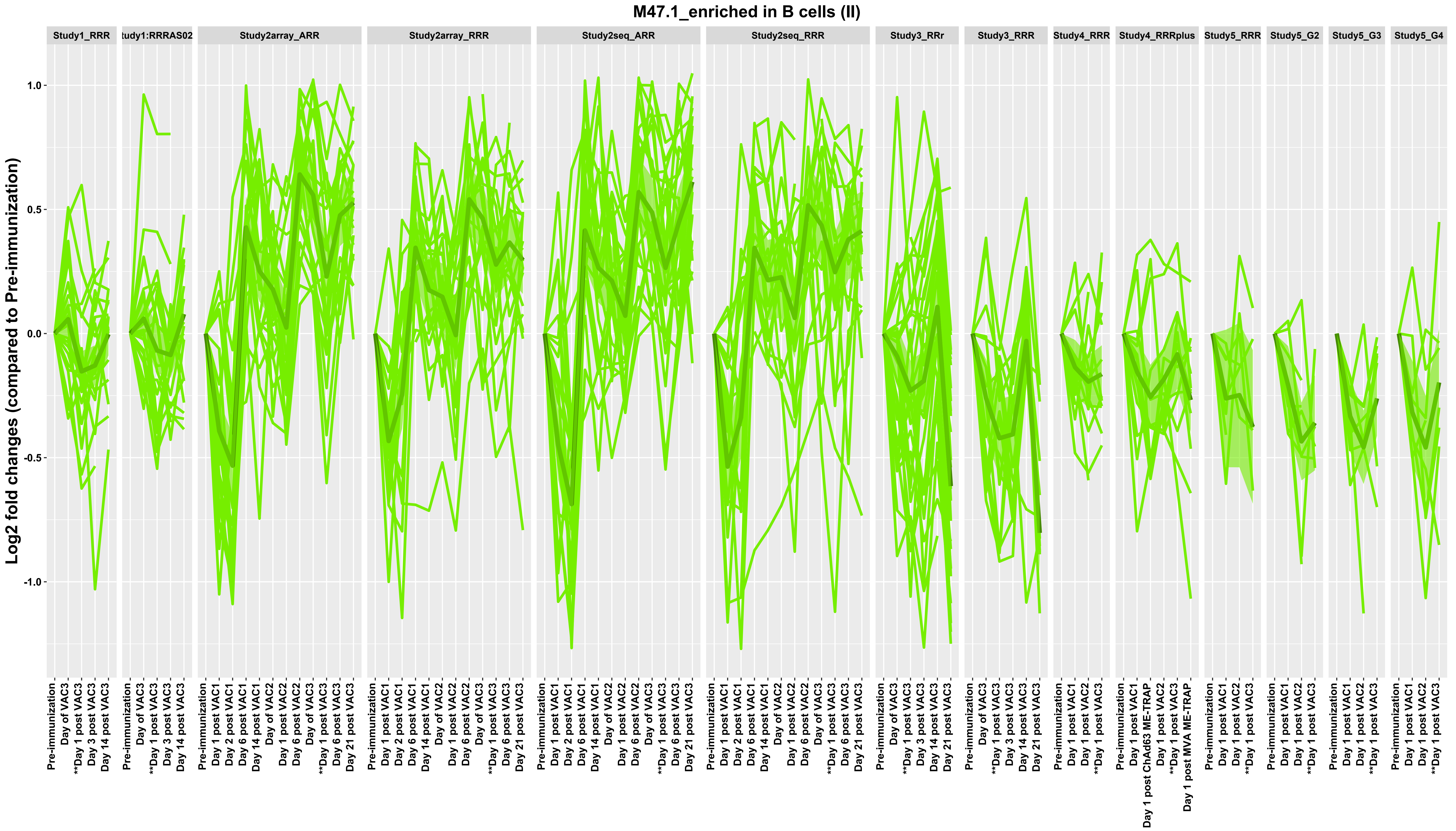

M47.1_enriched in B cells (II)
Study1_RRR
tudy1:RRRAS02
Study2array_ARR
Study2array_RRR
Study2seq_ARR
Study2seq_RRR
Study3_RRr
Study3_RRR
Study4_RRR
Study4_RRRplus
Study5_RRR
Study5_G2
Study5_G3
Study5_G4
Log2 fold changes (compared to Pre-immunization)
1.0
0.5
0.0
-0.5
-1.0
Pre-immunization
Day of VAC3
**Day 1 post VAC3
Day 3 post VAC3
Day 14 post VAC3
Day 1 post VAC1
Day 2 post VAC1
Day 6 post VAC1
Day 14 post VAC1
Day of VAC2
Day 1 post VAC2
Day 6 post VAC2
Day 6 post VAC3
Day 21 post VAC3
Day 1 post ChAd63 ME-TRAP
Day 1 post MVA ME-TRAP
**Day 1 post VAC2

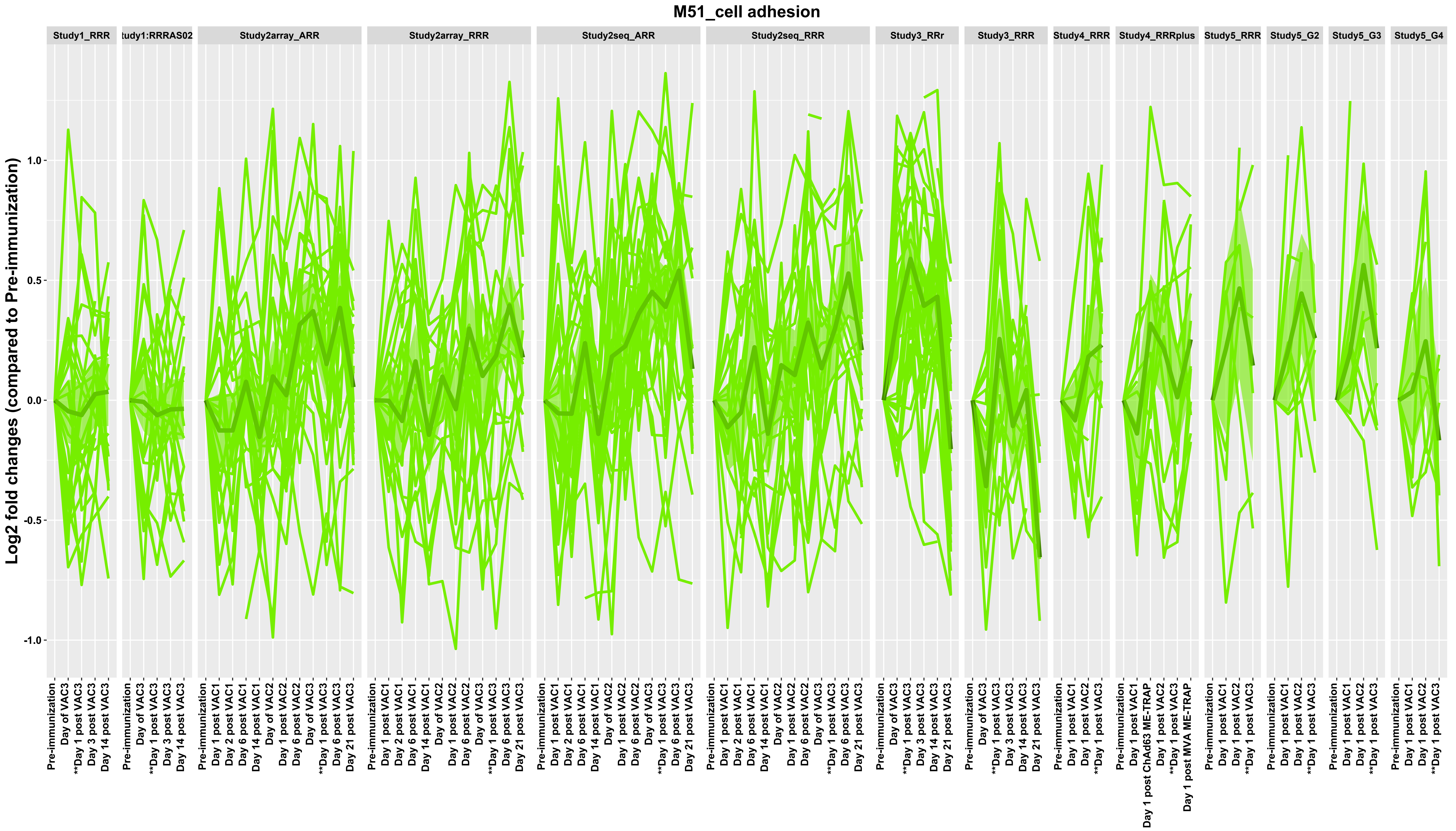
M51_cell adhesion
Study1_RRR
tudy1:RRRAS02
Study2array_ARR
Study2array_RRR
Study2seq_ARR
Study2seq_RRR
Study3_RRr
Study3_RRR
Study4_RRR
Study4_RRRplus
Study5_RRR
Study5_G2
Study5_G3
Study5_G4
Log2 fold changes (compared to Pre-immunization)
1.0
0.5
0.0
-0.5
-1.0
Pre-immunization
Day of VAC3
**Day 1 post VAC3
Day 3 post VAC3
Day 14 post VAC3
Day 1 post VAC1
Day 2 post VAC1
Day 6 post VAC1
Day 14 post VAC1
Day of VAC2
Day 1 post VAC2
Day 6 post VAC2
Day 6 post VAC3
Day 21 post VAC3
**Day 1 post VAC2
Day 1 post ChAd63 ME-TRAP
Day 1 post MVA ME-TRAP

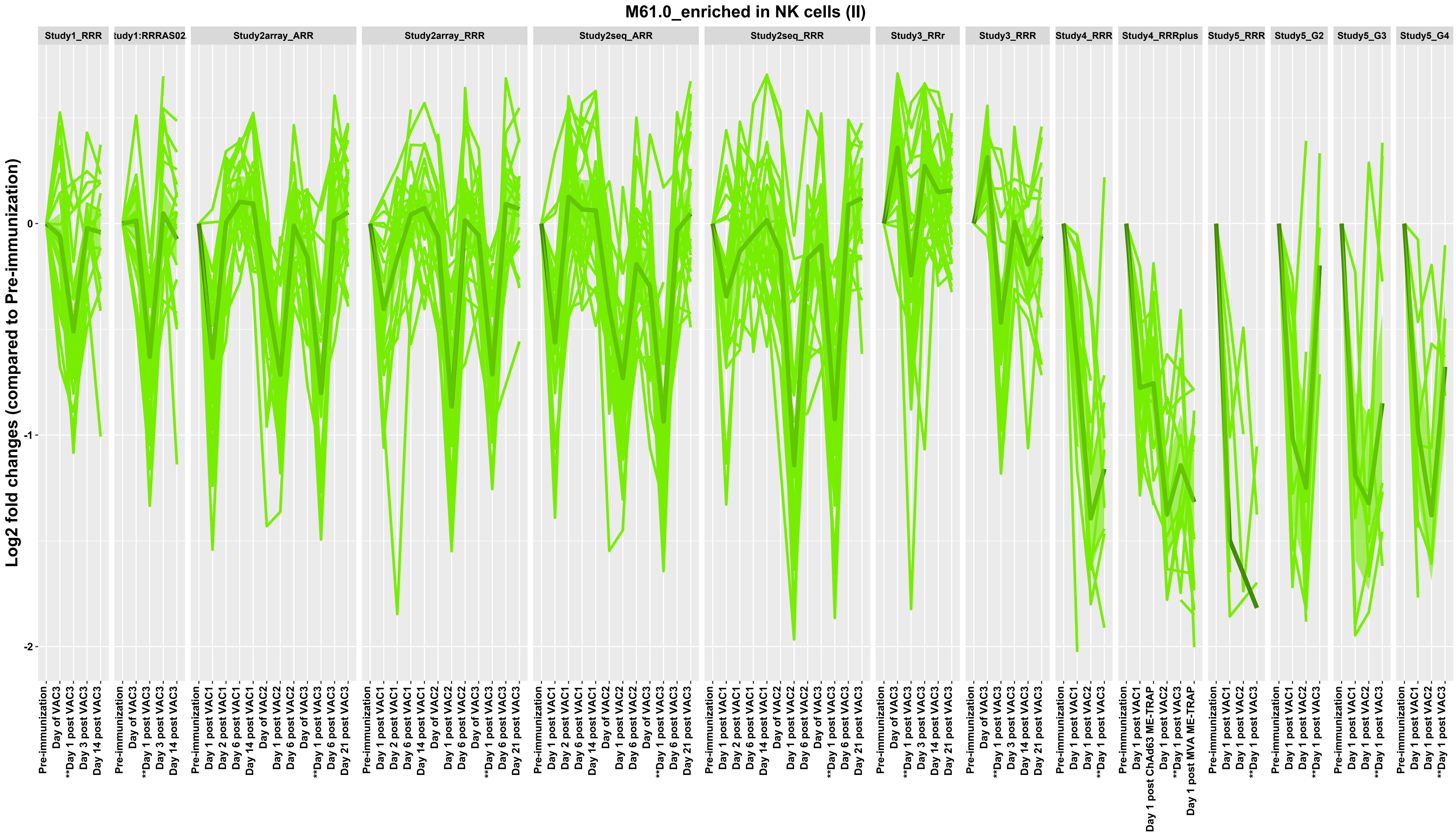
M61.0_enriched in NK cells (II)
Study1_RRR
tudy1:RRRAS02
Study2array_ARR
Study2array_RRR
Study2seq_ARR
Study2seq_RRR
Study3_RRr
Study3_RRR
Study4_RRR
Study4_RRRplus
Study5_RRR
Study5_G2
Study5_G3
Study5_G4
Log2 fold changes (compared to Pre-immunization)
0
-1
-2
Pre-immunization
Day of VAC3
**Day 1 post VAC3
Day 3 post VAC3
Day 14 post VAC3
Day 1 post VAC1
Day 2 post VAC1
Day 6 post VAC1
Day 14 post VAC1
Day of VAC2
Day 1 post VAC2
Day 6 post VAC2
Day of VAC3
Day 6 post VAC3
Day 21 post VAC3
Day 1 post ChAd63 ME-TRAP
Day 1 post MVA ME-TRAP

M61.2_enriched in NK cells (receptor activation)

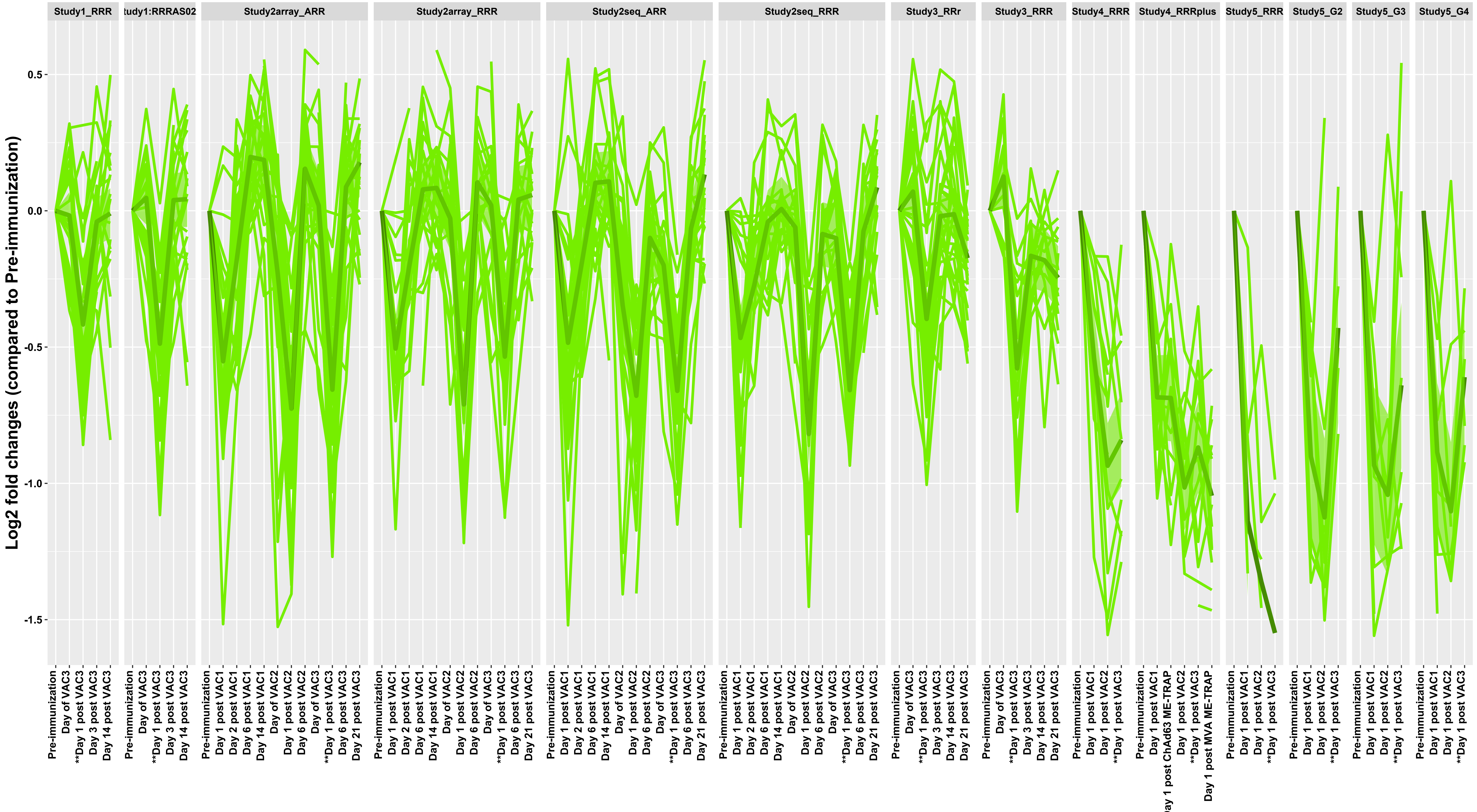

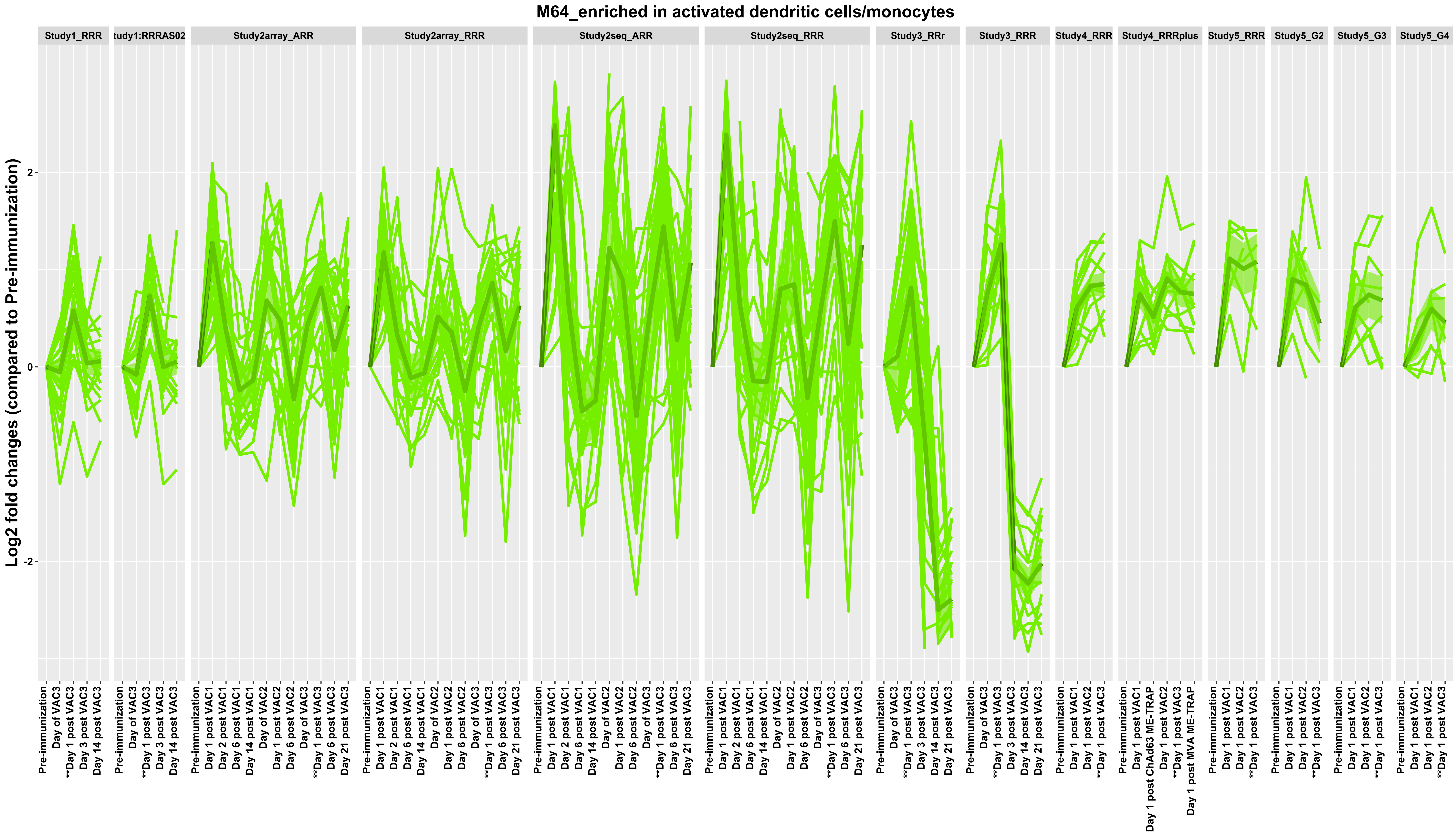
M64_enriched in activated dendritic cells/monocytes
Log2 fold changes (compared to Pre-immunization)
Study1_RRR
tudy1:RRRAS02
Study2array_ARR
Study2array_RRR
Study2seq_ARR
Study2seq_RRR
Study3_RRr
Study3_RRR
Study4_RRR
Study4_RRRplus
Study5_RRR
Study5_G2
Study5_G3
Study5_G4
2
0
-2
Pre-immunization
Day of VAC3
**Day 1 post VAC3
Day 3 post VAC3
Day 14 post VAC3
Day 1 post VAC1
Day 2 post VAC1
Day 6 post VAC1
Day 14 post VAC1
Day of VAC2
Day 1 post VAC2
Day 6 post VAC2
Day 6 post VAC3
Day 21 post VAC3
Day 1 post ChAd63 ME-TRAP
**Day 1 post VAC2
Day 1 post MVA ME-TRAP

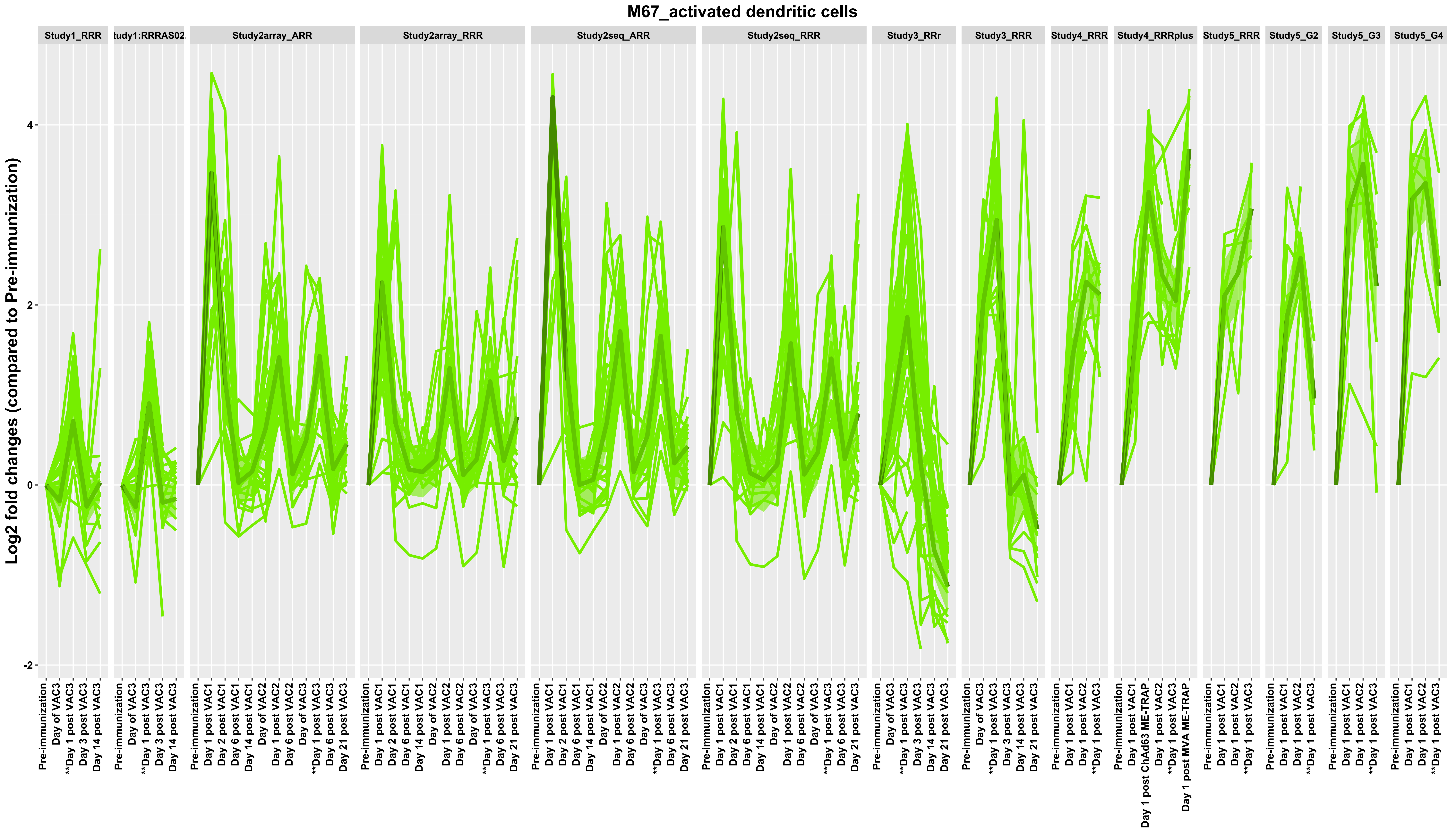
M67_activated dendritic cells
Study1_RRR
tudy1:RRRAS02
Study2array_ARR
Study2array_RRR
Study2seq_ARR
Study2seq_RRR
Study3_RRr
Study3_RRR
Study4_RRR
Study4_RRRplus
Study5_RRR
Study5_G2
Study5_G3
Study5_G4
Log2 fold changes (compared to Pre-immunization)
4
2
0
-2
Pre-immunization
Day of VAC3
**Day 1 post VAC3
Day 3 post VAC3
Day 14 post VAC3
Day 1 post VAC1
Day 2 post VAC1
Day 6 post VAC1
Day 14 post VAC1
Day of VAC2
Day 1 post VAC2
Day 6 post VAC2
Day 6 post VAC3
Day 21 post VAC3
**Day 1 post VAC3
Day 1 post ChAd63 ME-TRAP
Day 1 post MVA ME-TRAP

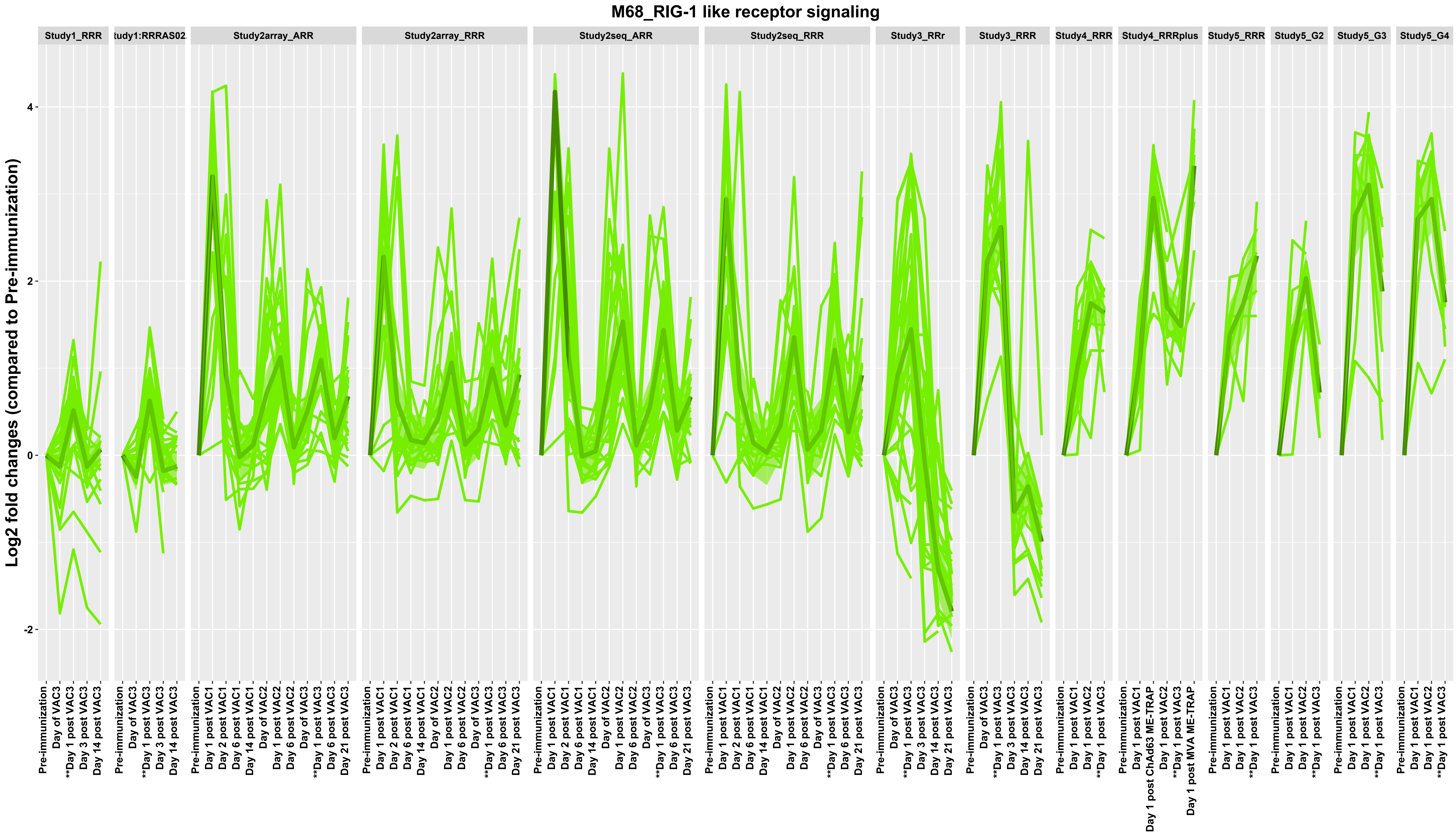
M68_RIG-1 like receptor signaling
Log2 fold changes (compared to Pre-immunization)
4
2
0
-2
Study1_RRR
tudy1:RRRAS02
Study2array_ARR
Study2array_RRR
Study2seq_ARR
Study2seq_RRR
Study3_RRr
Study3_RRR
Study4_RRR
Study4_RRRplus
Study5_RRR
Study5_G2
Study5_G3
Study5_G4
Pre-immunization
Day of VAC3
**Day 1 post VAC3
Day 3 post VAC3
Day 14 post VAC3
Day 1 post VAC1
Day 2 post VAC1
Day 6 post VAC1
Day 14 post VAC1
Day of VAC2
Day 1 post VAC2
Day 6 post VAC2
Day 6 post VAC3
Day 21 post VAC3
**Day 1 post VAC1
Day 1 post ChAd63 ME-TRAP
Day 1 post MVA ME-TRAP
**Day 1 post VAC2

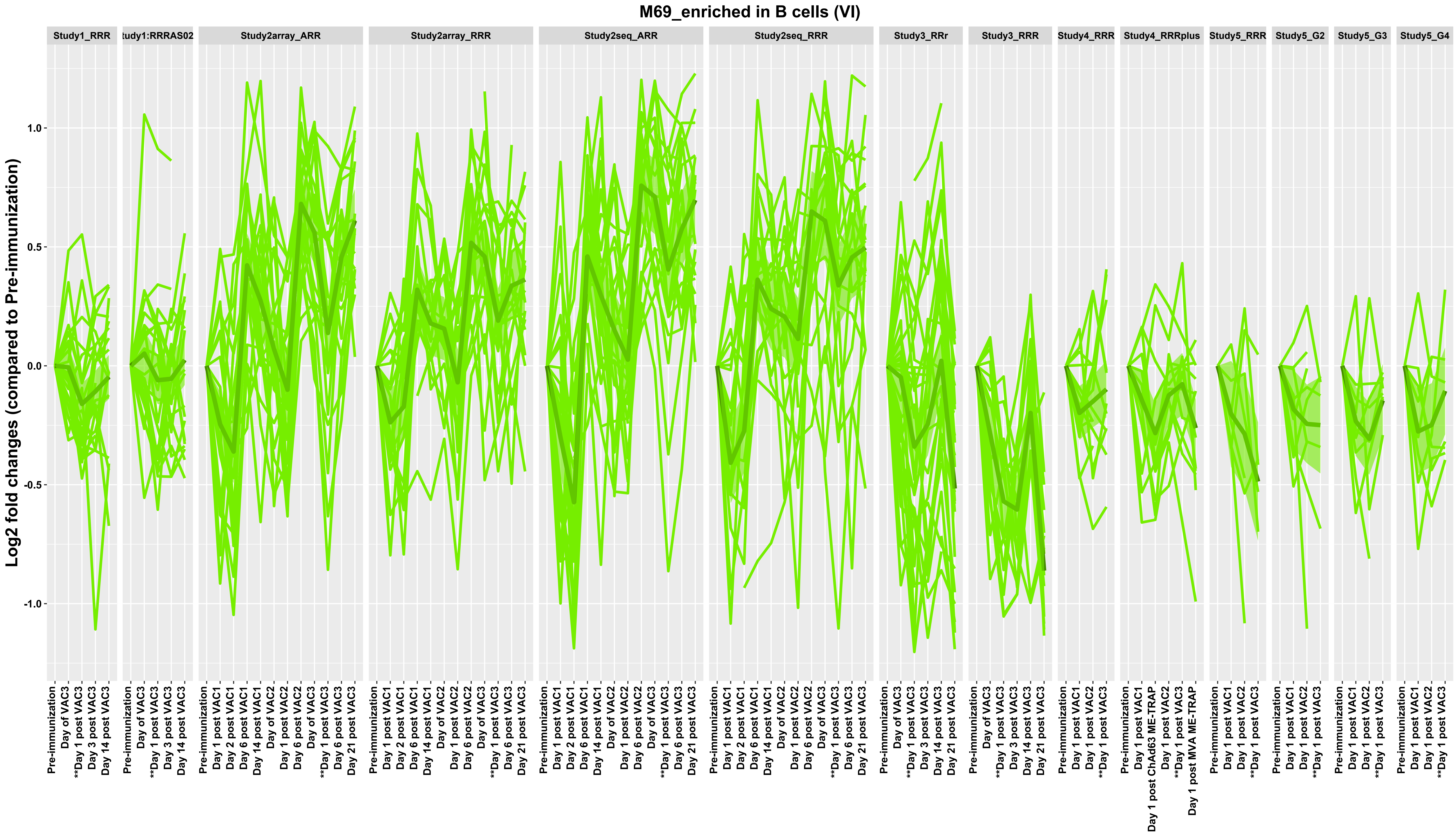
M69_enriched in B cells (VI)
Log2 fold changes (compared to Pre-immunization)
1.0
0.5
0.0
-0.5
-1.0
Study1_RRR
tudy1:RRRAS02
Study2array_ARR
Study2array_RRR
Study2seq_ARR
Study2seq_RRR
Study3_RRr
Study3_RRR
Study4_RRR
Study4_RRRplus
Study5_RRR
Study5_G2
Study5_G3
Study5_G4
Pre-immunization
Day of VAC3
**Day 1 post VAC3
Day 3 post VAC3
Day 14 post VAC3
Day 1 post VAC1
Day 2 post VAC1
Day 6 post VAC1
Day 14 post VAC1
Day of VAC2
Day 1 post VAC2
Day 6 post VAC2
Day 6 post VAC3
Day 21 post VAC3
Day 1 post ChAd63 ME-TRAP
Day 1 post MVA ME-TRAP

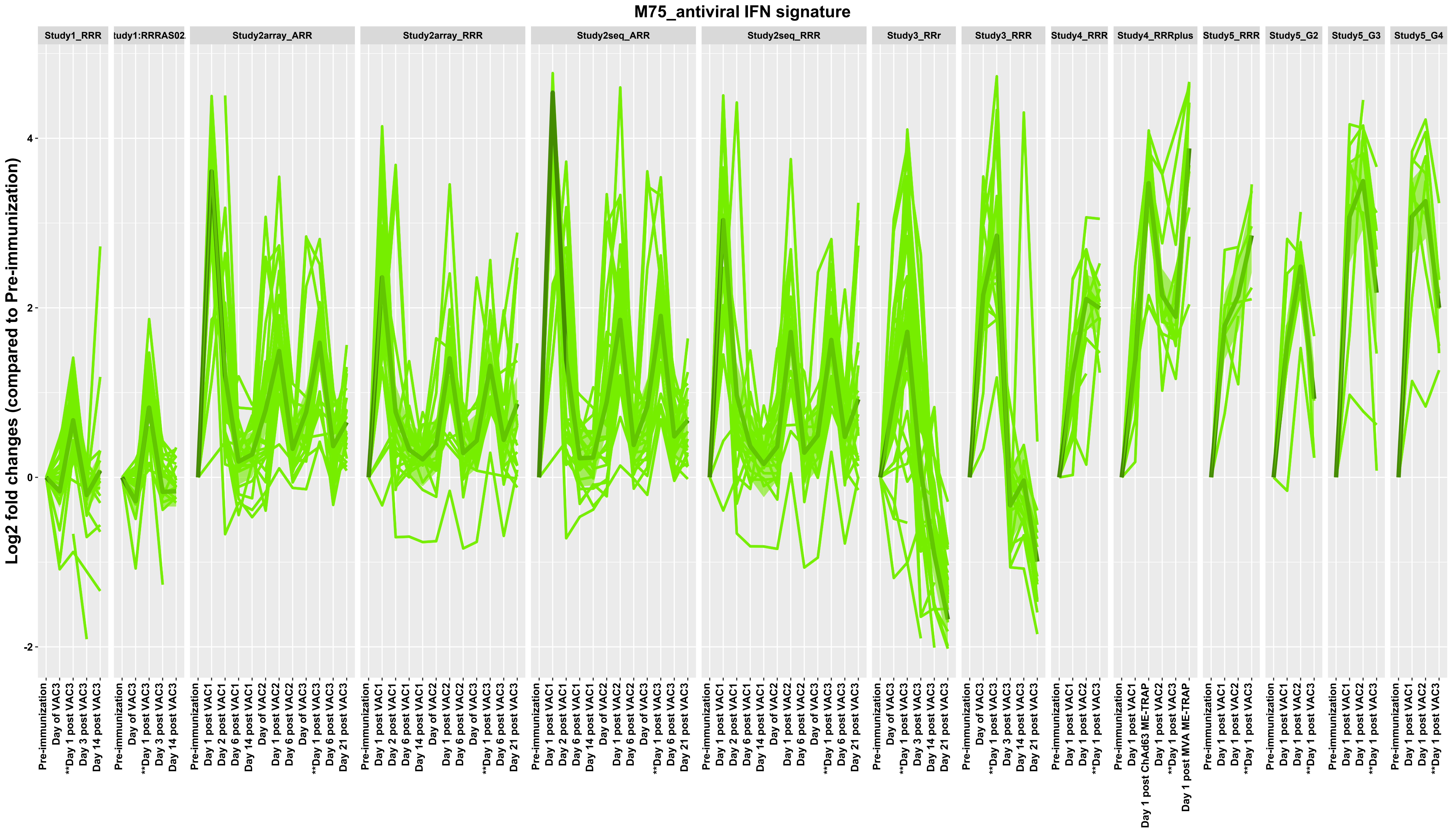
M75_antiviral IFN signature
Study1_RRR
tudy1:RRRAS02
Study2array_ARR
Study2array_RRR
Study2seq_ARR
Study2seq_RRR
Study3_RRr
Study3_RRR
Study4_RRR
Study4_RRRplus
Study5_RRR
Study5_G2
Study5_G3
Study5_G4
Log2 fold changes (compared to Pre-immunization)
4
2
0
-2
Pre-immunization
Day of VAC3
**Day 1 post VAC3
Day 3 post VAC3
Day 14 post VAC3
Day 1 post VAC1
Day 2 post VAC1
Day 6 post VAC1
Day 14 post VAC1
Day of VAC2
Day 1 post VAC2
Day 6 post VAC2
Day 6 post VAC3
Day 21 post VAC3
Day 1 post ChAd63 ME-TRAP
**Day 1 post VAC3
Day 1 post MVA ME-TRAP

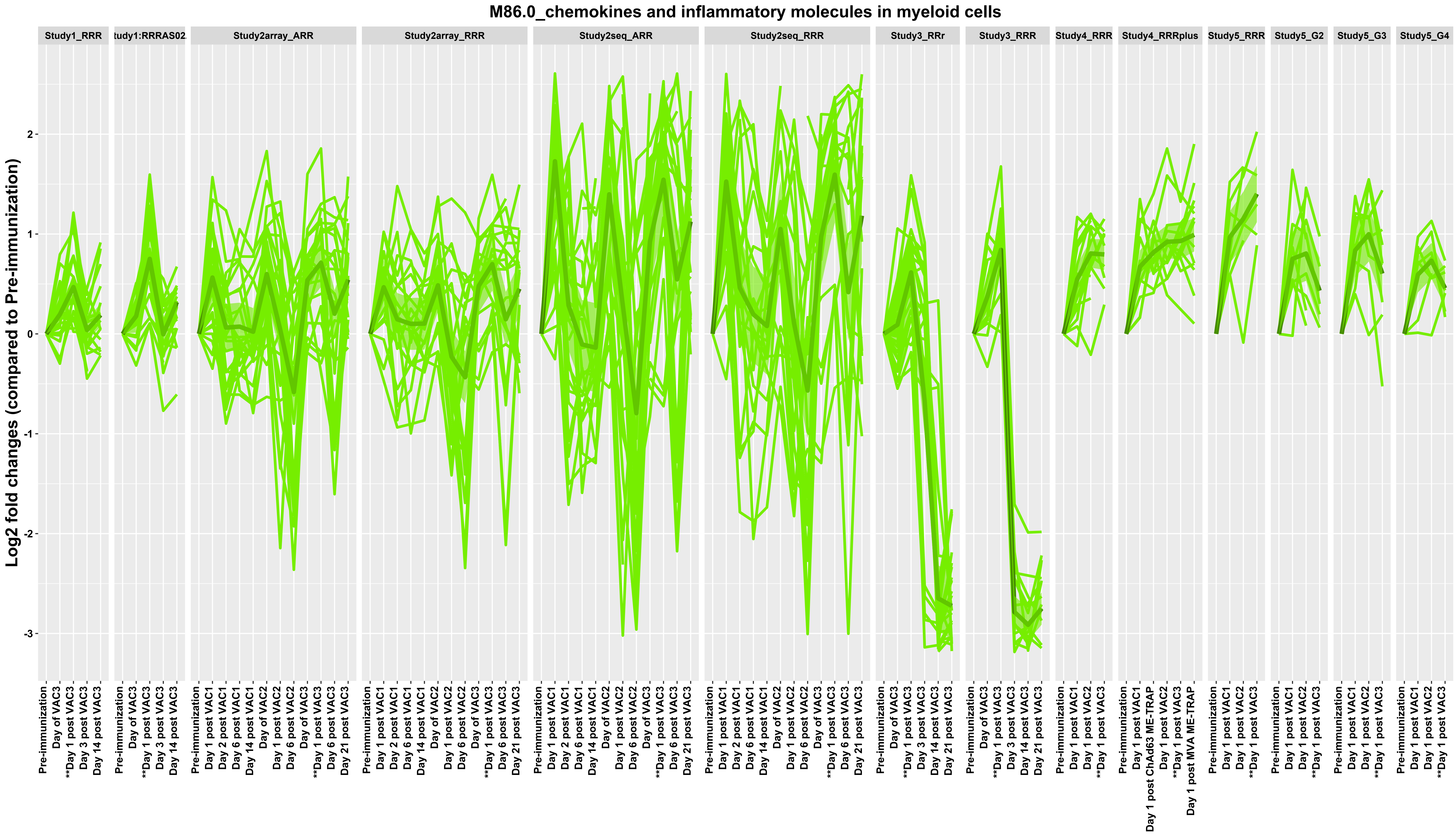

M86.0_chemokines and inflammatory molecules in myeloid cells
Study1_RRR
tudy1:RRRAS02
Study2array_ARR
Study2array_RRR
Study2seq_ARR
Study2seq_RRR
Study3_RRr
Study3_RRR
Study4_RRR
Study4_RRRplus
Study5_RRR
Study5_G2
Study5_G3
Study5_G4
Log2 fold changes (compared to Pre-immunization)
2
1
0
-1
-2
-3
Pre-immunization
Day of VAC3
**Day 1 post VAC3
Day 3 post VAC3
Day 14 post VAC3
Day 1 post VAC1
Day 2 post VAC1
Day 6 post VAC1
Day 14 post VAC1
Day of VAC2
Day 1 post VAC2
Day 6 post VAC2
Day 6 post VAC3
Day 21 post VAC3
**Day 1 post VAC1
**Day 1 post VAC2
Day 1 post ChAd63 ME-TRAP
Day 1 post MVA ME-TRAP

M111.0_viral sensing & immunity; IRF2 targets network (I)

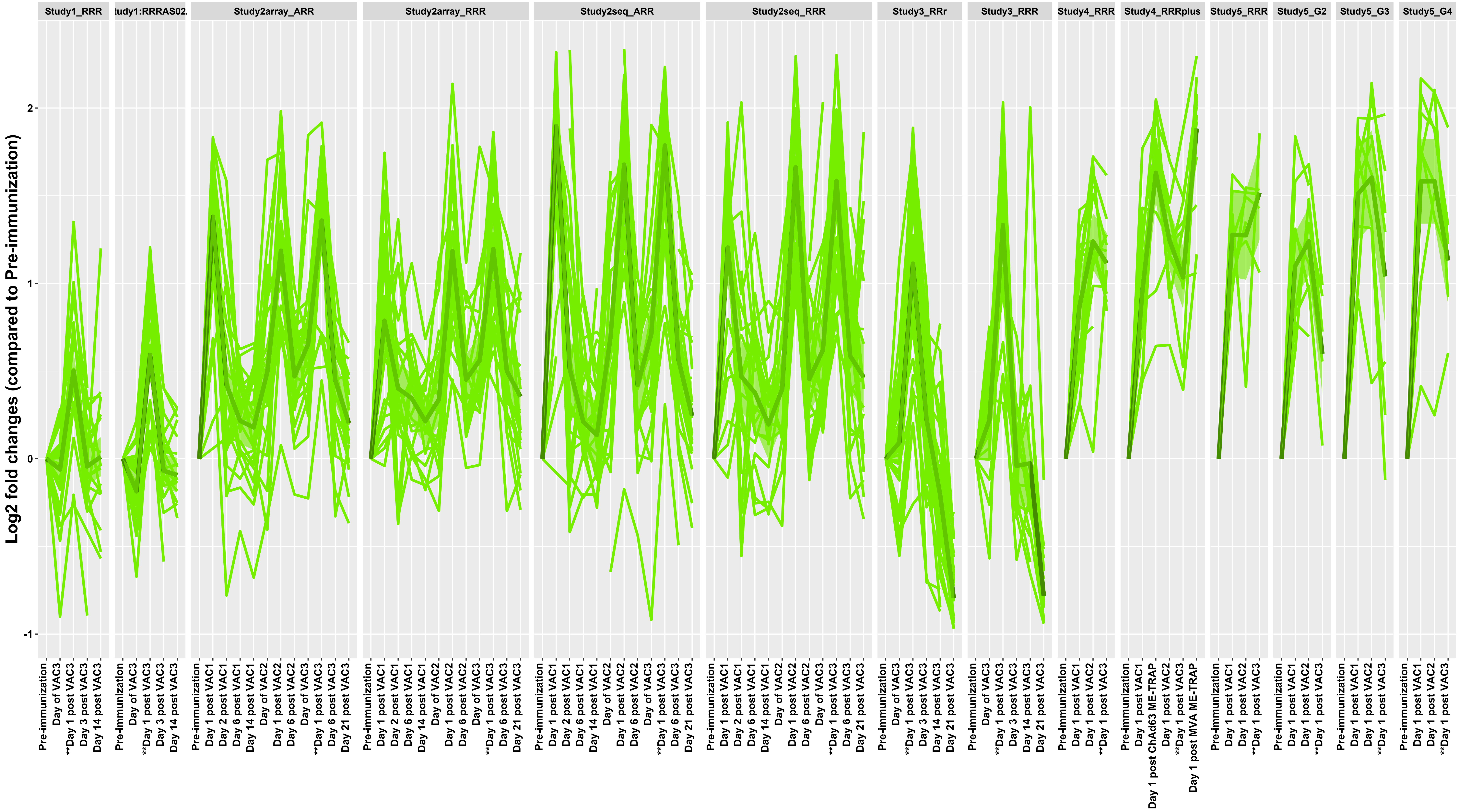

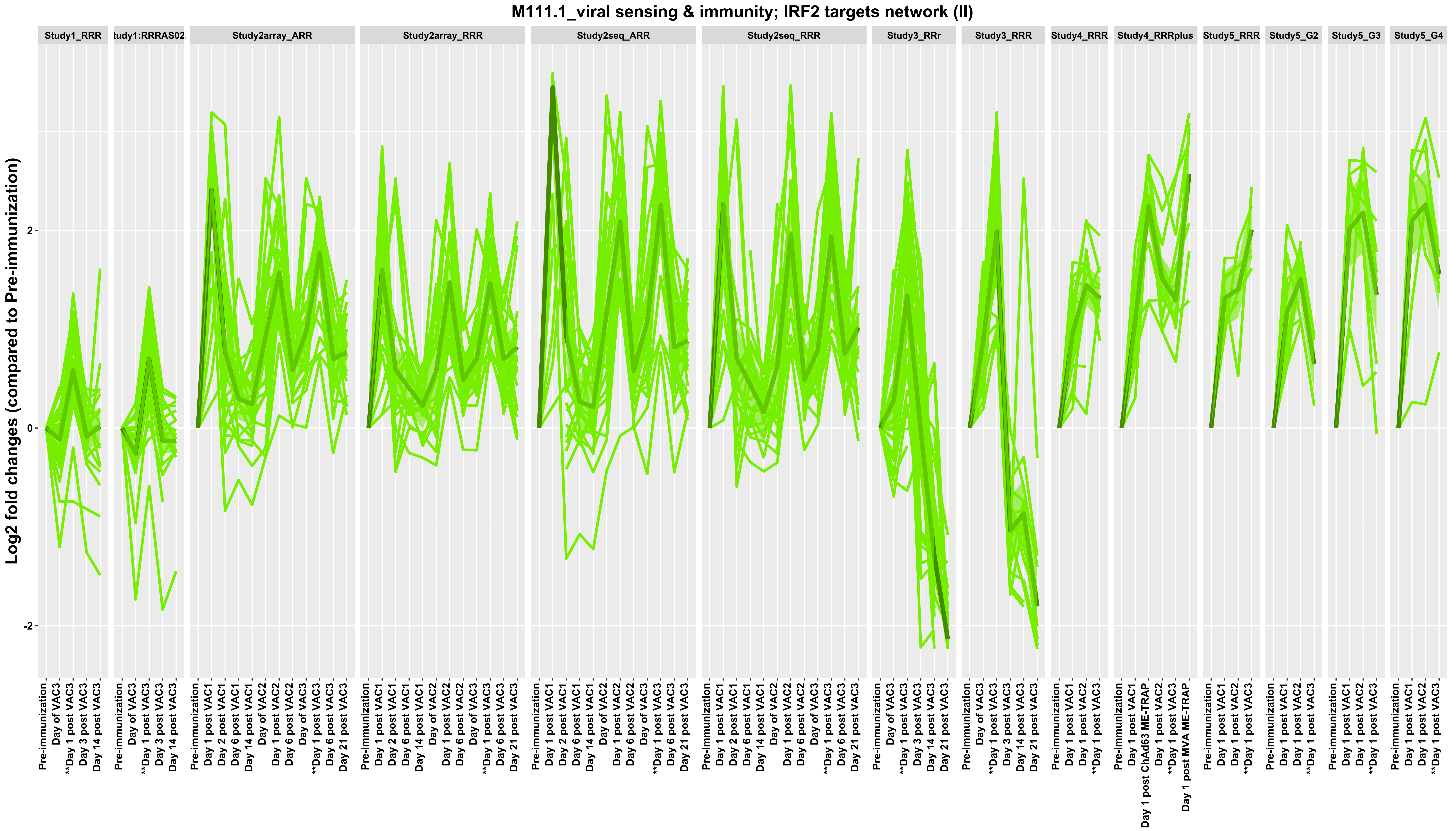

M111.1_viral sensing & immunity; IRF2 targets network (II)
Study1_RRR
tudy1:RRRAS02
Study2array_ARR
Study2array_RRR
Study2seq_ARR
Study2seq_RRR
Study3_RRr
Study3_RRR
Study4_RRR
Study4_RRRplus
Study5_RRR
Study5_G2
Study5_G3
Study5_G4
Log2 fold changes (compared to Pre-immunization)
2
0
-2
Pre-immunization
Day of VAC3
**Day 1 post VAC3
Day 3 post VAC3
Day 14 post VAC3
Day 1 post VAC1
Day 2 post VAC1
Day 6 post VAC1
Day 14 post VAC1
Day of VAC2
Day 1 post VAC2
Day 6 post VAC2
Day 6 post VAC3
Day 21 post VAC3
Day 1 post ChAd63 ME-TRAP
Day 1 post MVA ME-TRAP

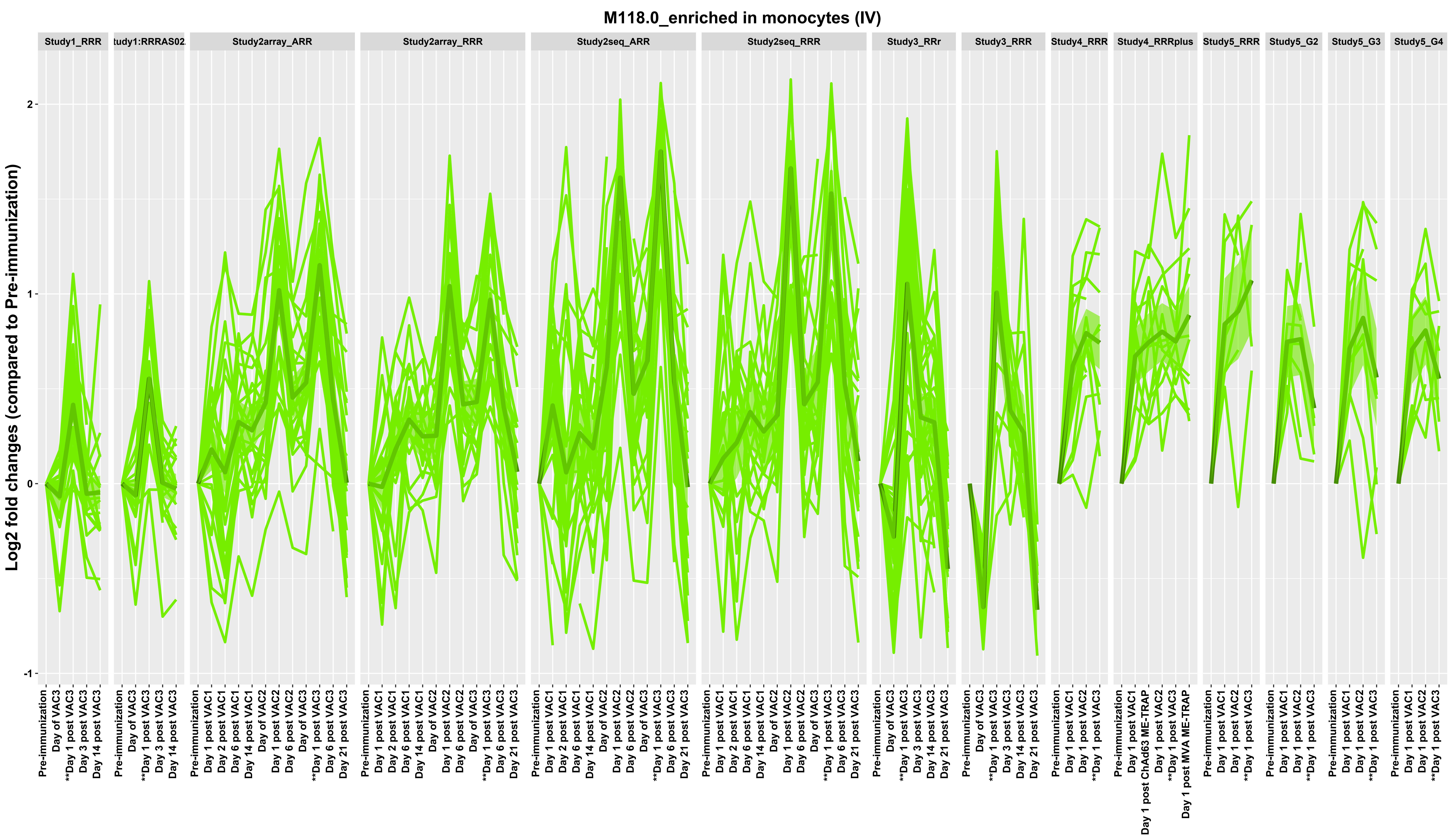
M118.0_enriched in monocytes (IV)
Log2 fold changes (compared to Pre-immunization)
Study1_RRR
tudy1:RRRAS02
Study2array_ARR
Study2array_RRR
Study2seq_ARR
Study2seq_RRR
Study3_RRr
Study3_RRR
Study4_RRR
Study4_RRRplus
Study5_RRR
Study5_G2
Study5_G3
Study5_G4
2
1
0
-1
Pre-immunization
Day of VAC3
**Day 1 post VAC3
Day 3 post VAC3
Day 14 post VAC3
Day 1 post VAC1
Day 2 post VAC1
Day 6 post VAC1
Day 14 post VAC1
Day of VAC2
Day 1 post VAC2
Day 6 post VAC2
Day 6 post VAC3
Day 21 post VAC3
Day 1 post ChAd63 ME-TRAP
Day 1 post MVA ME-TRAP

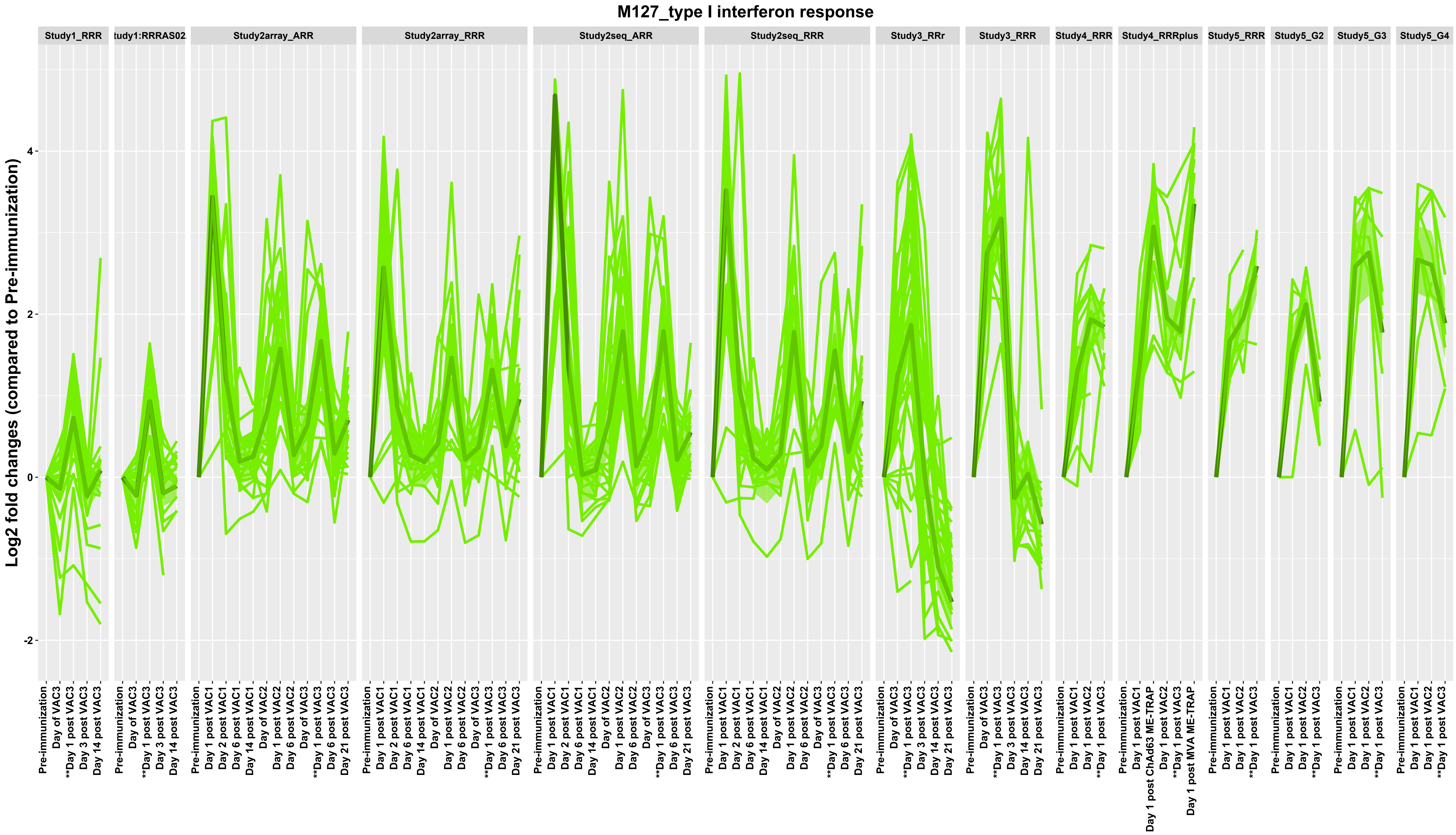
M127_type I interferon response
Log2 fold changes (compared to Pre-immunization)
Study1_RRR
tudy1:RRRAS02
Study2array_ARR
Study2array_RRR
Study2seq_ARR
Study2seq_RRR
Study3_RRr
Study3_RRR
Study4_RRR
Study4_RRRplus
Study5_RRR
Study5_G2
Study5_G3
Study5_G4
4
2
0
-2
Pre-immunization
Day of VAC3
**Day 1 post VAC3
Day 3 post VAC3
Day 14 post VAC3
Day 1 post VAC1
Day 2 post VAC1
Day 6 post VAC1
Day 14 post VAC1
Day of VAC2
Day 1 post VAC2
Day 6 post VAC2
Day 6 post VAC3
Day 21 post VAC3
Day 1 post ChAd63 ME-TRAP
Day 1 post MVA ME-TRAP

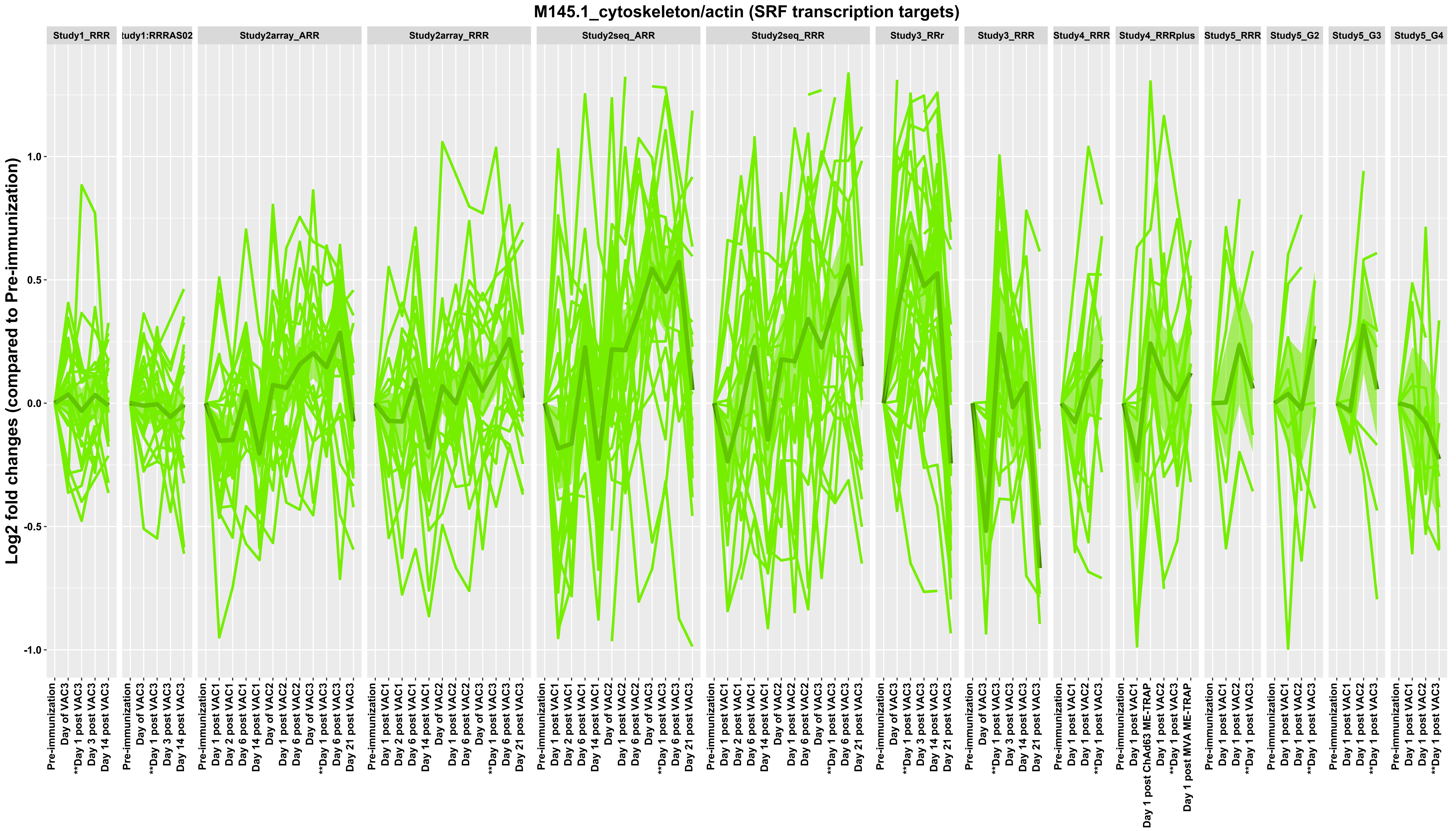
M145.1_cytoskeleton/actin (SRF transcription targets)
Study1_RRR
tudy1:RRRAS02
Study2array_ARR
Study2array_RRR
Study2seq_ARR
Study2seq_RRR
Study3_RRr
Study3_RRR
Study4_RRR
Study4_RRRplus
Study5_RRR
Study5_G2
Study5_G3
Study5_G4
Log2 fold changes (compared to Pre-immunization)
1.0
0.5
0.0
-0.5
-1.0
Pre-immunization
Day of VAC3
**Day 1 post VAC3
Day 3 post VAC3
Day 14 post VAC3
Day 1 post VAC1
Day 2 post VAC1
Day 6 post VAC1
Day 14 post VAC1
Day of VAC2
Day 1 post VAC2
Day 6 post VAC2
Day 6 post VAC3
Day 21 post VAC3
Day 1 post ChAd63 ME-TRAP
Day 1 post MVA ME-TRAP
**Day 1 post VAC3
Day 1 post VAC2

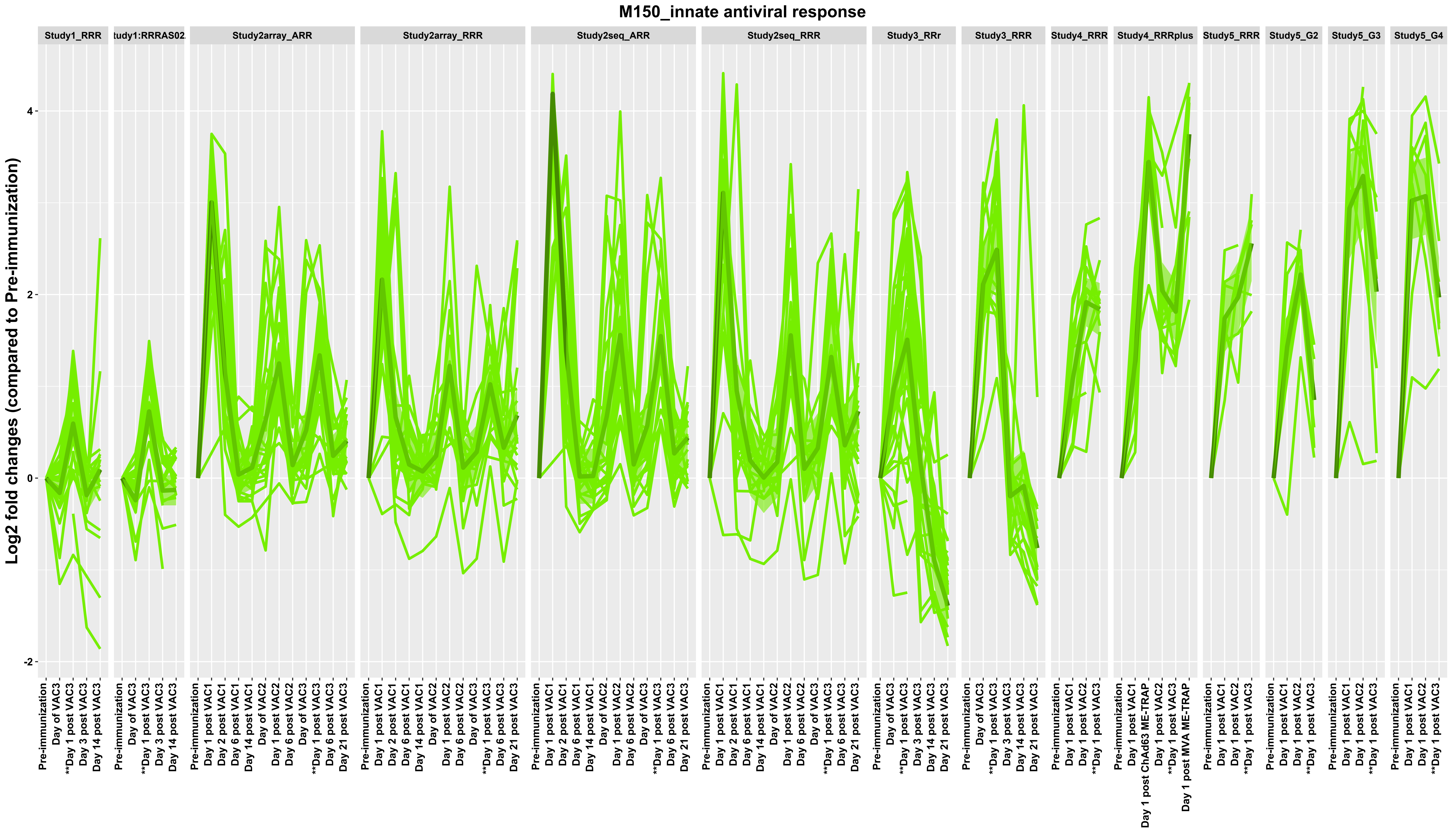

M150_innate antiviral response
Study1_RRR
tudy1:RRRAS02
Study2array_ARR
Study2array_RRR
Study2seq_ARR
Study2seq_RRR
Study3_RRr
Study3_RRR
Study4_RRR
Study4_RRRplus
Study5_RRR
Study5_G2
Study5_G3
Study5_G4
Log2 fold changes (compared to Pre-immunization)
4
2
0
-2
Pre-immunization
Day of VAC3
**Day 1 post VAC3
Day 3 post VAC3
Day 14 post VAC3
Day 1 post VAC1
Day 2 post VAC1
Day 6 post VAC1
Day 14 post VAC1
Day of VAC2
Day 1 post VAC2
Day 6 post VAC2
Day 6 post VAC3
Day 21 post VAC3
Day 21 post VAC3
Day 1 post ChAd63 ME-TRAP
Day 1 post MVA ME-TRAP
**Day 1 post VAC3

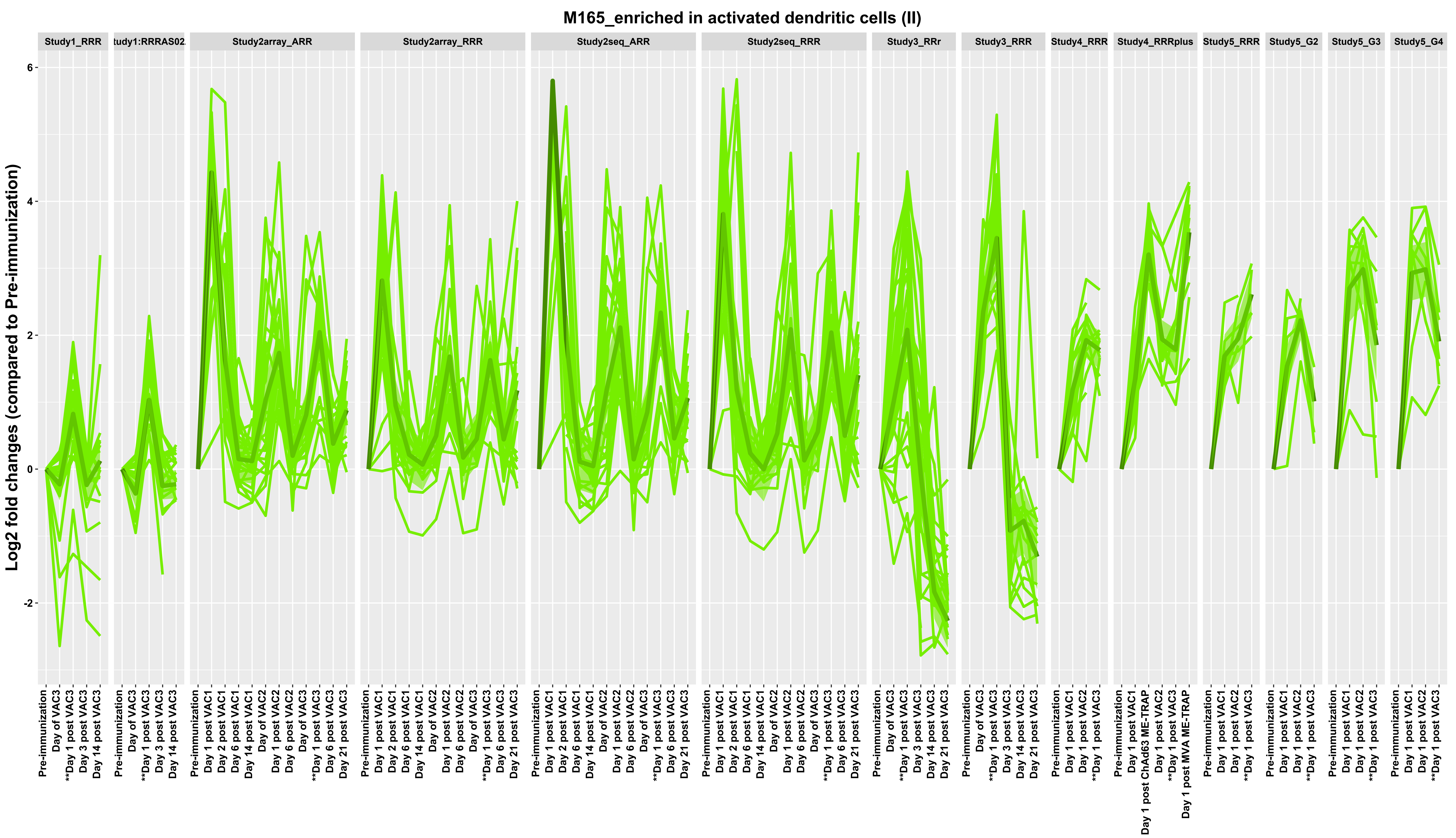

M165_enriched in activated dendritic cells (II)
Log2 fold changes (compared to Pre-immunization)
Study1_RRR
tudy1:RRRAS02
Study2array_ARR
Study2array_RRR
Study2seq_ARR
Study2seq_RRR
Study3_RRr
Study3_RRR
Study4_RRR
Study4_RRRplus
Study5_RRR
Study5_G2
Study5_G3
Study5_G4
6
4
2
0
-2
Pre-immunization
Day of VAC3
**Day 1 post VAC3
Day 3 post VAC3
Day 14 post VAC3
Day 1 post VAC1
Day 2 post VAC1
Day 6 post VAC1
Day 14 post VAC1
Day of VAC2
Day 1 post VAC2
Day 6 post VAC2
Day 6 post VAC3
Day 21 post VAC3
Day 1 post ChAd63 ME-TRAP
**Day 1 post VAC3
Day 1 post MVA ME-TRAP
**Day 1 post VAC2

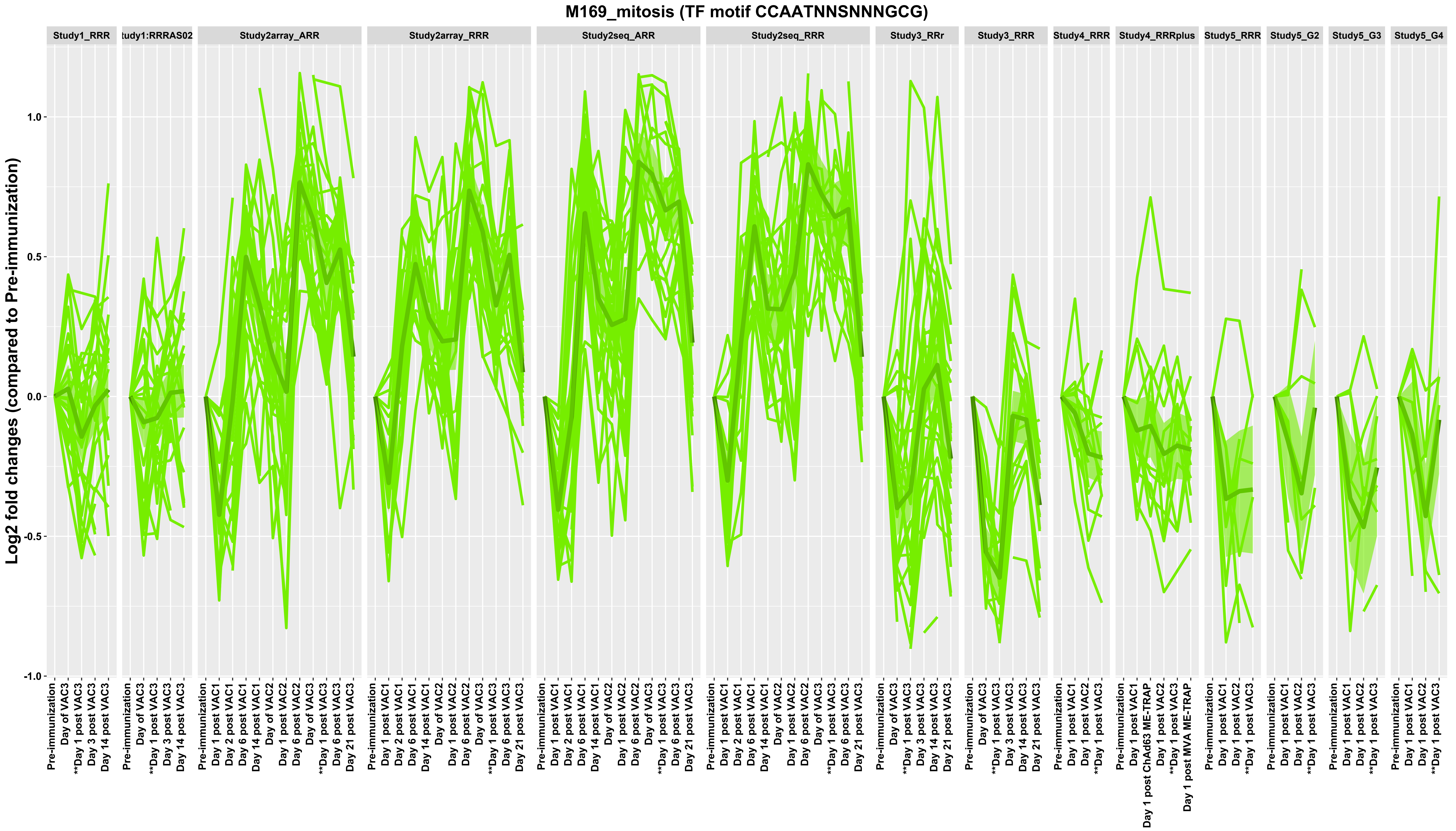
M169_mitosis (TF motif CCAATNNSNNNGCG)
Study1_RRR
tudy1:RRRAS02
Study2array_ARR
Study2array_RRR
Study2seq_ARR
Study2seq_RRR
Study3_RRr
Study3_RRR
Study4_RRR
Study4_RRRplus
Study5_RRR
Study5_G2
Study5_G3
Study5_G4
Log2 fold changes (compared to Pre-immunization)
1.0
0.5
0.0
-0.5
-1.0
Pre-immunization
Day of VAC3
**Day 1 post VAC3
Day 3 post VAC3
Day 14 post VAC3
Day 1 post VAC1
Day 2 post VAC1
Day 6 post VAC1
Day 14 post VAC1
Day of VAC2
Day 1 post VAC2
Day 6 post VAC2
Day 6 post VAC3
Day 21 post VAC3
Day 1 post ChAd63 ME-TRAP
Day 1 post MVA ME-TRAP
**Day 1 post VAC3

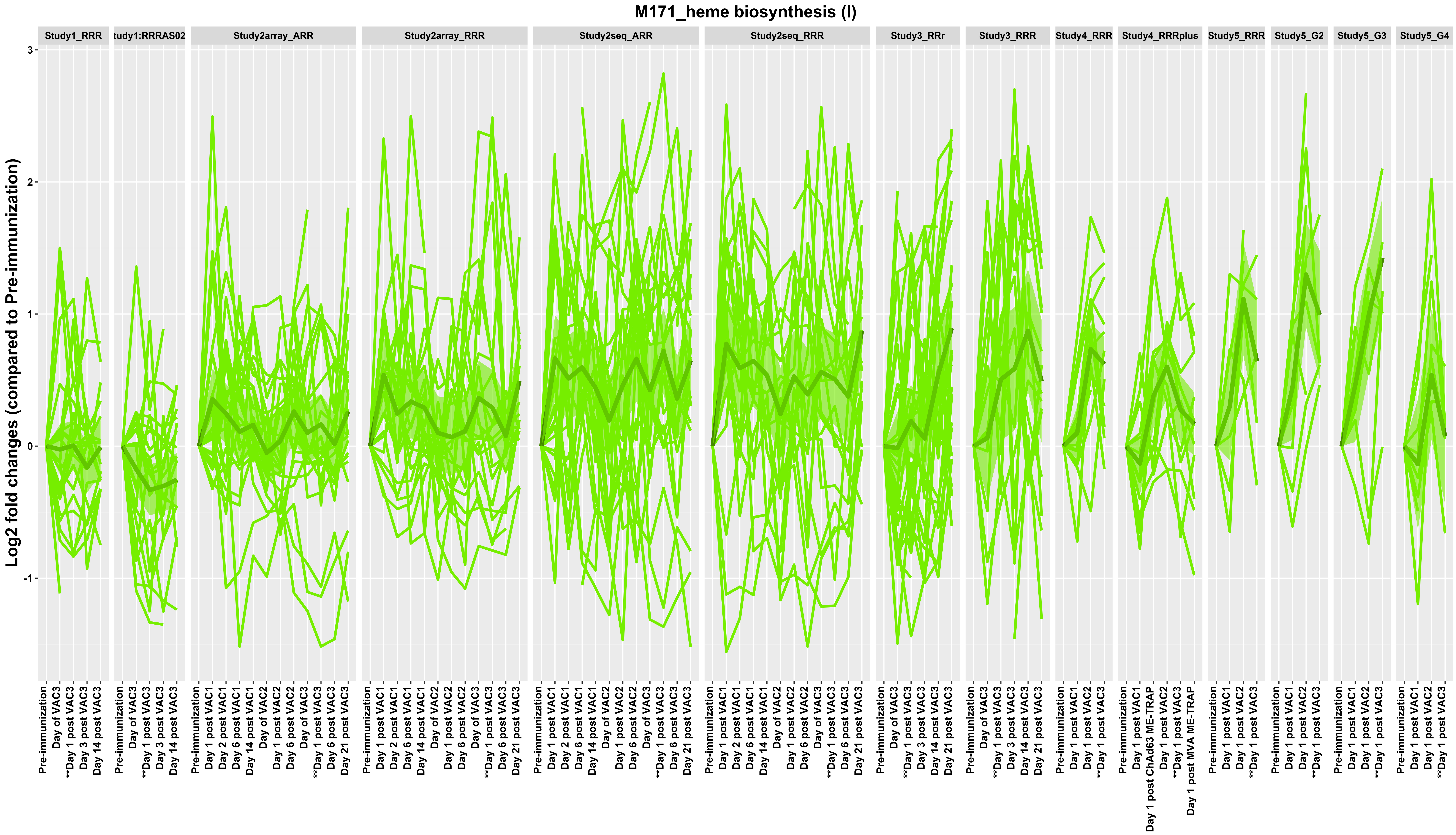

M171_heme biosynthesis (I)
Study1_RRR
tudy1:RRRAS02
Study2array_ARR
Study2array_RRR
Study2seq_ARR
Study2seq_RRR
Study3_RRr
Study3_RRR
Study4_RRR
Study4_RRRplus
Study5_RRR
Study5_G2
Study5_G3
Study5_G4
Log2 fold changes (compared to Pre-immunization)
3
2
1
0
-1
Pre-immunization
Day of VAC3
**Day 1 post VAC3
Day 3 post VAC3
Day 14 post VAC3
Day 1 post VAC1
Day 2 post VAC1
Day 6 post VAC1
Day 14 post VAC1
Day of VAC2
Day 1 post VAC2
Day 6 post VAC2
Day 6 post VAC3
Day 21 post VAC3
Day 1 post ChAd63 ME-TRAP
Day 1 post MVA ME-TRAP

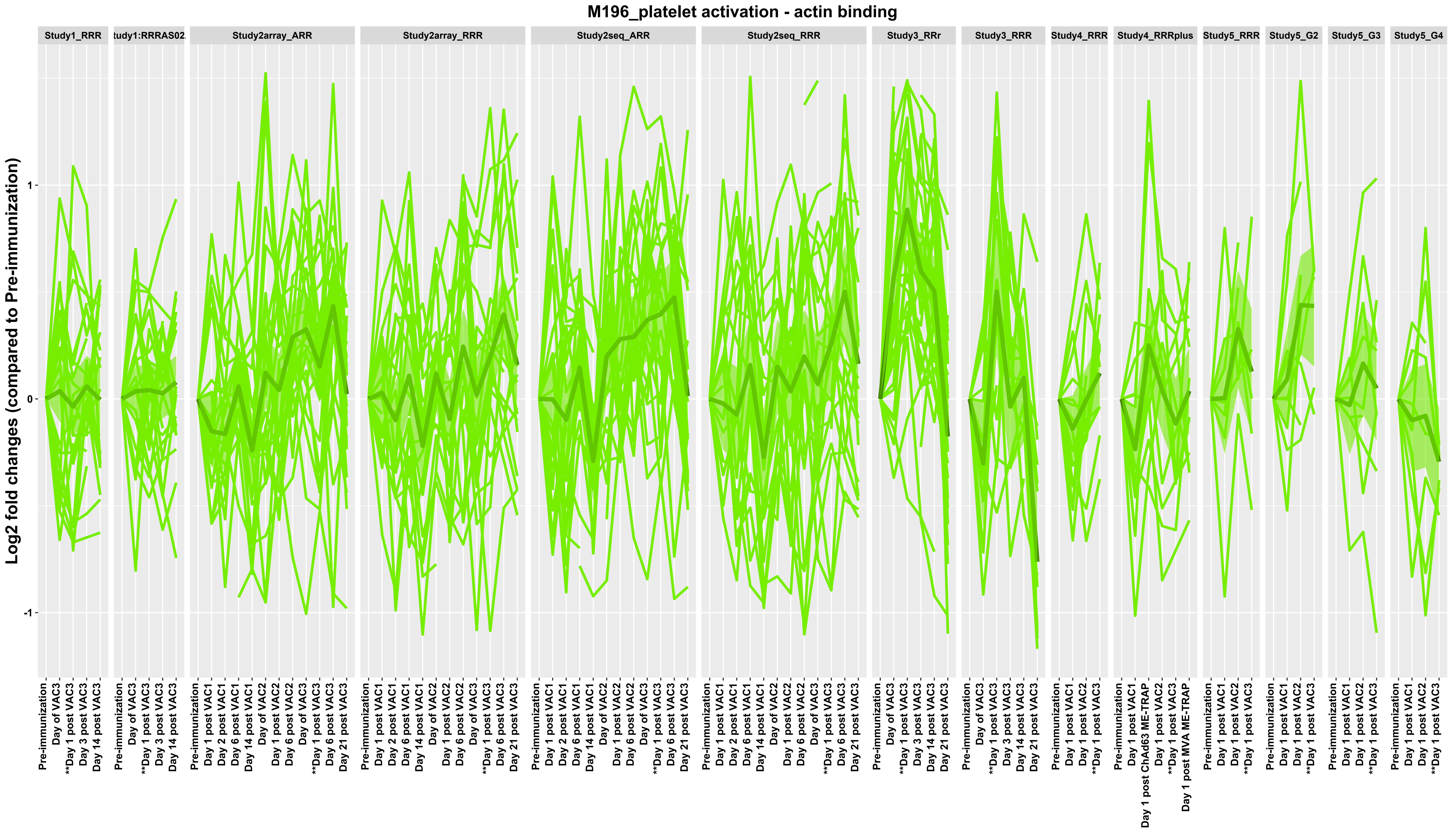
M196_platelet activation - actin binding
Log2 fold changes (compared to Pre-immunization)
Study1_RRR
tudy1:RRRAS02
Study2array_ARR
Study2array_RRR
Study2seq_ARR
Study2seq_RRR
Study3_RRr
Study3_RRR
Study4_RRR
Study4_RRRplus
Study5_RRR
Study5_G2
Study5_G3
Study5_G4
1
0
-1
Pre-immunization
Day of VAC3
**Day 1 post VAC3
Day 3 post VAC3
Day 14 post VAC3
Day 1 post VAC1
Day 2 post VAC1
Day 6 post VAC1
Day 14 post VAC1
Day of VAC2
Day 1 post VAC2
Day 6 post VAC2
Day 6 post VAC3
Day 21 post VAC3
Day 1 post ChAd63 ME-TRAP
Day 1 post MVA ME-TRAP

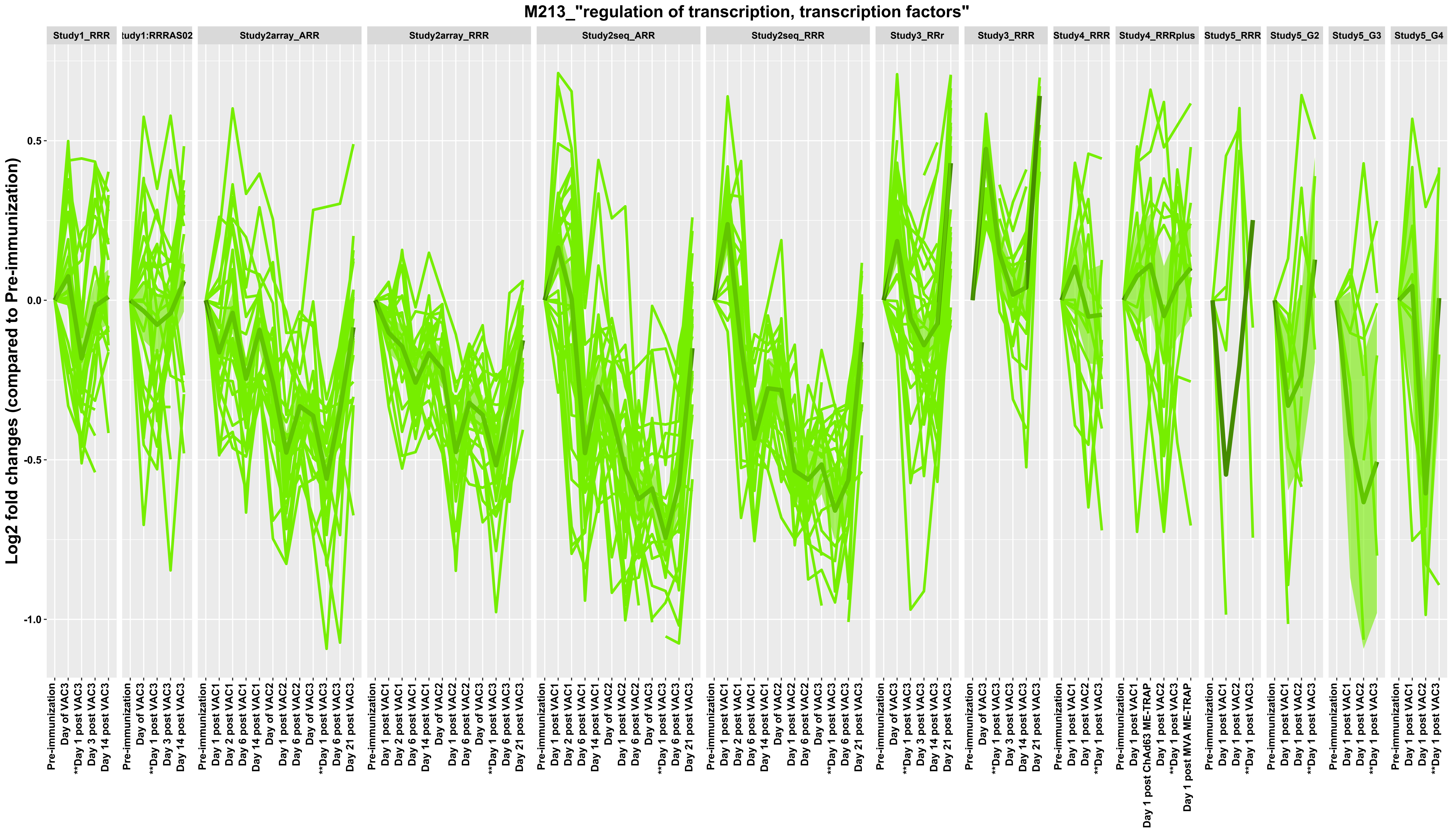

M213_"regulation of transcription, transcription factors"
Log2 fold changes (compared to Pre-immunization)
0.5
0.0
-0.5
-1.0
Study1_RRR
tudy1:RRRAS02
Study2array_ARR
Study2array_RRR
Study2seq_ARR
Study2seq_RRR
Study3_RRr
Study3_RRR
Study4_RRR
Study4_RRRplus
Study5_RRR
Study5_G2
Study5_G3
Study5_G4
Pre-immunization
Day of VAC3
**Day 1 post VAC3
Day 3 post VAC3
Day 14 post VAC3
Day 1 post VAC1
Day 2 post VAC1
Day 6 post VAC1
Day 14 post VAC1
Day of VAC2
Day 1 post VAC2
Day 6 post VAC2
Day 6 post VAC3
Day 21 post VAC3
Day 1 post ChAd63 ME-TRAP
**Day 1 post VAC3
Day 1 post MVA ME-TRAP

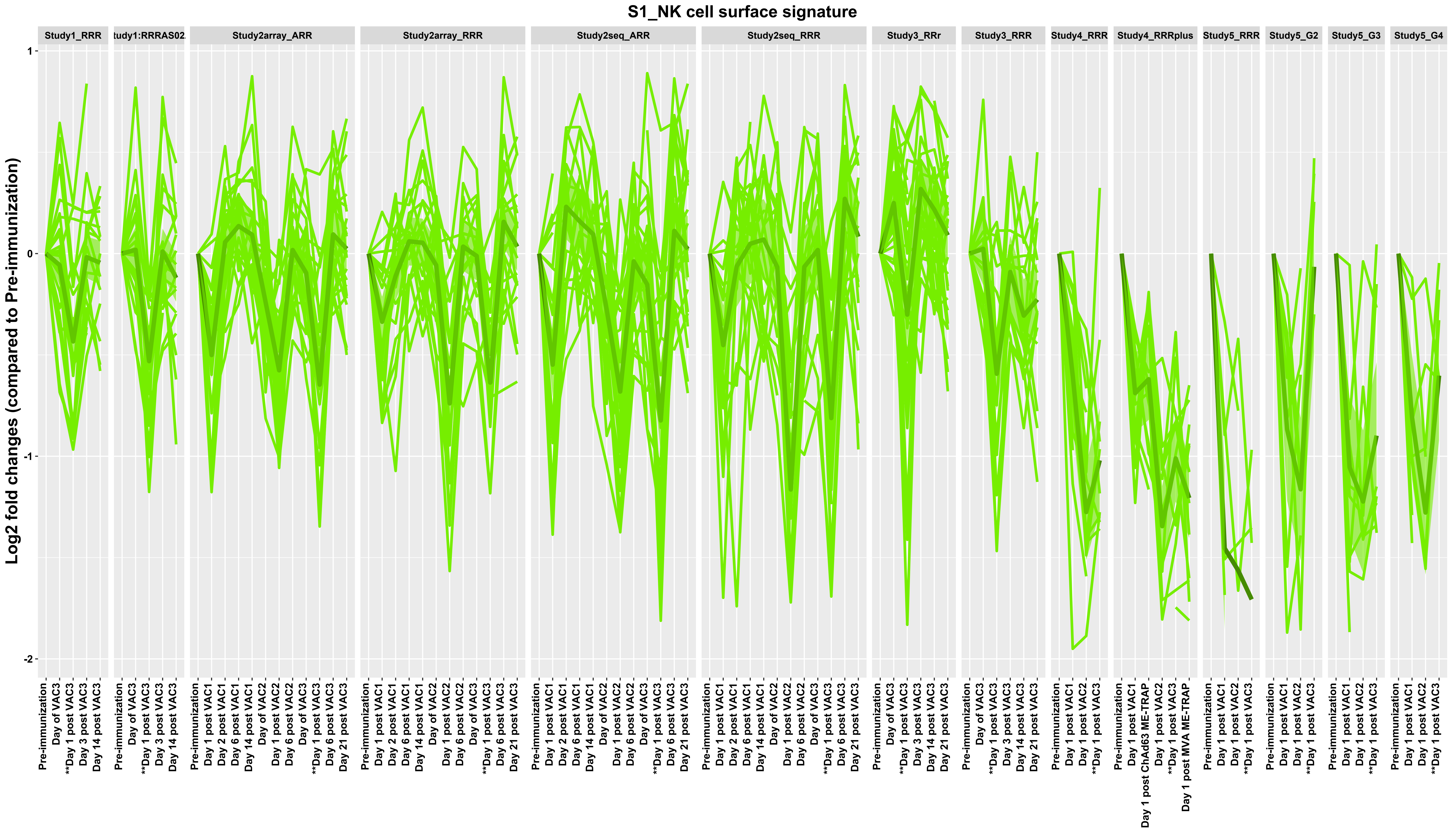
S1_NK cell surface signature
Study1_RRR
tudy1:RRRAS02
Study2array_ARR
Study2array_RRR
Study2seq_ARR
Study2seq_RRR
Study3_RRr
Study3_RRR
Study4_RRR
Study4_RRRplus
Study5_RRR
Study5_G2
Study5_G3
Study5_G4
Log2 fold changes (compared to Pre-immunization)
1
0
-1
-2
Pre-immunization
Day of VAC3
**Day 1 post VAC3
Day 3 post VAC3
Day 14 post VAC3
Day 1 post VAC1
Day 2 post VAC1
Day 6 post VAC1
Day 14 post VAC1
Day of VAC2
Day 1 post VAC2
Day 6 post VAC2
Day 6 post VAC3
Day 21 post VAC3
Day 1 post ChAd63 ME-TRAP
Day 1 post MVA ME-TRAP

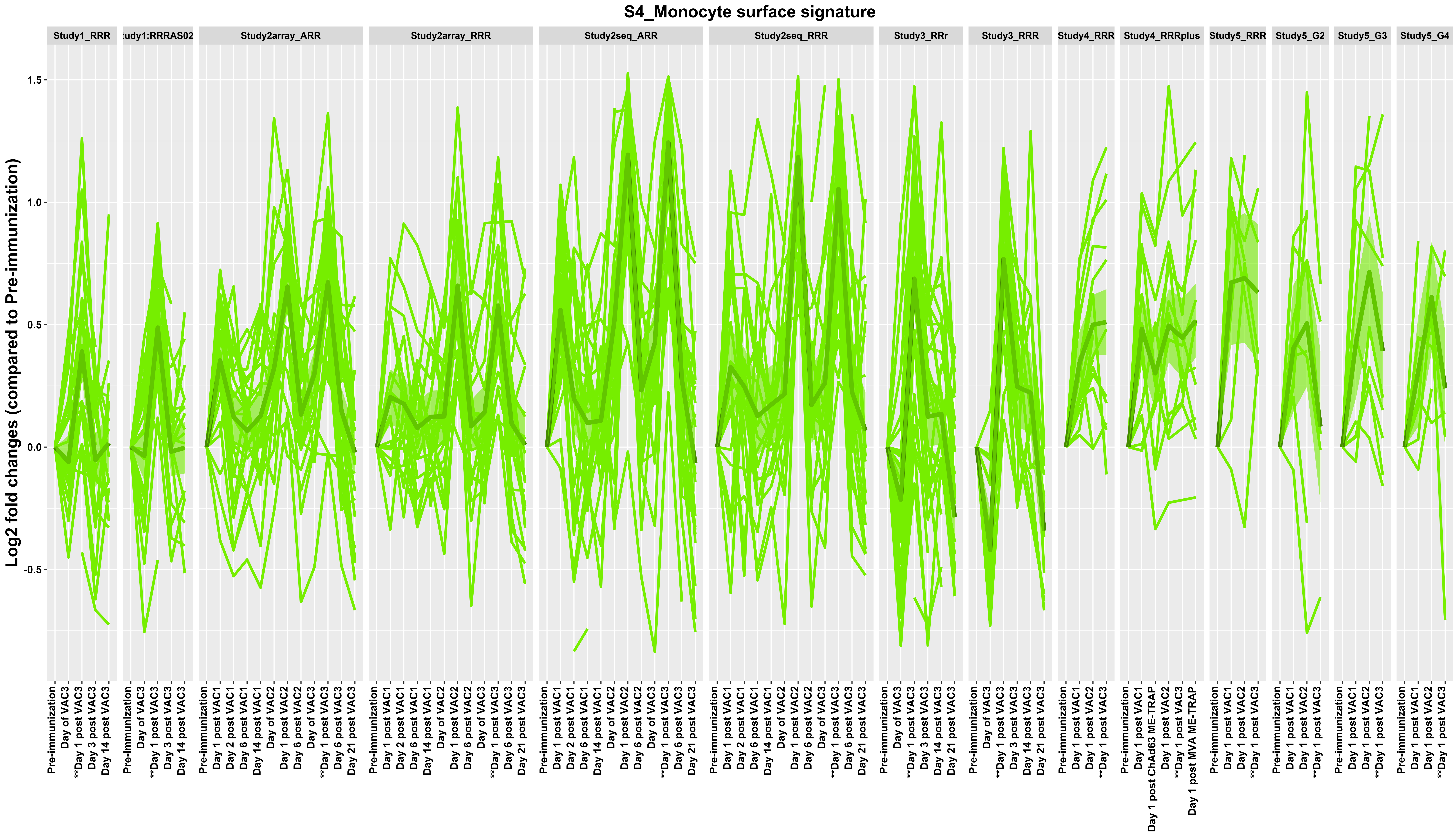
S4_Monocyte surface signature
Study1_RRR
tudy1:RRRAS02
Study2array_ARR
Study2array_RRR
Study2seq_ARR
Study2seq_RRR
Study3_RRr
Study3_RRR
Study4_RRR
Study4_RRRplus
Study5_RRR
Study5_G2
Study5_G3
Study5_G4
Log2 fold changes (compared to Pre-immunization)
1.5
1.0
0.5
0.0
-0.5
Pre-immunization
Day of VAC3
**Day 1 post VAC3
Day 3 post VAC3
Day 14 post VAC3
Day 1 post VAC1
Day 2 post VAC1
Day 6 post VAC1
Day 14 post VAC1
Day of VAC2
Day 1 post VAC2
Day 6 post VAC2
Day 6 post VAC3
Day 21 post VAC3
Day 1 post ChAd63 ME-TRAP
Day 1 post MVA ME-TRAP

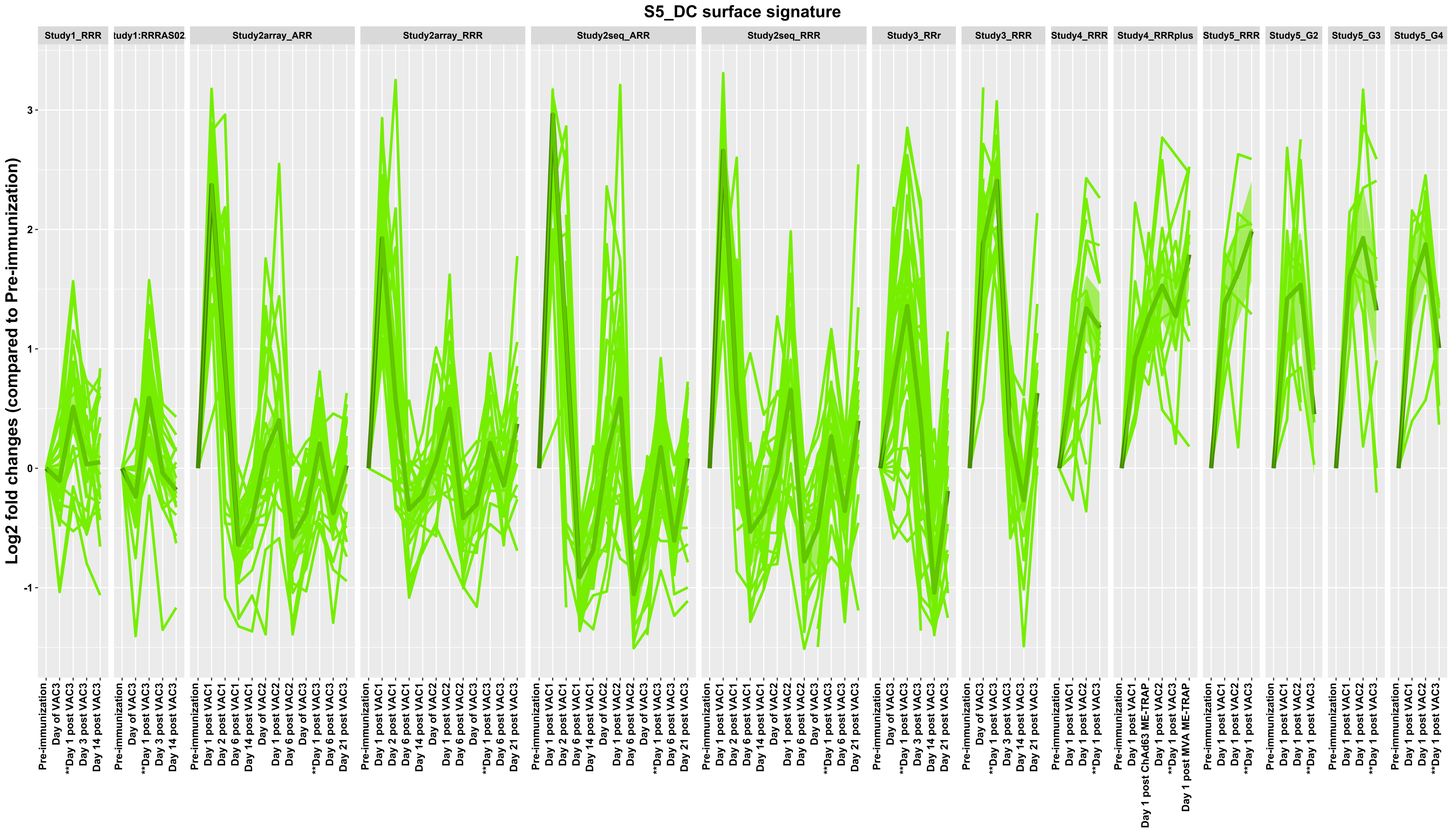
S5_DC surface signature
Study1_RRR
tudy1:RRRAS02
Study2array_ARR
Study2array_RRR
Study2seq_ARR
Study2seq_RRR
Study3_RRr
Study3_RRR
Study4_RRR
Study4_RRRplus
Study5_RRR
Study5_G2
Study5_G3
Study5_G4
Log2 fold changes (compared to Pre-immunization)
3
2
1
0
-1
Pre-immunization
Day of VAC3
**Day 1 post VAC3
Day 3 post VAC3
Day 14 post VAC3
Day 1 post VAC1
Day 2 post VAC1
Day 6 post VAC1
Day 14 post VAC1
Day of VAC2
Day 1 post VAC2
Day 6 post VAC2
Day 6 post VAC3
Day 21 post VAC3
Day 1 post ChAd63 ME-TRAP
Day 1 post MVA ME-TRAP

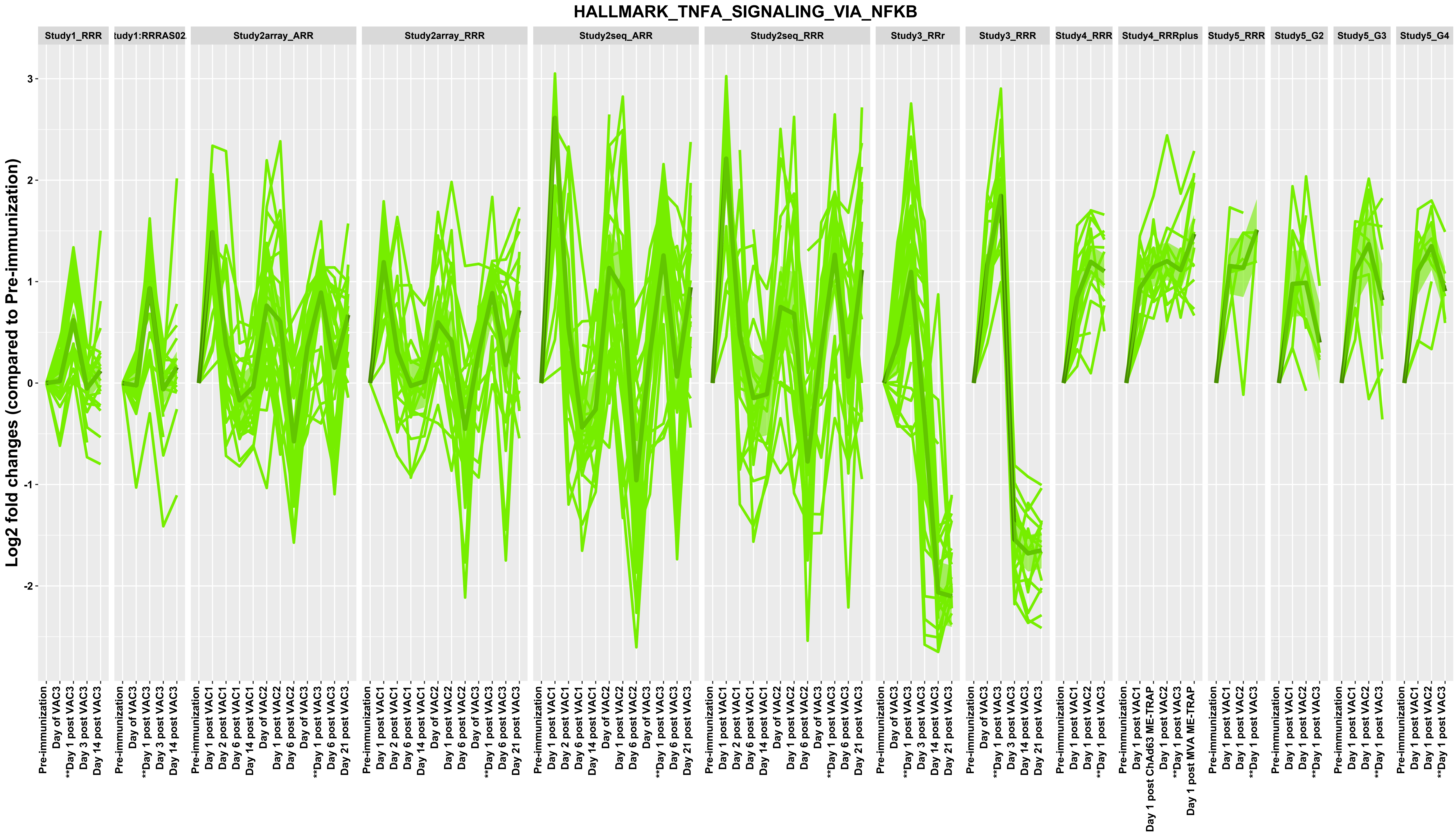
HALLMARK_TNFA_SIGNALING_VIA_NFKB
Study1_RRR
tudy1:RRRAS02
Study2array_ARR
Study2array_RRR
Study2seq_ARR
Study2seq_RRR
Study3_RRr
Study3_RRR
Study4_RRR
Study4_RRRplus
Study5_RRR
Study5_G2
Study5_G3
Study5_G4
Log2 fold changes (compared to Pre-immunization)
3
2
1
0
-1
-2
Pre-immunization
Day of VAC3
**Day 1 post VAC3
Day 3 post VAC3
Day 14 post VAC3
Day 1 post VAC1
Day 2 post VAC1
Day 6 post VAC1
Day 14 post VAC1
Day of VAC2
Day 1 post VAC2
Day 6 post VAC2
Day 6 post VAC3
Day 21 post VAC3
Day 1 post ChAd63 ME-TRAP
Day 1 post MVA ME-TRAP
**Day 1 post VAC2

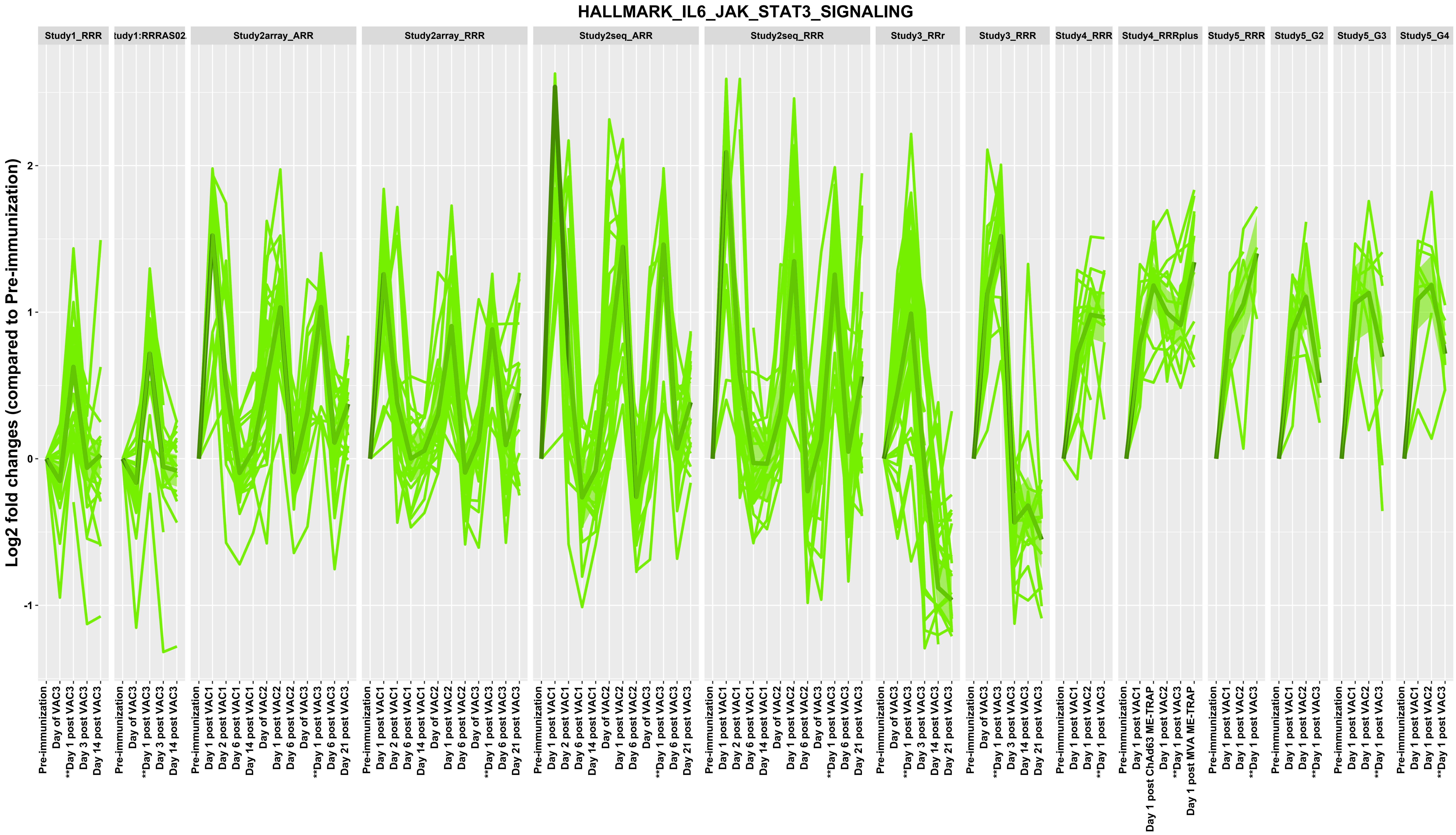
HALLMARK_IL6_JAK_STAT3_SIGNALING
Study1_RRR
tudy1:RRRAS02
Study2array_ARR
Study2array_RRR
Study2seq_ARR
Study2seq_RRR
Study3_RRr
Study3_RRR
Study4_RRR
Study4_RRRplus
Study5_RRR
Study5_G2
Study5_G3
Study5_G4
Log2 fold changes (compared to Pre-immunization)
2
1
0
-1
Pre-immunization
Day of VAC3
**Day 1 post VAC3
Day 3 post VAC3
Day 14 post VAC3
Day 1 post VAC1
Day 2 post VAC1
Day 6 post VAC1
Day 14 post VAC1
Day of VAC2
Day 1 post VAC2
Day 6 post VAC2
Day 6 post VAC3
Day 21 post VAC3
Day 1 post ChAd63 ME-TRAP
Day 1 post MVA ME-TRAP

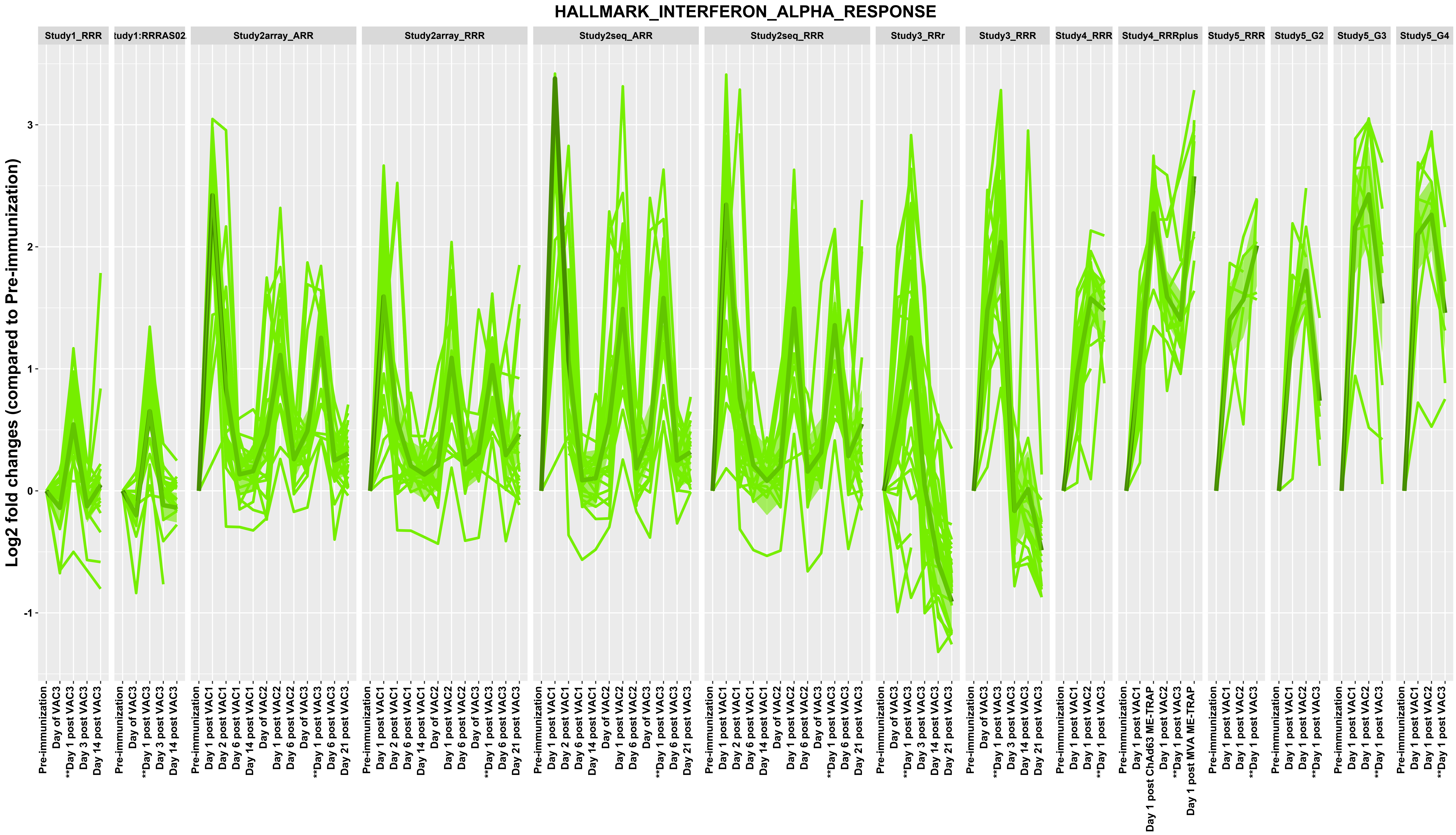
HALLMARK_INTERFERON_ALPHA_RESPONSE
Study1_RRR
tudy1:RRRAS02
Study2array_ARR
Study2array_RRR
Study2seq_ARR
Study2seq_RRR
Study3_RRr
Study3_RRR
Study4_RRR
Study4_RRRplus
Study5_RRR
Study5_G2
Study5_G3
Study5_G4
Log2 fold changes (compared to Pre-immunization)
3
2
1
0
-1
Pre-immunization
Day of VAC3
**Day 1 post VAC3
Day 3 post VAC3
Day 14 post VAC3
Day 1 post VAC1
Day 2 post VAC1
Day 6 post VAC1
Day 14 post VAC1
Day of VAC2
Day 1 post VAC2
Day 6 post VAC2
Day 6 post VAC3
Day 21 post VAC3
Day 1 post ChAd63 ME-TRAP
**Day 1 post VAC3
Day 1 post MVA ME-TRAP
**Day 1 post VAC3

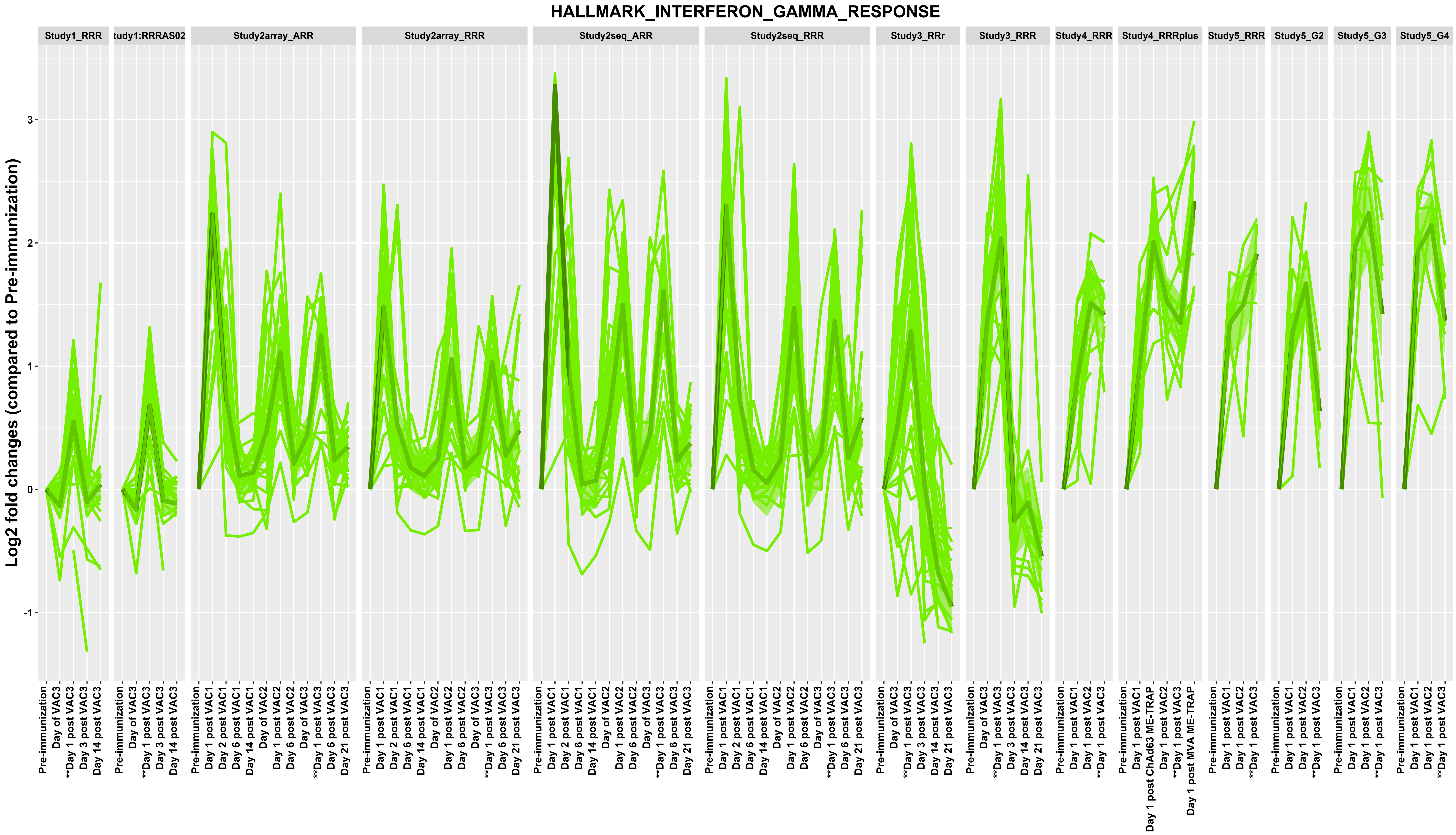
HALLMARK_INTERFERON_GAMMA_RESPONSE
Study1_RRR
tudy1:RRRAS02
Study2array_ARR
Study2array_RRR
Study2seq_ARR
Study2seq_RRR
Study3_RRr
Study3_RRR
Study4_RRR
Study4_RRRplus
Study5_RRR
Study5_G2
Study5_G3
Study5_G4
Log2 fold changes (compared to Pre-immunization)
3
2
1
0
-1
Pre-immunization
Day of VAC3
**Day 1 post VAC3
Day 3 post VAC3
Day 14 post VAC3
Day 1 post VAC1
Day 2 post VAC1
Day 6 post VAC1
Day 14 post VAC1
Day of VAC2
Day 1 post VAC2
Day 6 post VAC2
Day 6 post VAC3
Day 21 post VAC3
**Day 1 post VAC2
Day 1 post ChAd63 ME-TRAP
Day 1 post MVA ME-TRAP

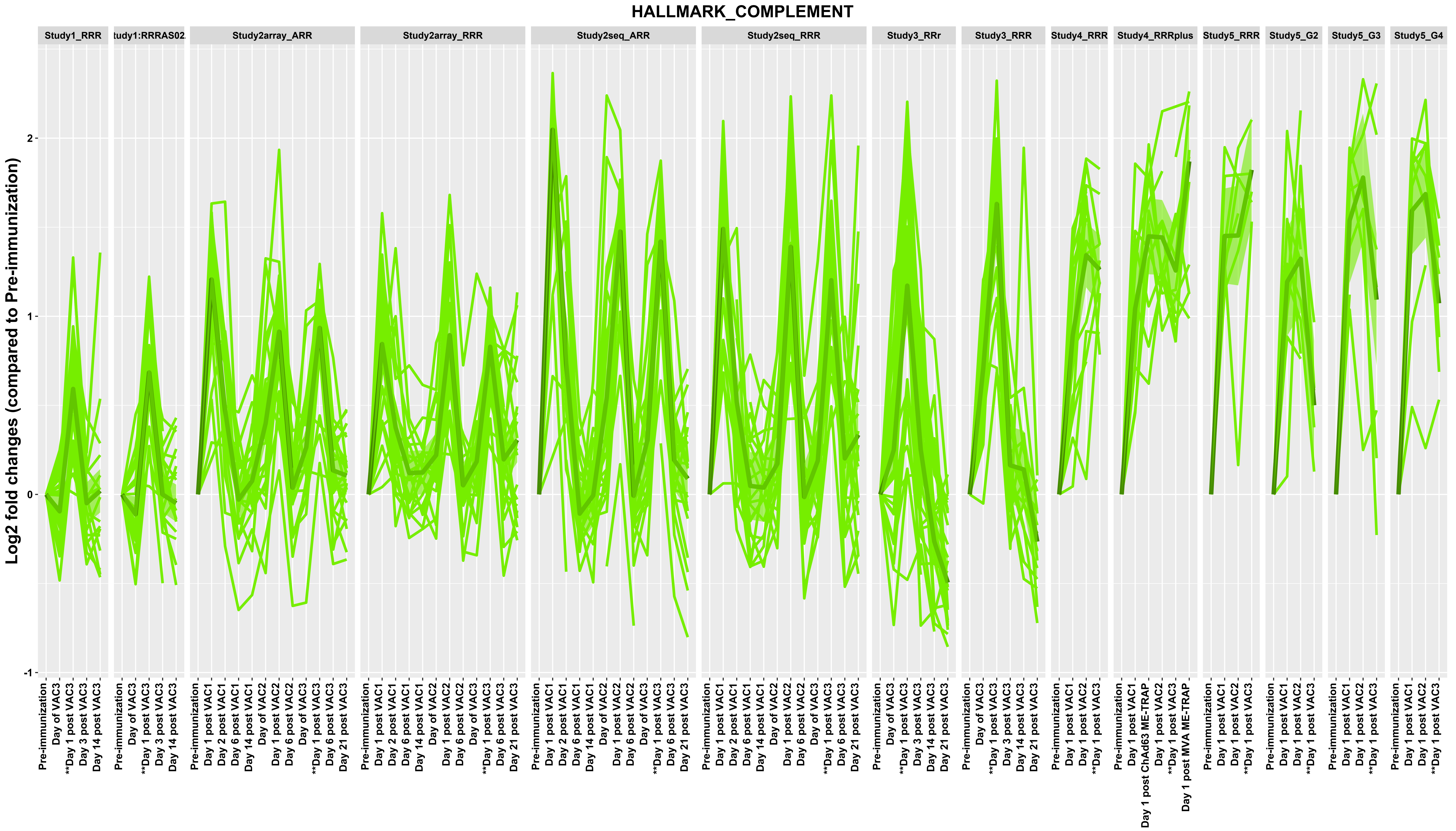

HALLMARK_COMPLEMENT
Log2 fold changes (compared to Pre-immunization)
Study1_RRR
tudy1:RRRAS02
Study2array_ARR
Study2array_RRR
Study2seq_ARR
Study2seq_RRR
Study3_RRr
Study3_RRR
Study4_RRR
Study4_RRRplus
Study5_RRR
Study5_G2
Study5_G3
Study5_G4
2
1
0
-1
Pre-immunization
Day of VAC3
**Day 1 post VAC3
Day 3 post VAC3
Day 14 post VAC3
Day 1 post VAC1
Day 2 post VAC1
Day 6 post VAC1
Day 14 post VAC1
Day of VAC2
Day 1 post VAC2
Day 6 post VAC2
Day 6 post VAC3
Day 21 post VAC3
Day 1 post ChAd63 ME-TRAP
Day 1 post MVA ME-TRAP

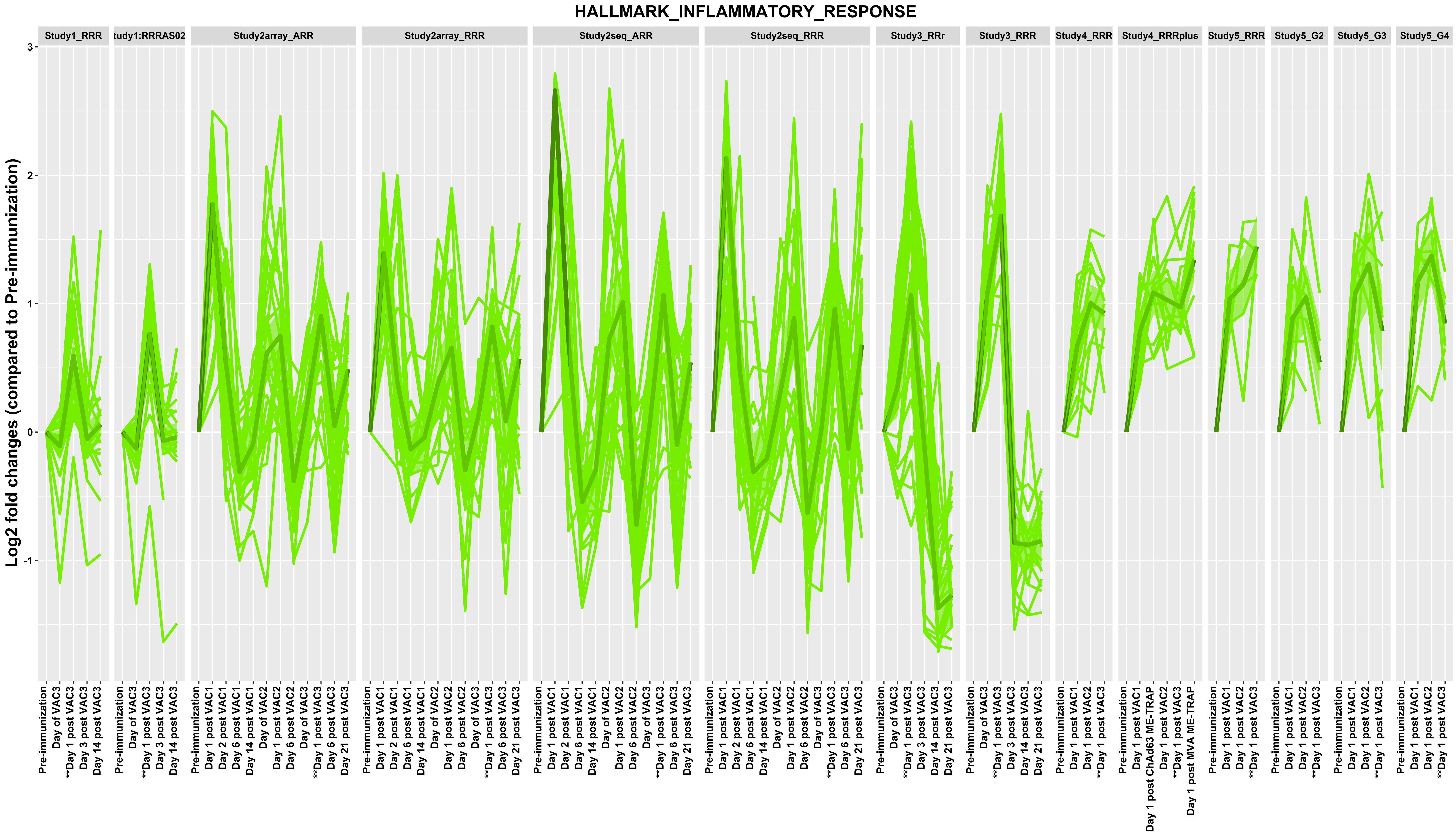
HALLMARK_INFLAMMATORY_RESPONSE
Study1_RRR
tudy1:RRRAS02
Study2array_ARR
Study2array_RRR
Study2seq_ARR
Study2seq_RRR
Study3_RRr
Study3_RRR
Study4_RRR
Study4_RRRplus
Study5_RRR
Study5_G2
Study5_G3
Study5_G4
Log2 fold changes (compared to Pre-immunization)
3
2
1
0
-1
Pre-immunization
Day of VAC3
**Day 1 post VAC3
Day 3 post VAC3
Day 14 post VAC3
Day 1 post VAC1
Day 2 post VAC1
Day 6 post VAC1
Day 14 post VAC1
Day of VAC2
Day 1 post VAC2
Day 6 post VAC2
Day 6 post VAC3
Day 21 post VAC3
Day 1 post ChAd63 ME-TRAP
**Day 1 post VAC3
Day 1 post MVA ME-TRAP

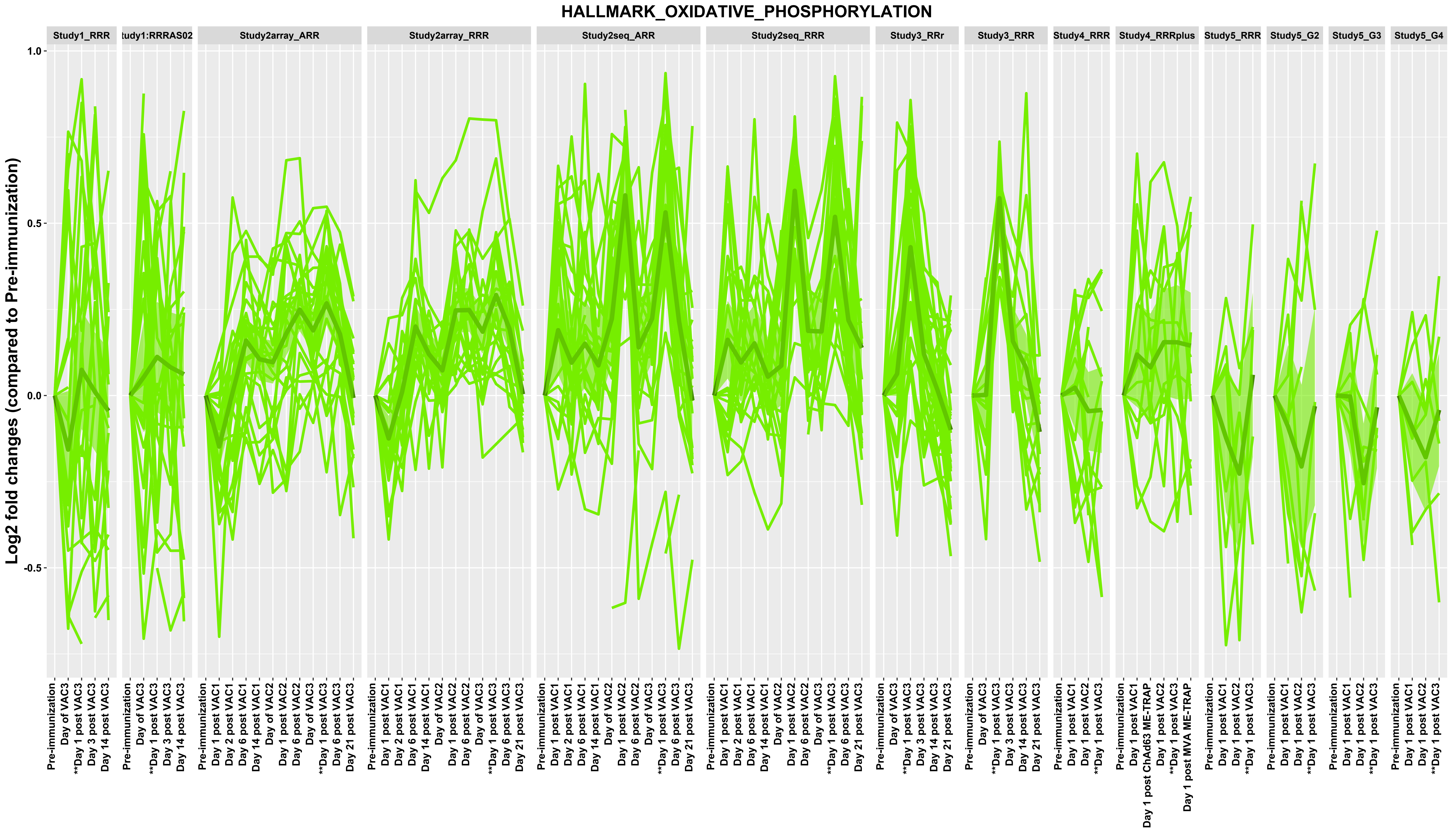

HALLMARK_OXIDATIVE_PHOSPHORYLATION
Log2 fold changes (compared to Pre-immunization)
1.0
0.5
0.0
-0.5
Study1_RRR
tudy1:RRRAS02
Study2array_ARR
Study2array_RRR
Study2seq_ARR
Study2seq_RRR
Study3_RRr
Study3_RRR
Study4_RRR
Study4_RRRplus
Study5_RRR
Study5_G2
Study5_G3
Study5_G4
Pre-immunization
Day of VAC3
**Day 1 post VAC3
Day 3 post VAC3
Day 14 post VAC3
Day 1 post VAC1
Day 2 post VAC1
Day 6 post VAC1
Day 14 post VAC1
Day of VAC2
Day 1 post VAC2
Day 6 post VAC2
Day 6 post VAC3
Day 21 post VAC3
Day 1 post ChAd63 ME-TRAP
**Day 1 post VAC2
Day 1 post MVA ME-TRAP

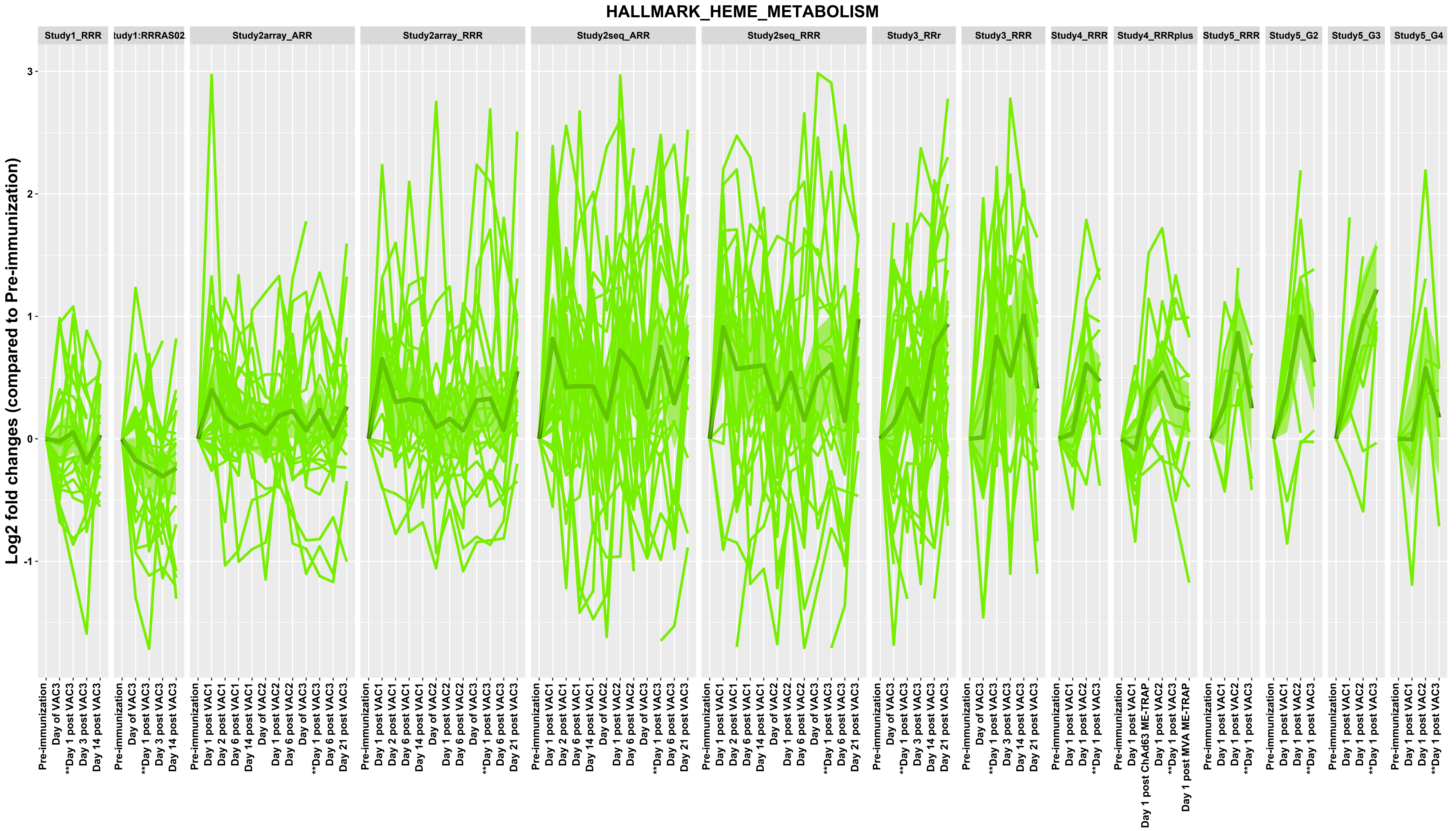
HALLMARK_HEME_METABOLISM
Log2 fold changes (compared to Pre-immunization)
Study1_RRR
tudy1:RRRAS02
Study2array_ARR
Study2array_RRR
Study2seq_ARR
Study2seq_RRR
Study3_RRr
Study3_RRR
Study4_RRR
Study4_RRRplus
Study5_RRR
Study5_G2
Study5_G3
Study5_G4
3
2
1
0
-1
Pre-immunization
Day of VAC3
**Day 1 post VAC3
Day 3 post VAC3
Day 14 post VAC3
Day 1 post VAC1
Day 2 post VAC1
Day 6 post VAC1
Day 14 post VAC1
Day of VAC2
Day 1 post VAC2
Day 6 post VAC2
Day 6 post VAC3
Day 21 post VAC3
**Day 1 post VAC3
Day 1 post ChAd63 ME-TRAP
Day 1 post MVA ME-TRAP

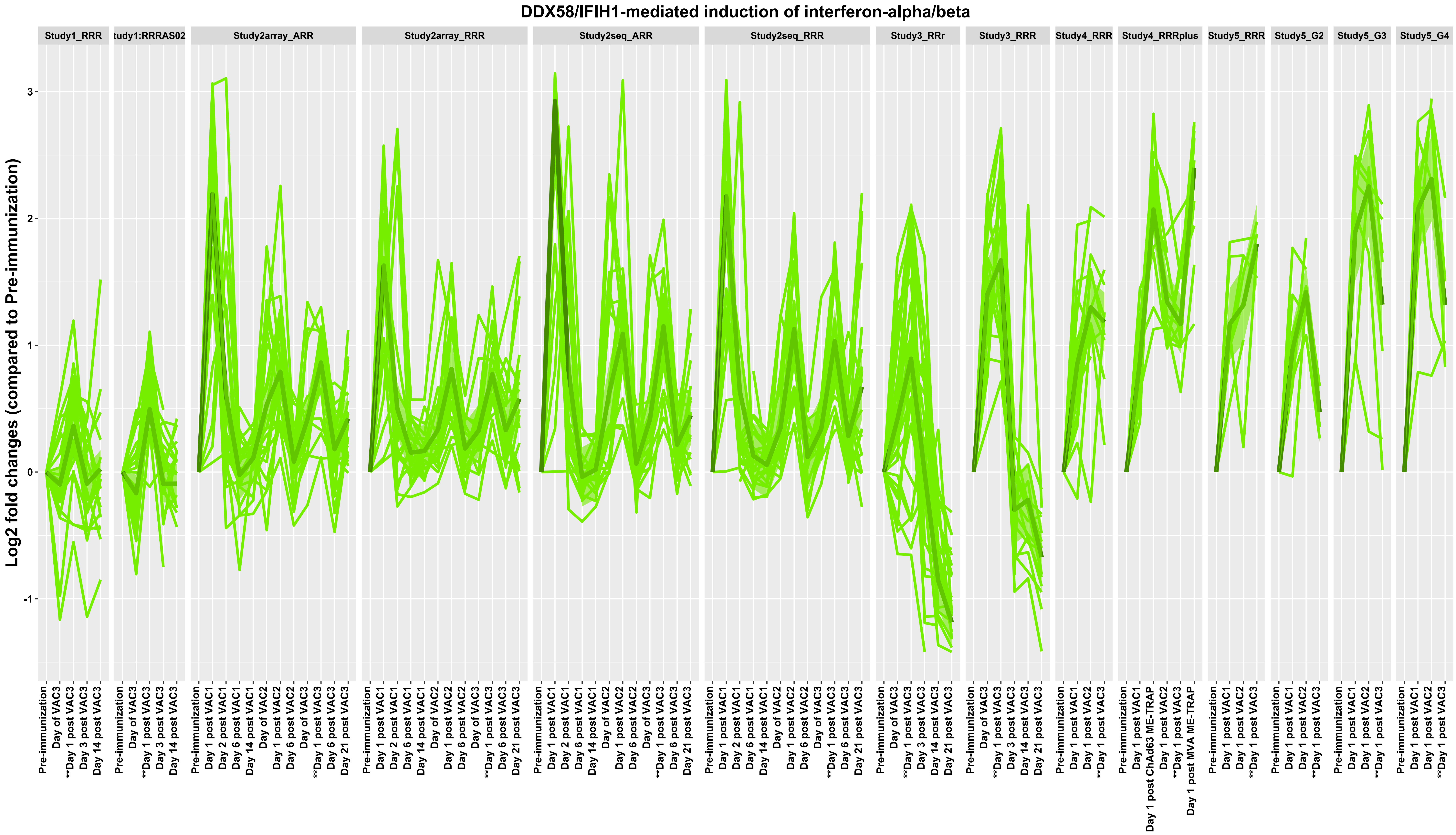
DDX58/IFIH1-mediated induction of interferon-alpha/beta
Study1_RRR
tudy1:RRRAS02
Study2array_ARR
Study2array_RRR
Study2seq_ARR
Study2seq_RRR
Study3_RRr
Study3_RRR
Study4_RRR
Study4_RRRplus
Study5_RRR
Study5_G2
Study5_G3
Study5_G4
Log2 fold changes (compared to Pre-immunization)
3
2
1
0
-1
Pre-immunization
Day of VAC3
**Day 1 post VAC3
Day 3 post VAC3
Day 14 post VAC3
Day 1 post VAC1
Day 2 post VAC1
Day 6 post VAC1
Day 14 post VAC1
Day of VAC2
Day 1 post VAC2
Day 6 post VAC2
Day 6 post VAC3
Day 21 post VAC3
**Day 1 post VAC2
Day 1 post ChAd63 ME-TRAP
**Day 1 post VAC3
Day 1 post MVA ME-TRAP

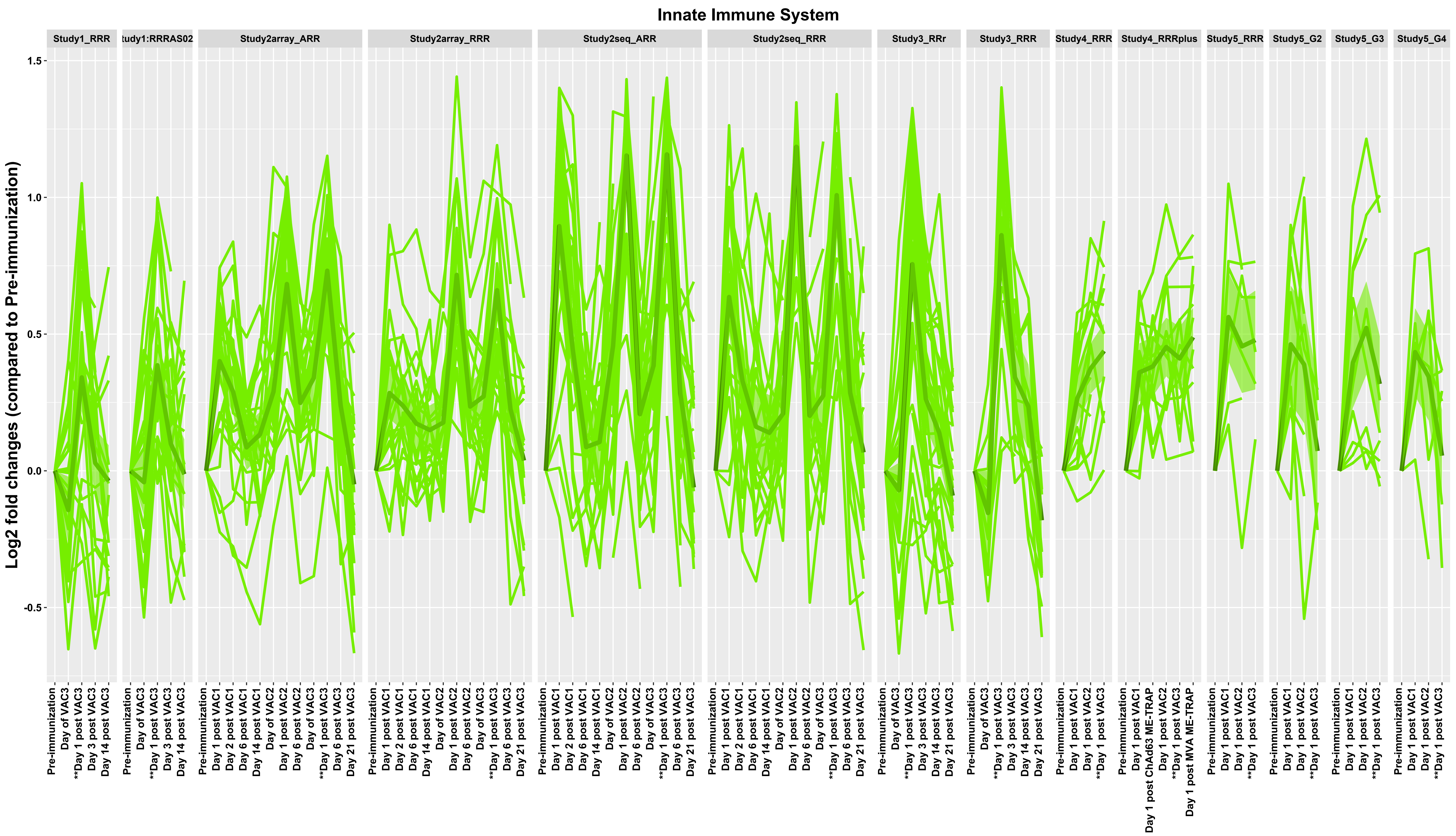
Innate Immune System
Study1_RRR
tudy1:RRRAS02
Study2array_ARR
Study2array_RRR
Study2seq_ARR
Study2seq_RRR
Study3_RRr
Study3_RRR
Study4_RRR
Study4_RRRplus
Study5_RRR
Study5_G2
Study5_G3
Study5_G4
Log2 fold changes (compared to Pre-immunization)
1.5
1.0
0.5
0.0
-0.5
Pre-immunization
Day of VAC3
**Day 1 post VAC3
Day 3 post VAC3
Day 14 post VAC3
Day 1 post VAC1
Day 2 post VAC1
Day 6 post VAC1
Day 14 post VAC1
Day of VAC2
Day 1 post VAC2
Day 6 post VAC2
Day 6 post VAC3
Day 21 post VAC3
Day 1 post ChAd63 ME-TRAP
Day 1 post MVA ME-TRAP

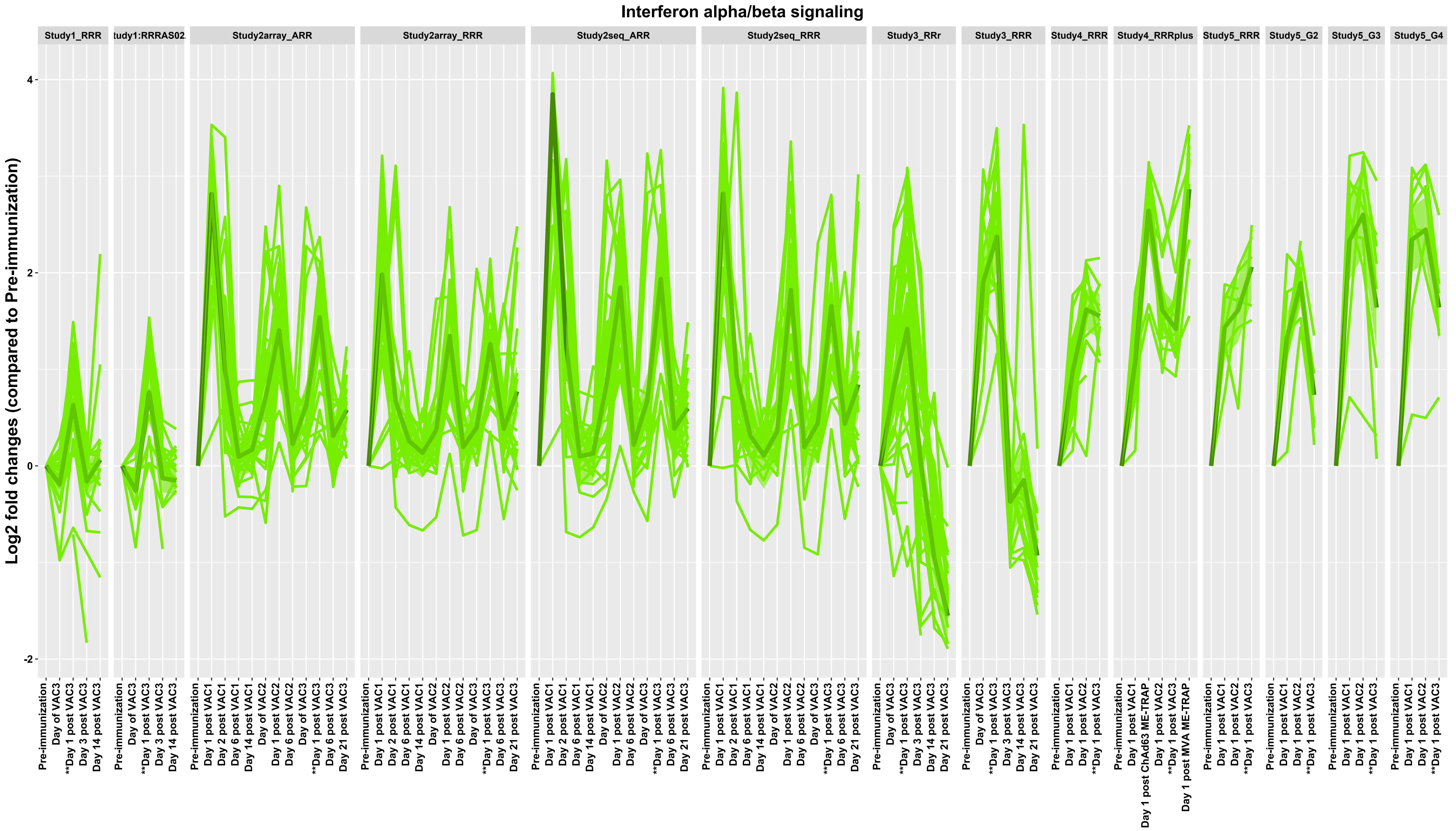

Interferon alpha/beta signaling
Study1_RRR
tudy1:RRRAS02
Study2array_ARR
Study2array_RRR
Study2seq_ARR
Study2seq_RRR
Study3_RRr
Study3_RRR
Study4_RRR
Study4_RRRplus
Study5_RRR
Study5_G2
Study5_G3
Study5_G4
Log2 fold changes (compared to Pre-immunization)
4
2
0
-2
Pre-immunization
Day of VAC3
**Day 1 post VAC3
Day 3 post VAC3
Day 14 post VAC3
Day 1 post VAC1
Day 2 post VAC1
Day 6 post VAC1
Day 14 post VAC1
Day of VAC2
Day 1 post VAC2
Day 6 post VAC2
Day 6 post VAC3
Day 21 post VAC3
Day 2 post VAC3
Day 1 post ChAd63 ME-TRAP
Day 1 post MVA ME-TRAP
**Day 1 post VAC2

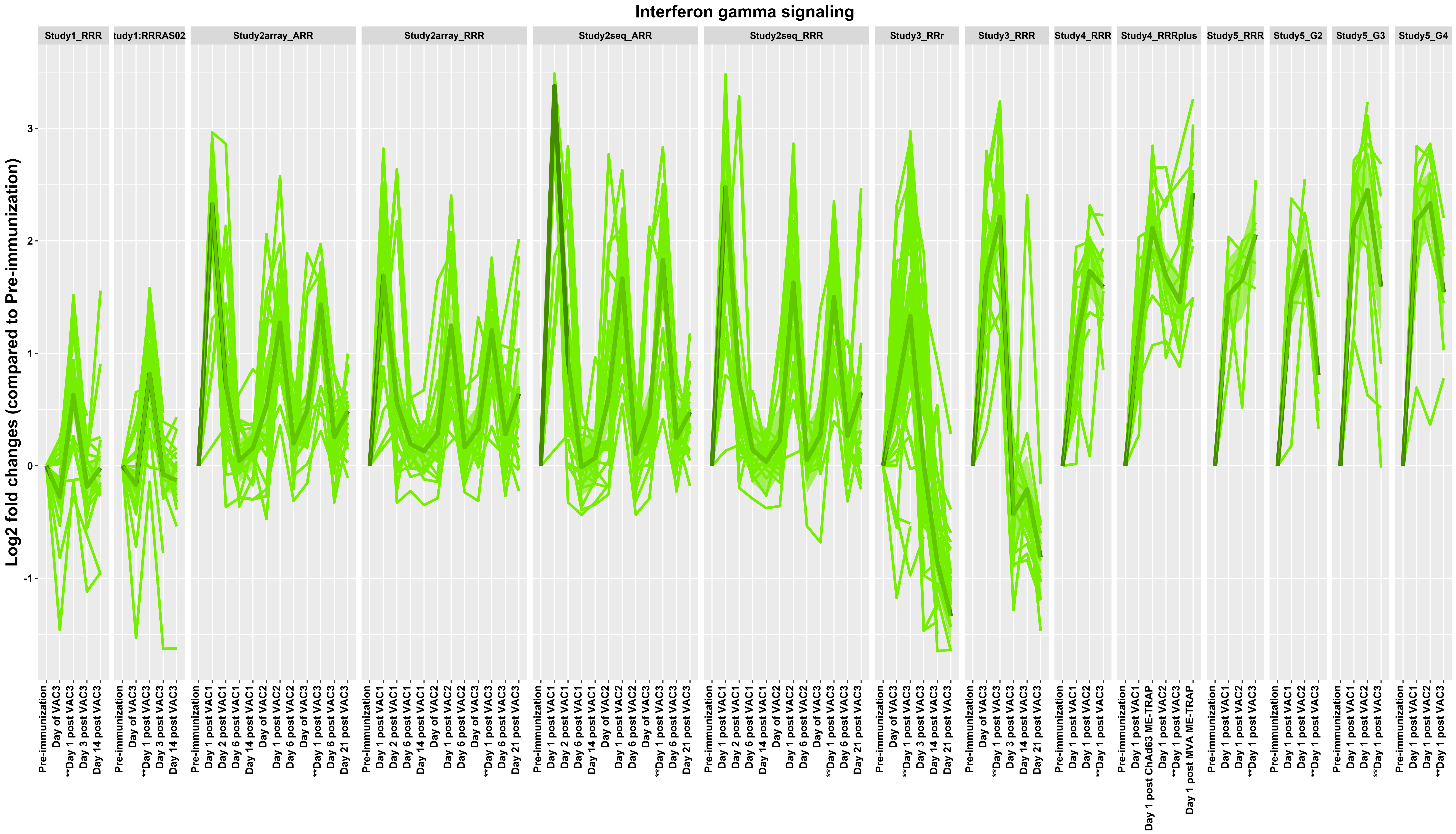

Interferon gamma signaling
Study1_RRR
tudy1:RRRAS02
Study2array_ARR
Study2array_RRR
Study2seq_ARR
Study2seq_RRR
Study3_RRr
Study3_RRR
Study4_RRR
Study4_RRRplus
Study5_RRR
Study5_G2
Study5_G3
Study5_G4
Log2 fold changes (compared to Pre-immunization)
3
2
1
0
-1
Pre-immunization
Day of VAC3
**Day 1 post VAC3
Day 3 post VAC3
Day 14 post VAC3
Day 1 post VAC1
Day 2 post VAC1
Day 6 post VAC1
Day 14 post VAC1
Day of VAC2
Day 1 post VAC2
Day 6 post VAC2
Day 6 post VAC3
Day 21 post VAC3
Day 1 post ChAd63 ME-TRAP
Day 1 post MVA ME-TRAP

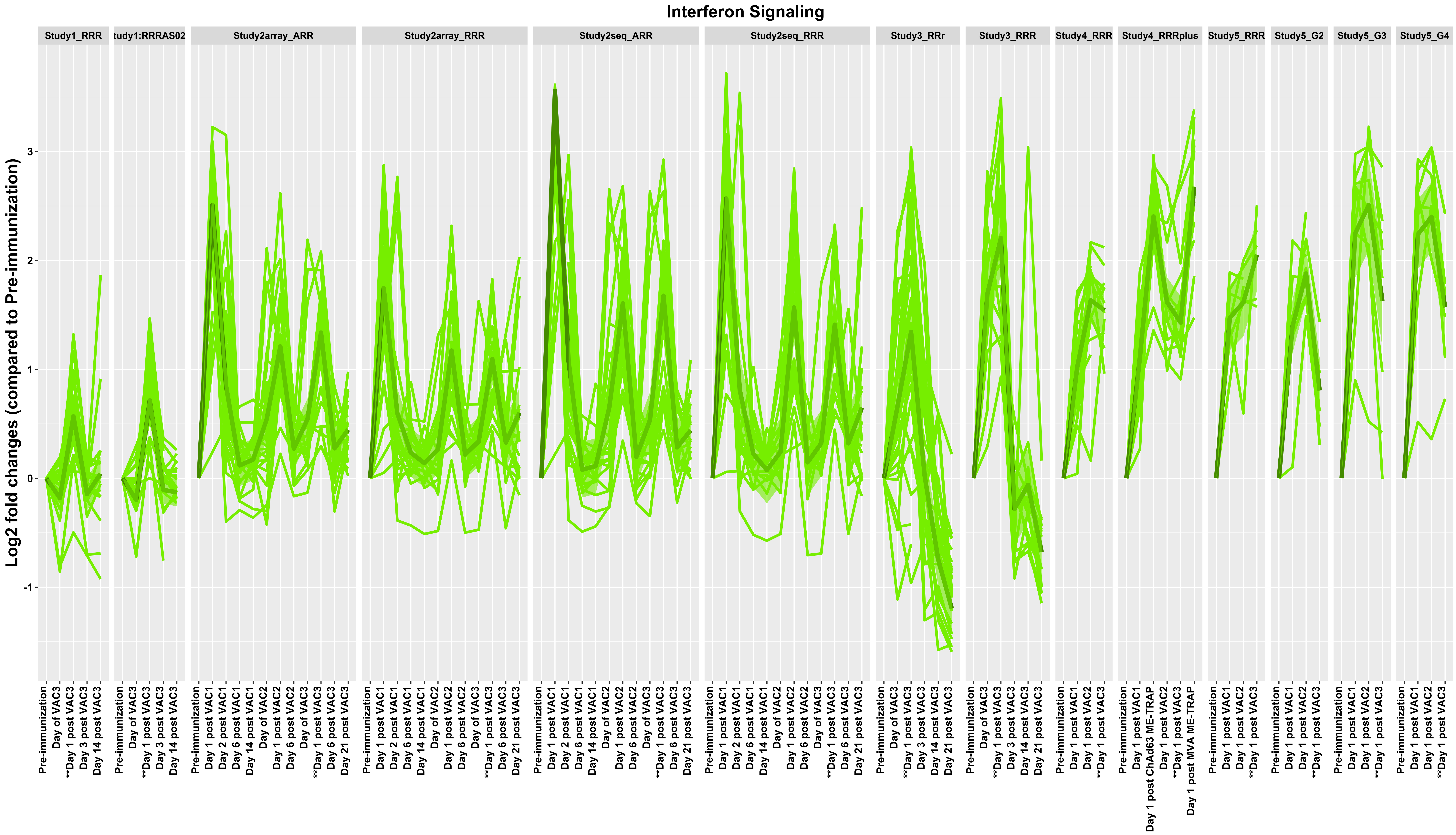
Interferon Signaling
Log2 fold changes (compared to Pre-immunization)
Study1_RRR
tudy1:RRRAS02
Study2array_ARR
Study2array_RRR
Study2seq_ARR
Study2seq_RRR
Study3_RRr
Study3_RRR
Study4_RRR
Study4_RRRplus
Study5_RRR
Study5_G2
Study5_G3
Study5_G4
3
2
1
0
-1
Pre-immunization
Day of VAC3
**Day 1 post VAC3
Day 3 post VAC3
Day 14 post VAC3
Day 1 post VAC1
Day 2 post VAC1
Day 6 post VAC1
Day 14 post VAC1
Day of VAC2
Day 1 post VAC2
Day 6 post VAC2
Day 6 post VAC3
Day 21 post VAC3
Day 1 post ChAd63 ME-TRAP
Day 1 post MVA ME-TRAP
**Day 1 post VAC3

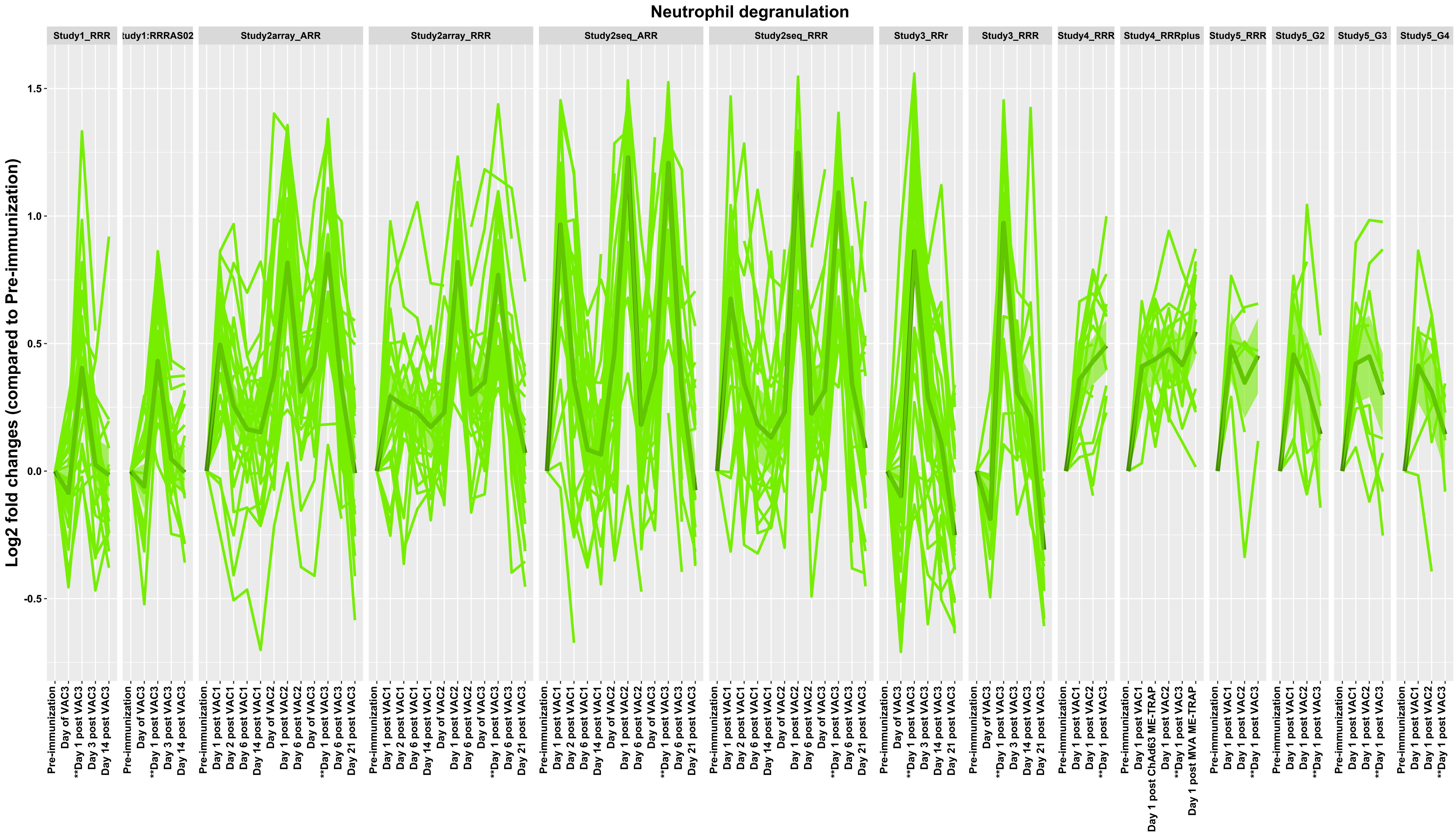
Neutrophil degranulation
Log2 fold changes (compared to Pre-immunization)
Study1_RRR
tudy1:RRRAS02
Study2array_ARR
Study2array_RRR
Study2seq_ARR
Study2seq_RRR
Study3_RRr
Study3_RRR
Study4_RRR
Study4_RRRplus
Study5_RRR
Study5_G2
Study5_G3
Study5_G4
1.5
1.0
0.5
0.0
-0.5
Pre-immunization
Day of VAC3
**Day 1 post VAC3
Day 3 post VAC3
Day 14 post VAC3
Day 1 post VAC1
Day 2 post VAC1
Day 6 post VAC1
Day 14 post VAC1
Day of VAC2
Day 1 post VAC2
Day 6 post VAC2
Day 6 post VAC3
Day 21 post VAC3
Day 1 post ChAd63 ME-TRAP
Day 1 post MVA ME-TRAP

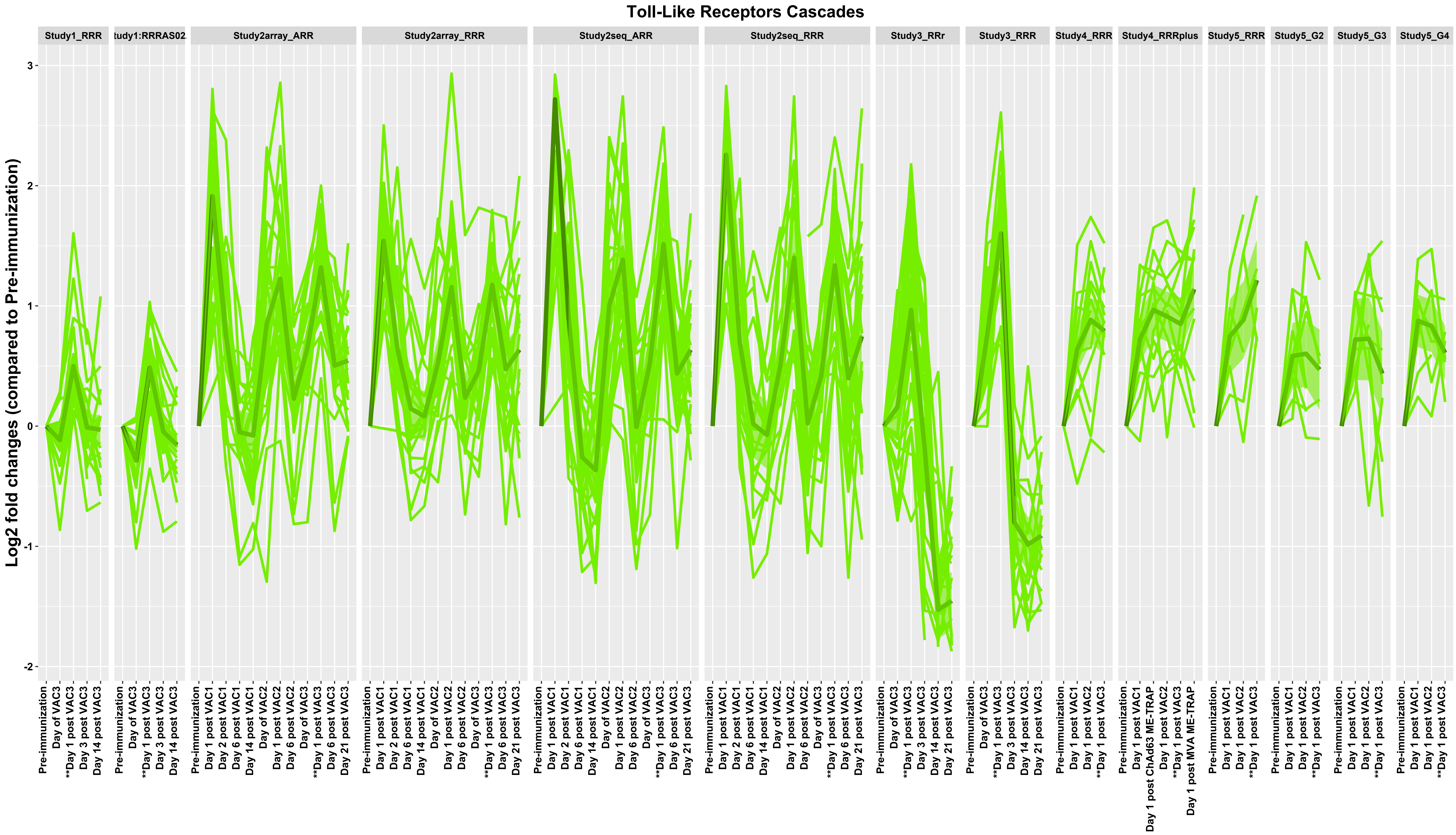
Toll-Like Receptors Cascades
Study1_RRR
tudy1:RRRAS02
Study2array_ARR
Study2array_RRR
Study2seq_ARR
Study2seq_RRR
Study3_RRr
Study3_RRR
Study4_RRR
Study4_RRRplus
Study5_RRR
Study5_G2
Study5_G3
Study5_G4
Log2 fold changes (compared to Pre-immunization)
3
2
1
0
-1
-2
Pre-immunization
Day of VAC3
**Day 1 post VAC3
Day 3 post VAC3
Day 14 post VAC3
Day 1 post VAC1
Day 2 post VAC1
Day 6 post VAC1
Day 14 post VAC1
Day of VAC2
Day 1 post VAC2
Day 6 post VAC2
Day 6 post VAC3
Day 21 post VAC3
Day 1 post ChAd63 ME-TRAP
Day 1 post MVA ME-TRAP

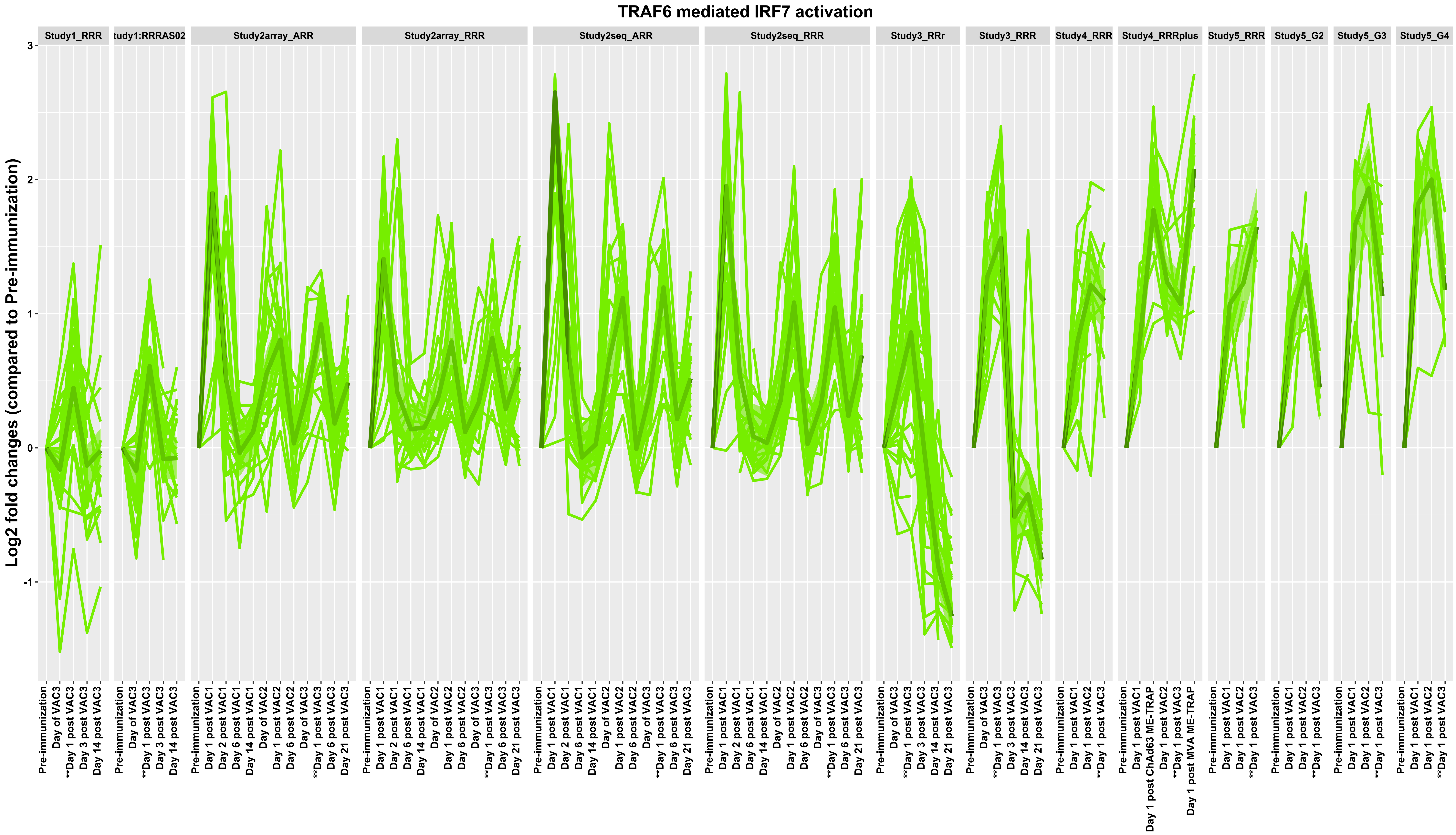
TRAF6 mediated IRF7 activation
Study1_RRR
tudy1:RRRAS02
Study2array_ARR
Study2array_RRR
Study2seq_ARR
Study2seq_RRR
Study3_RRr
Study3_RRR
Study4_RRR
Study4_RRRplus
Study5_RRR
Study5_G2
Study5_G3
Study5_G4
Log2 fold changes (compared to Pre-immunization)
3
2
1
0
-1
Pre-immunization
Day of VAC3
**Day 1 post VAC3
Day 3 post VAC3
Day 14 post VAC3
Day 1 post VAC1
Day 2 post VAC1
Day 6 post VAC1
Day 14 post VAC1
Day of VAC2
Day 1 post VAC2
Day 6 post VAC2
Day 6 post VAC3
Day 21 post VAC3
Day 1 post ChAd63 ME-TRAP
Day 1 post MVA ME-TRAP
