# Supplementary material for: The Ratiometric Transcript Signature MX2/GPR183 Is Consistently Associated With RTS,S-Mediated Protection Against Controlled Human Malaria Infection
Source: Front Immunol. 2020 Apr 28;11:669. doi: 10.3389/fimmu.2020.00669 (PMC7199517; doi:10.3389/fimmu.2020.00669)
Supplement: Supplementary file 12 [file Data_Sheet_2.docx]

**Supplementary Methods**

**Supplementary Methods #1: RNA-Seq data generation and microarray normalization.**

***1.1 Generation of PBMC RNA-Seq data for Study 2 and Study 3 and dataset normalizations*.** PBMC RNA-Seq data for Study 2 and Study 3 were generated by Expression Analysis Inc. using total RNA that was extracted from PBMCs lysed in TriZol. Libraries were prepared using the TruSeq Stranded protocol and the sequencing strategy was at least 30million 50bp paired-end reads per sample. PBMC RNA-Seq data for Study 2 and Study 3 were processed using standard methods as described before [26]. Read pairs were preprocessed using in-house scripts that adjust base calls with phred scores <5 to ‘N’ and remove read pairs for which either end has fewer than 30 unambiguous base calls, a method that also indirectly removes pairs containing mostly adaptor sequences. Read pairs were aligned to the human genome (hg19) using STAR (v2.3.1d) [27]. Mapped read pairs were assigned to genes by collapsing all transcripts into a single gene model and then counting the number of reads that fully overlap the resulting exons using htseq (v. 0.6.0) [28] with strict intersection and including strand information. Gene models for protein-coding genes were downloaded Ensembl (GRCh37.74). Reads that mapped to multiple locations were only counted once and those mapping to ambiguous regions were excluded. Log2-transformed values of counts normalized by adjusted library counts were computed using the cpm function of the edgeR package [29].

***1.2 Microarray data normalization*.** To normalize the Affymetrix-based Study 1 and Study 2 PBMC microarray data, .CEL files were downloaded from Array Express or GEO and then normalized using the *justRMA* function in the Bioconductor package *Affy* with the Ensembl Gene-based CDF for the HG-U133_Plus_2 platform downloaded from the BrainArray website (<http://mbni.org/customcdf/20.0.0/ensg.download/hgu133plus2hsensgcdf_20.0.0.zip>). This normalization scheme provides a single expression-level estimate for each Ensembl Gene ID. Gene IDs were mapped to gene symbols using Ensembl Biomart. CEL files for Study 1 and Study 2 were normalized together.

To normalize Illumina HumanHT-12 V4.0 expression beadchip microarray data for Study 4 and Study 5, raw data for both studies were merged, probes were background corrected using negative control probes (negqc) followed by quantile normalization. Outliers and mislabeled samples were identified and removed for Study 4 and Study 5 individually, and all probes with detection p-values greater than 0.05 in more than 95% of the samples were removed, as were probes with insertions, more than 2 mismatches, or mapping lengths less than 40 bases to Gencode v23, RefSeq (refMrna.fa) or GenBank (mrna.fa). Representative probes for transcripts assayed by multiple probes were selected on the basis of maximizing the median absolute deviation from the median across samples.

**Supplementary Methods #2: identification of coherent transcriptional modules**.

Candidate blood transcriptional modules were assembled from published transcriptomics studies [13, 30], REACTOME Immune networks [31], and MSIGDB HALLMARKS [32]. Module definitions were filtered to retain genes that were represented in all transcriptomics platforms analyzed. The coherence in gene expression for each module within each study was determined in four steps: (1) computing the 25% trimmed mean of median-centered expression values for all genes within a module within a given study using all available pre-challenge time points. (2) Computing the Spearman rank correlation between the expression profile of all genes in that module and the trimmed mean profile was computed for each study. (3) Genes that had Spearman rank correlations > 0.55 with the trimmed mean profile for all studies were retained as the coherent gene set for that particular module. (4) Only modules containing at least 5 genes after this filtering step were retained for subsequent analysis – leaving 79 coherent transcriptional modules (Supplementary Table 4, Supplementary Figure 2).

**Supplementary Methods #3: logistic regression modeling to identify transcriptional signatures that consistently discriminate protected from non-protected recipients of RRR regimen RTS,S.** Briefly, logistic regression (LR) was used to model challenge outcome (protected=1 or not protected=0) as a function of individual transcriptional readouts and a “STUDY” term. The STUDY term served as an additive categorical constant for each Study that accounts for gross expression intensity differences in individual genes arising from differences in expression platform (RNA-Seq, Affymetrix microarray, or Illumina microarray), source of RNA (PBMC vs. whole blood), or other additive technical variables. The statistical significance of the influence of transcriptional readouts on challenge outcome was assessed using Chi-squared tests comparing LR models containing the categorical STUDY term to LR models containing both the transcriptional readout and the STUDY term. In the formulae below, the transcriptional readout is represented as “TRANSCRIPT” and is Log2 fold-change comparing Day 1 post 3^rd^ vaccination (usually D57 in a given study) to pre-vaccination expression levels. The transcriptional readouts analyzed included 79 Log2 (blood transcriptional modules); 10,657 genes Log2(individual transcripts); 3,081 Log2(ratios between blood transcriptional modules); 841,903 Log2(ratios between individual transcripts and blood transcriptional modules); and 2727 Log2(ratios between individual transcripts). To assess the statistical significance of each transcriptional variable for discriminating protected from non-protected participants, chi-squared tests were used to compare the following two models:

Full model: PROTECTION ~ TRANSCRIPT + STUDY

Reduced model: PROTECTION ~ STUDY

Note that the full model in this case intentionally does not include an interaction term between TRANSCRIPT and STUDY. This means that we are seeking transcriptional variables that associated with protection status in a consistent manner across studies. As mentioned in the main text, RNA-Seq-based and microarray based transcriptomes for Study 2 were treated in separate analyses and the *worst performance* for either platform (largest p-value and smallest ROC) was retained - ensuring that both platforms reached the reported significance and discrimination thresholds. The Benjamini-Hochberg FDR algorithm was used to address the multiple testing issues inherent in this very large number of tests.

This analysis identified 2 genes (Supplementary Table 5) and 241 gene/module pairs (Supplementary Table 6) that were significantly associated with protection (p<0.0025, FDR=20.1%) and consistently discriminated between protected and non-protected participants in the RRR arms of all 5 trials (ROC AUC>0.65). The 241 gene/module pairs included 121 unique genes, and 48 unique modules. The 241 gene/module ratios were extended to 2727 transcript/transcript ratios using the cognate module definitions. Re-applying the same filtering criteria that was used for the original selection of 241 gene/module ratios yielded the final set of 247 transcript/transcript ratios (Supplementary Table 7).

**Supplementary Methods #4: logistic regression analysis testing transcript/transcript ratios for discrimination of protected from non-protected recipients of alternative regimen RTS,S.**

We hypothesized that the protection-associated signatures identified for RRR regimen RTS,S would be applicable to some, but not necessarily all, of the alternative RTS,S regimens that were evaluated in the different studies. Mathematically, the possibility that the protection-associated signatures would be applicable to a subset of alternative RTS,S regimens can be captured by extending the “Full” LR model above to include interactions between TRANSCRIPT and STUDY. The interpretation is that, for some alternative regimen studies, the interaction term negates or reverses the influence that the TRANSCRIPT variable has on vaccine-mediated protection, while allowing that influence to be applicable for other alternative regimens. As before, Chi-squared tests were used to compare two models:

Full interaction model: PROTECTION ~ TRANSCRIPT + STUDY + TRANSCRIPT:STUDY

Reduced model: PROTECTION ~ STUDY

In contrast to Supplementary Methods #3 (above), the comparison is between the Full interaction model with both the linear TRANSCRIPT and TRANSCRIPT:STUDY terms and a model with only the STUDY term. The interpretation of the resulting p-value is whether the TRANSCRIPT variable has a non-random influence on protection status in some form, which could occur as a linear variable that is applicable across all studies or as a linear variable that is relevant for only a subset of regimens. Including the TRANSCRIPT:STUDY interaction term in the model markedly increases the degrees of freedom and increases the stringency of the statistical testing.

Please not that transcriptional data for the alternative regimen in Study #4 (“RRRplus”) was only available for protected participants (Supplementary Table 2). For this reason, Study #4 was not included in the alternative regimen analyses. Although three distinct alternative vaccination regimens were evaluated in Study #5 (G2, G3, and G4), the number volunteers vaccinated with each were comparatively small. For this reason, the Study #5 alternative regimens were treated as a single group in the logistic regression with the interaction terms.

Of the 247 discriminatory transcript/transcript ratio fold-changes identified by the analysis of RRR regimen RTS,S cohorts (Supplementary Table 7), nine transcript/transcript ratio fold-changes exhibited significant differences between the full interaction model and reduced model for discriminating protected from non-protected recipients of alternative regimen RTS,S (p<0.002, FDR=6%) (Supplementary Table 8).

Obtaining significant p-values resulting from the Full interaction model and Reduced model comparison above does not explicitly indicate in which particular alternative regimens the TRANSCRIPT variable is associated with protection status. To obtain this information, we performed a second modeling step involving pairs of alternative regimens as a means to increase statistical power compared to analysis of the alternative regimen volunteer populations individually (which generally involved small numbers of volunteers). We performed LR modeling of all possible pairs of alternative regimen RTS,S arms, but now employing a Full vs. reduced model comparison that did not include interaction terms. In this analysis, we treated each alternative regimen separately (rather than grouping the distinct regimens from Study #5), and therefore replaced the “STUDY” term with “STUDYARM” in the models. As before, Chi-squared tests were used to compare two models, and detecting significant impact of TRANSCRIPT on protection for at least one of these pairwise combinations would provide evidence that the TRANSCRIPT signature has some generalized value and is not restricted to a single alternative regimen or to the RRR regimen.

Full model: PROTECTION ~ STUDYARM + TRANSCRIPT

Reduced model: PROTECTION ~ STUDYARM

As above, RNA-Seq-based and microarray-based transcriptomes for Study 2 were modeled separately and the worst performance for either platform was retained (ensuring that both platforms reached the specified significance and discrimination thresholds). Given that transcriptional data was available for only one non-protected volunteer for G2 from Study #5, this particular alternative regimen was not included in this second alternative regimen modeling analysis. Therefore, the above modeling was performed for ten (^5^C_2_) pairwise subsets of 5 alternative regimens (Study #1 RRR_AS02A, Study #2 ARR, Study #3 RRr, Study #5 G3, and Study #5 G4).

Of the nine transcript/transcript ratio fold-changes that exhibited significant differences between the full interaction model and reduced model, six transcript/transcript ratios exhibited nominally significant (p<0.05) discrimination between protected and non-protected volunteers for at least two pairwise sets of alternative RTS,S regimens (Supplementary Table 8). The top scoring ratio among these was Log2(MX2/GPR183), which was taken as the final signature for subsequent analyses.

Visualization of discrimination between protected and non-protected volunteers by the Log2(MX2/GPR183) ratio for RRR RTS,S regimen and alternative regimen recipients (Supplementary Figure 5) demonstrated that consistent trends for this ratio being expressed higher in protected volunteers was observed for all RRR RTS,S regimen recipients and recipients of ARR (Study #2), RRr (Study #3), G2 (Study #5), and G3 (Study #5) alternative regimens but not RRR_AS02A (Study #1) and G4 (Study #5) alternative regimens. For this reason, scatterplot visualizations (Figure 1 B & C) and ROC curves (Supplementary Figures 6-7) did not include RRR_AS02A and G4 alternative regimen groups. Note that for scatterplot visualizations (Figure 1 B & C), the gene fold-change values for MX2 and GPR183 from multiple trials and platforms were adjusted using the coefficients of the STUDY term obtained from the above LR models without interactions.

**Supplementary Methods #5: logistic regression analysis testing for improved discrimination between protected and non-protected recipients of RTS,S using MX2/GPR183 in combination with anti-CSP titers.** LR modeling used to determine whether the Log2(MX2/GPR183) ratio fold-change was redundant with anti-CSP titers for discriminating protected from non-protected volunteers or whether Log2(MX2/GPR183) provided information that complemented anti-CSP titers for discriminating these groups. For each study with available antibody data (Study 2-5), anti-CSP (repeat region) titers from the Day of Challenge (DoC) or most proximal pre-challenge timepoint were first log10 transformed. These log10-transformed data were then Z-score standardized by subtracting the mean value from each study and then dividing by the standard deviation for each study. All of the vaccine arms (RRR and alternative arms) within a given study were transformed together, preserving differences between arms. As before, Chi-squared tests were used to compare two models:

Full model: PROTECTION ~ ANTI-CSP + STUDY + TRANSCRIPT

Reduced model: PROTECTION ~ ANTI-CSP + STUDY

Where “ANTI-CSP” indicates the Z-score transformed anti-CSP titers and “TRANSCRIPT” specifically represents the Log2(MX2/GPR183) ratio fold-change. As above, RNA-Seq-based and microarray-based transcriptomes for Study 2 were modeled separately and the worst performance for either platform was retained (ensuring that both platforms reached the specified significance and discrimination thresholds).

The comparisons between the Full and Reduced model were performed separately for recipients of RRR RTS,S vaccination and for recipients of alternative regimens. As anti-CSP titers were not available for Study #1, data for volunteers from Study #1 were not included in the analysis. Data for recipients of the G4 (Study #5) alternative regimen were not included in the analysis given that the MX2/GPR183 ratio did not discriminate protected from non-protected volunteers in this group in a manner that was consistent with the other regimens (Supplementary Figure 5). Lastly, data for recipients of the G2 and G3 alternative regimens (Study #5) were treated as a single group. For scatterplot visualizations (Figure 2 B & C), the MX2/GPR183 ratio was adjusted using the STUDY coefficient obtained from the above LR models.
